# Small dense low density lipoprotein predominance in patients with type 2 diabetes mellitus using Mendelian randomization

**DOI:** 10.1371/journal.pone.0298070

**Published:** 2024-02-08

**Authors:** Fengyi Zhang, Yufeng Zhang, Jiayi Zhang, Xin Wang, Yujie Li, Wenbo Wang

**Affiliations:** 1 Shandong University of Traditional Chinese Medicine, Jinan, Shandong Province, People’s Republic of China; 2 Second Affiliated Hospital of Shandong University of Traditional Chinese Medicine, Jinan, Shandong Province, People’s Republic of China; Shahid Beheshti University of Medical Sciences, ISLAMIC REPUBLIC OF IRAN

## Abstract

**Background:**

Patients with T2DM often suffer from CVD-related complications, significantly impacting morbidity and mortality rates. The upsurge in CVD prevalence among them is partly linked to sd LDL particles. Understanding the mechanisms behind elevated sd LDL levels is critical for preventing and managing cardiovascular complications in diabetes.

**Methods:**

MR was employed to identify instrumental variables and establish causality, exploring underlying mechanisms.

**Results:**

Notably, T2DM itself, insulin resistance, and fasting glucose seemingly do not directly impact sd LDL levels. Instead, the presence of T2DM or insulin resistance, leading to reduced HDL cholesterol or elevated TG levels, directly contributes to subsequent sd LDL increases, indicating a comprehensive mediating effect. While LDL cholesterol levels correlate positively with sd LDL, they appear unaffected by T2DM or insulin resistance. Importantly, hypertension induced by T2DM or insulin resistance exhibits a positive effect on sd LDL reversal. Unlike T2DM or insulin resistance, blood glucose levels show no significant impact on all processes.

**Conclusions:**

It is hoped that these insights might influence the treatment of patients with diabetes and the management of blood parameters in clinical practice. Examining the effect of T2DM or insulin resistance on sd LDL within HDL cholesterol and triglycerides pathways might provide valuable insights for targeted cardiovascular treatments. Additionally, the study’s exploration of the potential positive effects of elevated blood pressure on sd LDL reversal may introduce novel considerations for blood pressure management in patients with diabetes.

## Introduction

T2DM, characterized by its non-insulin-dependent nature, is a complex condition that is frequently diagnosed without a full understanding of its underlying causes. Essentially, it represents a confluence of diverse contributing factors [1]. According to the International Diabetes Federation, the estimated number of diabetes cases was reported at 463 million in 2019 and is projected to increase to 700 million by 2045 [2]. And over 90% of patients with diabetes are diagnosed with T2DM [3]. It’s noteworthy that patients with diabetes often experience various associated complications. More than two-thirds of them presently present with hypertension [4]. Poorly controlled diabetes often leads to heightened blood lipid levels, exacerbating the prevalence of hyperlipidemia in these individuals compared to those without diabetes [5, 6]. Furthermore, CVD, a prevalent comorbidity of T2DM, stands as a significant contributor to morbidity, disability, and mortality among individuals with T2DM [6]. A large cohort study involving 435,369 patients diagnosed with T2DM and followed over a period of 4.6 years revealed that CVD mortality rates were 17.15 per 1000 patient-years among those with T2DM, whereas controls exhibited a rate of 12.86 per 1000 patient-years [7]. And as research into diabetes and its associated CVD progresses, the specific mechanisms behind their development remain still unclear [8], but studies have found that sd LDL may plays an important role in this process [9].

LDL plays a pivotal role in both the initiation and progression of atherosclerosis which stands as the pathological foundation of CVD [10]. It can be fractionated into large buoyant and small dense particles based on size and density [11]. Multiple prospective and case-control studies as well as clinical evidence have shown that sd LDL are more atherogenic, that sd LDL levels are more associated with CHD than LDL cholesterol and lb-LDL concentrations, and that the superiority of sd LDL increases the risk of coronary artery disease threefold [11–16]. Past studies have revealed elevated sd LDL concentrations in patients experiencing incident CHD, myocardial infarction, stroke, and overall CVD [13]. Markers such as sd LDL particles and sd LDL cholesterol content can additionally evaluate the risk of CVD. A review [9] which has been conducted to consolidate the potential impact of hypolipidaemic drugs, including statins and fibrates, as well as hypoglycaemic drugs such as peroxisome proliferator-activated receptor γ ligands (glitazones and thiazolidinediones), on sd LDL to some extent, believes that adjusting treatments to modulate the quantity and distribution of small LDL may potentially reduce the risk of CVD.

Significantly, the study observed elevated levels of sd LDL in prediabetic individuals in contrast to young adults with normal blood glucose levels [17]. And patients with T2DM exhibit an elevation in the proportion of sd LDL, at least twofold [15, 18, 19]. These findings indicate a potentially significant new role for sd LDL in the link between T2DM and CVD. While some studies have indicated that sd LDL may result from hypertriglyceridemia and insulin resistance [20], in the case of patients with diabetes, various complex factors, including abnormal blood glucose, blood pressure, blood lipid levels, and diabetes itself, could potentially impact the level of sd LDL. However, a comprehensive and exhaustive explanation for these factors has not been fully outlined or established. Gaining further insight into the mechanism behind the elevated levels of sd LDL in patients with diabetes could be highly consequential for CVD prevention. Hence, we opted for a comprehensive MR approach to explore the potential mechanisms behind elevated sd LDL levels in patients with T2DM, considering aspects related to diabetes and glucose characteristics, hypertension, and lipid characteristics.

## Study design and methodology

### Ethics statement

This study, utilizing Mendelian randomization methodology, does not involve direct intervention with human participants. The analysis relies solely on naturally occurring genetic variations, and as such, it does not require specific ethical approval.

### MR study design

Extract summary data from the IEU OpenGWAS database (https://gwas.mrcieu.ac.uk/) for GWAS related to exposure and outcomes. Utilize these datasets for two-sample MR analysis to investigate potential mechanisms underlying the elevation of sd LDL in patients with diabetes.

### Assumptions for the MR study

UVMR relies on meeting three fundamental assumptions: (1) The instrumental variables must demonstrate a strong correlation with the exposure. Specifically, the genetic variants employed as instruments should reliably predict variations in the targeted exposure. (2) These instrumental variables must remain unassociated with potential confounding factors to ensure that the observed relationship between the instrumental variables and the outcome is not confounded by variables that could independently influence both the exposure and the outcome. (3) The instrumental variables are unable to impact the outcome except through the exposure. This implies that any observed effects on the outcome are solely mediated by variations in the exposure level determined by the instrumental variables. MVMR, on the other hand, can concurrently evaluate individual yet interrelated exposures by incorporating genetic variants of each risk factor into the same model [21–23]. MVMR has been utilized to investigate the direct influence of each risk factor, which is not mediated by other associated risk factors, on various health outcomes [24].

**Fig 1:**
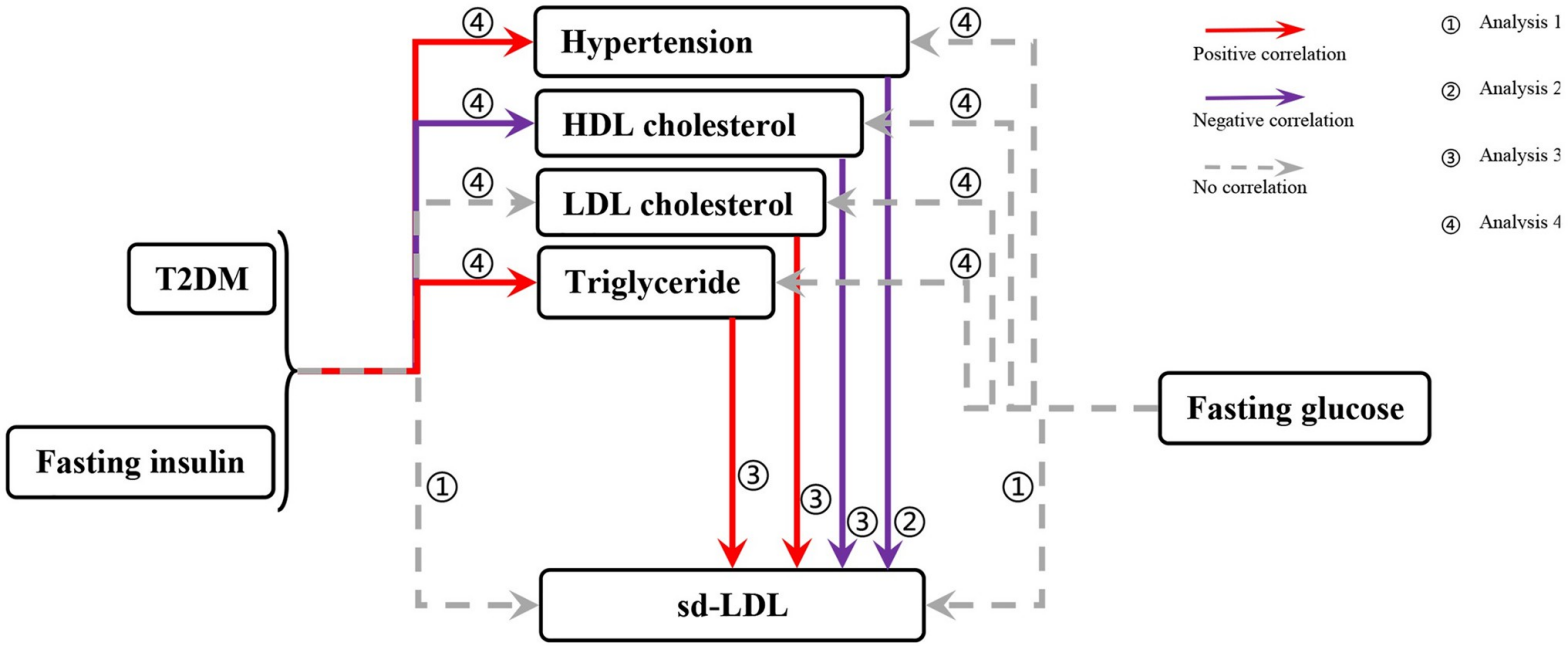
The specific analysis process. Initially, the first MR analysis was conducted with T2DM and glucose characteristics (fasting insulin and fasting plasma glucose) as the exposure variables and sd LDL as the outcome. This aimed to explore potential causal relationships, and the absence of a direct association ruled out the possibility of a direct mechanism of action. Subsequently, considering blood pressure and dyslipidemia as common complications of diabetes, these factors were employed as exposure variables in a subsequent MR Analysis to investigate indirect mechanisms. After establishing the corresponding causal relationship, the final MR Analysis was conducted again. This involved using T2DM and blood glucose characteristics (fasting insulin and fasting blood glucose) as exposure variables, and hypertension and blood lipid characteristics as outcomes. The results were then integrated with previous findings to ultimately elucidate the possible mechanism behind elevated sd LDL levels in patients with T2DM.

In this study, T2DM, blood glucose characteristics (fasting glucose and fasting insulin), hypertension, and blood lipid characteristics (HDL cholesterol, LDL cholesterol, and triglycerides) were utilized as exposures. The outcomes included small dense LDL particles and small dense LDL cholesterol. Subsequently, instrumental variables for the two-sample UVMR analysis were selected, and Cochran Q analysis was employed to assess heterogeneity. To ensure the robustness of the causal results, sensitivity analysis, including horizontal pleiotropy analysis and the "leave-one method," was conducted. MVMR analysis was executed to identify significant and potentially interrelated risk factors and ascertain independent risk factors. Another round of MVMR analysis involved T2DM and glucose characteristics as exposures, while hypertension and lipid characteristics were considered as outcomes. The cumulative results were then analyzed to elucidate the causal relationships among T2DM, blood glucose characteristics, hypertension, and hyperlipidemia. In the concluding phase, all the results were consolidated to analyze the potential mechanism of elevated sd LDL in patients with diabetes. The specific analysis process is detailed in Fig 1.

### Data sources

The study incorporated genome-wide association studies data accessed through https://gwas.mrcieu.ac.uk/. To ensure the robustness of the results from the two samples, precautions were taken to avoid using exposure and outcome data from the same database. Data with a large sample size and relatively recent data within the past five years were selected specifically. All data originated from European populations, and summarized information is provided in Table 1.

**Table 1:** Description of contributing studies.

Type	Phenotype	Year	Consortium	Sex	Population	SNP	Simple size	Access address	PMID
Type 2 Diabetes	Type 2 diabetes, strict(exclude DM1)	2021	-	Males and Females	Europeans	16,380,434	-	https://gwas.mrcieu.ac.uk/datasets/finn-b-E4_DM2_STRICT/	-
Blood sugar	Fasting glucose	2021	-	-	Europeans	31,008,728	200,622	https://gwas.mrcieu.ac.uk/datasets/ebi-a-GCST90002232/	34059833
	Fasting insulin	2021	-	-	Europeans	29,664,438	151,013	https://gwas.mrcieu.ac.uk/datasets/ebi-a-GCST90002238/	34059833
hypertension	Diagnoses—secondary ICD10: I10 Essential (primary) hypertension	2018	MRC-IEU	Males and Females	Europeans	9,851,867	463,010	https://gwas.mrcieu.ac.uk/datasets/ukb-b-12493/	-
Blood fat	HDL cholesterol	2020	UK Biobank	Males and Females	Europeans	12,321,875	403,943	https://gwas.mrcieu.ac.uk/datasets/ieu-b-109/	32203549
	LDL cholesterol	2020	UK Biobank	Males and Females	Europeans	12,321,875	440,546	https://gwas.mrcieu.ac.uk/datasets/ieu-b-110/	32203549
	Triglycerides	2020	UK Biobank	Males and Females	Europeans	12,321,875	441,016	https://gwas.mrcieu.ac.uk/datasets/ieu-b-111/	32203549
Small LDL	Concentration of small LDL particles	2020	-	Males and Females	Europeans	12,321,875	115,078	https://gwas.mrcieu.ac.uk/datasets/met-d-S_LDL_P/	-
	Cholesterol in small LDL	2020	-	Males and Females	Europeans	12,321,875	115,078	https://gwas.mrcieu.ac.uk/datasets/met-d-S_LDL_C/	-

### Choice of tool variables

SNPs that exhibited significant associations with exposure (p<5e-08, with a parameter r^2^ threshold of 0.001 and a kilobase pair threshold of 10,000) were meticulously selected for genome-wide reference. The LD_clumping function was employed to mitigate the impact of linkage disequilibrium. Subsequently, missing SNPs were excluded from the resultant database. The analysis was performed using the R software package TwoSampleMR 4.0. Effective SNPs displaying a significant correlation with exposure were identified as instrumental variables. To assess the potential for weak instrumental variable bias, the F statistic (F = beta^2^/se^2^) of each SNP was employed in two-sample univariate MR analysis [25]. SNPs demonstrating weak correlation (F<10) were systematically eliminated, leaving only those with a robust correlation for further analysis. Finally, data extraction from the outcome database was executed, and the collected information was collated and merged. In the MVMR analysis, the MVMR package was utilized to calculate the F statistic for each exposed population individually (F>10 indicates the absence of weak instrumental variables).

### Statistical analysis and data visualization

In the UVMR analysis, the impact of the exposure on the outcome was assessed using the Wald ratio for each SNP. The effect of each SNP is reported based on the normalized log-transformed level of exposure. The primary MR analysis utilized the IVW method. In this approach, the estimate of the SNP-to-outcome association is regressed on the SNP-to-exposure estimate. Subsequently, the causal effect estimates, equivalent to beta coefficients, were computed. Additionally, MR-Egger, simple mode, weighted mode, and weighted median methods served as supplementary approaches to IVW. Cochrane’s Q values were employed to assess heterogeneity. Horizontal pleiotropy occurs when the variant has an effect on disease outside of its effect on the exposure in MR. Violation of the ’no horizontal pleiotropy’ assumption can cause severe bias in MR [26]. The MR-Egger intercept was used for detecting horizontal pleiotropy, and if an outlier was identified, it was systematically removed, and the MR causal estimate was re-evaluated. In the MVMR analysis, the IVW method remained the primary approach, supplemented by MR-Egger, lasso, and median methods. The MRPRESSO method was employed to complement the MVMR results. All models adopted a random-effects model to minimize the occurrence of false-positive results.

For UVMR analysis, the R package "TwoSampleMR" played a central role. MVMR analysis was executed using R packages "TwoSampleMR," "MendelianRandomization," "MRPRESSO," and "MVMR."

## Results

### Instrumental variable

The UVMR analysis yielded the following results: T2DM was assessed with 58 instrumental variables, blood glucose characteristics with 66 for fast glucose and 38 for fasting insulin, hypertension with 71, HDL cholesterol with 362, LDL cholesterol with 177, and triglycerides with 313 instrumental variables. For the six groups in the subsequent MVMR analysis, the instrumental variables numbered 394, 394, 117, 117, 117, and 117, respectively. In the realm of MR, the strength of instrumental variables was evaluated using the F statistic, where a value exceeding 10 signifies a robust instrumental variable. The instrumental variables demonstrated substantial strength in our analysis, as evidenced by F statistics ranging from 22.44 to 5569.87 in the UVMR Analysis (S1 Table). Additionally, F statistics for SNPs used in MVMR varied from 15.85 to 51.47 (S8 and S11 Tables). Importantly, no evidence of weak instrumental variable bias was detected, affirming the reliability of these instrumental variables for estimating the causal effect of exposure on outcomes.

### T2DM or blood glucose characteristics unrelated to abnormal sd LDL levels

The beta and their respective 95% CI, calculated using IVW, are as follows: T2DM and sd LDL particles [-0.028, (-0.084;0.029), p = 0.338]; T2DM and sd LDL cholesterol [-0.040, (-0.102;0.022), p = 0.205]; Fasting glucose and sd LDL particles [0.009, (-0.119;0.137), p = 0.892]; Fasting glucose and sd LDL cholesterol [-0.006, (-0.136;0.124), p = 0.927]; Fasting insulin and sd LDL particles [0.180, (-0.089;0.449), p = 0.189]; Fasting insulin and sd LDL cholesterol [0.042, (-0.212;0.296), p = 0.747].

The research demonstrates that T2DM itself, as well as blood sugar levels and insulin resistance, do not directly influence the levels of sd LDL. Furthermore, it suggests the existence of other indirect mechanisms. Comprehensive details can be found in S2 Table.

### Lipid characteristics for the risk of sd LDL

The UVMR analysis indicated a negative correlation between HDL cholesterol and sd LDL levels [HDL cholesterol and sd LDL particles, sd LDL cholesterol: -0.173, (-0.239;-0.107), p = 3.14E-07; -0.130, (-0.198;-0.063), p = 0.0002]. Conversely, there was a positive correlation observed between LDL cholesterol and sd LDL [LDL cholesterol and sd LDL particles, sd LDL cholesterol: 0.881, (0.826;0.936), p = 1.97E-216; 0.897, (0.849;0.945), p = 4.09E-293]. Additionally, triglycerides exhibited a positive correlation with sd LDL levels [triglycerides and sd LDL particles, sd LDL cholesterol: 0.420, (0.373;0.466), p = 6.58E-71; 0.279, (0.232;0.326), p = 1.78E-31]. Detailed information can be found in S2 Table.

**Fig 2:**
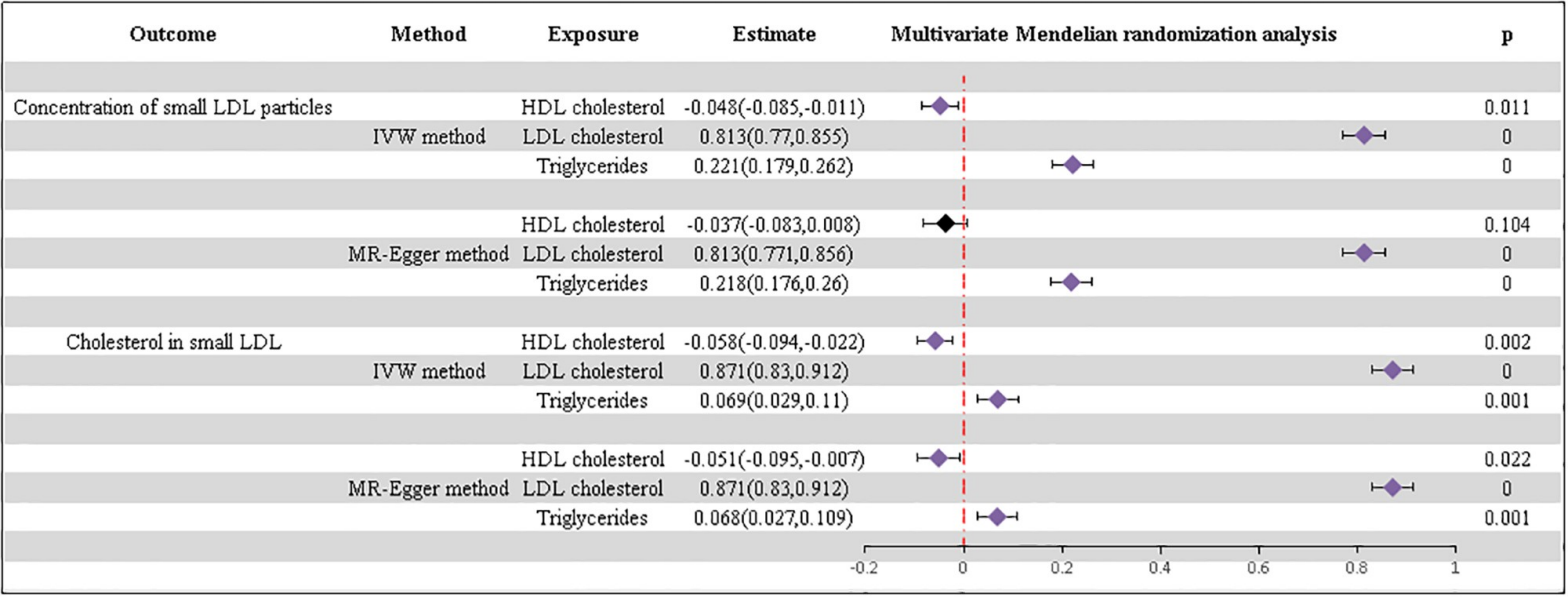
Results of the MVMR analysis. Exposure: lipid characteristics, outcomes: sd LDL particles and sd LDL cholesterol.

Based on the univariate analysis outcomes and the potential mutual interference between HDL cholesterol, LDL cholesterol, and triglycerides, a MVMR analysis was performed. This analysis aimed to investigate sd LDL particles and sd LDL cholesterol as outcomes, incorporating blood lipid characteristics as the exposure variables. Each of the parameters—HDL cholesterol, LDL cholesterol, and triglycerides—demonstrates an individual causal association with sd LDL particles, with estimated values obtained via the IVW method: [-0.048, (-0.085;-0.011), p = 0.011; 0.813, (0.770;0.855), p = 0.000; 0.221, (0.179;0.262), p = 0.000]. Similarly, these three factors also exhibit a causal relationship with sd LDL cholesterol, with estimated values obtained through the IVW method: [-0.058, (-0.094;-0.022), p = 0.002; 0.871, (0.830;0.912), p = 0.000; 0.069, (0.029,0.110), p = 0.001] (Fig 2). Additional information is provided in S6 Table.

The study employed MRPRSSO to complement and confirm the outcomes derived from the MVMR analysis. The MRPRSSO findings, available in S7 Table, indicated consistency with the previous results. Additionally, tests such as the Cochran Q test and MR-Egger regression conducted with IVW highlighted heterogeneity among SNPs. The Egger intercept did not demonstrate statistical significance, implying an absence of horizontal pleiotropy among the SNPs. Moreover, the F-statistic for the SNPs used in each exposure group exceeded 10, signifying a robust association of the SNPs with the respective exposure factors (S8 Table).

The ultimate conclusion aligns with the univariate analysis, affirming a significant causal relationship between HDL cholesterol, HDL cholesterol, triglycerides, and sd LDL. Specifically, HDL cholesterol demonstrates an inverse correlation with sd LDL levels, whereas LDL cholesterol and triglycerides are positively associated with sd LDL levels.

### Hypertension reduced the risk of increased sd LDL

**Fig 3:**
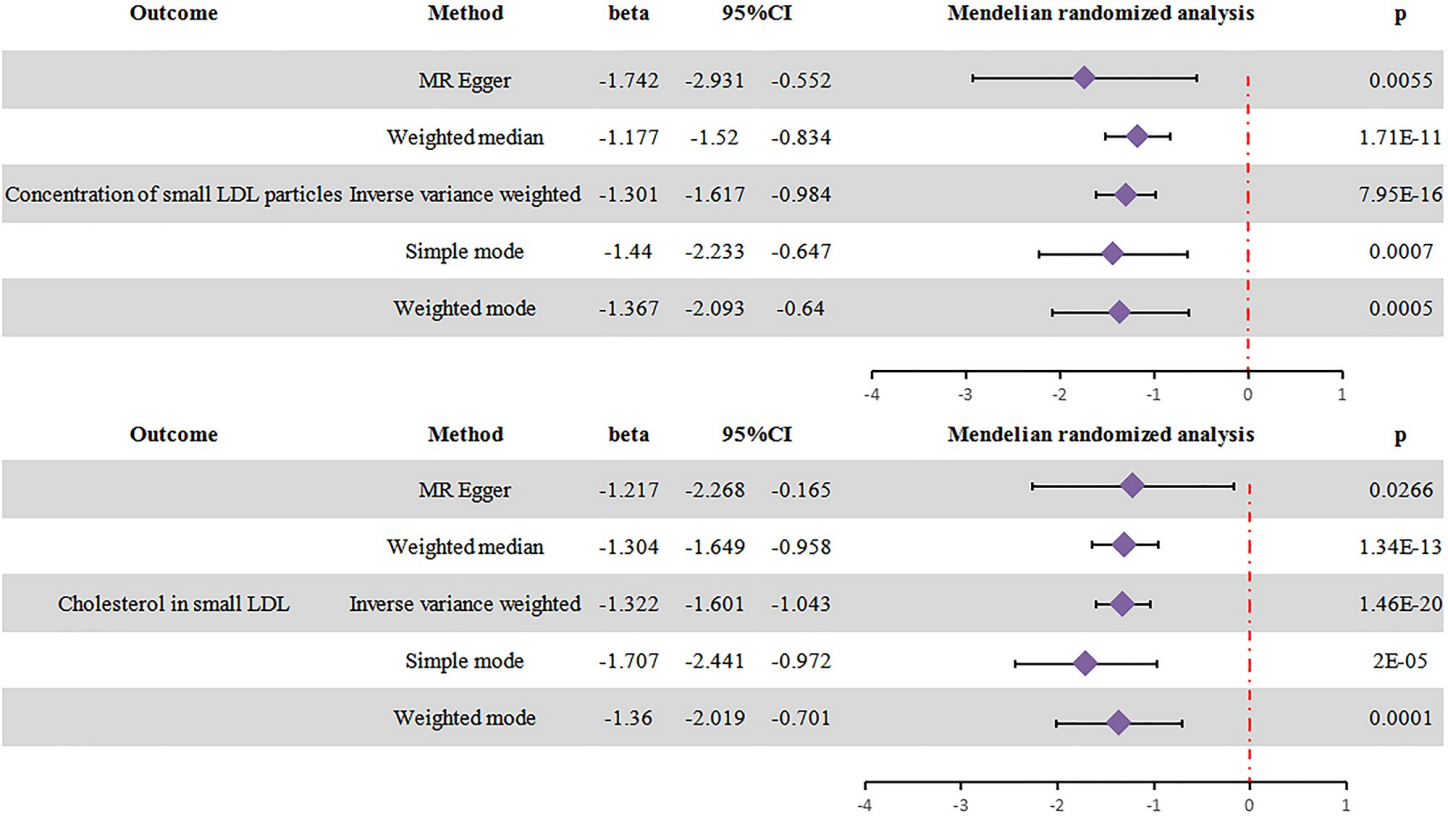
Results of the UVMR analysis. Exposure: hypertension, outcomes: sd LDL particles and sd LDL cholesterol.

In the UVMR with hypertension as exposure and sd LDL as outcome, the IVW analysis showed beta and 95%CI [Hypertension and sd LDL particles, sd LDL cholesterol: -1.301, (-1.617;-0.984), p = 7.59E-16; -1.322, (-1.601;-1.043), p = 1.46E-20 (Fig 3).

The outcomes, as depicted in Fig 3 are noteworthy, suggesting that hypertension reduces the risk of increased sd LDL.

### The risk of hypertension and lipid characteristics concerning T2DM and glycemic characteristics

**Fig 4:**
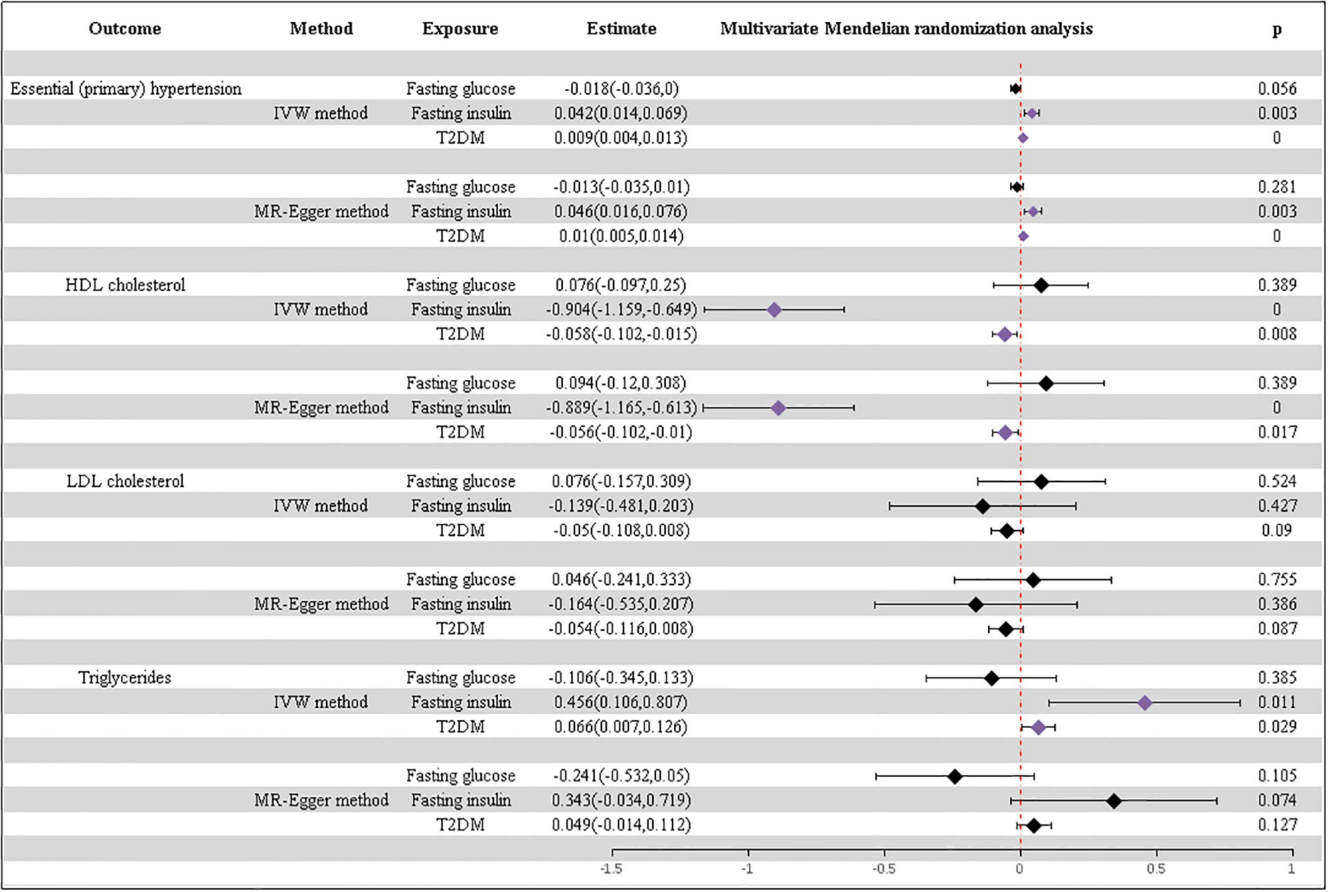
Results of the MVMR analysis. Exposures: T2DM and glycemic characteristics, outcomes: hypertension and lipid characteristics.

MVMR analysis was also conducted utilizing T2DM, fasting glucose, and fasting insulin levels as exposures, while hypertension, HDL cholesterol, LDL cholesterol, and triglycerides were considered as outcomes, respectively. The findings indicated that blood glucose levels did not demonstrate any significant association with hypertension, HDL cholesterol, LDL cholesterol, or triglyceride levels. T2DM and insulin resistance were observed to correlate with increased blood pressure, reduced levels of HDL cholesterol, and elevated levels of triglycerides, yet no significant association was noted with LDL cholesterol levels (Fig 4). The conclusion was further supported by the results of the MRPRSSO analysis. S9–S11 Tables offer additional information regarding this analysis.

### Additional results from the UVMR analysis

**Fig 5:**
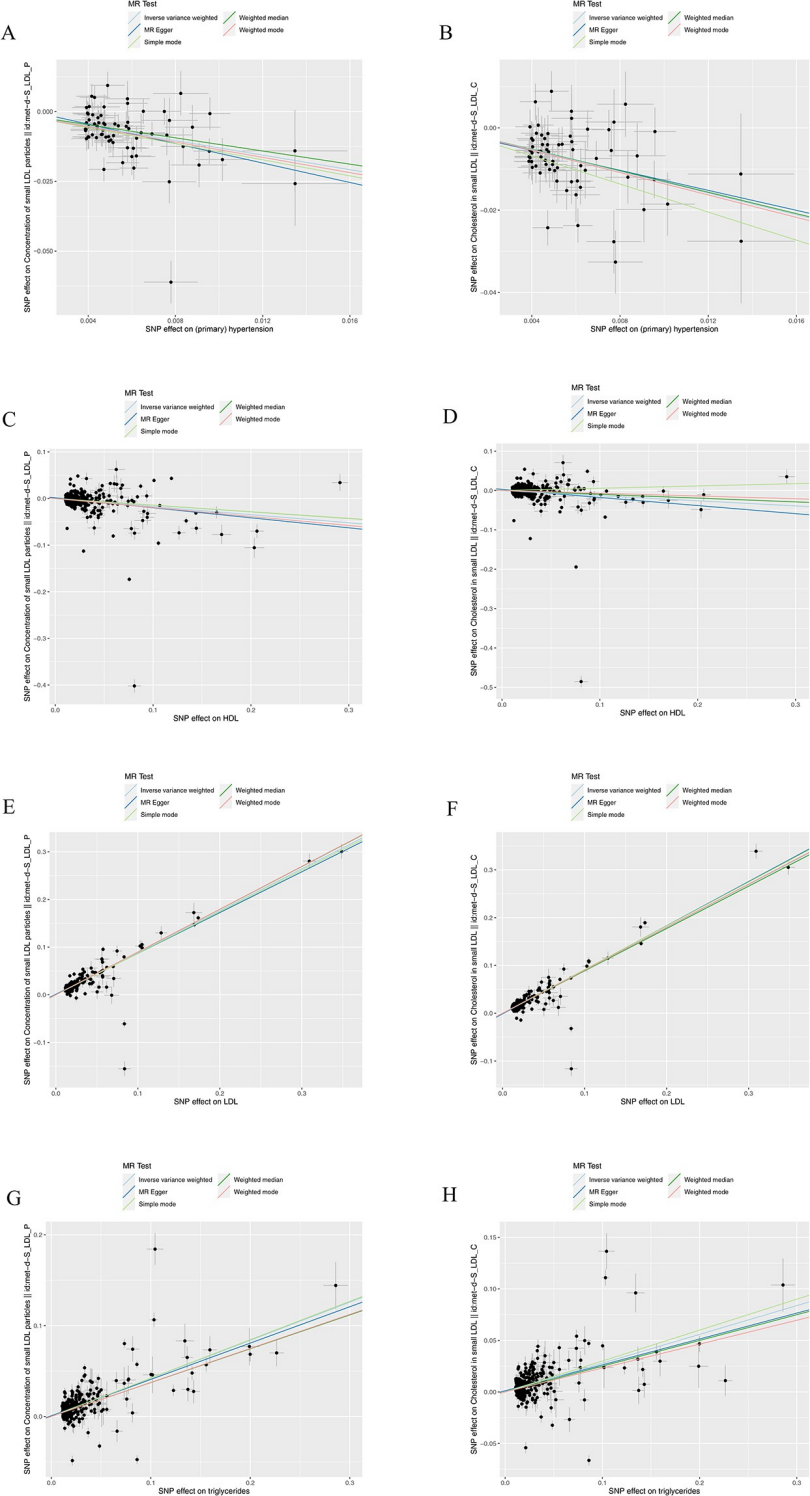
Partial scattered point plots obtained by UVMR. Horizontal ordinate: SNP effect on “exposure”; Vertical coordinates: SNP effect on “outcome.” (A) Exposure: hypertension, outcome: sd LDL particles; (B)Exposure: hypertension, outcome: sd LDL cholesterol; (C) Exposure: HDL cholesterol, sd LDL particles; (D) Exposure: HDL cholesterol, outcome: sd LDL cholesterol; (E) Exposure: LDL cholesterol, outcome: sd LDL particles; (F) Exposure: LDL cholesterol, outcome: sd LDL cholesterol; (G) Exposure: triglycerides, outcome: sd LDL particles; (H) Exposure: triglycerides, outcome: sd LDL cholesterol.

**Fig 6:**
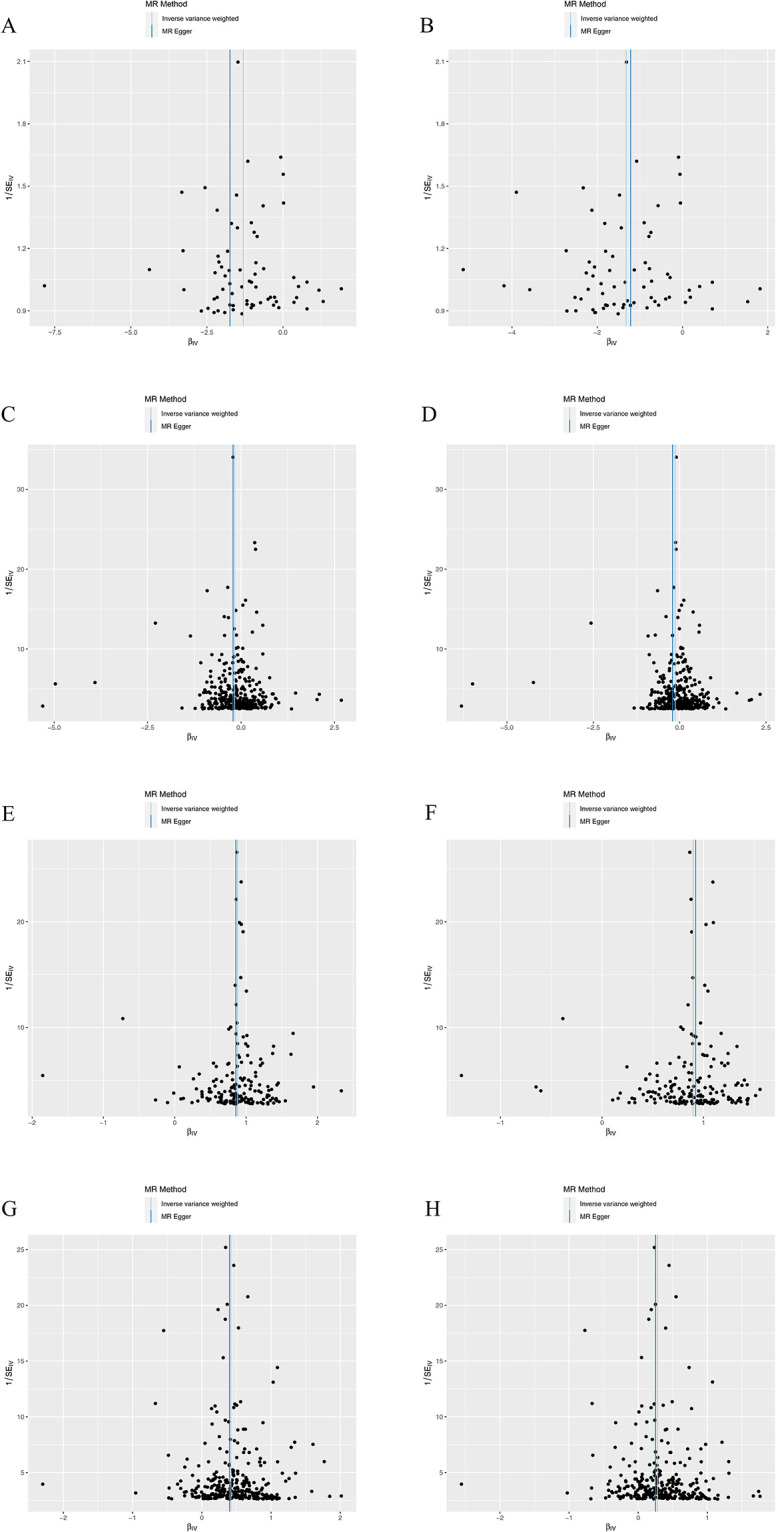
Partial funnel plots obtained by UVMR. Exposure: hypertension, outcome: sd LDL particles; (B)Exposure: hypertension, outcome: sd LDL cholesterol; (C) Exposure: HDL cholesterol, sd LDL particles; (D) Exposure: HDL cholesterol, outcome: sd LDL cholesterol; (E) Exposure: LDL cholesterol, outcome: sd LDL particles; (F) Exposure: LDL cholesterol, outcome: sd LDL cholesterol; (G) Exposure: triglycerides, outcome: sd LDL particles; (H) Exposure: triglycerides, outcome: sd LDL cholesterol.

The outcomes of the UVMR analysis of a single SNP are showcased in S3 and S4 Tables. Subsequent to this, an analysis of heterogeneity was carried out using MR-Egger regression and an intercept term analysis, demonstrating noticeable heterogeneity but no substantial pleiotropy, as indicated in S5 Table. Figs 5 and 6 show partial scatter point plots and funnel plots, respectively, with the remainder seen in S1-S12 Figs in S1 File. Additionally, a sensitivity analysis, employing the " leave–one–out sensitivity analysis" was conducted to sequentially eliminate individual SNPs from the data for further analysis (S13-S26 Figs in S1 File). Ultimately, no SNPs significantly impacting the results were identified, thereby affirming the credibility of the findings.

## Discussion

Diabetes is emerging as a global health challenge, as indicated by studies projecting a potential increase in the diabetic population to 700 million by the year 2045 [2]. Remarkably, patients with diabetes face a significant burden of morbidity, disability, and mortality primarily attributed to CVD [6]. Research indicates a pivotal role for sd LDL in the additional assessment of CVD risk in patients with diabetes [9, 27]. Consequently, delving deeper into the mechanisms governing the elevated levels of sd LDL in patients with T2DM holds significant promise. Such exploration bears potential significance in the pursuit of effective strategies for controlling and preventing CVD and, in turn, mitigating the risk of mortality in patients with T2DM. However, the intricate interconnection of sd LDL with various risk factors, such as triglycerides, HDL, diabetes, and insulin resistance [9], has contributed to the ongoing lack of clarity regarding the specific underlying mechanism.

Previous studies have documented elevated sd LDL levels in patients with T2DM [15, 18, 19, 27], accompanied by an association with insulin resistance [20, 28]. However, the MR analysis revealed no direct correlation between T2DM or fasting glucose and sd LDL. Moreover, insulin levels were not found to directly contribute to the elevation of sd LDL levels. Although recent evidence suggests that the generation of sd LDL particles within the insulin-resistant environment constitutes a significant metabolic transition [29], a specific study also revealed that neither "insulin resistance" nor "insulin level" independently exerted an effect on LDL particle size [30]. This suggests that insulin resistance may have a significant but indirect influence, and a similar pattern may apply to T2DM and blood glucose levels.

Hyperlipidemia stands as a significant complication in patients with T2DM. MR analysis revealed an inverse association between T2DM or insulin resistance with HDL cholesterol and a positive association with triglycerides, but no significant correlation with LDL cholesterol. Additionally, there was no observed correlation between blood glucose levels and lipid profiles. These findings suggest that T2DM or insulin resistance, rather than blood glucose levels, influences lipid metabolism by affecting HDL cholesterol and triglycerides, without significant impact on LDL cholesterol. Interestingly, multiple studies have identified a pattern characterized by low levels of HDL cholesterol and elevated total triglycerides in populations exhibiting high levels of sd LDL [18, 31], and some have proposed a potential negative correlation between LDL particle size and triglyceride levels, along with a positive correlation with HDL cholesterol [32]. This MR analysis also revealed a negative correlation between sd LDL levels and HDL cholesterol, as well as a positive correlation between sd LDL levels and LDL cholesterol and triglycerides. This not only substantiates the hypotheses from prior studies regarding lipid levels and LDL particle size, but also establishes a connection between the mechanism of elevated sd LDL levels in patients with diabetes and blood lipids. Specifically, T2DM or insulin resistance appears to have no direct impact on sd LDL levels but indirectly leads to their increase by decreasing HDL cholesterol levels and increasing triglyceride levels. Blood glucose levels and LDL cholesterol are not implicated in this mechanism.

Hypertension emerges as another prevalent complication associated with diabetes. Research indicates that patients with diabetes face a doubled likelihood of developing high blood pressure [33]. The outcomes of MR analysis demonstrated a positive correlation between T2DM or insulin resistance and the risk of hypertension, while no significant correlation was observed between blood glucose levels and hypertension. Intriguingly, MR analysis also revealed an inverse relationship between hypertension and sd LDL levels, suggesting that a certain level of hypertension may contribute to the reduction of sd LDL levels. A particular study noted that individuals with malignant hypertension, the most severe form of hypertension, exhibited lower levels of sd LDL compared to those with non-malignant hypertension, although their sd LDL levels remained higher than those of normotensive controls [34]. When considered alongside the MR analysis results indicating a positive correlation between T2DM or insulin resistance and hypertension, as well as a negative correlation between hypertension and sd LDL levels, we postulate that this phenomenon may be attributed to the indirect benefit of hypertension, induced by T2DM or insulin resistance, in mitigating the increase of sd LDL levels. Moreover, the observation that sd LDL levels persisted at higher levels in the malignant hypertension group than in the normal group may be attributed to a combination of various factors. After all, patients with malignant hypertension exhibited significantly lower levels of HDL cholesterol, coupled with notably higher serum triglyceride concentrations [34]. Certainly, the clinical accuracy of this conclusion and the underlying mechanisms warrant confirmation through extensive studies.

In summary, this MR analysis revealed that T2DM, insulin resistance, and elevated blood glucose levels did not directly contribute to the elevation of sd LDL levels, indicating no direct correlation among them. However, the decrease in HDL cholesterol and the increase in triglycerides induced by T2DM or insulin resistance were identified as direct factors leading to elevated sd LDL levels. Notably, LDL cholesterol did not play a role in this process despite showing a positive correlation with sd LDL. Conversely, hypertension induced by T2DM or insulin resistance exhibited a negative correlation with sd LDL levels, suggesting that a certain level of hypertension in patients with diabetes might be associated with a reduction in sd LDL levels. Blood glucose was not implicated in any of these processes. Nonetheless, further extensive research is essential to validate these conclusions and elucidate the underlying mechanisms.

It is hoped that these findings might have a certain impact on the approach to treating patients with diabetes and the potential management of blood glucose, blood pressure, and lipid levels in clinical practice. Through an examination of the effect of T2DM or insulin resistance on sd LDL within the HDL cholesterol and TG pathways, we aim to provide insights that could potentially contribute to the development of more targeted treatments for cardiovascular complications in patients with diabetes. Additionally, the exploration of the potential positive effects of elevated blood pressure on sd LDL reversal in our study may offer some new considerations for blood pressure management in patients with diabetes in clinical practice.

## Conclusions

The MR Analysis conducted in this study investigated potential mechanisms responsible for the elevated levels of sd LDL in patients with T2DM. A comprehensive understanding of it may hold valuable significance for managing blood glucose, blood pressure and lipid levels, controlling sd LDL levels, and consequently lowering the incidence and mortality of CVD in patients with diabetes.

Certainly, this study still has some limitations. The primary composition of the study population is of European descent, which may restrict the generalizability of the results to other ethnic groups. Potential pleiotropy remains a seemingly reasonable limitation. Additionally, inherent heterogeneity in data arises from differences in exposure and outcome databases, variations in experimental populations, and diverse experimental methods, although MR analysis results maintain robustness and reliability. Due to screening conditions and database limitations, certain data may not reflect the latest information. These limitations underscore the necessity for a cautious interpretation of results and highlight avenues for future research to enhance the comprehensiveness and applicability of the study.

## Supporting information

S1 Table Strength of instrumental variables (value of F). (PDF)

S2 Table Results of UVMR analysis. (PDF)

S3 Table Mendelian randomization analysis of individual SNPS (with concentration of small LDL particles as the outcome). (PDF)

S4 Table Mendelian randomization analysis of individual SNPS (with cholesterol in small LDL as the outcome). (PDF)

S5 Table Heterogeneity and pleiotropic analysis. (PDF)

S6 Table Results of MVMR analysis (lipid profile as exposure, sd-LDL level as outcome). (PDF)

S7 Table MR-PRESSO validation of MVMR analysis (lipid profile as exposure, sd-LDL level as outcome). (PDF)

S8 Table Information about the MVMR (lipid profile as exposure, sd-LDL level as outcome). (PDF)

S9 Table Results of MVMR analysis (T2DM and glucose characteristics as exposures and hypertension and lipid characteristics as outcomes). (PDF)

S10 Table MR-PRESSO validated the results of MVMR analysis (T2DM and glucose characteristics as exposure, hypertension and lipid characteristics as outcome). (PDF)

S11 Table Information about the MVMR (T2DM and glucose characteristics as exposure, hypertension and lipid characteristics as outcome). (PDF)

S1 File (PDF)

## Abbreviations

95% CI: 95% confidence intervals
CHD: Coronary heart disease
CVD: Cardiovascular disease
GWAS: genome-wide association studies
HDL: High density lipoprotein
IVW: Inverse variance weighting
lb LDL: large buoyant low density lipoprotein
LDL: Low density lipoprotein
MR: Mendelian randomization
MVMR: Multivariate Mendelian randomization
sd LDL: Small dense low density lipoprotein
SNP: Single nucleotide polymorphism
T2DM: Type 2 diabetes mellitus
TG: Triglycerides
UVMR: Univariate Mendelian randomization
