# Supplementary material for: Small dense low density lipoprotein predominance in patients with type 2 diabetes mellitus using Mendelian randomization
Source: PLoS One. 2024 Feb 8;19(2):e0298070. doi: 10.1371/journal.pone.0298070 (PMC10852223; doi:10.1371/journal.pone.0298070)
Supplement: S1 Table — (PDF) [file pone.0298070.s001.pdf]

Supplementary Table 1

Strength of instrumental variables (Value of F)

SNP	exposure	F
rs10050393	Fasting insulin || id:ebi-a-GCST90002238	22.43767313
rs17270243	Fasting glucose || id:ebi-a-GCST90002232	24.5260771
rs13258890	Fasting insulin || id:ebi-a-GCST90002238	26.2144
rs11727676	Fasting insulin || id:ebi-a-GCST90002238	27.09335963
rs17331151	Fasting insulin || id:ebi-a-GCST90002238	27.30905307
rs2845885	Fasting insulin || id:ebi-a-GCST90002238	27.36094675
rs5017305	Fasting insulin || id:ebi-a-GCST90002238	27.7647929
rs62271373	Fasting insulin || id:ebi-a-GCST90002238	28.44444444
rs2657879	Fasting glucose || id:ebi-a-GCST90002232	29.25826446
rs39713	Fasting glucose || id:ebi-a-GCST90002232	29.72008325
rs1870735	Diagnoses - secondary ICD10: I10 Essential (primary) hypertension || id:ukb-b-12493	29.74799525
rs745805	Type 2 diabetes, strict (exclude DM1) || id:finn-b-E4_DM2_STRICT	29.81734765
rs2290866	HDL cholesterol || id:ieu-b-109	29.85906772
rs76962725	HDL cholesterol || id:ieu-b-109	29.8597891
rs407133	HDL cholesterol || id:ieu-b-109	29.87351432
rs6805924	triglycerides || id:ieu-b-111	29.90544578
rs17437560	Fasting glucose || id:ebi-a-GCST90002232	29.90722656
rs880674	HDL cholesterol || id:ieu-b-109	29.95330619
rs11704977	HDL cholesterol || id:ieu-b-109	29.98749843
rs113851927	HDL cholesterol || id:ieu-b-109	30.0178498
rs62331150	HDL cholesterol || id:ieu-b-109	30.02132144
rs117657619	Type 2 diabetes, strict (exclude DM1) || id:finn-b-E4_DM2_STRICT	30.03719728
rs112350227	HDL cholesterol || id:ieu-b-109	30.08554735
rs7135509	triglycerides || id:ieu-b-111	30.09090153
rs1798085	Type 2 diabetes, strict (exclude DM1) || id:finn-b-E4_DM2_STRICT	30.09881323
rs147772065	HDL cholesterol || id:ieu-b-109	30.10475417
rs146433259	LDL cholesterol || id:ieu-b-110	30.10661559
rs143076454	triglycerides || id:ieu-b-111	30.18254454
rs278981	triglycerides || id:ieu-b-111	30.19676619
rs1760940	HDL cholesterol || id:ieu-b-109	30.21401757
rs73455693	HDL cholesterol || id:ieu-b-109	30.21771132
rs6432622	triglycerides || id:ieu-b-111	30.23008732
rs2155220	HDL cholesterol || id:ieu-b-109	30.23893288

rs12967878	Type 2 diabetes, strict (exclude DM1) || id:finn-b-E4_DM2_STRICT	30.25
rs4760278	Fasting glucose || id:ebi-a-GCST90002232	30.25
rs76468627	LDL cholesterol || id:ieu-b-110	30.28808747
rs7224685	Type 2 diabetes, strict (exclude DM1) || id:finn-b-E4_DM2_STRICT	30.29702681
rs57760538	HDL cholesterol || id:ieu-b-109	30.32882292
rs7297416	Diagnoses - secondary ICD10: I10 Essential (primary) hypertension || id:ukb-b-12493	30.34071854
rs9889402	triglycerides || id:ieu-b-111	30.35751666
rs77767539	HDL cholesterol || id:ieu-b-109	30.36781734
rs3758413	triglycerides || id:ieu-b-111	30.39199568
rs12781812	HDL cholesterol || id:ieu-b-109	30.4451033
rs134551	triglycerides || id:ieu-b-111	30.47182662
rs12762222	Diagnoses - secondary ICD10: I10 Essential (primary) hypertension || id:ukb-b-12493	30.50044869
rs7903146	Fasting insulin || id:ebi-a-GCST90002238	30.51247166
rs972283	Fasting insulin || id:ebi-a-GCST90002238	30.5401662
rs71603401	HDL cholesterol || id:ieu-b-109	30.5581365
rs12948505	triglycerides || id:ieu-b-111	30.56578239
rs454968	HDL cholesterol || id:ieu-b-109	30.59789305
rs17558745	Diagnoses - secondary ICD10: I10 Essential (primary) hypertension || id:ukb-b-12493	30.61245455
rs111363680	HDL cholesterol || id:ieu-b-109	30.63876076
rs34672664	triglycerides || id:ieu-b-111	30.67007984
rs12986742	HDL cholesterol || id:ieu-b-109	30.69941322
rs79828839	LDL cholesterol || id:ieu-b-110	30.74124019
rs6572807	triglycerides || id:ieu-b-111	30.77292096
rs9471968	LDL cholesterol || id:ieu-b-110	30.78210296
rs62473520	triglycerides || id:ieu-b-111	30.81345444
rs2604568	triglycerides || id:ieu-b-111	30.84027814
rs2070341	triglycerides || id:ieu-b-111	30.86680737
rs28439112	triglycerides || id:ieu-b-111	30.88358308
rs34302257	triglycerides || id:ieu-b-111	30.90187604
rs2391825	LDL cholesterol || id:ieu-b-110	30.96225519
rs34207171	LDL cholesterol || id:ieu-b-110	30.97997646
rs34265667	LDL cholesterol || id:ieu-b-110	30.99527031
rs75942983	triglycerides || id:ieu-b-111	31.05922271
rs2131311	triglycerides || id:ieu-b-111	31.07872417
rs12705595	HDL cholesterol || id:ieu-b-109	31.0797282
rs200610097	triglycerides || id:ieu-b-111	31.1015304
rs6074012	LDL cholesterol || id:ieu-b-110	31.10233789

rs61884005	HDL cholesterol || id:ieu-b-109	31.10246632
rs7108486	LDL cholesterol || id:ieu-b-110	31.11698788
rs5832650	triglycerides || id:ieu-b-111	31.12418869
rs201639483	HDL cholesterol || id:ieu-b-109	31.13709804
rs394872	triglycerides || id:ieu-b-111	31.16313491
rs1955512	HDL cholesterol || id:ieu-b-109	31.16944979
rs3887925	Type 2 diabetes, strict (exclude DM1) || id:finn-b-E4_DM2_STRICT	31.21631176
rs7528118	Diagnoses - secondary ICD10: I10 Essential (primary) hypertension || id:ukb-b-12493	31.25823656
rs880315	triglycerides || id:ieu-b-111	31.2758186
rs10031010	HDL cholesterol || id:ieu-b-109	31.35485887
rs55670730	Diagnoses - secondary ICD10: I10 Essential (primary) hypertension || id:ukb-b-12493	31.35828649
rs28553330	Type 2 diabetes, strict (exclude DM1) || id:finn-b-E4_DM2_STRICT	31.38935184
rs2187114	triglycerides || id:ieu-b-111	31.40781844
rs4899251	HDL cholesterol || id:ieu-b-109	31.41781352
rs7507893	Type 2 diabetes, strict (exclude DM1) || id:finn-b-E4_DM2_STRICT	31.42168168
rs56273825	Diagnoses - secondary ICD10: I10 Essential (primary) hypertension || id:ukb-b-12493	31.42504007
rs3746915	HDL cholesterol || id:ieu-b-109	31.42819559
rs117316645	triglycerides || id:ieu-b-111	31.44296284
rs182788819	Type 2 diabetes, strict (exclude DM1) || id:finn-b-E4_DM2_STRICT	31.45573131
rs11637681	triglycerides || id:ieu-b-111	31.50272227
rs6028716	triglycerides || id:ieu-b-111	31.54073359
rs73238173	triglycerides || id:ieu-b-111	31.54941669
rs35786744	triglycerides || id:ieu-b-111	31.55827368
rs55646464	triglycerides || id:ieu-b-111	31.55851274
rs77320712	HDL cholesterol || id:ieu-b-109	31.6186581
rs73013411	Fasting insulin || id:ebi-a-GCST90002238	31.640625
rs346078	Diagnoses - secondary ICD10: I10 Essential (primary) hypertension || id:ukb-b-12493	31.6938344
rs7861679	triglycerides || id:ieu-b-111	31.69680493
rs62137406	Type 2 diabetes, strict (exclude DM1) || id:finn-b-E4_DM2_STRICT	31.70277899
rs28406917	LDL cholesterol || id:ieu-b-110	31.70766974
rs12449219	Type 2 diabetes, strict (exclude DM1) || id:finn-b-E4_DM2_STRICT	31.73768207
rs61748951	HDL cholesterol || id:ieu-b-109	31.76540343
rs117291242	triglycerides || id:ieu-b-111	31.79510192
rs10277582	triglycerides || id:ieu-b-111	31.80723429
rs499293	triglycerides || id:ieu-b-111	31.85890288
rs11185542	triglycerides || id:ieu-b-111	31.88257218
rs62089932	Diagnoses - secondary ICD10: I10 Essential (primary) hypertension || id:ukb-b-12493	31.89309717

rs12740811	HDL cholesterol || id:ieu-b-109	31.95038638
rs2137557	triglycerides || id:ieu-b-111	31.97810255
rs990619	LDL cholesterol || id:ieu-b-110	32.0300902
rs28814720	LDL cholesterol || id:ieu-b-110	32.04385448
rs62135012	triglycerides || id:ieu-b-111	32.05362555
rs4471666	triglycerides || id:ieu-b-111	32.05796602
rs680321	HDL cholesterol || id:ieu-b-109	32.05838604
rs2304969	triglycerides || id:ieu-b-111	32.09653238
rs194518	Fasting glucose || id:ebi-a-GCST90002232	32.11111111
rs7218647	HDL cholesterol || id:ieu-b-109	32.14705255
rs201441	HDL cholesterol || id:ieu-b-109	32.15057681
rs4291	Diagnoses - secondary ICD10: I10 Essential (primary) hypertension || id:ukb-b-12493	32.20751272
rs7077812	triglycerides || id:ieu-b-111	32.23382717
rs2750411	HDL cholesterol || id:ieu-b-109	32.24343276
rs1383732	HDL cholesterol || id:ieu-b-109	32.25032218
rs7826177	HDL cholesterol || id:ieu-b-109	32.25215186
rs12454712	Fasting insulin || id:ebi-a-GCST90002238	32.2624
rs35566853	HDL cholesterol || id:ieu-b-109	32.27844353
rs13121616	LDL cholesterol || id:ieu-b-110	32.29371435
rs34073570	HDL cholesterol || id:ieu-b-109	32.30423
rs869412	LDL cholesterol || id:ieu-b-110	32.3811433
rs1918898	Diagnoses - secondary ICD10: I10 Essential (primary) hypertension || id:ukb-b-12493	32.38241922
rs1411432	HDL cholesterol || id:ieu-b-109	32.38918766
rs42125	HDL cholesterol || id:ieu-b-109	32.39042873
rs6762415	HDL cholesterol || id:ieu-b-109	32.42921067
rs2983896	triglycerides || id:ieu-b-111	32.42935592
rs564832	HDL cholesterol || id:ieu-b-109	32.47371672
rs72964564	HDL cholesterol || id:ieu-b-109	32.49368989
rs11009262	HDL cholesterol || id:ieu-b-109	32.49539039
rs75479205	HDL cholesterol || id:ieu-b-109	32.53864192
rs7316878	HDL cholesterol || id:ieu-b-109	32.54596734
rs2362541	HDL cholesterol || id:ieu-b-109	32.598355
rs78545596	triglycerides || id:ieu-b-111	32.61476046
rs202079372	HDL cholesterol || id:ieu-b-109	32.65527424
rs429358	Type 2 diabetes, strict (exclude DM1) || id:finn-b-E4_DM2_STRICT	32.77965681
rs1395221	HDL cholesterol || id:ieu-b-109	32.8019887
rs7241918	LDL cholesterol || id:ieu-b-110	32.80412615

rs35184780	Diagnoses - secondary ICD10: I10 Essential (primary) hypertension || id:ukb-b-12493	32.80805258
rs34389751	HDL cholesterol || id:ieu-b-109	32.81732405
rs7847285	triglycerides || id:ieu-b-111	32.83250523
rs5215	Type 2 diabetes, strict (exclude DM1) || id:finn-b-E4_DM2_STRICT	32.84987997
rs1133400	triglycerides || id:ieu-b-111	32.87119401
rs6059958	HDL cholesterol || id:ieu-b-109	32.89138047
rs12445804	LDL cholesterol || id:ieu-b-110	32.94459621
rs12926854	HDL cholesterol || id:ieu-b-109	32.94720503
rs117762989	HDL cholesterol || id:ieu-b-109	32.97475769
rs61754230	LDL cholesterol || id:ieu-b-110	33.0261596
rs2108349	Fasting insulin || id:ebi-a-GCST90002238	33.0625
rs67016280	HDL cholesterol || id:ieu-b-109	33.07291362
rs6560499	LDL cholesterol || id:ieu-b-110	33.07297439
rs56236159	LDL cholesterol || id:ieu-b-110	33.08157426
rs2645979	HDL cholesterol || id:ieu-b-109	33.11031669
rs12932686	Diagnoses - secondary ICD10: I10 Essential (primary) hypertension || id:ukb-b-12493	33.12414037
rs325485	triglycerides || id:ieu-b-111	33.12500562
rs2728624	Diagnoses - secondary ICD10: I10 Essential (primary) hypertension || id:ukb-b-12493	33.1589353
rs55637835	LDL cholesterol || id:ieu-b-110	33.18612613
rs10743152	Type 2 diabetes, strict (exclude DM1) || id:finn-b-E4_DM2_STRICT	33.21398892
rs56017932	HDL cholesterol || id:ieu-b-109	33.22751977
rs12898997	Fasting glucose || id:ebi-a-GCST90002232	33.23183391
rs162395	Diagnoses - secondary ICD10: I10 Essential (primary) hypertension || id:ukb-b-12493	33.23984724
rs4128205	triglycerides || id:ieu-b-111	33.24412257
rs12669911	triglycerides || id:ieu-b-111	33.30262857
rs7685862	Diagnoses - secondary ICD10: I10 Essential (primary) hypertension || id:ukb-b-12493	33.32354491
rs6866614	Diagnoses - secondary ICD10: I10 Essential (primary) hypertension || id:ukb-b-12493	33.32701645
rs141440048	HDL cholesterol || id:ieu-b-109	33.32747525
rs58925536	Fasting glucose || id:ebi-a-GCST90002232	33.33428266
rs112381903	triglycerides || id:ieu-b-111	33.39386968
rs2339234	HDL cholesterol || id:ieu-b-109	33.45987864
rs9373056	triglycerides || id:ieu-b-111	33.47238381
rs75268115	triglycerides || id:ieu-b-111	33.57735976
rs55807798	triglycerides || id:ieu-b-111	33.59990046
rs4148826	LDL cholesterol || id:ieu-b-110	33.62534878
rs4855582	HDL cholesterol || id:ieu-b-109	33.68703898
rs117431393	triglycerides || id:ieu-b-111	33.6890375

rs7244	triglycerides || id:ieu-b-111	33.69734031
rs498475	Type 2 diabetes, strict (exclude DM1) || id:finn-b-E4_DM2_STRICT	33.70162111
rs11187019	triglycerides || id:ieu-b-111	33.7096316
rs1340819	triglycerides || id:ieu-b-111	33.72934966
rs7281183	HDL cholesterol || id:ieu-b-109	33.74681209
rs4691379	HDL cholesterol || id:ieu-b-109	33.75814394
rs9465693	HDL cholesterol || id:ieu-b-109	33.7682601
rs71538127	triglycerides || id:ieu-b-111	33.76904891
rs2256814	LDL cholesterol || id:ieu-b-110	33.79972715
rs1250258	LDL cholesterol || id:ieu-b-110	33.84501181
rs9505086	Type 2 diabetes, strict (exclude DM1) || id:finn-b-E4_DM2_STRICT	33.85123967
rs2303700	Type 2 diabetes, strict (exclude DM1) || id:finn-b-E4_DM2_STRICT	33.86035969
rs6961048	Diagnoses - secondary ICD10: I10 Essential (primary) hypertension || id:ukb-b-12493	33.88866893
rs1349852	HDL cholesterol || id:ieu-b-109	33.90515212
rs1862205	HDL cholesterol || id:ieu-b-109	33.90609701
rs1009590	triglycerides || id:ieu-b-111	33.92511445
rs549058	HDL cholesterol || id:ieu-b-109	33.93786265
rs61003864	LDL cholesterol || id:ieu-b-110	33.96532058
rs438568	LDL cholesterol || id:ieu-b-110	34.00152204
rs2407278	triglycerides || id:ieu-b-111	34.02211469
rs75246752	HDL cholesterol || id:ieu-b-109	34.05496442
rs7308584	triglycerides || id:ieu-b-111	34.08558821
rs6766859	Diagnoses - secondary ICD10: I10 Essential (primary) hypertension || id:ukb-b-12493	34.10532048
rs320369	triglycerides || id:ieu-b-111	34.11641822
rs7251640	HDL cholesterol || id:ieu-b-109	34.15390121
rs111338114	LDL cholesterol || id:ieu-b-110	34.16487667
rs8042127	Diagnoses - secondary ICD10: I10 Essential (primary) hypertension || id:ukb-b-12493	34.25563797
rs113966472	HDL cholesterol || id:ieu-b-109	34.25910708
rs2003476	Diagnoses - secondary ICD10: I10 Essential (primary) hypertension || id:ukb-b-12493	34.30432882
rs72603744	triglycerides || id:ieu-b-111	34.34682707
rs10811662	triglycerides || id:ieu-b-111	34.35419639
rs6017317	Type 2 diabetes, strict (exclude DM1) || id:finn-b-E4_DM2_STRICT	34.37002506
rs115912456	HDL cholesterol || id:ieu-b-109	34.39830829
rs7714361	triglycerides || id:ieu-b-111	34.43749632
rs1077394	Diagnoses - secondary ICD10: I10 Essential (primary) hypertension || id:ukb-b-12493	34.44313295
rs11708067	Fasting insulin || id:ebi-a-GCST90002238	34.45179584
rs9584870	triglycerides || id:ieu-b-111	34.49636569

rs7305678	HDL cholesterol || id:ieu-b-109	34.52935494
rs150861794	HDL cholesterol || id:ieu-b-109	34.61160449
rs35882350	LDL cholesterol || id:ieu-b-110	34.61542755
rs11263763	Type 2 diabetes, strict (exclude DM1) || id:finn-b-E4_DM2_STRICT	34.63270421
rs7095788	Fasting glucose || id:ebi-a-GCST90002232	34.67901235
rs1412234	HDL cholesterol || id:ieu-b-109	34.6803044
rs12263737	Diagnoses - secondary ICD10: I10 Essential (primary) hypertension || id:ukb-b-12493	34.70252317
rs2781655	Type 2 diabetes, strict (exclude DM1) || id:finn-b-E4_DM2_STRICT	34.71928994
rs62246443	HDL cholesterol || id:ieu-b-109	34.72208292
rs17124112	HDL cholesterol || id:ieu-b-109	34.72512726
rs58298943	HDL cholesterol || id:ieu-b-109	34.72903205
rs2238435	Fasting glucose || id:ebi-a-GCST90002232	34.74792244
rs6905288	Fasting insulin || id:ebi-a-GCST90002238	34.74792244
rs1206760	Fasting insulin || id:ebi-a-GCST90002238	34.74792244
rs10910476	LDL cholesterol || id:ieu-b-110	34.76742046
rs7400002	triglycerides || id:ieu-b-111	34.84482589
rs13111599	HDL cholesterol || id:ieu-b-109	34.85861904
rs112694524	Type 2 diabetes, strict (exclude DM1) || id:finn-b-E4_DM2_STRICT	34.87172669
rs12162782	LDL cholesterol || id:ieu-b-110	34.90383827
rs254562	HDL cholesterol || id:ieu-b-109	34.9186832
rs1083470	HDL cholesterol || id:ieu-b-109	34.93314606
rs2364723	HDL cholesterol || id:ieu-b-109	34.93570679
rs68096471	Diagnoses - secondary ICD10: I10 Essential (primary) hypertension || id:ukb-b-12493	35.02356968
rs11014204	LDL cholesterol || id:ieu-b-110	35.03819708
rs12921195	HDL cholesterol || id:ieu-b-109	35.05169991
rs73151974	HDL cholesterol || id:ieu-b-109	35.07211765
rs6487237	Fasting insulin || id:ebi-a-GCST90002238	35.08284024
rs61596977	HDL cholesterol || id:ieu-b-109	35.1360693
rs76645731	triglycerides || id:ieu-b-111	35.14674549
rs11904650	triglycerides || id:ieu-b-111	35.17115778
rs5755799	triglycerides || id:ieu-b-111	35.19782265
rs9831084	triglycerides || id:ieu-b-111	35.23621397
rs56094641	Diagnoses - secondary ICD10: I10 Essential (primary) hypertension || id:ukb-b-12493	35.26320801
rs11171710	HDL cholesterol || id:ieu-b-109	35.34014797
rs11746801	triglycerides || id:ieu-b-111	35.36501643
rs138191773	triglycerides || id:ieu-b-111	35.48460045
rs12475332	HDL cholesterol || id:ieu-b-109	35.52574276

rs148150904	LDL cholesterol || id:ieu-b-110	35.54326034
rs12471768	LDL cholesterol || id:ieu-b-110	35.54472557
rs78588343	triglycerides || id:ieu-b-111	35.55017529
rs7622114	HDL cholesterol || id:ieu-b-109	35.56068643
rs2196808	HDL cholesterol || id:ieu-b-109	35.56098986
rs6506033	triglycerides || id:ieu-b-111	35.56564558
rs76247316	HDL cholesterol || id:ieu-b-109	35.60367504
rs9859117	triglycerides || id:ieu-b-111	35.61012129
rs150419156	triglycerides || id:ieu-b-111	35.61192345
rs11664106	triglycerides || id:ieu-b-111	35.62608981
rs62274099	triglycerides || id:ieu-b-111	35.66147225
rs76177300	Type 2 diabetes, strict (exclude DM1) || id:finn-b-E4_DM2_STRICT	35.69032402
rs9894946	LDL cholesterol || id:ieu-b-110	35.69202955
rs1431659	HDL cholesterol || id:ieu-b-109	35.69552378
rs60612724	LDL cholesterol || id:ieu-b-110	35.71376452
rs10804330	Diagnoses - secondary ICD10: I10 Essential (primary) hypertension || id:ukb-b-12493	35.7290442
rs1835346	triglycerides || id:ieu-b-111	35.83185267
rs57139556	Diagnoses - secondary ICD10: I10 Essential (primary) hypertension || id:ukb-b-12493	35.84075161
rs78447482	LDL cholesterol || id:ieu-b-110	35.87364305
rs76452347	Diagnoses - secondary ICD10: I10 Essential (primary) hypertension || id:ukb-b-12493	35.9191412
rs73025562	triglycerides || id:ieu-b-111	35.92626093
rs6562773	triglycerides || id:ieu-b-111	35.94260634
rs10631642	triglycerides || id:ieu-b-111	35.94460647
rs3794752	HDL cholesterol || id:ieu-b-109	36.00022443
rs35224557	HDL cholesterol || id:ieu-b-109	36.00073879
rs6705285	HDL cholesterol || id:ieu-b-109	36.04077576
rs6822044	Diagnoses - secondary ICD10: I10 Essential (primary) hypertension || id:ukb-b-12493	36.0748813
rs1347188	triglycerides || id:ieu-b-111	36.0986045
rs12530679	triglycerides || id:ieu-b-111	36.17263634
rs554146	HDL cholesterol || id:ieu-b-109	36.19222779
rs144155527	Type 2 diabetes, strict (exclude DM1) || id:finn-b-E4_DM2_STRICT	36.20027778
rs9368503	triglycerides || id:ieu-b-111	36.20273835
rs2098368	HDL cholesterol || id:ieu-b-109	36.23370812
rs61988556	LDL cholesterol || id:ieu-b-110	36.28842655
rs12902047	triglycerides || id:ieu-b-111	36.29886554
rs56902258	triglycerides || id:ieu-b-111	36.31586353
rs2240533	triglycerides || id:ieu-b-111	36.42306496

rs12998038	HDL cholesterol || id:ieu-b-109	36.46065778
rs6460894	HDL cholesterol || id:ieu-b-109	36.46720511
rs11226108	LDL cholesterol || id:ieu-b-110	36.46926322
rs118164457	Fasting insulin || id:ebi-a-GCST90002238	36.63434903
rs12504746	triglycerides || id:ieu-b-111	36.66224984
rs1970811	HDL cholesterol || id:ieu-b-109	36.66424101
rs3027167	HDL cholesterol || id:ieu-b-109	36.7104235
rs75032664	HDL cholesterol || id:ieu-b-109	36.76544206
rs117847213	HDL cholesterol || id:ieu-b-109	36.90202321
rs3808477	triglycerides || id:ieu-b-111	36.90354895
rs1420384	triglycerides || id:ieu-b-111	36.90596838
rs79357714	triglycerides || id:ieu-b-111	36.91016494
rs7725218	HDL cholesterol || id:ieu-b-109	36.96503329
rs147460434	HDL cholesterol || id:ieu-b-109	36.98477798
rs28510484	HDL cholesterol || id:ieu-b-109	37.00536333
rs7424120	triglycerides || id:ieu-b-111	37.02479631
rs960596	LDL cholesterol || id:ieu-b-110	37.09764544
rs3842753	Fasting glucose || id:ebi-a-GCST90002232	37.09917355
rs139386986	triglycerides || id:ieu-b-111	37.10530067
rs2745353	LDL cholesterol || id:ieu-b-110	37.13861541
rs348330	Fasting glucose || id:ebi-a-GCST90002232	37.21
rs12078100	LDL cholesterol || id:ieu-b-110	37.23989338
rs6066148	HDL cholesterol || id:ieu-b-109	37.24926921
rs77924615	Diagnoses - secondary ICD10: I10 Essential (primary) hypertension || id:ukb-b-12493	37.27976947
rs6489811	Fasting glucose || id:ebi-a-GCST90002232	37.34567901
rs8102873	triglycerides || id:ieu-b-111	37.45434654
rs4930163	LDL cholesterol || id:ieu-b-110	37.47704804
rs149142833	triglycerides || id:ieu-b-111	37.51395814
rs2820226	LDL cholesterol || id:ieu-b-110	37.62069442
rs7488780	HDL cholesterol || id:ieu-b-109	37.63599774
rs10053349	HDL cholesterol || id:ieu-b-109	37.78432559
rs11568318	LDL cholesterol || id:ieu-b-110	37.81480717
rs189548	Fasting glucose || id:ebi-a-GCST90002232	37.8225
rs140584594	LDL cholesterol || id:ieu-b-110	37.8378052
rs7786339	triglycerides || id:ieu-b-111	37.86354327
rs62128802	triglycerides || id:ieu-b-111	37.92280075
rs1351394	Fasting insulin || id:ebi-a-GCST90002238	38.02777778

rs34568880	LDL cholesterol || id:ieu-b-110	38.08179123
rs75179845	Fasting insulin || id:ebi-a-GCST90002238	38.08653061
rs2965169	HDL cholesterol || id:ieu-b-109	38.22031641
rs896854	Fasting glucose || id:ebi-a-GCST90002232	38.28515625
rs11434143	triglycerides || id:ieu-b-111	38.2989987
rs13389219	Type 2 diabetes, strict (exclude DM1) || id:finn-b-E4_DM2_STRICT	38.3530317
rs2098918	HDL cholesterol || id:ieu-b-109	38.38119216
rs6031587	LDL cholesterol || id:ieu-b-110	38.42417201
rs188502504	HDL cholesterol || id:ieu-b-109	38.47290285
rs183906992	HDL cholesterol || id:ieu-b-109	38.49311996
rs72911393	LDL cholesterol || id:ieu-b-110	38.55189755
rs12541643	Fasting glucose || id:ebi-a-GCST90002232	38.57063712
rs12475332	triglycerides || id:ieu-b-111	38.58740983
rs2417125	HDL cholesterol || id:ieu-b-109	38.61363089
rs11191559	Diagnoses - secondary ICD10: I10 Essential (primary) hypertension || id:ukb-b-12493	38.61673232
rs16948048	Diagnoses - secondary ICD10: I10 Essential (primary) hypertension || id:ukb-b-12493	38.62933125
rs6662924	Fasting glucose || id:ebi-a-GCST90002232	38.65595463
rs2780215	Fasting insulin || id:ebi-a-GCST90002238	38.71604938
rs193735	triglycerides || id:ieu-b-111	38.73431129
rs6031435	Diagnoses - secondary ICD10: I10 Essential (primary) hypertension || id:ukb-b-12493	38.86796933
rs117291242	HDL cholesterol || id:ieu-b-109	38.87218997
rs2302593	Fasting glucose || id:ebi-a-GCST90002232	38.87889273
rs62427982	triglycerides || id:ieu-b-111	39.00711289
rs8118848	Diagnoses - secondary ICD10: I10 Essential (primary) hypertension || id:ukb-b-12493	39.05232302
rs6855363	Fasting insulin || id:ebi-a-GCST90002238	39.0625
rs483808	triglycerides || id:ieu-b-111	39.09847516
rs62397245	triglycerides || id:ieu-b-111	39.15109667
rs61993685	triglycerides || id:ieu-b-111	39.17584337
rs34389637	triglycerides || id:ieu-b-111	39.20116505
rs13107325	LDL cholesterol || id:ieu-b-110	39.23820491
rs10865959	Fasting insulin || id:ebi-a-GCST90002238	39.34710744
rs1270076	HDL cholesterol || id:ieu-b-109	39.36480271
rs1240820	HDL cholesterol || id:ieu-b-109	39.39535532
rs9376511	triglycerides || id:ieu-b-111	39.53225262
rs5849920	LDL cholesterol || id:ieu-b-110	39.63648763
rs9289196	LDL cholesterol || id:ieu-b-110	39.70280337
rs275184	triglycerides || id:ieu-b-111	39.72264844

rs7036107	HDL cholesterol || id:ieu-b-109	39.73311217
rs7763350	Diagnoses - secondary ICD10: I10 Essential (primary) hypertension || id:ukb-b-12493	39.74970349
rs8353	Type 2 diabetes, strict (exclude DM1) || id:finn-b-E4_DM2_STRICT	39.75409365
rs11604462	Diagnoses - secondary ICD10: I10 Essential (primary) hypertension || id:ukb-b-12493	39.81921187
rs3974807	triglycerides || id:ieu-b-111	39.84308956
rs10245376	Diagnoses - secondary ICD10: I10 Essential (primary) hypertension || id:ukb-b-12493	39.85409392
rs12926107	triglycerides || id:ieu-b-111	39.86382236
rs10899490	triglycerides || id:ieu-b-111	39.87424039
rs6786846	Type 2 diabetes, strict (exclude DM1) || id:finn-b-E4_DM2_STRICT	39.92932521
rs72784786	triglycerides || id:ieu-b-111	39.97418028
rs460428	HDL cholesterol || id:ieu-b-109	40.01917121
rs185073199	HDL cholesterol || id:ieu-b-109	40.0739356
rs60229127	LDL cholesterol || id:ieu-b-110	40.08299627
rs55730499	Diagnoses - secondary ICD10: I10 Essential (primary) hypertension || id:ukb-b-12493	40.11920967
rs7133378	Fasting insulin || id:ebi-a-GCST90002238	40.3225
rs9902027	triglycerides || id:ieu-b-111	40.35945445
rs74090351	triglycerides || id:ieu-b-111	40.36256906
rs4916426	HDL cholesterol || id:ieu-b-109	40.42329093
rs28631087	LDL cholesterol || id:ieu-b-110	40.44048732
rs1292065	triglycerides || id:ieu-b-111	40.54477336
rs731839	Fasting insulin || id:ebi-a-GCST90002238	40.5567867
rs74862545	Type 2 diabetes, strict (exclude DM1) || id:finn-b-E4_DM2_STRICT	40.58161866
rs6532798	triglycerides || id:ieu-b-111	40.63765638
rs8007841	HDL cholesterol || id:ieu-b-109	40.6947501
rs157512	Fasting glucose || id:ebi-a-GCST90002232	40.71655329
rs2298214	HDL cholesterol || id:ieu-b-109	40.87868974
rs852388	triglycerides || id:ieu-b-111	40.92840232
rs151235402	triglycerides || id:ieu-b-111	40.98211873
rs11000468	triglycerides || id:ieu-b-111	41.08452206
rs3821843	Diagnoses - secondary ICD10: I10 Essential (primary) hypertension || id:ukb-b-12493	41.08456022
rs28590710	LDL cholesterol || id:ieu-b-110	41.11585797
rs117287238	triglycerides || id:ieu-b-111	41.15310457
rs330089	HDL cholesterol || id:ieu-b-109	41.19311274
rs1327235	Diagnoses - secondary ICD10: I10 Essential (primary) hypertension || id:ukb-b-12493	41.34650079
rs4875043	HDL cholesterol || id:ieu-b-109	41.35729977
rs12686780	HDL cholesterol || id:ieu-b-109	41.48494185
rs17476364	LDL cholesterol || id:ieu-b-110	41.50219622

rs2516331	HDL cholesterol || id:ieu-b-109	41.59632763
rs1350559	LDL cholesterol || id:ieu-b-110	41.60079459
rs36043408	triglycerides || id:ieu-b-111	41.60553414
rs32578	HDL cholesterol || id:ieu-b-109	41.62381294
rs11801879	Diagnoses - secondary ICD10: I10 Essential (primary) hypertension || id:ukb-b-12493	41.65460412
rs6918911	Diagnoses - secondary ICD10: I10 Essential (primary) hypertension || id:ukb-b-12493	41.71047418
rs2131919	triglycerides || id:ieu-b-111	41.79284607
rs62428831	HDL cholesterol || id:ieu-b-109	41.80419166
rs13066793	triglycerides || id:ieu-b-111	41.82146234
rs6939861	HDL cholesterol || id:ieu-b-109	41.88503178
rs4862423	Fasting glucose || id:ebi-a-GCST90002232	41.90858726
rs6517522	triglycerides || id:ieu-b-111	41.93979865
rs11722924	triglycerides || id:ieu-b-111	42.04427444
rs56348580	Type 2 diabetes, strict (exclude DM1) || id:finn-b-E4_DM2_STRICT	42.08899665
rs5843957	LDL cholesterol || id:ieu-b-110	42.22886213
rs111273322	LDL cholesterol || id:ieu-b-110	42.24848625
rs983663	HDL cholesterol || id:ieu-b-109	42.32099242
rs12045101	HDL cholesterol || id:ieu-b-109	42.61712968
rs7583067	HDL cholesterol || id:ieu-b-109	42.62784012
rs115458560	LDL cholesterol || id:ieu-b-110	42.68515672
rs77735929	Type 2 diabetes, strict (exclude DM1) || id:finn-b-E4_DM2_STRICT	42.71035503
rs35763453	triglycerides || id:ieu-b-111	42.74767659
rs10405944	triglycerides || id:ieu-b-111	42.76206226
rs8100204	Type 2 diabetes, strict (exclude DM1) || id:finn-b-E4_DM2_STRICT	42.81901716
rs1771582	HDL cholesterol || id:ieu-b-109	42.82177878
rs148827772	triglycerides || id:ieu-b-111	42.91161066
rs6667939	LDL cholesterol || id:ieu-b-110	43.165107
rs17569873	LDL cholesterol || id:ieu-b-110	43.24362646
rs2382825	triglycerides || id:ieu-b-111	43.25614183
rs1016988	LDL cholesterol || id:ieu-b-110	43.27145943
rs9884482	Fasting insulin || id:ebi-a-GCST90002238	43.28254848
rs7853377	HDL cholesterol || id:ieu-b-109	43.29728612
rs34045894	HDL cholesterol || id:ieu-b-109	43.32067496
rs200573817	HDL cholesterol || id:ieu-b-109	43.34552098
rs10749409	Diagnoses - secondary ICD10: I10 Essential (primary) hypertension || id:ukb-b-12493	43.50544052
rs35990695	LDL cholesterol || id:ieu-b-110	43.5617955
rs10152471	triglycerides || id:ieu-b-111	43.59579557

rs140288	triglycerides || id:ieu-b-111	43.60466408
rs11254464	HDL cholesterol || id:ieu-b-109	43.66092424
rs13144151	HDL cholesterol || id:ieu-b-109	43.69190391
rs3775380	Fasting insulin || id:ebi-a-GCST90002238	43.70679012
rs2236464	HDL cholesterol || id:ieu-b-109	43.75255767
rs4917675	HDL cholesterol || id:ieu-b-109	43.95730389
rs13066793	HDL cholesterol || id:ieu-b-109	43.98276321
rs35587371	Diagnoses - secondary ICD10: I10 Essential (primary) hypertension || id:ukb-b-12493	44.04430239
rs6075860	HDL cholesterol || id:ieu-b-109	44.09329825
rs11688682	HDL cholesterol || id:ieu-b-109	44.33346796
rs970069	triglycerides || id:ieu-b-111	44.45466823
rs3780181	LDL cholesterol || id:ieu-b-110	44.4766423
rs71330995	Type 2 diabetes, strict (exclude DM1) || id:finn-b-E4_DM2_STRICT	44.57243414
rs78132593	Fasting glucose || id:ebi-a-GCST90002232	44.64669421
rs11240358	triglycerides || id:ieu-b-111	44.67402148
rs9916613	HDL cholesterol || id:ieu-b-109	44.68379003
rs3796581	Diagnoses - secondary ICD10: I10 Essential (primary) hypertension || id:ukb-b-12493	44.68468907
rs74500135	HDL cholesterol || id:ieu-b-109	44.73180877
rs9832727	LDL cholesterol || id:ieu-b-110	44.85598173
rs2910949	HDL cholesterol || id:ieu-b-109	44.93583764
rs6598541	Fasting glucose || id:ebi-a-GCST90002232	44.96885813
rs9327468	HDL cholesterol || id:ieu-b-109	45.08120905
rs867939	triglycerides || id:ieu-b-111	45.13003204
rs878521	Type 2 diabetes, strict (exclude DM1) || id:finn-b-E4_DM2_STRICT	45.18265589
rs7178572	Fasting glucose || id:ebi-a-GCST90002232	45.1882716
rs11021232	HDL cholesterol || id:ieu-b-109	45.3024841
rs10448340	LDL cholesterol || id:ieu-b-110	45.3043305
rs62492368	Type 2 diabetes, strict (exclude DM1) || id:finn-b-E4_DM2_STRICT	45.38504155
rs954244	triglycerides || id:ieu-b-111	45.39573119
rs150460588	triglycerides || id:ieu-b-111	45.45120612
rs11100083	triglycerides || id:ieu-b-111	45.49327478
rs12888855	Fasting glucose || id:ebi-a-GCST90002232	45.5625
rs12360772	Diagnoses - secondary ICD10: I10 Essential (primary) hypertension || id:ukb-b-12493	45.59209239
rs62033400	LDL cholesterol || id:ieu-b-110	45.61961461
rs2066714	LDL cholesterol || id:ieu-b-110	45.67122849
rs9480889	triglycerides || id:ieu-b-111	45.7785505
rs11631178	HDL cholesterol || id:ieu-b-109	45.79174955

rs59037995	HDL cholesterol || id:ieu-b-109	45.83739046
rs6495122	LDL cholesterol || id:ieu-b-110	46.05305268
rs56397607	triglycerides || id:ieu-b-111	46.06992966
rs581080	triglycerides || id:ieu-b-111	46.07776894
rs286965	HDL cholesterol || id:ieu-b-109	46.10582748
rs9330353	Diagnoses - secondary ICD10: I10 Essential (primary) hypertension || id:ukb-b-12493	46.3200307
rs4412193	Diagnoses - secondary ICD10: I10 Essential (primary) hypertension || id:ukb-b-12493	46.48017263
rs6991641	Diagnoses - secondary ICD10: I10 Essential (primary) hypertension || id:ukb-b-12493	46.50111351
rs79634051	HDL cholesterol || id:ieu-b-109	46.53183097
rs2237029	triglycerides || id:ieu-b-111	46.68507787
rs1044808	triglycerides || id:ieu-b-111	46.69326213
rs7776054	LDL cholesterol || id:ieu-b-110	46.7112013
rs13264304	triglycerides || id:ieu-b-111	46.77135841
rs80276949	triglycerides || id:ieu-b-111	46.7777335
rs35889227	Fasting glucose || id:ebi-a-GCST90002232	46.81440443
rs2520096	HDL cholesterol || id:ieu-b-109	46.82823308
rs1567353	triglycerides || id:ieu-b-111	46.92045475
rs6693842	HDL cholesterol || id:ieu-b-109	46.98770444
rs2092203	triglycerides || id:ieu-b-111	47.07365812
rs4954192	LDL cholesterol || id:ieu-b-110	47.09965103
rs36036416	HDL cholesterol || id:ieu-b-109	47.13613316
rs7998259	Type 2 diabetes, strict (exclude DM1) || id:finn-b-E4_DM2_STRICT	47.14293686
rs10938397	Type 2 diabetes, strict (exclude DM1) || id:finn-b-E4_DM2_STRICT	47.20198903
rs9425589	triglycerides || id:ieu-b-111	47.26073114
rs6792725	triglycerides || id:ieu-b-111	47.26337866
rs6752845	triglycerides || id:ieu-b-111	47.29620719
rs3735533	Diagnoses - secondary ICD10: I10 Essential (primary) hypertension || id:ukb-b-12493	47.33895047
rs2943656	Type 2 diabetes, strict (exclude DM1) || id:finn-b-E4_DM2_STRICT	47.37407678
rs3732359	LDL cholesterol || id:ieu-b-110	47.41843486
rs1057394	Fasting glucose || id:ebi-a-GCST90002232	47.45679012
rs34940374	HDL cholesterol || id:ieu-b-109	47.57132149
rs880315	LDL cholesterol || id:ieu-b-110	47.58322647
rs150237291	HDL cholesterol || id:ieu-b-109	47.65510336
rs116857878	HDL cholesterol || id:ieu-b-109	47.89304889
rs9347737	HDL cholesterol || id:ieu-b-109	47.91570293
rs12258967	Diagnoses - secondary ICD10: I10 Essential (primary) hypertension || id:ukb-b-12493	47.9177425
rs76602912	HDL cholesterol || id:ieu-b-109	48.1147247

rs921971	triglycerides || id:ieu-b-111	48.12190295
rs1225053	HDL cholesterol || id:ieu-b-109	48.16187305
rs11257658	Type 2 diabetes, strict (exclude DM1) || id:finn-b-E4_DM2_STRICT	48.20649085
rs140064750	HDL cholesterol || id:ieu-b-109	48.28070754
rs2812208	triglycerides || id:ieu-b-111	48.36550364
rs13020929	LDL cholesterol || id:ieu-b-110	48.45586666
rs145730801	LDL cholesterol || id:ieu-b-110	48.50088095
rs17036126	Fasting insulin || id:ebi-a-GCST90002238	48.53444444
rs72644085	triglycerides || id:ieu-b-111	48.58636382
rs58123204	HDL cholesterol || id:ieu-b-109	48.73207024
rs55767272	triglycerides || id:ieu-b-111	48.82311422
rs28615248	LDL cholesterol || id:ieu-b-110	48.92003811
rs7708285	Fasting glucose || id:ebi-a-GCST90002232	49
rs742036	triglycerides || id:ieu-b-111	49.03659172
rs7704653	triglycerides || id:ieu-b-111	49.20318283
rs12440800	triglycerides || id:ieu-b-111	49.30220815
rs2759315	Diagnoses - secondary ICD10: I10 Essential (primary) hypertension || id:ukb-b-12493	49.36755423
rs77981966	Fasting glucose || id:ebi-a-GCST90002232	49.40081633
rs11600815	triglycerides || id:ieu-b-111	49.43385835
rs17713879	HDL cholesterol || id:ieu-b-109	49.46966698
rs4761234	triglycerides || id:ieu-b-111	49.47357274
rs10119644	HDL cholesterol || id:ieu-b-109	49.69909761
rs12055786	Fasting glucose || id:ebi-a-GCST90002232	49.82698962
rs10773000	triglycerides || id:ieu-b-111	49.83633596
rs4675812	triglycerides || id:ieu-b-111	50.0366219
rs149778057	triglycerides || id:ieu-b-111	50.11003963
rs860598	Fasting insulin || id:ebi-a-GCST90002238	50.1264
rs3790604	Diagnoses - secondary ICD10: I10 Essential (primary) hypertension || id:ukb-b-12493	50.2000096
rs144033177	HDL cholesterol || id:ieu-b-109	50.25398955
rs78508096	LDL cholesterol || id:ieu-b-110	50.32441706
rs73113806	Type 2 diabetes, strict (exclude DM1) || id:finn-b-E4_DM2_STRICT	50.3744074
rs76428106	HDL cholesterol || id:ieu-b-109	50.42843426
rs4599108	HDL cholesterol || id:ieu-b-109	50.4290843
rs28362901	HDL cholesterol || id:ieu-b-109	50.46133735
rs2237035	HDL cholesterol || id:ieu-b-109	50.50936422
rs11030107	triglycerides || id:ieu-b-111	50.52077641
rs2305746	triglycerides || id:ieu-b-111	50.53927762

rs77655131	Type 2 diabetes, strict (exclude DM1) || id:finn-b-E4_DM2_STRICT	50.61306122
rs698927	triglycerides || id:ieu-b-111	51.01268479
rs968050	HDL cholesterol || id:ieu-b-109	51.25298343
rs9496567	LDL cholesterol || id:ieu-b-110	51.34406531
rs689183	HDL cholesterol || id:ieu-b-109	51.35084895
rs138354839	HDL cholesterol || id:ieu-b-109	51.44421831
rs1316753	triglycerides || id:ieu-b-111	51.5500427
rs55831924	LDL cholesterol || id:ieu-b-110	51.57133439
rs10108282	HDL cholesterol || id:ieu-b-109	51.75174261
rs117139027	LDL cholesterol || id:ieu-b-110	51.86743106
rs10883026	triglycerides || id:ieu-b-111	52.02992103
rs4804101	HDL cholesterol || id:ieu-b-109	52.0867109
rs71647892	HDL cholesterol || id:ieu-b-109	52.1288403
rs3731696	triglycerides || id:ieu-b-111	52.15027739
rs10832963	LDL cholesterol || id:ieu-b-110	52.16230229
rs7158166	HDL cholesterol || id:ieu-b-109	52.1795392
rs551243	triglycerides || id:ieu-b-111	52.23392629
rs7665587	HDL cholesterol || id:ieu-b-109	52.33127615
rs35443	Diagnoses - secondary ICD10: I10 Essential (primary) hypertension || id:ukb-b-12493	52.35390933
rs116141873	Fasting insulin || id:ebi-a-GCST90002238	52.62395863
rs11558471	Type 2 diabetes, strict (exclude DM1) || id:finn-b-E4_DM2_STRICT	52.82174745
rs7924036	HDL cholesterol || id:ieu-b-109	52.85465013
rs2839671	Fasting glucose || id:ebi-a-GCST90002232	52.89256198
rs1043897	triglycerides || id:ieu-b-111	52.95189728
rs10231941	LDL cholesterol || id:ieu-b-110	53.01593908
rs4253750	triglycerides || id:ieu-b-111	53.0950355
rs6123685	HDL cholesterol || id:ieu-b-109	53.17858096
rs10882099	Type 2 diabetes, strict (exclude DM1) || id:finn-b-E4_DM2_STRICT	53.23593964
rs3747973	HDL cholesterol || id:ieu-b-109	53.43288489
rs2726111	HDL cholesterol || id:ieu-b-109	53.49052532
rs9933509	HDL cholesterol || id:ieu-b-109	53.64354772
rs117230571	HDL cholesterol || id:ieu-b-109	53.83434194
rs10305457	Fasting glucose || id:ebi-a-GCST90002232	53.93066406
rs1055582	HDL cholesterol || id:ieu-b-109	53.9346658
rs13118477	triglycerides || id:ieu-b-111	54.07259763
rs13269725	HDL cholesterol || id:ieu-b-109	54.13534946
rs2643826	Diagnoses - secondary ICD10: I10 Essential (primary) hypertension || id:ukb-b-12493	54.21037565

rs73541184	Type 2 diabetes, strict (exclude DM1) || id:finn-b-E4_DM2_STRICT	54.40638469
rs440677	LDL cholesterol || id:ieu-b-110	54.63424405
rs740746	Diagnoses - secondary ICD10: I10 Essential (primary) hypertension || id:ukb-b-12493	54.64504563
rs10513688	triglycerides || id:ieu-b-111	54.75303601
rs116006942	HDL cholesterol || id:ieu-b-109	54.8027991
rs367677	HDL cholesterol || id:ieu-b-109	54.9981793
rs4666384	LDL cholesterol || id:ieu-b-110	55.1357778
rs61729990	triglycerides || id:ieu-b-111	55.13610651
rs2298632	HDL cholesterol || id:ieu-b-109	55.43539708
rs4134963	triglycerides || id:ieu-b-111	55.5839499
rs3814883	triglycerides || id:ieu-b-111	55.63805859
rs12205778	HDL cholesterol || id:ieu-b-109	55.67179656
rs41785	triglycerides || id:ieu-b-111	55.74936204
rs6808574	Fasting glucose || id:ebi-a-GCST90002232	55.80968858
rs17326656	triglycerides || id:ieu-b-111	55.84594387
rs6538804	Fasting glucose || id:ebi-a-GCST90002232	55.85595568
rs146534110	LDL cholesterol || id:ieu-b-110	55.8677995
rs77605964	HDL cholesterol || id:ieu-b-109	55.94211849
rs71311871	LDL cholesterol || id:ieu-b-110	55.94503053
rs2586116	HDL cholesterol || id:ieu-b-109	56.0379904
rs62459095	triglycerides || id:ieu-b-111	56.14586254
rs12246352	LDL cholesterol || id:ieu-b-110	56.35583577
rs11078597	triglycerides || id:ieu-b-111	56.46215043
rs1037117	triglycerides || id:ieu-b-111	56.47819001
rs213494	triglycerides || id:ieu-b-111	56.5557212
rs17265513	Fasting glucose || id:ebi-a-GCST90002232	56.60770975
rs35140741	triglycerides || id:ieu-b-111	56.74437848
rs9904004	HDL cholesterol || id:ieu-b-109	57.10706178
rs12462109	HDL cholesterol || id:ieu-b-109	57.27209557
rs11712037	Type 2 diabetes, strict (exclude DM1) || id:finn-b-E4_DM2_STRICT	57.4016686
rs59104589	HDL cholesterol || id:ieu-b-109	57.41794031
rs3820897	triglycerides || id:ieu-b-111	57.4322727
rs11610045	Fasting glucose || id:ebi-a-GCST90002232	57.44044321
rs1046317	Type 2 diabetes, strict (exclude DM1) || id:finn-b-E4_DM2_STRICT	57.54110868
rs1045241	HDL cholesterol || id:ieu-b-109	57.9952024
rs2723065	HDL cholesterol || id:ieu-b-109	58.13344663
rs2268840	HDL cholesterol || id:ieu-b-109	58.32978547

rs1556562	LDL cholesterol || id:ieu-b-110	58.42308731
rs9604045	HDL cholesterol || id:ieu-b-109	58.54374952
rs13354321	triglycerides || id:ieu-b-111	58.6834995
rs12880341	triglycerides || id:ieu-b-111	58.7217392
rs1281959	HDL cholesterol || id:ieu-b-109	59.09726314
rs1010759	LDL cholesterol || id:ieu-b-110	59.12379361
rs4074448	HDL cholesterol || id:ieu-b-109	59.38152316
rs140164052	HDL cholesterol || id:ieu-b-109	59.5889728
rs28577186	triglycerides || id:ieu-b-111	59.61095854
rs1446585	HDL cholesterol || id:ieu-b-109	59.73377962
rs112108602	triglycerides || id:ieu-b-111	59.76909365
rs12046972	HDL cholesterol || id:ieu-b-109	59.839247
rs3732356	HDL cholesterol || id:ieu-b-109	59.85011267
rs9929977	LDL cholesterol || id:ieu-b-110	59.89696719
rs830620	HDL cholesterol || id:ieu-b-109	59.97994494
rs77960347	LDL cholesterol || id:ieu-b-110	60.40278786
rs1168114	HDL cholesterol || id:ieu-b-109	60.45508225
rs2071887	triglycerides || id:ieu-b-111	60.52540338
rs10242866	triglycerides || id:ieu-b-111	60.59874672
rs16851397	Fasting glucose || id:ebi-a-GCST90002232	60.61734694
rs9561643	triglycerides || id:ieu-b-111	60.73372431
rs2250802	LDL cholesterol || id:ieu-b-110	60.85624161
rs10504477	HDL cholesterol || id:ieu-b-109	60.87663498
rs507666	Fasting glucose || id:ebi-a-GCST90002232	60.98866213
rs568546	Diagnoses - secondary ICD10: I10 Essential (primary) hypertension || id:ukb-b-12493	61.03386418
rs35278712	LDL cholesterol || id:ieu-b-110	61.1409326
rs141062196	HDL cholesterol || id:ieu-b-109	61.16628476
rs4782568	LDL cholesterol || id:ieu-b-110	61.30792313
rs71571682	HDL cholesterol || id:ieu-b-109	61.35998445
rs10797119	triglycerides || id:ieu-b-111	61.42527734
rs8014289	HDL cholesterol || id:ieu-b-109	61.43451602
rs7274718	triglycerides || id:ieu-b-111	61.7770172
rs13097947	HDL cholesterol || id:ieu-b-109	61.82384791
rs71269068	HDL cholesterol || id:ieu-b-109	61.87274646
rs11099097	LDL cholesterol || id:ieu-b-110	62.4055462
rs2238162	LDL cholesterol || id:ieu-b-110	62.42427663
rs2804894	HDL cholesterol || id:ieu-b-109	62.78608706

rs12656497	Diagnoses - secondary ICD10: I10 Essential (primary) hypertension || id:ukb-b-12493	62.82704366
rs10210970	triglycerides || id:ieu-b-111	63.08880287
rs1938566	triglycerides || id:ieu-b-111	63.09482286
rs2043085	LDL cholesterol || id:ieu-b-110	63.13517324
rs11601507	LDL cholesterol || id:ieu-b-110	63.22295865
rs16928809	HDL cholesterol || id:ieu-b-109	63.34238308
rs13107325	triglycerides || id:ieu-b-111	63.38923837
rs557933	HDL cholesterol || id:ieu-b-109	63.87057807
rs729761	triglycerides || id:ieu-b-111	64.5213002
rs6780171	Type 2 diabetes, strict (exclude DM1) || id:finn-b-E4_DM2_STRICT	64.54817737
rs16913693	Fasting glucose || id:ebi-a-GCST90002232	64.6547272
rs12229011	HDL cholesterol || id:ieu-b-109	64.91669761
rs55966194	triglycerides || id:ieu-b-111	65.09884823
rs7239575	triglycerides || id:ieu-b-111	65.17894734
rs11640494	HDL cholesterol || id:ieu-b-109	65.28218757
rs114165349	LDL cholesterol || id:ieu-b-110	65.35822141
rs3814883	HDL cholesterol || id:ieu-b-109	65.4582292
rs6026744	Diagnoses - secondary ICD10: I10 Essential (primary) hypertension || id:ukb-b-12493	65.53713013
rs703966	HDL cholesterol || id:ieu-b-109	65.81989545
rs13101504	triglycerides || id:ieu-b-111	66.00630237
rs12424054	triglycerides || id:ieu-b-111	66.0110507
rs3829109	Fasting glucose || id:ebi-a-GCST90002232	66.4225
rs1474696	Fasting insulin || id:ebi-a-GCST90002238	66.69444444
rs28746806	HDL cholesterol || id:ieu-b-109	66.7250823
rs9622830	HDL cholesterol || id:ieu-b-109	67.11678892
rs3918226	Diagnoses - secondary ICD10: I10 Essential (primary) hypertension || id:ukb-b-12493	67.26885054
rs8126001	triglycerides || id:ieu-b-111	67.27251138
rs2383208	Type 2 diabetes, strict (exclude DM1) || id:finn-b-E4_DM2_STRICT	67.62898199
rs13108218	LDL cholesterol || id:ieu-b-110	67.82397197
rs140107293	triglycerides || id:ieu-b-111	67.99287278
rs2487294	triglycerides || id:ieu-b-111	68.0414758
rs4865796	Fasting insulin || id:ebi-a-GCST90002238	68.0625
rs1132274	HDL cholesterol || id:ieu-b-109	68.07895543
rs34138141	HDL cholesterol || id:ieu-b-109	68.09176204
rs2519093	triglycerides || id:ieu-b-111	68.20689329
rs7497304	Diagnoses - secondary ICD10: I10 Essential (primary) hypertension || id:ukb-b-12493	68.21064505
rs10774439	HDL cholesterol || id:ieu-b-109	68.24046172

rs10786114	HDL cholesterol || id:ieu-b-109	68.60421681
rs7794796	HDL cholesterol || id:ieu-b-109	68.61268429
rs3823376	LDL cholesterol || id:ieu-b-110	68.91744075
rs1229984	LDL cholesterol || id:ieu-b-110	68.91923203
rs1125873	HDL cholesterol || id:ieu-b-109	69.04922953
rs6142206	HDL cholesterol || id:ieu-b-109	69.34340221
rs71368855	triglycerides || id:ieu-b-111	69.46664528
rs6934962	HDL cholesterol || id:ieu-b-109	69.57831805
rs4930352	HDL cholesterol || id:ieu-b-109	69.58695146
rs79287178	triglycerides || id:ieu-b-111	69.60650479
rs75634664	triglycerides || id:ieu-b-111	69.84240119
rs2160994	LDL cholesterol || id:ieu-b-110	69.8776494
rs2773469	triglycerides || id:ieu-b-111	70.08321852
rs373798	triglycerides || id:ieu-b-111	70.23589947
rs2247355	HDL cholesterol || id:ieu-b-109	70.29014443
rs3822855	LDL cholesterol || id:ieu-b-110	70.53036447
rs13087167	HDL cholesterol || id:ieu-b-109	71.38027319
rs55993634	Type 2 diabetes, strict (exclude DM1) || id:finn-b-E4_DM2_STRICT	71.98735253
rs7735249	triglycerides || id:ieu-b-111	72.27020255
rs117233107	triglycerides || id:ieu-b-111	72.305281
rs61830291	triglycerides || id:ieu-b-111	72.37982984
rs28642213	Type 2 diabetes, strict (exclude DM1) || id:finn-b-E4_DM2_STRICT	72.39413962
rs78470967	Type 2 diabetes, strict (exclude DM1) || id:finn-b-E4_DM2_STRICT	72.45892275
rs2435307	HDL cholesterol || id:ieu-b-109	72.67717215
rs13137144	HDL cholesterol || id:ieu-b-109	72.69828482
rs7134375	triglycerides || id:ieu-b-111	73.10597967
rs75721796	triglycerides || id:ieu-b-111	73.21862432
rs35511051	LDL cholesterol || id:ieu-b-110	73.25384392
rs3103310	triglycerides || id:ieu-b-111	73.25543447
rs17035646	Diagnoses - secondary ICD10: I10 Essential (primary) hypertension || id:ukb-b-12493	73.42863332
rs7569317	LDL cholesterol || id:ieu-b-110	73.73754353
rs2937124	triglycerides || id:ieu-b-111	73.92115573
rs459193	Fasting insulin || id:ebi-a-GCST90002238	74.28798186
rs11619319	Fasting glucose || id:ebi-a-GCST90002232	74.8225
rs55935382	HDL cholesterol || id:ieu-b-109	74.9022811
rs4976033	triglycerides || id:ieu-b-111	75.18552781
rs35980001	LDL cholesterol || id:ieu-b-110	75.42924429

rs2963468	HDL cholesterol || id:ieu-b-109	75.54416797
rs11614202	HDL cholesterol || id:ieu-b-109	75.90937972
rs6732741	LDL cholesterol || id:ieu-b-110	75.94763306
rs1534696	HDL cholesterol || id:ieu-b-109	75.95414781
rs4969179	triglycerides || id:ieu-b-111	75.99308052
rs167479	Diagnoses - secondary ICD10: I10 Essential (primary) hypertension || id:ukb-b-12493	76.15263596
rs2737265	LDL cholesterol || id:ieu-b-110	76.23726474
rs12185242	triglycerides || id:ieu-b-111	76.87294097
rs2814982	HDL cholesterol || id:ieu-b-109	77.12333182
rs13076933	LDL cholesterol || id:ieu-b-110	77.14298938
rs9834932	LDL cholesterol || id:ieu-b-110	77.31178343
rs633185	Diagnoses - secondary ICD10: I10 Essential (primary) hypertension || id:ukb-b-12493	77.5508007
rs2244278	triglycerides || id:ieu-b-111	77.73634043
rs28752924	triglycerides || id:ieu-b-111	77.87026005
rs113177823	LDL cholesterol || id:ieu-b-110	78.05401873
rs10750766	HDL cholesterol || id:ieu-b-109	78.21859142
rs6674544	Fasting insulin || id:ebi-a-GCST90002238	78.3225
rs3778321	Fasting glucose || id:ebi-a-GCST90002232	78.44897959
rs10750766	triglycerides || id:ieu-b-111	78.53977572
rs696825	triglycerides || id:ieu-b-111	78.61891989
rs10775406	triglycerides || id:ieu-b-111	78.88627345
rs1799831	triglycerides || id:ieu-b-111	80.0189295
rs72801474	triglycerides || id:ieu-b-111	80.06098452
rs7734476	LDL cholesterol || id:ieu-b-110	80.25738684
rs116734477	LDL cholesterol || id:ieu-b-110	80.3188428
rs186413375	triglycerides || id:ieu-b-111	80.51840615
rs150555490	triglycerides || id:ieu-b-111	80.76625611
rs2595701	Fasting glucose || id:ebi-a-GCST90002232	81
rs7562734	LDL cholesterol || id:ieu-b-110	81.43256823
rs3104412	LDL cholesterol || id:ieu-b-110	81.49953825
rs2461385	Fasting glucose || id:ebi-a-GCST90002232	81.75173611
rs7947951	triglycerides || id:ieu-b-111	81.93634894
rs1275985	Diagnoses - secondary ICD10: I10 Essential (primary) hypertension || id:ukb-b-12493	82.20506836
rs45551238	Type 2 diabetes, strict (exclude DM1) || id:finn-b-E4_DM2_STRICT	82.2856942
rs11621792	LDL cholesterol || id:ieu-b-110	82.33546009
rs71603401	triglycerides || id:ieu-b-111	82.49575228
rs112233856	HDL cholesterol || id:ieu-b-109	83.05967382

rs1473886	triglycerides || id:ieu-b-111	83.32944866
rs8081548	HDL cholesterol || id:ieu-b-109	83.34539387
rs3184504	Diagnoses - secondary ICD10: I10 Essential (primary) hypertension || id:ukb-b-12493	83.84619014
rs60856912	triglycerides || id:ieu-b-111	84.07454914
rs13379043	HDL cholesterol || id:ieu-b-109	84.11810799
rs2068888	LDL cholesterol || id:ieu-b-110	84.12563551
rs11274835	triglycerides || id:ieu-b-111	84.29511534
rs7170463	HDL cholesterol || id:ieu-b-109	84.34786845
rs62102718	triglycerides || id:ieu-b-111	84.48741391
rs4614	HDL cholesterol || id:ieu-b-109	84.53970872
rs1009360	triglycerides || id:ieu-b-111	84.74065097
rs1045241	triglycerides || id:ieu-b-111	84.8570749
rs35000407	Fasting insulin || id:ebi-a-GCST90002238	84.90306122
rs235314	HDL cholesterol || id:ieu-b-109	85.40019027
rs1047891	HDL cholesterol || id:ieu-b-109	85.59285067
rs9375459	Diagnoses - secondary ICD10: I10 Essential (primary) hypertension || id:ukb-b-12493	85.76023951
rs1064173	triglycerides || id:ieu-b-111	86.01241969
rs1760801	triglycerides || id:ieu-b-111	86.34525714
rs17309930	HDL cholesterol || id:ieu-b-109	86.36314993
rs267738	HDL cholesterol || id:ieu-b-109	87.18354277
rs57512892	HDL cholesterol || id:ieu-b-109	87.2361117
rs72631343	LDL cholesterol || id:ieu-b-110	88.67635612
rs532436	HDL cholesterol || id:ieu-b-109	89.05393022
rs7018475	Type 2 diabetes, strict (exclude DM1) || id:finn-b-E4_DM2_STRICT	89.38842975
rs2075423	Fasting glucose || id:ebi-a-GCST90002232	89.69204152
rs7215055	triglycerides || id:ieu-b-111	90.09905917
rs4650994	HDL cholesterol || id:ieu-b-109	90.25204935
rs11456863	HDL cholesterol || id:ieu-b-109	90.29690139
rs13269725	triglycerides || id:ieu-b-111	90.3739421
rs907866	HDL cholesterol || id:ieu-b-109	91.11249155
rs11118310	triglycerides || id:ieu-b-111	91.44612221
rs1544980	triglycerides || id:ieu-b-111	92.26163975
rs6602912	LDL cholesterol || id:ieu-b-110	92.35629265
rs6113722	Fasting glucose || id:ebi-a-GCST90002232	92.85950413
rs62112763	triglycerides || id:ieu-b-111	92.87419936
rs11381821	HDL cholesterol || id:ieu-b-109	93.2600054
rs6108171	Diagnoses - secondary ICD10: I10 Essential (primary) hypertension || id:ukb-b-12493	93.4110641

rs4871624	HDL cholesterol || id:ieu-b-109	93.59328363
rs17050272	LDL cholesterol || id:ieu-b-110	94.45209453
rs6475606	LDL cholesterol || id:ieu-b-110	94.62425695
rs2307111	HDL cholesterol || id:ieu-b-109	94.78624871
rs112108223	Type 2 diabetes, strict (exclude DM1) || id:finn-b-E4_DM2_STRICT	95.26787396
rs142288236	HDL cholesterol || id:ieu-b-109	95.36293826
rs9348441	Fasting glucose || id:ebi-a-GCST90002232	95.60493827
rs10838693	Fasting glucose || id:ebi-a-GCST90002232	96.69444444
rs62271373	triglycerides || id:ieu-b-111	96.76859107
rs2159607	HDL cholesterol || id:ieu-b-109	96.93276986
rs2237897	Type 2 diabetes, strict (exclude DM1) || id:finn-b-E4_DM2_STRICT	97.23333581
rs2699805	triglycerides || id:ieu-b-111	97.62407522
rs174583	Fasting glucose || id:ebi-a-GCST90002232	97.66089965
rs1471251	HDL cholesterol || id:ieu-b-109	98.30243271
rs62117489	triglycerides || id:ieu-b-111	98.31678952
rs72926946	HDL cholesterol || id:ieu-b-109	98.58608248
rs2287622	LDL cholesterol || id:ieu-b-110	98.7276036
rs72647336	HDL cholesterol || id:ieu-b-109	98.9459628
rs62271373	HDL cholesterol || id:ieu-b-109	99.02118002
rs73243877	HDL cholesterol || id:ieu-b-109	99.04820964
rs55714927	LDL cholesterol || id:ieu-b-110	99.07052214
rs13402475	HDL cholesterol || id:ieu-b-109	99.53618059
rs17326656	HDL cholesterol || id:ieu-b-109	100.0339489
rs635769	HDL cholesterol || id:ieu-b-109	100.1096964
rs2068888	HDL cholesterol || id:ieu-b-109	100.2957488
rs150474434	LDL cholesterol || id:ieu-b-110	100.3229683
rs7700842	Diagnoses - secondary ICD10: I10 Essential (primary) hypertension || id:ukb-b-12493	101.0806097
rs67981690	triglycerides || id:ieu-b-111	101.1943172
rs9884390	LDL cholesterol || id:ieu-b-110	101.8757656
rs113344423	triglycerides || id:ieu-b-111	102.600823
rs10811660	Fasting glucose || id:ebi-a-GCST90002232	102.7458678
rs72831345	Diagnoses - secondary ICD10: I10 Essential (primary) hypertension || id:ukb-b-12493	102.9119546
rs79153732	triglycerides || id:ieu-b-111	104.0018387
rs1801689	LDL cholesterol || id:ieu-b-110	104.2813347
rs7202323	LDL cholesterol || id:ieu-b-110	106.2572394
rs2081194	triglycerides || id:ieu-b-111	106.299184
rs34682685	triglycerides || id:ieu-b-111	106.4881451

rs7746081	LDL cholesterol || id:ieu-b-110	107.2042058
rs12928099	HDL cholesterol || id:ieu-b-109	107.3613336
rs61435086	HDL cholesterol || id:ieu-b-109	107.3831788
rs4330777	HDL cholesterol || id:ieu-b-109	109.3752192
rs13389219	Fasting insulin || id:ebi-a-GCST90002238	109.6980609
rs6824451	HDL cholesterol || id:ieu-b-109	109.9411491
rs11206374	triglycerides || id:ieu-b-111	110.9371249
rs150564454	triglycerides || id:ieu-b-111	111.6000178
rs7012637	Fasting glucose || id:ebi-a-GCST90002232	112.1107266
rs10233430	HDL cholesterol || id:ieu-b-109	113.0270806
rs11664369	HDL cholesterol || id:ieu-b-109	113.3090871
rs2800710	HDL cholesterol || id:ieu-b-109	113.3298485
rs2267373	triglycerides || id:ieu-b-111	113.7953676
rs11218738	HDL cholesterol || id:ieu-b-109	113.8035299
rs2740488	LDL cholesterol || id:ieu-b-110	113.9421272
rs133015	HDL cholesterol || id:ieu-b-109	114.0900113
rs2298624	HDL cholesterol || id:ieu-b-109	114.3231366
rs11603349	Fasting glucose || id:ebi-a-GCST90002232	115.0743802
rs9940128	Type 2 diabetes, strict (exclude DM1) || id:finn-b-E4_DM2_STRICT	116.402323
rs117733303	LDL cholesterol || id:ieu-b-110	117.3182769
rs11065385	LDL cholesterol || id:ieu-b-110	117.6953554
rs2302263	HDL cholesterol || id:ieu-b-109	118.1158472
rs57996145	triglycerides || id:ieu-b-111	118.3151874
rs17184382	triglycerides || id:ieu-b-111	118.9385213
rs10513801	HDL cholesterol || id:ieu-b-109	119.8809752
rs12784552	Fasting glucose || id:ebi-a-GCST90002232	120.2677778
rs2111216	HDL cholesterol || id:ieu-b-109	120.4631004
rs6018652	HDL cholesterol || id:ieu-b-109	120.6423744
rs1604038	Fasting glucose || id:ebi-a-GCST90002232	121
rs75152587	HDL cholesterol || id:ieu-b-109	123.431779
rs62117487	HDL cholesterol || id:ieu-b-109	123.453372
rs7451008	Type 2 diabetes, strict (exclude DM1) || id:finn-b-E4_DM2_STRICT	123.8865784
rs2642438	LDL cholesterol || id:ieu-b-110	124.0965497
rs12411732	HDL cholesterol || id:ieu-b-109	124.4370507
rs62102718	HDL cholesterol || id:ieu-b-109	125.8145846
rs2618566	LDL cholesterol || id:ieu-b-110	128.3132132
rs1183851	LDL cholesterol || id:ieu-b-110	129.8267998

rs112001035	HDL cholesterol || id:ieu-b-109	130.4229425
rs147627829	HDL cholesterol || id:ieu-b-109	130.9520495
rs1801689	triglycerides || id:ieu-b-111	131.0265281
rs9647335	HDL cholesterol || id:ieu-b-109	131.0701123
rs7012814	Fasting insulin || id:ebi-a-GCST90002238	132.8559557
rs75662196	HDL cholesterol || id:ieu-b-109	133.5834921
rs7186799	HDL cholesterol || id:ieu-b-109	134.1022088
rs10830963	Type 2 diabetes, strict (exclude DM1) || id:finn-b-E4_DM2_STRICT	135.6297282
rs10974438	Fasting glucose || id:ebi-a-GCST90002232	135.6539792
rs3924313	HDL cholesterol || id:ieu-b-109	136.4305594
rs326222	triglycerides || id:ieu-b-111	136.712511
rs4765148	triglycerides || id:ieu-b-111	137.4378121
rs771481	HDL cholesterol || id:ieu-b-109	137.5598242
rs6800707	triglycerides || id:ieu-b-111	138.614277
rs11045171	HDL cholesterol || id:ieu-b-109	139.0463684
rs6765484	HDL cholesterol || id:ieu-b-109	139.2245197
rs77009508	triglycerides || id:ieu-b-111	140.8066774
rs13235365	HDL cholesterol || id:ieu-b-109	145.0387592
rs144311893	HDL cholesterol || id:ieu-b-109	146.385192
rs4760254	triglycerides || id:ieu-b-111	146.5177752
rs1260326	Fasting insulin || id:ebi-a-GCST90002238	147.8144044
rs200571020	LDL cholesterol || id:ieu-b-110	149.9719402
rs4841580	triglycerides || id:ieu-b-111	150.3921445
rs114165349	triglycerides || id:ieu-b-111	151.9971652
rs1820176	Fasting glucose || id:ebi-a-GCST90002232	152.5225
rs684773	triglycerides || id:ieu-b-111	153.847371
rs3127580	LDL cholesterol || id:ieu-b-110	154.1411721
rs5112	LDL cholesterol || id:ieu-b-110	155.7901256
rs2302263	triglycerides || id:ieu-b-111	156.1055345
rs2081687	triglycerides || id:ieu-b-111	156.2159315
rs9987289	LDL cholesterol || id:ieu-b-110	157.0190592
rs186696265	triglycerides || id:ieu-b-111	158.5296185
rs12740374	HDL cholesterol || id:ieu-b-109	158.710991
rs34596921	LDL cholesterol || id:ieu-b-110	158.7146166
rs36057735	HDL cholesterol || id:ieu-b-109	160.2420644
rs11429307	HDL cholesterol || id:ieu-b-109	160.3140982
rs61805075	HDL cholesterol || id:ieu-b-109	161.2458834

rs2611867	LDL cholesterol || id:ieu-b-110	161.7506254
rs114165349	HDL cholesterol || id:ieu-b-109	162.12033
rs11239536	HDL cholesterol || id:ieu-b-109	166.0223975
rs79153732	HDL cholesterol || id:ieu-b-109	166.5276346
rs2271308	HDL cholesterol || id:ieu-b-109	167.2295223
rs12928099	triglycerides || id:ieu-b-111	167.2679331
rs2498786	HDL cholesterol || id:ieu-b-109	167.5069247
rs597808	LDL cholesterol || id:ieu-b-110	167.9694126
rs7140110	triglycerides || id:ieu-b-111	169.0211291
rs13125101	Diagnoses - secondary ICD10: I10 Essential (primary) hypertension || id:ukb-b-12493	169.4527084
rs56130071	LDL cholesterol || id:ieu-b-110	170.9141351
rs2943646	Fasting insulin || id:ebi-a-GCST90002238	173.1301939
rs6709904	LDL cholesterol || id:ieu-b-110	173.7091066
rs4307732	LDL cholesterol || id:ieu-b-110	174.0171259
rs6680227	LDL cholesterol || id:ieu-b-110	175.6468806
rs3860846	triglycerides || id:ieu-b-111	175.8138839
rs2073547	LDL cholesterol || id:ieu-b-110	176.8962598
rs2642438	HDL cholesterol || id:ieu-b-109	177.038712
rs17168486	Fasting glucose || id:ebi-a-GCST90002232	177.7777778
rs559355	HDL cholesterol || id:ieu-b-109	178.7716411
rs6916318	triglycerides || id:ieu-b-111	180.2731475
rs2256609	HDL cholesterol || id:ieu-b-109	181.5898093
rs7163757	Fasting glucose || id:ebi-a-GCST90002232	183.9414063
rs7817574	HDL cholesterol || id:ieu-b-109	185.2097147
rs7903146	Fasting glucose || id:ebi-a-GCST90002232	185.8199446
rs10642257	triglycerides || id:ieu-b-111	186.6424504
rs61352607	HDL cholesterol || id:ieu-b-109	189.3757168
rs2297409	HDL cholesterol || id:ieu-b-109	192.2535657
rs12208357	LDL cholesterol || id:ieu-b-110	193.5327601
rs3184504	HDL cholesterol || id:ieu-b-109	194.1727202
rs17138358	HDL cholesterol || id:ieu-b-109	195.3345254
rs35919498	LDL cholesterol || id:ieu-b-110	196.5771966
rs11708067	Fasting glucose || id:ebi-a-GCST90002232	197.4025
rs72555385	triglycerides || id:ieu-b-111	200.4415297
rs17585887	triglycerides || id:ieu-b-111	200.9329025
rs13389219	HDL cholesterol || id:ieu-b-109	203.0736302
rs10773049	triglycerides || id:ieu-b-111	203.2560874

rs4738684	LDL cholesterol || id:ieu-b-110	203.8420927
rs516316	LDL cholesterol || id:ieu-b-110	205.2026701
rs174564	LDL cholesterol || id:ieu-b-110	212.2213972
rs140584594	HDL cholesterol || id:ieu-b-109	212.9799596
rs79220007	LDL cholesterol || id:ieu-b-110	213.7954024
rs188247550	triglycerides || id:ieu-b-111	216.2709336
rs183130	LDL cholesterol || id:ieu-b-110	218.3115755
rs13108218	triglycerides || id:ieu-b-111	219.8587039
rs2925979	triglycerides || id:ieu-b-111	220.1203699
rs10838524	Fasting glucose || id:ebi-a-GCST90002232	221.265625
rs6874202	LDL cholesterol || id:ieu-b-110	223.4381682
rs76895963	Type 2 diabetes, strict (exclude DM1) || id:finn-b-E4_DM2_STRICT	223.5862984
rs3745683	HDL cholesterol || id:ieu-b-109	226.6239022
rs2043085	triglycerides || id:ieu-b-111	227.6417111
rs150844304	HDL cholesterol || id:ieu-b-109	228.3202374
rs4969141	HDL cholesterol || id:ieu-b-109	240.3917615
rs12446515	triglycerides || id:ieu-b-111	244.834897
rs4784709	HDL cholesterol || id:ieu-b-109	245.1156957
rs35493868	HDL cholesterol || id:ieu-b-109	245.1184439
rs34518086	HDL cholesterol || id:ieu-b-109	249.2185539
rs9650069	Fasting glucose || id:ebi-a-GCST90002232	252.4567901
rs2068888	triglycerides || id:ieu-b-111	254.1972338
rs78058190	HDL cholesterol || id:ieu-b-109	255.3435133
rs78058190	triglycerides || id:ieu-b-111	255.3941343
rs6882076	triglycerides || id:ieu-b-111	257.8909827
rs113740515	HDL cholesterol || id:ieu-b-109	260.7136279
rs10822163	triglycerides || id:ieu-b-111	261.1369233
rs150224153	HDL cholesterol || id:ieu-b-109	263.4339717
rs10487796	Fasting glucose || id:ebi-a-GCST90002232	266.0976563
rs1260326	LDL cholesterol || id:ieu-b-110	266.9938103
rs2066714	HDL cholesterol || id:ieu-b-109	267.8433024
rs4731701	triglycerides || id:ieu-b-111	268.2556577
rs6469605	HDL cholesterol || id:ieu-b-109	271.3304484
rs375972689	LDL cholesterol || id:ieu-b-110	274.0014553
rs1260326	Fasting glucose || id:ebi-a-GCST90002232	275.1695502
rs3775228	triglycerides || id:ieu-b-111	275.9150703
rs556107	LDL cholesterol || id:ieu-b-110	283.6103325

rs1883711	LDL cholesterol || id:ieu-b-110	284.5949449
rs2792751	HDL cholesterol || id:ieu-b-109	286.2751074
rs78484485	triglycerides || id:ieu-b-111	297.3784646
rs71445274	HDL cholesterol || id:ieu-b-109	300.5532286
rs75609851	HDL cholesterol || id:ieu-b-109	310.8789444
rs998584	HDL cholesterol || id:ieu-b-109	320.514517
rs4871603	HDL cholesterol || id:ieu-b-109	326.6264993
rs34042070	LDL cholesterol || id:ieu-b-110	327.7816818
rs2925979	HDL cholesterol || id:ieu-b-109	328.9265556
rs686030	HDL cholesterol || id:ieu-b-109	330.7753556
rs77542162	LDL cholesterol || id:ieu-b-110	333.0737766
rs367070	HDL cholesterol || id:ieu-b-109	333.9489412
rs41272086	HDL cholesterol || id:ieu-b-109	334.8454544
rs11429307	triglycerides || id:ieu-b-111	337.1029918
rs28383314	triglycerides || id:ieu-b-111	342.9077146
rs13389219	triglycerides || id:ieu-b-111	344.5164084
rs7707394	LDL cholesterol || id:ieu-b-110	346.5377903
rs964184	LDL cholesterol || id:ieu-b-110	353.2850297
rs3768321	HDL cholesterol || id:ieu-b-109	356.6972335
rs308	HDL cholesterol || id:ieu-b-109	357.2741319
rs2943645	triglycerides || id:ieu-b-111	378.3199737
rs472495	LDL cholesterol || id:ieu-b-110	381.0759835
rs59781045	HDL cholesterol || id:ieu-b-109	382.0327679
rs1077835	triglycerides || id:ieu-b-111	388.0532311
rs75609851	triglycerides || id:ieu-b-111	393.3111348
rs55781197	HDL cholesterol || id:ieu-b-109	396.0267754
rs141469619	HDL cholesterol || id:ieu-b-109	399.0776699
rs2738447	LDL cholesterol || id:ieu-b-110	401.3013828
rs4803773	HDL cholesterol || id:ieu-b-109	403.6774394
rs998584	triglycerides || id:ieu-b-111	406.4256324
rs921919	HDL cholesterol || id:ieu-b-109	407.2789476
rs10162642	HDL cholesterol || id:ieu-b-109	418.7343742
rs2519093	LDL cholesterol || id:ieu-b-110	430.3157598
rs118039278	LDL cholesterol || id:ieu-b-110	461.9291602
rs145947882	triglycerides || id:ieu-b-111	465.272516
rs2943645	HDL cholesterol || id:ieu-b-109	477.9850779
rs12575456	HDL cholesterol || id:ieu-b-109	480.7890084

rs13107325	HDL cholesterol || id:ieu-b-109	491.4103451
rs34872471	Type 2 diabetes, strict (exclude DM1) || id:finn-b-E4_DM2_STRICT	494.6565614
rs6073958	triglycerides || id:ieu-b-111	498.9277862
rs308	triglycerides || id:ieu-b-111	520.9388838
rs139974673	triglycerides || id:ieu-b-111	525.9341116
rs150423652	triglycerides || id:ieu-b-111	540.659723
rs174566	triglycerides || id:ieu-b-111	543.8335587
rs7000494	triglycerides || id:ieu-b-111	546.3172703
rs11122450	triglycerides || id:ieu-b-111	559.1450201
rs6544713	LDL cholesterol || id:ieu-b-110	584.0056706
rs676210	HDL cholesterol || id:ieu-b-109	634.8188255
rs6073958	HDL cholesterol || id:ieu-b-109	648.8034669
rs80005209	HDL cholesterol || id:ieu-b-109	654.3896352
rs9987289	HDL cholesterol || id:ieu-b-109	702.3821176
rs8107974	LDL cholesterol || id:ieu-b-110	718.5642742
rs145947882	HDL cholesterol || id:ieu-b-109	731.304783
rs58542926	triglycerides || id:ieu-b-111	744.8538981
rs878521	Fasting glucose || id:ebi-a-GCST90002232	753.5025
rs4263041	LDL cholesterol || id:ieu-b-110	777.1967261
rs174566	HDL cholesterol || id:ieu-b-109	793.8477305
rs429358	HDL cholesterol || id:ieu-b-109	822.0467898
rs28601761	LDL cholesterol || id:ieu-b-110	842.617318
rs12916	LDL cholesterol || id:ieu-b-110	852.8497757
rs116843064	HDL cholesterol || id:ieu-b-109	888.3288219
rs676210	triglycerides || id:ieu-b-111	900.6491814
rs2281718	HDL cholesterol || id:ieu-b-109	931.6038869
rs116843064	triglycerides || id:ieu-b-111	986.9024224
rs2740488	HDL cholesterol || id:ieu-b-109	1008.474726
rs5112	triglycerides || id:ieu-b-111	1028.751549
rs79600951	HDL cholesterol || id:ieu-b-109	1073.771315
rs8086351	HDL cholesterol || id:ieu-b-109	1121.739567
rs77960347	HDL cholesterol || id:ieu-b-109	1228.37674
rs483082	triglycerides || id:ieu-b-111	1351.901495
rs9436661	triglycerides || id:ieu-b-111	1399.394273
rs964184	HDL cholesterol || id:ieu-b-109	1423.586686
rs934197	LDL cholesterol || id:ieu-b-110	1427.541597
rs343	HDL cholesterol || id:ieu-b-109	1491.28612

rs537183	Fasting glucose || id:ebi-a-GCST90002232	1521
rs343	triglycerides || id:ieu-b-111	1535.784922
rs4970834	LDL cholesterol || id:ieu-b-110	1541.813891
rs200046586	LDL cholesterol || id:ieu-b-110	1560.116181
rs10830963	Fasting glucose || id:ebi-a-GCST90002232	1650.925208
rs2240466	triglycerides || id:ieu-b-111	1654.248812
rs480823	triglycerides || id:ieu-b-111	1752.522572
rs6999569	triglycerides || id:ieu-b-111	1889.262684
rs11591147	LDL cholesterol || id:ieu-b-110	1930.425903
rs1551891	LDL cholesterol || id:ieu-b-110	2250.134791
rs1601934	HDL cholesterol || id:ieu-b-109	2380.869424
rs4665972	triglycerides || id:ieu-b-111	2424.997763
rs35980001	HDL cholesterol || id:ieu-b-109	2572.902856
rs143020224	LDL cholesterol || id:ieu-b-110	2773.119661
rs61905078	triglycerides || id:ieu-b-111	2774.64273
rs9989419	HDL cholesterol || id:ieu-b-109	5569.868743
