# Supplementary material for: Small dense low density lipoprotein predominance in patients with type 2 diabetes mellitus using Mendelian randomization
Source: PLoS One. 2024 Feb 8;19(2):e0298070. doi: 10.1371/journal.pone.0298070 (PMC10852223; doi:10.1371/journal.pone.0298070)
Supplement: S2 Table — (PDF) [file pone.0298070.s002.pdf]

Supplementary Table 2

Results of UVMR analysis.

Exposure	Outcome	Method	b	95% CI		p
T2DM	Concentration of small LDL particles	MR Egger	-0.017716076	-0.141006848	0.105574697	0.779258369
		Weighted median	-0.02645286	-0.046869287	-0.006036434	0.01110096
		Inverse variance weighted	-0.027695151	-0.084361599	0.028971297	0.338098011
		Simple mode	-0.020759321	-0.06133504	0.019816397	0.320208088
		Weighted mode	-0.030963564	-0.056196975	-0.005730153	0.019437987
	Cholesterol in small LDL	MR Egger	-0.015411765	-0.150037617	0.119214086	0.823279962
		Weighted median	-0.019735915	-0.039682881	0.000211052	0.05246945
		Inverse variance weighted	-0.040047129	-0.101995112	0.021900854	0.205130594
		Simple mode	-0.013947425	-0.054726634	0.026831783	0.505332237
		Weighted mode	-0.030037836	-0.054133744	-0.005941929	0.017672799
Fasting glucose	Concentration of small LDL particles	MR Egger	-0.120814002	-0.352431461	0.110803457	0.310585194
		Weighted median	0.02917043	-0.053626437	0.111967297	0.489858509
		Inverse variance weighted	0.008902614	-0.119103075	0.136908304	0.891572088
		Simple mode	0.027721551	-0.138424103	0.193867205	0.744731006
		Weighted mode	0.027721551	-0.050859874	0.106302977	0.491830095
	Cholesterol in small LDL	MR Egger	-0.121894595	-0.358543772	0.114754581	0.316626252
		Weighted median	0.018619779	-0.058810336	0.096049894	0.637408503
		Inverse variance weighted	-0.00607092	-0.136437982	0.124296143	0.92727561
		Simple mode	-0.00896801	-0.16372587	0.145789851	0.909932421
		Weighted mode	0.003067852	-0.071268195	0.077403898	0.935786433
Fasting insulin	Concentration of small LDL particles	MR Egger	-0.385595563	-1.177061884	0.405870759	0.345999222
		Weighted median	0.23946881	0.068020151	0.41091747	0.006188818
		Inverse variance weighted	0.18009595	-0.088744576	0.448936476	0.189182381
		Simple mode	0.241646886	-0.099249353	0.582543125	0.173028156
		Weighted mode	0.208263319	-0.060051159	0.476577797	0.136676961
	Cholesterol in small LDL	MR Egger	-0.518832222	-1.264739479	0.227075034	0.181247277
		Weighted median	0.009762974	-0.163430899	0.182956848	0.912024265
		Inverse variance weighted	0.041802775	-0.212331091	0.29593664	0.747147658
		Simple mode	0.046633529	-0.325866917	0.419133976	0.807523225
		Weighted mode	0.036111124	-0.253045542	0.325267791	0.807984486
Hypertension	Concentration of small LDL particles	MR Egger	-1.741783869	-2.931386886	-0.552180852	0.00549075
		Weighted median	-1.17730486	-1.520220247	-0.834389472	1.71E-11
		Inverse variance weighted	-1.300674081	-1.61716434	-0.984183823	7.95E-16

		Simple mode	-1.439751128	-2.232894933	-0.646607323	0.000686696
		Weighted mode	-1.366852329	-2.093290366	-0.640414292	0.000450797
		MR Egger	-1.216678422	-2.268414632	-0.164942212	0.026597761
		Weighted median	-1.303610785	-1.648798384	-0.958423187	1.34E-13
	Cholesterol in small LDL	Inverse variance weighted	-1.321880138	-1.600595288	-1.043164987	1.46E-20
		Simple mode	-1.706743088	-2.441004995	-0.972481182	2.23E-05
		Weighted mode	-1.36002003	-2.018597435	-0.701442626	0.000134858
		MR Egger	-0.214795286	-0.316446873	-0.113143699	4.34E-05
		Weighted median	-0.142076633	-0.18909059	-0.095062676	3.16E-09
	Concentration of small LDL particles	Inverse variance weighted	-0.173135252	-0.239480973	-0.106789532	3.14E-07
		Simple mode	-0.141384381	-0.294816289	0.012047527	0.071769057
HDL cholesterol		Weighted mode	-0.192660954	-0.250279176	-0.135042732	2.03E-10
		MR Egger	-0.205694736	-0.308857949	-0.102531524	0.00011196
		Weighted median	-0.096317529	-0.136302446	-0.056332612	2.34E-06
	Cholesterol in small LDL	Inverse variance weighted	-0.130374343	-0.19794299	-0.062805697	0.000155674
		Simple mode	0.058051243	-0.061749633	0.177852119	0.342902182
		Weighted mode	-0.069711494	-0.109220388	-0.030202601	0.000611177
		MR Egger	0.856607973	0.774320823	0.938895124	3.21E-47
		Weighted median	0.897142567	0.855383617	0.938901517	0
	Concentration of small LDL particles	Inverse variance weighted	0.881020948	0.826028884	0.936013012	1.97E-216
		Simple mode	0.871146103	0.786277958	0.956014247	1.31E-46
LDL cholesterol		Weighted mode	0.898706337	0.863612377	0.933800298	4.63E-103
		MR Egger	0.922271312	0.850475336	0.994067287	9.72E-59
		Weighted median	0.886059862	0.837935998	0.934183725	3.54E-285
	Cholesterol in small LDL	Inverse variance weighted	0.896585088	0.848558419	0.944611757	4.09E-293
		Simple mode	0.914445642	0.809239129	1.019652155	1.91E-38
		Weighted mode	0.900811994	0.850286942	0.951337046	5.64E-79
		MR Egger	0.400156257	0.329666476	0.470646038	2.78E-24
		Weighted median	0.372786351	0.326409566	0.419163136	6.36E-56
	Concentration of small LDL particles	Inverse variance weighted	0.419592851	0.373400761	0.46578494	6.58E-71
		Simple mode	0.422824546	0.298612049	0.547037043	1.22E-10
Triglycerides		Weighted mode	0.374628572	0.325601931	0.423655212	3.19E-38
		MR Egger	0.251355254	0.179839818	0.32287069	3.35E-11
		Weighted median	0.24986872	0.20089028	0.29884716	1.54E-23
	Cholesterol in small LDL	Inverse variance weighted	0.279314364	0.232408648	0.326220081	1.78E-31
		Simple mode	0.300016895	0.148266096	0.451767695	0.000131072
		Weighted mode	0.231825258	0.164437385	0.299213131	8.02E-11
