# Supplementary material for: Small dense low density lipoprotein predominance in patients with type 2 diabetes mellitus using Mendelian randomization
Source: PLoS One. 2024 Feb 8;19(2):e0298070. doi: 10.1371/journal.pone.0298070 (PMC10852223; doi:10.1371/journal.pone.0298070)
Supplement: S3 Table — (PDF) [file pone.0298070.s003.pdf]

Supplementary Table 3

Mendelian randomization analysis of individual SNPS (with concentration of small LDL particles as the outcome)

Exposure	SNP	b	se	p	95% CI	
T2DM	rs1046317	-0.075348337	0.052630285	0.15224298	-0.178503696	0.027807021
T2DM	rs10743152	-0.012694581	0.06503379	0.845236552	-0.14016081	0.114771647
T2DM	rs10830963	-0.00020221	0.035180775	0.995413997	-0.069156529	0.068752109
T2DM	rs10882099	0.071588706	0.053371954	0.179817577	-0.033020325	0.176197736
T2DM	rs10938397	0.059829515	0.056290027	0.287836608	-0.050498938	0.170157968
T2DM	rs112108223	-0.051908601	0.051078458	0.309509252	-0.152022378	0.048205177
T2DM	rs11257658	-0.026023185	0.061301522	0.671193012	-0.146174169	0.094127799
T2DM	rs11263763	-0.033198947	0.062646316	0.596151774	-0.155985726	0.089587832
T2DM	rs112694524	-0.03971212	0.045123852	0.378821733	-0.128154869	0.04873063
T2DM	rs11558471	0.085451106	0.054713022	0.118333786	-0.021786418	0.192688629
T2DM	rs11712037	0.024747571	0.058476352	0.67214462	-0.089866079	0.139361221
T2DM	rs117657619	-0.170300566	0.087601132	0.051889992	-0.341998784	0.001397652
T2DM	rs12449219	-0.051267712	0.047425172	0.279686785	-0.14422105	0.041685625
T2DM	rs12967878	0.09896	0.064471948	0.124800746	-0.027405018	0.225325018
T2DM	rs13389219	0.320726629	0.059957507	8.83E-08	0.203209915	0.438243343
T2DM	rs144155527	0.020675392	0.071433518	0.77224817	-0.119334303	0.160685088
T2DM	rs1798085	0.071959699	0.069379599	0.29964817	-0.064024314	0.207943712
T2DM	rs182788819	-0.009206472	0.10899302	0.932683982	-0.222832792	0.204419848
T2DM	rs2237897	-0.010251514	0.052971746	0.846545515	-0.114076135	0.093573108
T2DM	rs2303700	0.034332	0.066373185	0.60497671	-0.095759443	0.164423443
T2DM	rs2383208	0.04608344	0.0431512	0.285541854	-0.038492912	0.130659792
T2DM	rs2781655	-0.094811097	0.069760183	0.174114757	-0.231541055	0.041918862
T2DM	rs28553330	0.007427124	0.102979735	0.942504668	-0.194413157	0.209267405
T2DM	rs28642213	-0.036556873	0.047520518	0.441723505	-0.129697088	0.056583343
T2DM	rs2943656	0.067595681	0.056146335	0.228620605	-0.042451136	0.177642497
T2DM	rs34872471	-0.036490318	0.014912931	0.014409435	-0.065719663	-0.007260974
T2DM	rs3887925	-0.156461248	0.068401149	0.022172095	-0.290527501	-0.022394995
T2DM	rs429358	-2.132681427	0.070269988	2.53E-202	-2.270410603	-1.994952251
T2DM	rs45551238	0.133141176	0.115233551	0.247925485	-0.092716584	0.358998937
T2DM	rs498475	-0.063177896	0.065587348	0.335415318	-0.191729098	0.065373305
T2DM	rs5215	-0.030978837	0.069746204	0.656923146	-0.167681396	0.105723722
T2DM	rs55993634	-0.047872904	0.046337181	0.301537288	-0.138693779	0.042947971
T2DM	rs56348580	0.156905732	0.057254522	0.006134671	0.044686869	0.269124596
T2DM	rs6017317	-1.76E-05	0.074096354	0.999810888	-0.145246416	0.145211292
T2DM	rs62137406	-0.02274301	0.172202568	0.894927928	-0.360260043	0.314774023

T2DM	rs62492368	0.154240885	0.058845833	0.008764624	0.038903052	0.269578719
T2DM	rs6780171	-0.027511809	0.047467979	0.562193373	-0.120549047	0.06552543
T2DM	rs6786846	-0.045772033	0.058587858	0.434652776	-0.160604235	0.069060169
T2DM	rs7018475	-0.026601049	0.04131757	0.519692617	-0.107583486	0.054381388
T2DM	rs71330995	0.050387823	0.050237069	0.315860447	-0.048076832	0.148852478
T2DM	rs7224685	0.024868789	0.069207919	0.719344914	-0.110778733	0.160516311
T2DM	rs73113806	-0.019747057	0.054907771	0.719116015	-0.127366287	0.087872174
T2DM	rs73541184	0.03466489	0.052985632	0.512962418	-0.069186948	0.138516728
T2DM	rs7451008	-0.012345547	0.036799063	0.737258984	-0.084471709	0.059780616
T2DM	rs745805	-0.110786988	0.065440241	0.090465183	-0.23904986	0.017475884
T2DM	rs74862545	-0.003876589	0.054573256	0.943370222	-0.110840171	0.103086992
T2DM	rs7507893	-0.064790344	0.069240753	0.349414496	-0.200502219	0.070921532
T2DM	rs76177300	-0.031175108	0.066993939	0.641686407	-0.162483229	0.100133013
T2DM	rs76895963	0.087827777	0.031724798	0.005632766	0.025647173	0.15000838
T2DM	rs77655131	-0.074837751	0.063510743	0.238657937	-0.199318807	0.049643305
T2DM	rs77735929	0.005666286	0.096051195	0.952958202	-0.182594056	0.193926627
T2DM	rs78470967	-0.003950676	0.043051937	0.926884476	-0.088332473	0.080431121
T2DM	rs7998259	0.047519636	0.056911443	0.403731751	-0.064026793	0.159066065
T2DM	rs8100204	-0.579156923	0.061537333	4.89E-21	-0.699770096	-0.45854375
T2DM	rs8353	0.032743952	0.062027823	0.597574742	-0.088830581	0.154318484
T2DM	rs878521	0.0357	0.053434228	0.504062689	-0.069031087	0.140431087
T2DM	rs9505086	0.050578906	0.066398906	0.446213368	-0.07956295	0.180720763
T2DM	rs9940128	-0.071703741	0.035590136	0.043935225	-0.141460408	-0.001947075
T2DM	All - Inverse variance weighted	-0.027695151	0.028911453	0.338098011	-0.084361599	0.028971297
T2DM	All - MR Egger	-0.017716076	0.062903455	0.779258369	-0.141006848	0.105574697
Fasting glucose	rs10305457	0.095937021	0.300799574	0.749771999	-0.493630145	0.685504187
Fasting glucose	rs1057394	0.434737097	0.341537903	0.203060284	-0.234677194	1.104151387
Fasting glucose	rs10811660	0.192469058	0.244486099	0.431141812	-0.286723695	0.671661812
Fasting glucose	rs10830963	-0.000344699	0.059971373	0.995413997	-0.117888591	0.117199192
Fasting glucose	rs10838524	-0.310148739	0.175007983	0.076361751	-0.653164387	0.032866908
Fasting glucose	rs10838693	0.098347458	0.249462147	0.693405334	-0.39059835	0.587293266
Fasting glucose	rs10974438	-0.364461616	0.218655051	0.095547469	-0.793025515	0.064102283
Fasting glucose	rs11603349	0.020347034	0.241784746	0.932934384	-0.453551068	0.494245136
Fasting glucose	rs11610045	0.326173611	0.286907639	0.255597118	-0.236165361	0.888512583
Fasting glucose	rs11619319	0.142019653	0.28815896	0.622117328	-0.422771908	0.706811214
Fasting glucose	rs11708067	0.252082918	0.171994662	0.142745339	-0.085026619	0.589192456
Fasting glucose	rs12055786	0.1291	0.351204167	0.713177145	-0.559260167	0.817460167
Fasting glucose	rs12541643	0.75184661	0.352924576	0.033144192	0.060114441	1.44357878

Fasting glucose	rs1260326	-1.705067376	0.149773404	5.01E-30	-1.998623248	-1.411511504
Fasting glucose	rs12784552	0.101324316	0.218299392	0.642538137	-0.326542492	0.529191125
Fasting glucose	rs12888855	0.275432593	0.365428148	0.451013937	-0.440806578	0.991671763
Fasting glucose	rs12898997	-2.002153061	0.449106122	8.27E-06	-2.882401061	-1.121905061
Fasting glucose	rs157512	0.895798507	0.362098507	0.013364399	0.186085433	1.605511582
Fasting glucose	rs1604038	-0.289371717	0.231336364	0.210981911	-0.74279099	0.164047556
Fasting glucose	rs16851397	0.429556575	0.300261468	0.152542482	-0.158955902	1.018069052
Fasting glucose	rs16913693	-0.440611675	0.337263959	0.191406449	-1.101649036	0.220425685
Fasting glucose	rs17168486	-0.214309286	0.19543	0.272814614	-0.597352086	0.168733514
Fasting glucose	rs17265513	0.731	0.327332911	0.02553573	0.089427494	1.372572506
Fasting glucose	rs17270243	0.98975	0.467725962	0.034337196	0.073007115	1.906492885
Fasting glucose	rs17437560	-0.051801143	0.402686857	0.897643485	-0.841067383	0.737465097
Fasting glucose	rs174583	1.889619048	0.257144643	2.00E-13	1.385615548	2.393622548
Fasting glucose	rs1820176	-0.07248502	0.185351417	0.695746924	-0.435773798	0.290803757
Fasting glucose	rs189548	0.604570732	0.373126829	0.105171687	-0.126757854	1.335899317
Fasting glucose	rs194518	0.524485294	0.407281373	0.197825559	-0.273786196	1.322756784
Fasting glucose	rs2075423	0.077767081	0.272526708	0.775371556	-0.456385267	0.611919429
Fasting glucose	rs2238435	-0.252129464	0.380042857	0.507059188	-0.997013464	0.492754536
Fasting glucose	rs2461385	-0.129506452	0.26361659	0.623236757	-0.646194968	0.387182065
Fasting glucose	rs2595701	0.207084127	0.235566667	0.37935288	-0.25462654	0.668794794
Fasting glucose	rs2657879	1.132848739	0.448969748	0.011628615	0.252868034	2.012829445
Fasting glucose	rs2839671	-0.1347325	0.338359375	0.690487565	-0.797916875	0.528451875
Fasting glucose	rs348330	-0.118727049	0.352459836	0.73622794	-0.809548328	0.57209423
Fasting glucose	rs35889227	0.381414615	0.330366923	0.248287794	-0.266104554	1.028933785
Fasting glucose	rs3778321	-0.023505376	0.290177419	0.935439197	-0.592253118	0.545242366
Fasting glucose	rs3829109	0.076529448	0.287045399	0.78976872	-0.486079534	0.639138429
Fasting glucose	rs3842753	0.331656716	0.341304478	0.331183552	-0.33730006	1.000613493
Fasting glucose	rs39713	0.252991716	0.424676331	0.551356738	-0.579373893	1.085357325
Fasting glucose	rs4760278	1.674863636	0.44955	0.000194817	0.793745636	2.555981636
Fasting glucose	rs4862423	-0.32984878	0.345729268	0.340049883	-1.007478146	0.347780585
Fasting glucose	rs507666	2.964158537	0.325618902	8.77E-20	2.325945488	3.602371585
Fasting glucose	rs537183	0.044620965	0.065446606	0.495370554	-0.083654383	0.172896314
Fasting glucose	rs58925536	0.150745752	0.373558824	0.686551361	-0.581429542	0.882921046
Fasting glucose	rs6113722	-0.242554245	0.255408019	0.342278143	-0.743153962	0.258045472
Fasting glucose	rs6489811	-0.183521818	0.376618182	0.626053691	-0.921693455	0.554649818
Fasting glucose	rs6538804	0.099261268	0.301095775	0.741651775	-0.490886451	0.689408986
Fasting glucose	rs6598541	-0.045515351	0.379454386	0.904523179	-0.789245947	0.698215246
Fasting glucose	rs6662924	0.804734266	0.381074825	0.03470801	0.057827608	1.551640923

Fasting glucose	rs6808574	-0.330014961	0.335530709	0.325331348	-0.98765515	0.327625228
Fasting glucose	rs7012637	-0.251378333	0.231672778	0.277896097	-0.705456978	0.202700311
Fasting glucose	rs7095788	-0.027867547	0.406372642	0.945326855	-0.824357925	0.76862283
Fasting glucose	rs7163757	-0.539032258	0.192617051	0.005134598	-0.916561677	-0.161502839
Fasting glucose	rs7178572	0.478284298	0.376881818	0.204420936	-0.260404066	1.216972661
Fasting glucose	rs7708285	-0.067095113	0.336245113	0.841838582	-0.726135534	0.591945308
Fasting glucose	rs77981966	-0.456971545	0.31783252	0.150498001	-1.079923285	0.165980195
Fasting glucose	rs78132593	0.84455102	0.340972789	0.013253391	0.176244354	1.512857687
Fasting glucose	rs7903146	-0.469833977	0.17530888	0.007361441	-0.813439382	-0.126228571
Fasting glucose	rs878521	0.058134426	0.087013115	0.504062689	-0.112411279	0.228680131
Fasting glucose	rs896854	0.025363838	0.417391919	0.951544422	-0.792724323	0.843452
Fasting glucose	rs9348441	-0.115182955	0.267375568	0.666620436	-0.639239068	0.408873159
Fasting glucose	rs9650069	0.153647902	0.157913287	0.330558734	-0.15586214	0.463157944
Fasting glucose	All - Inverse variance weighted	0.008902614	0.065309025	0.891572088	-0.119103075	0.136908304
Fasting glucose	All - MR Egger	-0.120814002	0.118172173	0.310585194	-0.352431461	0.110803457
Fasting insulin	rs10050393	0.268866667	0.46343	0.561802189	-0.639456133	1.177189467
Fasting insulin	rs10865959	-0.511788406	0.321305072	0.111195507	-1.141546348	0.117969536
Fasting insulin	rs116141873	0.27864486	0.226174299	0.217952184	-0.164656766	0.721946486
Fasting insulin	rs11708067	-0.524705926	0.358003704	0.142745339	-1.226393185	0.176981333
Fasting insulin	rs11727676	0.356316749	0.344882759	0.301532179	-0.319653458	1.032286956
Fasting insulin	rs118164457	0.619724638	0.298817391	0.038086345	0.034042551	1.205406725
Fasting insulin	rs1206760	0.550151786	0.373482143	0.140741947	-0.181873214	1.282176786
Fasting insulin	rs12454712	0.421878873	0.299709859	0.159242355	-0.165552451	1.009310197
Fasting insulin	rs1260326	-2.081510823	0.18284026	5.01E-30	-2.439877732	-1.723143913
Fasting insulin	rs13258890	-0.330028125	0.377040625	0.381404251	-1.06902775	0.4089715
Fasting insulin	rs13389219	1.137854271	0.212713568	8.83E-08	0.720935678	1.554772864
Fasting insulin	rs1351394	-0.021935676	0.373081081	0.953114594	-0.753174595	0.709303243
Fasting insulin	rs1474696	1.325333333	0.282236735	2.66E-06	0.772149333	1.878517333
Fasting insulin	rs17036126	0.108755024	0.294544976	0.711955835	-0.468553129	0.686063177
Fasting insulin	rs17331151	1.997283951	0.402016667	6.76E-07	1.209331284	2.785236617
Fasting insulin	rs2108349	0.31135913	0.385142609	0.418845641	-0.443520383	1.066238643
Fasting insulin	rs2780215	-0.082906378	0.225546684	0.71318667	-0.524977878	0.359165122
Fasting insulin	rs2845885	0.203036275	0.44379902	0.64731458	-0.666809804	1.072882353
Fasting insulin	rs2943646	0.2431012	0.1724204	0.158559488	-0.094842784	0.581045184
Fasting insulin	rs35000407	0.160912016	0.247360853	0.515359432	-0.323915256	0.645739287
Fasting insulin	rs3775380	0.222279832	0.348745378	0.523883883	-0.461261109	0.905820773
Fasting insulin	rs459193	0.72438674	0.262348619	0.00575963	0.210183448	1.238590033
Fasting insulin	rs4865796	-0.222634545	0.271649091	0.412463171	-0.755066764	0.309797673

Fasting insulin	rs5017305	0.040684234	0.40109781	0.919207376	-0.745467474	0.826835942
Fasting insulin	rs62271373	0.755894531	0.346692188	0.029234899	0.076377844	1.435411219
Fasting insulin	rs6487237	0.307383117	0.335145455	0.359057066	-0.349501974	0.964268208
Fasting insulin	rs6674544	0.419562147	0.239014124	0.079193234	-0.048905537	0.888029831
Fasting insulin	rs6855363	-0.4406656	0.3530288	0.211942214	-1.132602048	0.251270848
Fasting insulin	rs6905288	1.034535714	0.371667857	0.005377665	0.306066714	1.763004714
Fasting insulin	rs7012814	-0.210708676	0.190393151	0.268422347	-0.583879251	0.1624619
Fasting insulin	rs7133378	1.756503937	0.349059055	4.85E-07	1.072348189	2.440659685
Fasting insulin	rs73013411	1.069166667	0.341253889	0.001729979	0.400309044	1.738024289
Fasting insulin	rs731839	0.114812397	0.36177438	0.750971127	-0.594265388	0.823890182
Fasting insulin	rs75179845	-1.030810185	0.405679167	0.011055196	-1.825941352	-0.235679019
Fasting insulin	rs7903146	1.049025862	0.391422414	0.007361441	0.281837931	1.816213793
Fasting insulin	rs860598	-0.394270621	0.318151977	0.215252017	-1.017848497	0.229307254
Fasting insulin	rs972283	1.414447619	0.393379048	0.000323601	0.643424686	2.185470552
Fasting insulin	rs9884482	0.6543728	0.3426472	0.056164541	-0.017215712	1.325961312
Fasting insulin	All - Inverse variance weighted	0.18009595	0.137163534	0.189182381	-0.088744576	0.448936476
Fasting insulin	All - MR Egger	-0.385595563	0.403809348	0.345999222	-1.177061884	0.405870759
Hypertension	rs10245376	0.509432597	0.982691834	0.604175559	-1.416643397	2.435508592
Hypertension	rs10749409	0.353634229	0.942969653	0.707644213	-1.494586292	2.201854749
Hypertension	rs1077394	1.319073242	1.058288693	0.212609879	-0.755172596	3.39331908
Hypertension	rs10804330	0.450482278	1.037520645	0.664149642	-1.583058187	2.484022742
Hypertension	rs11191559	-3.256632812	0.997552855	0.001096103	-5.211836408	-1.301429215
Hypertension	rs11604462	-1.352686756	0.984811038	0.169581569	-3.282916389	0.577542878
Hypertension	rs11801879	-1.103437394	0.958679479	0.249733388	-2.982449173	0.775574385
Hypertension	rs12258967	-2.019119897	0.899371469	0.024765923	-3.781887976	-0.256351819
Hypertension	rs12263737	-1.18463084	1.053916333	0.2610014	-3.250306853	0.881045172
Hypertension	rs12360772	-2.232637123	0.923273194	0.015598528	-4.042252584	-0.423021662
Hypertension	rs12656497	-0.947237199	0.782391329	0.226012354	-2.480724203	0.586249805
Hypertension	rs1275985	-1.527661422	0.686091704	0.02597308	-2.872401161	-0.182921683
Hypertension	rs12762222	-1.913498966	1.121043618	0.087842363	-4.110744456	0.283746525
Hypertension	rs12932686	-1.633905543	1.079810809	0.130243956	-3.750334729	0.482523642
Hypertension	rs13125101	-1.481570275	0.476783822	0.001887245	-2.416066566	-0.547073983
Hypertension	rs1327235	-1.030679422	0.963319856	0.284653338	-2.918786339	0.857427496
Hypertension	rs162395	-1.746812363	1.077019095	0.104825737	-3.857769789	0.364145063
Hypertension	rs167479	-0.654054286	0.711580974	0.358013714	-2.048752995	0.740644423
Hypertension	rs16948048	1.180768443	1.000665822	0.238007062	-0.780536569	3.142073455
Hypertension	rs17035646	-2.163338061	0.7223164	0.002744443	-3.579078205	-0.747597918
Hypertension	rs17558745	-2.263957751	1.121232609	0.043469692	-4.461573665	-0.066341837

Hypertension	rs1918898	-0.138845065	1.092126062	0.898835276	-2.279412146	2.001722016
Hypertension	rs2003476	-0.215606858	1.058634303	0.838615219	-2.290530092	1.859316375
Hypertension	rs2643826	-1.819090543	0.842769473	0.0308915	-3.47091871	-0.167262376
Hypertension	rs2728624	-0.308732704	1.078201268	0.774617332	-2.42200719	1.804541782
Hypertension	rs2759315	-0.890460826	0.883767896	0.313659412	-2.622645903	0.841724251
Hypertension	rs3184504	-3.322696607	0.679800069	1.02E-06	-4.655104742	-1.990288472
Hypertension	rs346078	-1.632207299	1.10481653	0.139580242	-3.797647697	0.5332331
Hypertension	rs35443	-2.132293112	0.859998102	0.01315977	-3.817889392	-0.446696831
Hypertension	rs35587371	-1.90091684	0.935908524	0.042245606	-3.735297547	-0.066536133
Hypertension	rs3735533	-0.63633216	0.906714017	0.482803718	-2.413491633	1.140827313
Hypertension	rs3790604	-2.105848454	0.880834333	0.016814321	-3.832283745	-0.379413162
Hypertension	rs3796581	-0.91535047	0.928987174	0.324466474	-2.736165332	0.905464391
Hypertension	rs3821843	-1.746333534	0.969863073	0.071766261	-3.647265157	0.154598088
Hypertension	rs3918226	-1.693870317	0.757074492	0.025260912	-3.17773632	-0.210004313
Hypertension	rs4291	-2.464524192	1.096528681	0.024603667	-4.613720407	-0.315327977
Hypertension	rs4412193	-4.38690678	0.910222458	1.44E-06	-6.170942797	-2.602870763
Hypertension	rs55670730	-2.684525297	1.111941293	0.015766821	-4.863930231	-0.505120363
Hypertension	rs55730499	-7.837112026	0.97975416	1.25E-15	-9.76E+00	-5.916793872
Hypertension	rs56094641	-2.260759242	1.04507287	0.030521563	-4.309102068	-0.212416416
Hypertension	rs56273825	-1.044587547	1.093455795	0.339421742	-3.187760904	1.098585811
Hypertension	rs568546	-0.848803209	0.794972482	0.285649564	-2.406949273	0.709342856
Hypertension	rs57139556	-0.409803589	1.035166061	0.692192096	-2.438729069	1.61912189
Hypertension	rs6026744	-1.496513155	0.769585755	0.051826787	-3.004901235	0.011874926
Hypertension	rs6031435	-1.974881331	0.996182058	0.047428875	-3.927398164	-0.022364498
Hypertension	rs6108171	0.00847602	0.642129595	0.989468342	-1.250097986	1.267050026
Hypertension	rs62089932	0.790440815	1.099006213	0.471997774	-1.363611363	2.944492993
Hypertension	rs633185	0.021099207	0.704612321	0.97611138	-1.359940943	1.402139358
Hypertension	rs6766859	-0.738716758	1.064491224	0.487705864	-2.825119557	1.347686041
Hypertension	rs68096471	-0.491612854	1.045955672	0.638345668	-2.54168597	1.558460262
Hypertension	rs6822044	-0.276795406	1.035830921	0.789299282	-2.30702401	1.753433199
Hypertension	rs6866614	-1.014085225	1.075511396	0.345738937	-3.122087561	1.093917111
Hypertension	rs6918911	0.78885777	0.963132953	0.41275568	-1.098882817	2.676598358
Hypertension	rs6961048	-1.182718647	1.072901621	0.270307294	-3.285605825	0.92016853
Hypertension	rs6991641	-1.410797149	0.911626089	0.121727983	-3.197584283	0.375989984
Hypertension	rs72831345	-0.075265453	0.609873278	0.901781152	-1.270617077	1.120086172
Hypertension	rs7297416	-1.347967172	1.128549318	0.232311883	-3.559923836	0.863989493
Hypertension	rs740746	-3.282453418	0.840581242	9.42E-05	-4.929992653	-1.634914183
Hypertension	rs7497304	-1.036913639	0.75520788	0.169746905	-2.517121084	0.443293806

Hypertension	rs7528118	-2.132830735	1.110341709	0.054747116	-4.309100485	0.043439014
Hypertension	rs76452347	-2.161517045	1.036645056	0.037059485	-4.193341355	-0.129692736
Hypertension	rs7685862	-0.968383063	1.079258467	0.369576433	-3.083729657	1.146963532
Hypertension	rs7700842	-1.15256154	0.617191607	0.061841695	-2.36225709	0.05713401
Hypertension	rs7763350	-0.891481004	0.985521294	0.365688929	-2.823102739	1.040140732
Hypertension	rs77924615	-1.668414274	1.016824674	0.100837233	-3.661390636	0.324562088
Hypertension	rs8042127	0.363017194	1.062203668	0.732532599	-1.718901994	2.444936383
Hypertension	rs8118848	1.920159823	0.993739909	0.053327926	-0.027570398	3.867890045
Hypertension	rs9330353	-1.771020905	0.913769184	0.052605037	-3.562008506	0.019966696
Hypertension	rs9375459	-2.560980521	0.669926371	0.000131956	-3.874036207	-1.247924834
Hypertension	All - Inverse variance weighted	-1.300674081	0.161474622	7.95E-16	-1.61716434	-0.984183823
Hypertension	All - MR Egger	-1.741783869	0.606940315	0.00549075	-2.931386886	-0.552180852
HDL cholesterol	rs10031010	0.060915247	0.386628332	0.874807425	-0.696876285	0.818706778
HDL cholesterol	rs10053349	-0.108193486	0.352742128	0.759055599	-0.799568056	0.583181085
HDL cholesterol	rs10108282	-0.178785937	0.302719478	0.554788878	-0.772116113	0.41454424
HDL cholesterol	rs10162642	0.580758853	0.106489254	4.93E-08	0.372039915	0.789477792
HDL cholesterol	rs10233430	0.361035698	0.203987919	0.076745859	-0.038780623	0.760852018
HDL cholesterol	rs1045241	-0.116777628	0.284742129	0.681720018	-0.6748722	0.441316944
HDL cholesterol	rs1047891	-0.558429813	0.235393208	0.017676408	-1.0198005	-0.097059125
HDL cholesterol	rs10504477	0.318662428	0.279251432	0.25381547	-0.228670379	0.865995234
HDL cholesterol	rs10513801	0.009326465	0.198164015	0.962461923	-0.379075005	0.397727935
HDL cholesterol	rs1055582	-0.176450079	0.294864921	0.549566482	-0.754385323	0.401485166
HDL cholesterol	rs10750766	0.316846114	0.245279889	0.19643552	-0.163902468	0.797594696
HDL cholesterol	rs10774439	0.07737191	0.262986399	0.768601269	-0.438081431	0.592825252
HDL cholesterol	rs10786114	0.14665424	0.262533772	0.576427264	-0.367911953	0.661220433
HDL cholesterol	rs1083470	-0.08179601	0.368306728	0.824246425	-0.803677197	0.640085177
HDL cholesterol	rs11009262	-0.490348881	0.380565307	0.197580669	-1.236256883	0.255559122
HDL cholesterol	rs11021232	0.057239123	0.322133733	0.858968437	-0.574142994	0.688621241
HDL cholesterol	rs11045171	-0.351316889	0.18405209	0.056289118	-0.712058986	0.009425208
HDL cholesterol	rs111363680	-1.23001423	0.388577906	0.001548566	-1.991626926	-0.468401535
HDL cholesterol	rs11171710	-0.325400631	0.364823792	0.372425537	-1.040455264	0.389654001
HDL cholesterol	rs112001035	-0.701589521	0.190645587	0.000233168	-1.075254872	-0.32792417
HDL cholesterol	rs11218738	0.523701695	0.203583399	0.010099074	0.124678232	0.922725158
HDL cholesterol	rs112233856	-0.098092337	0.240848423	0.683803597	-0.570155245	0.373970572
HDL cholesterol	rs112350227	1.346412883	0.398933555	0.000738075	0.564503114	2.128322651
HDL cholesterol	rs11239536	0.221340448	0.168403588	0.188730063	-0.108730585	0.55141148
HDL cholesterol	rs11254464	0.822567624	0.328537165	0.012289255	0.178634781	1.466500466
HDL cholesterol	rs1132274	-0.261271573	0.262025444	0.318704855	-0.774841444	0.252298298

HDL cholesterol	rs113740515	0.000937067	0.134507825	0.994441473	-0.26269827	0.264572405
HDL cholesterol	rs11381821	-0.004560994	0.225277825	0.983847066	-0.446105531	0.436983543
HDL cholesterol	rs113851927	0.174098777	0.396226598	0.660377559	-0.602505355	0.95070291
HDL cholesterol	rs113966472	-0.042399059	0.374333757	0.909820155	-0.776093223	0.691295105
HDL cholesterol	rs114165349	-0.915355973	0.171054839	8.74E-08	-1.250623458	-0.580088487
HDL cholesterol	rs11429307	-0.785859911	0.171000416	4.31E-06	-1.121020725	-0.450699096
HDL cholesterol	rs11456863	0.834034387	0.228325359	0.000259363	0.386516683	1.28155209
HDL cholesterol	rs115912456	-0.807936904	0.371770403	0.029764162	-1.536606894	-0.079266914
HDL cholesterol	rs116006942	-0.229370606	0.293557415	0.434597633	-0.80474314	0.346001928
HDL cholesterol	rs11614202	0.662850392	0.249082381	0.007786979	0.174648925	1.15105186
HDL cholesterol	rs11631178	0.489671943	0.319740166	0.125653684	-0.137018783	1.116362669
HDL cholesterol	rs11640494	0.124056942	0.27133038	0.64751487	-0.407750603	0.655864486
HDL cholesterol	rs11664369	-0.204979175	0.204529174	0.31624692	-0.605856356	0.195898005
HDL cholesterol	rs1168114	2.680713159	0.279171628	7.81E-22	2.133536768	3.22788955
HDL cholesterol	rs116843064	-0.33938684	0.071777784	2.26E-06	-0.480071298	-0.198702383
HDL cholesterol	rs116857878	-0.399000882	0.319088036	0.211138358	-1.024413433	0.226411668
HDL cholesterol	rs11688682	0.269832869	0.325972219	0.407795825	-0.369072682	0.908738419
HDL cholesterol	rs11704977	0.271673243	0.395255105	0.491870731	-0.503026763	1.046373249
HDL cholesterol	rs117230571	0.476951546	0.296198441	0.107344759	-0.103597398	1.05750049
HDL cholesterol	rs117291242	-0.777159462	0.348065284	0.02556227	-1.459367419	-0.094951505
HDL cholesterol	rs117762989	0.134922636	0.377390554	0.720706593	-0.60476285	0.874608122
HDL cholesterol	rs117847213	0.004531968	0.356197287	0.989848631	-0.693614715	0.702678652
HDL cholesterol	rs12045101	0.523552251	0.331952529	0.114751784	-0.127074705	1.174179207
HDL cholesterol	rs12046972	-0.472260868	0.281227018	0.093096128	-1.023465823	0.078944087
HDL cholesterol	rs12205778	-0.676973405	0.291022111	0.020008292	-1.247376741	-0.106570068
HDL cholesterol	rs12229011	-0.565241812	0.26874156	0.035440305	-1.091975271	-0.038508354
HDL cholesterol	rs1225053	0.013628372	0.31359592	0.965336139	-0.601019631	0.628276374
HDL cholesterol	rs1240820	-0.196011681	0.345713961	0.570730168	-0.873611044	0.481587682
HDL cholesterol	rs12411732	-0.151622904	0.19471154	0.436153346	-0.533257523	0.230011714
HDL cholesterol	rs12462109	0.218206833	0.285995901	0.445480159	-0.342345132	0.778758799
HDL cholesterol	rs12475332	-0.231168489	0.363824921	0.52517808	-0.944265333	0.481928355
HDL cholesterol	rs12575456	0.045397496	0.099061377	0.646753842	-0.148762803	0.239557795
HDL cholesterol	rs12686780	-0.313969497	0.336938425	0.351424136	-0.974368809	0.346429815
HDL cholesterol	rs1270076	0.301398397	0.346467759	0.384345444	-0.377678411	0.980475204
HDL cholesterol	rs12705595	0.173584854	0.389220652	0.65561127	-0.589287624	0.936457333
HDL cholesterol	rs12740374	-3.909482415	0.172286569	5.41E-114	-4.24716409	-3.571800741
HDL cholesterol	rs12740811	0.105873671	0.386670971	0.784232044	-0.652001432	0.863748775
HDL cholesterol	rs12781812	0.15447702	0.393424624	0.69458018	-0.616635243	0.925589284

HDL cholesterol	rs12921195	-0.161215085	0.370398936	0.663383034	-0.887196999	0.564766829
HDL cholesterol	rs12926854	0.060342978	0.382756396	0.874729654	-0.689859559	0.810545514
HDL cholesterol	rs12928099	0.279033711	0.212093549	0.188302889	-0.136669644	0.694737066
HDL cholesterol	rs12986742	0.645971774	0.391652988	0.099076517	-0.121668082	1.41361163
HDL cholesterol	rs12998038	-0.464000364	0.358822147	0.1959693	-1.167291772	0.239291044
HDL cholesterol	rs13066793	-0.15979055	0.327208077	0.625305229	-0.801118382	0.481537281
HDL cholesterol	rs13087167	-0.013948035	0.257206583	0.956752781	-0.518072938	0.490176868
HDL cholesterol	rs13097947	-0.453894534	0.276374707	0.100524585	-0.99558896	0.087799892
HDL cholesterol	rs13107325	-0.083230901	0.098092127	0.396161094	-0.275491469	0.109029667
HDL cholesterol	rs13111599	-0.317005808	0.367556539	0.388429952	-1.037416624	0.403405008
HDL cholesterol	rs13137144	-0.501538949	0.255055981	0.049253841	-1.001448672	-0.001629226
HDL cholesterol	rs13144151	0.166641484	0.328093679	0.611517694	-0.476422127	0.809705095
HDL cholesterol	rs13235365	0.415026667	0.179897049	0.021053526	0.062428451	0.767624883
HDL cholesterol	rs13269725	-0.343848687	0.296282588	0.245827792	-0.92456256	0.236865185
HDL cholesterol	rs13379043	-1.104787587	0.235806786	2.80E-06	-1.566968888	-0.642606287
HDL cholesterol	rs13389219	-0.816492622	0.152637348	8.83E-08	-1.115661825	-0.51732342
HDL cholesterol	rs13402475	-0.12530063	0.218031353	0.565500378	-0.552642081	0.302040822
HDL cholesterol	rs1349852	0.189272878	0.37317506	0.612017339	-0.54215024	0.920695997
HDL cholesterol	rs138354839	0.524407791	0.304664733	0.085203313	-0.072735085	1.121550667
HDL cholesterol	rs1383732	-0.143340525	0.382979003	0.708197673	-0.893979372	0.607298321
HDL cholesterol	rs1395221	-0.138031782	0.379250679	0.715888461	-0.881363113	0.60529955
HDL cholesterol	rs140064750	-0.826480039	0.313799176	0.008443856	-1.441526424	-0.211433655
HDL cholesterol	rs140164052	0.30772566	0.283603184	0.277896348	-0.248136581	0.863587901
HDL cholesterol	rs140584594	-0.478413955	0.148952721	0.001318827	-0.770361289	-0.186466622
HDL cholesterol	rs141062196	-0.045486097	0.27720853	0.869663279	-0.588814816	0.497842622
HDL cholesterol	rs1411432	-0.982820181	0.380487561	0.00979298	-1.728575799	-0.237064562
HDL cholesterol	rs1412234	0.646827926	0.368171657	0.07894081	-0.074788523	1.368444374
HDL cholesterol	rs141440048	-0.585013459	0.376153656	0.119886233	-1.322274625	0.152247706
HDL cholesterol	rs141469619	-0.517950255	0.107329012	1.39E-06	-0.728315119	-0.307585392
HDL cholesterol	rs142288236	-0.829738381	0.222397547	0.000190813	-1.265637574	-0.393839188
HDL cholesterol	rs1431659	-0.218210885	0.365557623	0.550556723	-0.934703826	0.498282056
HDL cholesterol	rs144033177	-0.099349829	0.308227461	0.747205481	-0.703475652	0.504775994
HDL cholesterol	rs144311893	-4.966681694	0.17752512	3.07E-172	-5.31463093	-4.618732459
HDL cholesterol	rs1446585	0.360840628	0.280963135	0.199037413	-0.189847116	0.911528371
HDL cholesterol	rs145947882	-0.180409858	0.079787189	0.023750702	-0.336792749	-0.024026968
HDL cholesterol	rs1471251	-1.002793527	0.219054848	4.70E-06	-1.432141029	-0.573446025
HDL cholesterol	rs147460434	0.185838475	0.357613048	0.60329808	-0.5150831	0.88676005
HDL cholesterol	rs147627829	-0.159226001	0.188919354	0.399326187	-0.529507935	0.211055933

HDL cholesterol	rs147772065	-0.238982898	0.393599367	0.543736126	-1.010437657	0.532471861
HDL cholesterol	rs150224153	0.060381047	0.134671532	0.653894915	-0.203575157	0.32433725
HDL cholesterol	rs150237291	0.143234641	0.31365962	0.647918107	-0.471538214	0.758007496
HDL cholesterol	rs150844304	-0.302515932	0.144433032	0.03621488	-0.585604674	-0.019427189
HDL cholesterol	rs150861794	0.388001199	0.373047443	0.298300226	-0.343171789	1.119174188
HDL cholesterol	rs1534696	-0.288654224	0.248304347	0.245031738	-0.775330744	0.198022297
HDL cholesterol	rs1601934	0.385794892	0.044469115	4.11E-18	0.298635427	0.472954358
HDL cholesterol	rs16928809	0.349014785	0.272967601	0.201040002	-0.186001713	0.884031283
HDL cholesterol	rs17124112	-0.363851813	0.368111838	0.322943392	-1.085351015	0.357647389
HDL cholesterol	rs17138358	-0.035728316	0.155054872	0.817762554	-0.339635864	0.268179233
HDL cholesterol	rs17309930	0.108909245	0.232957032	0.640136567	-0.347686539	0.565505028
HDL cholesterol	rs17326656	-0.405169188	0.217987433	0.063072311	-0.832424557	0.022086181
HDL cholesterol	rs174566	0.578774938	0.077006657	5.65E-14	0.42784189	0.729707986
HDL cholesterol	rs1760940	0.228517707	0.395084202	0.562992378	-0.545847329	1.002882742
HDL cholesterol	rs17713879	0.283157258	0.308508449	0.358709658	-0.321519301	0.887833818
HDL cholesterol	rs1771582	0.520714826	0.331536869	0.116273473	-0.129097437	1.17052709
HDL cholesterol	rs183906992	-0.154164644	0.35104689	0.660547894	-0.842216549	0.533887261
HDL cholesterol	rs185073199	-0.184536964	0.341054134	0.58845367	-0.853003066	0.483929139
HDL cholesterol	rs1862205	0.782655099	0.372319015	0.035543741	0.05290983	1.512400368
HDL cholesterol	rs188502504	-0.183587279	0.34822796	0.598051721	-0.866114081	0.498939524
HDL cholesterol	rs1955512	-0.508269234	0.387750259	0.189919944	-1.268259742	0.251721274
HDL cholesterol	rs1970811	0.128486289	0.357696879	0.719442193	-0.572599593	0.829572172
HDL cholesterol	rs201441	0.124357174	0.382682909	0.745210285	-0.625701328	0.874415676
HDL cholesterol	rs201639483	-0.023089637	0.393135468	0.953165561	-0.793635156	0.747455881
HDL cholesterol	rs2066714	0.401720255	0.132012523	0.002341961	0.142975711	0.6604648
HDL cholesterol	rs2068888	-0.85970501	0.216547186	7.19E-05	-1.284137495	-0.435272524
HDL cholesterol	rs2098368	-0.431954942	0.360764981	0.231177732	-1.139054305	0.275144421
HDL cholesterol	rs2098918	0.304053143	0.349879526	0.384834882	-0.381710728	0.989817015
HDL cholesterol	rs2111216	0.066455224	0.197877138	0.736990891	-0.321383967	0.454294416
HDL cholesterol	rs2155220	-0.258406524	0.395092392	0.513085552	-1.032787613	0.515974564
HDL cholesterol	rs2159607	-0.504539494	0.220486607	0.022120059	-0.936693244	-0.072385744
HDL cholesterol	rs2196808	0.269767098	0.365489202	0.460454485	-0.446591738	0.986125935
HDL cholesterol	rs2236464	0.234558125	0.325875921	0.471662058	-0.40415868	0.87327493
HDL cholesterol	rs2237035	0.098538499	0.305930791	0.747381417	-0.501085851	0.698162849
HDL cholesterol	rs2247355	-0.292373556	0.259639174	0.260133167	-0.801266336	0.216519225
HDL cholesterol	rs2256609	0.10216066	0.1601108	0.523433148	-0.211656508	0.415977828
HDL cholesterol	rs2268840	0.188272314	0.284267049	0.50777391	-0.368891101	0.745435729
HDL cholesterol	rs2271308	0.131051835	0.167516355	0.434024993	-0.197280222	0.459383891

HDL cholesterol	rs2281718	-0.450695889	0.071119375	2.34E-10	-0.590089865	-0.311301914
HDL cholesterol	rs2290866	-1.021715062	0.398928925	0.010432762	-1.803615755	-0.239814369
HDL cholesterol	rs2297409	0.216551797	0.156210034	0.165659564	-0.08961987	0.522723463
HDL cholesterol	rs2298214	-0.136699436	0.339883965	0.687540944	-0.802872006	0.529473135
HDL cholesterol	rs2298624	0.092909865	0.204089745	0.648936412	-0.307106036	0.492925766
HDL cholesterol	rs2298632	0.180974267	0.291900979	0.535267575	-0.391151651	0.753100186
HDL cholesterol	rs2302263	-0.116741087	0.200669369	0.560729443	-0.51005305	0.276570877
HDL cholesterol	rs2307111	1.458319521	0.222491621	5.58E-11	1.022235945	1.894403098
HDL cholesterol	rs2339234	0.356490974	0.374290732	0.340871837	-0.377118861	1.09010081
HDL cholesterol	rs235314	-0.183931239	0.233401719	0.430669931	-0.641398608	0.273536131
HDL cholesterol	rs2362541	0.208243609	0.379445273	0.583135397	-0.535469126	0.951956343
HDL cholesterol	rs2364723	-0.418372797	0.368207447	0.255855263	-1.140059393	0.3033138
HDL cholesterol	rs2417125	0.09521	0.350057784	0.785634578	-0.590903257	0.781323257
HDL cholesterol	rs2435307	-0.229166846	0.254903021	0.368633792	-0.728776767	0.270443075
HDL cholesterol	rs2498786	-0.133780192	0.167164689	0.423542922	-0.461422983	0.193862598
HDL cholesterol	rs2516331	-0.220134821	0.336702014	0.513242496	-0.880070769	0.439801127
HDL cholesterol	rs2520096	-0.096324174	0.317610136	0.761677994	-0.718840039	0.526191692
HDL cholesterol	rs254562	-0.298291969	0.36608948	0.415183266	-1.015827349	0.419243412
HDL cholesterol	rs2586116	0.601174213	0.290043487	0.038199862	0.032688978	1.169659448
HDL cholesterol	rs2642438	0.314883824	0.163686985	0.054392834	-0.005942667	0.635710315
HDL cholesterol	rs2645979	-0.342090986	0.377306814	0.364583832	-1.081612342	0.397430369
HDL cholesterol	rs267738	-0.584037589	0.232507959	0.012008187	-1.03975319	-0.128321989
HDL cholesterol	rs2723065	-0.209541314	0.284727615	0.461769623	-0.76760744	0.348524812
HDL cholesterol	rs2726111	-0.83712625	0.2970262	0.004826992	-1.419297603	-0.254954897
HDL cholesterol	rs2740488	0.417377625	0.068366605	1.03E-09	0.283379078	0.551376171
HDL cholesterol	rs2750411	0.176784281	0.382705595	0.644129485	-0.573318685	0.926887247
HDL cholesterol	rs2792751	0.175657483	0.128163037	0.170506438	-0.075542069	0.426857035
HDL cholesterol	rs2800710	-0.970376072	0.204589637	2.11E-06	-1.37137176	-0.569380384
HDL cholesterol	rs2804894	-0.347850205	0.273146195	0.202842587	-0.883216748	0.187516338
HDL cholesterol	rs2814982	0.574013003	0.24671158	0.019983576	0.090458306	1.0575677
HDL cholesterol	rs28362901	-0.009694296	0.307066821	0.974814458	-0.611545265	0.592156674
HDL cholesterol	rs28510484	-0.435533301	0.3570181	0.222495437	-1.135288777	0.264222176
HDL cholesterol	rs286965	-0.854772217	0.318971828	0.007367202	-1.479957	-0.229587434
HDL cholesterol	rs28746806	0.935170605	0.266073535	0.000440239	0.413666477	1.456674733
HDL cholesterol	rs2910949	0.385532404	0.323128302	0.232820485	-0.247799069	1.018863877
HDL cholesterol	rs2925979	-0.226132604	0.120739966	0.061083658	-0.462782938	0.01051773
HDL cholesterol	rs2943645	-0.137691911	0.099175075	0.165023753	-0.332075058	0.056691235
HDL cholesterol	rs2963468	-0.427339527	0.250064835	0.087466711	-0.917466604	0.06278755

HDL cholesterol	rs2965169	-5.305112099	0.350319868	8.35E-52	-5.99173904	-4.618485157
HDL cholesterol	rs3027167	-0.012592831	0.357179417	0.971875355	-0.712664488	0.687478826
HDL cholesterol	rs308	-0.579954154	0.116370247	6.24E-07	-0.808039839	-0.351868469
HDL cholesterol	rs3184504	0.762742712	0.156051728	1.02E-06	0.456881324	1.068604099
HDL cholesterol	rs32578	-0.001017848	0.336676549	0.997587821	-0.660903884	0.658868188
HDL cholesterol	rs330089	0.816016259	0.338372286	0.015882998	0.152806578	1.479225939
HDL cholesterol	rs34045894	-1.034029461	0.328790867	0.001661176	-1.67845956	-0.389599362
HDL cholesterol	rs34073570	-0.597846264	0.381364841	0.116963038	-1.345321353	0.149628824
HDL cholesterol	rs34138141	-0.791681402	0.262638748	0.002575425	-1.306453348	-0.276909456
HDL cholesterol	rs343	-0.358153807	0.056420189	2.18E-10	-0.468737377	-0.247570237
HDL cholesterol	rs34518086	-0.439451424	0.137322168	0.001373565	-0.708602873	-0.170299975
HDL cholesterol	rs34940374	-0.642982075	0.314461961	0.040883909	-1.259327519	-0.026636631
HDL cholesterol	rs35493868	-0.811554765	0.138306354	4.42E-09	-1.082635219	-0.540474311
HDL cholesterol	rs35980001	0.364989435	0.042841641	1.60E-17	0.281019819	0.448959051
HDL cholesterol	rs36057735	0.491636217	0.171273532	0.004098569	0.155940093	0.82733234
HDL cholesterol	rs367070	0.032149138	0.118603466	0.786341758	-0.200313655	0.264611931
HDL cholesterol	rs367677	-0.529167441	0.292962334	0.070877046	-1.103373617	0.045038734
HDL cholesterol	rs3732356	0.454743066	0.279368349	0.103576968	-0.092818899	1.00230503
HDL cholesterol	rs3745683	-0.170618812	0.143299112	0.23379177	-0.451485073	0.110247448
HDL cholesterol	rs3746915	-0.484977898	0.38587485	0.208816317	-1.241292603	0.271336807
HDL cholesterol	rs3747973	-0.405301945	0.297597148	0.173224792	-0.988592355	0.177988466
HDL cholesterol	rs3768321	0.003105732	0.115185852	0.978489414	-0.222658538	0.228870001
HDL cholesterol	rs3794752	-0.509115066	0.360998551	0.158452091	-1.216672225	0.198442093
HDL cholesterol	rs3814883	0.075175613	0.271282386	0.781694083	-0.456537864	0.606889091
HDL cholesterol	rs3924313	-0.319359195	0.185953807	0.085904022	-0.683828656	0.045110266
HDL cholesterol	rs4074448	-0.171818783	0.282216641	0.542644584	-0.7249634	0.381325834
HDL cholesterol	rs41272086	-0.076692448	0.118299328	0.516796432	-0.30855913	0.155174234
HDL cholesterol	rs42125	-1.583180951	0.382166068	3.43E-05	-2.332226445	-0.834135456
HDL cholesterol	rs429358	-2.291799396	0.075512786	2.53E-202	-2.439804455	-2.143794336
HDL cholesterol	rs4330777	-0.526474471	0.210122369	0.012225721	-0.938314314	-0.114634629
HDL cholesterol	rs454968	-0.622381431	0.391696348	0.112074265	-1.390106274	0.145343412
HDL cholesterol	rs4599108	-0.405227802	0.306246433	0.185766138	-1.005470809	0.195015206
HDL cholesterol	rs460428	-0.062601595	0.343277662	0.855296831	-0.735425813	0.610222623
HDL cholesterol	rs4614	0.111918055	0.236049311	0.635407221	-0.350738594	0.574574704
HDL cholesterol	rs4650994	-0.123053833	0.228680771	0.59050587	-0.571268144	0.325160479
HDL cholesterol	rs4691379	0.550312087	0.373380392	0.140518091	-0.181513482	1.282137655
HDL cholesterol	rs4784709	-0.077549636	0.141489259	0.583626284	-0.354868584	0.199769312
HDL cholesterol	rs4803773	-0.783038873	0.107626869	3.45E-13	-0.993987536	-0.572090209

HDL cholesterol	rs4804101	-0.01317271	0.299853282	0.964959788	-0.600885143	0.574539722
HDL cholesterol	rs4855582	0.562621359	0.37494447	0.133473073	-0.172269803	1.297512521
HDL cholesterol	rs4871603	-1.078142315	0.120829526	4.54E-19	-1.314968186	-0.841316444
HDL cholesterol	rs4871624	-0.679898119	0.225183679	0.002533557	-1.121258131	-0.238538107
HDL cholesterol	rs4875043	-0.260930916	0.338094093	0.440250597	-0.923595339	0.401733507
HDL cholesterol	rs4899251	-0.488265359	0.384730745	0.204402091	-1.24233762	0.265806902
HDL cholesterol	rs4917675	-0.508381404	0.326473699	0.119425509	-1.148269853	0.131507046
HDL cholesterol	rs4930352	-0.429117118	0.260652778	0.099698496	-0.939996562	0.081762326
HDL cholesterol	rs4969141	0.020148158	0.139919291	0.885501711	-0.254093652	0.294389968
HDL cholesterol	rs532436	2.08977443	0.230475886	1.22E-19	1.638041693	2.541507167
HDL cholesterol	rs549058	-0.427270814	0.370997984	0.249452752	-1.154426862	0.299885233
HDL cholesterol	rs554146	-0.437301681	0.360257589	0.224801822	-1.143406555	0.268803194
HDL cholesterol	rs55781197	-0.186429193	0.110379008	0.091221082	-0.402772049	0.029913663
HDL cholesterol	rs557933	2.032274778	0.27158757	7.27E-14	1.499963141	2.564586416
HDL cholesterol	rs55935382	0.373734694	0.250810658	0.136196128	-0.117854195	0.865323583
HDL cholesterol	rs559355	-0.148803215	0.162736358	0.360516096	-0.467766477	0.170160046
HDL cholesterol	rs56017932	-0.228802093	0.375762099	0.542589751	-0.965295807	0.50769162
HDL cholesterol	rs564832	-0.249841988	0.381824394	0.512894443	-0.9982178	0.498533823
HDL cholesterol	rs57512892	0.498271358	0.232968991	0.032452816	0.041652135	0.954890581
HDL cholesterol	rs57760538	-0.054154395	0.39490802	0.890926723	-0.828174115	0.719865325
HDL cholesterol	rs58123204	-0.709157303	0.312507179	0.023253052	-1.321671373	-0.096643233
HDL cholesterol	rs58298943	0.119221629	0.368680998	0.746412469	-0.603393126	0.841836385
HDL cholesterol	rs59037995	0.019638618	0.322702091	0.951473251	-0.61285748	0.652134715
HDL cholesterol	rs59104589	-0.523461756	0.286958143	0.06812568	-1.085899717	0.038976205
HDL cholesterol	rs59781045	-0.200484893	0.111214798	0.071438201	-0.418465897	0.017496111
HDL cholesterol	rs6018652	-0.303594694	0.197253453	0.123777528	-0.690211462	0.083022073
HDL cholesterol	rs6059958	0.186121973	0.377729782	0.622197433	-0.5542284	0.926472346
HDL cholesterol	rs6066148	-0.165312791	0.355126048	0.641570179	-0.861359845	0.530734263
HDL cholesterol	rs6073958	-0.127941126	0.085139808	0.132911768	-0.29481515	0.038932898
HDL cholesterol	rs6075860	-0.036250781	0.325904688	0.911432982	-0.675023969	0.602522406
HDL cholesterol	rs6123685	-0.172021429	0.297469067	0.563072788	-0.755060801	0.411017943
HDL cholesterol	rs61352607	-0.584186782	0.157682017	0.000211523	-0.893243536	-0.275130028
HDL cholesterol	rs6142206	-0.168667213	0.26027336	0.516959913	-0.678802999	0.341468573
HDL cholesterol	rs61435086	-0.528521541	0.208094646	0.011091167	-0.936387048	-0.120656033
HDL cholesterol	rs61596977	-0.181340524	0.367889676	0.622068453	-0.90240429	0.539723242
HDL cholesterol	rs61748951	0.163100907	0.381731653	0.669185689	-0.585093133	0.911294947
HDL cholesterol	rs61805075	-0.529587881	0.17076673	0.001927148	-0.864290672	-0.19488509
HDL cholesterol	rs61884005	-0.983556295	0.390059117	0.011683674	-1.748072164	-0.219040427

HDL cholesterol	rs62102718	-0.141139382	0.193414728	0.465558866	-0.520232249	0.237953484
HDL cholesterol	rs62117487	-0.561915421	0.194753731	0.003910888	-0.943632733	-0.180198108
HDL cholesterol	rs62246443	-0.519091456	0.368995745	0.159496155	-1.242323117	0.204140205
HDL cholesterol	rs62271373	-0.475841413	0.218245395	0.029234899	-0.903602387	-0.048080439
HDL cholesterol	rs62331150	-0.774835562	0.395296915	0.049979941	-1.549617515	-5.36084E-05
HDL cholesterol	rs62428831	0.271783487	0.335107858	0.417346796	-0.385027915	0.928594889
HDL cholesterol	rs635769	-0.957730541	0.216969691	1.01E-05	-1.382991135	-0.532469947
HDL cholesterol	rs6460894	0.402079686	0.359931116	0.263950815	-0.303385301	1.107544672
HDL cholesterol	rs6469605	-0.304499726	0.132460144	0.021516149	-0.564121608	-0.044877845
HDL cholesterol	rs6693842	0.565582553	0.317248244	0.074622775	-0.056224005	1.18738911
HDL cholesterol	rs67016280	-0.243370667	0.37664643	0.518181226	-0.981597669	0.494856335
HDL cholesterol	rs6705285	-0.288946843	0.362422217	0.425296672	-0.999294389	0.421400703
HDL cholesterol	rs676210	-1.355338505	0.085973538	5.45E-56	-1.523846639	-1.186830371
HDL cholesterol	rs6762415	-0.212055358	0.38055321	0.577370847	-0.95793965	0.533828934
HDL cholesterol	rs6765484	0.231219278	0.18418344	0.209342676	-0.129780264	0.592218821
HDL cholesterol	rs680321	-0.122601989	0.382904061	0.748824427	-0.873093948	0.627889971
HDL cholesterol	rs6824451	-0.10829708	0.207113209	0.601051964	-0.514238969	0.297644809
HDL cholesterol	rs686030	0.065328225	0.119452634	0.584449857	-0.168798939	0.299455388
HDL cholesterol	rs689183	-0.170825279	0.303366435	0.573367602	-0.765423492	0.423772935
HDL cholesterol	rs6934962	0.919292178	0.260326117	0.000413507	0.409052988	1.429531367
HDL cholesterol	rs6939861	-0.1895578	0.335304144	0.571848702	-0.846753923	0.467638323
HDL cholesterol	rs7036107	0.004617153	0.343720414	0.989282441	-0.669074859	0.678309164
HDL cholesterol	rs703966	-0.682253471	0.267571632	0.010778551	-1.206693869	-0.157813073
HDL cholesterol	rs71571682	-0.288916895	0.277089315	0.297094169	-0.832011952	0.254178162
HDL cholesterol	rs7158166	0.015266866	0.299839639	0.959391841	-0.572418826	0.602952559
HDL cholesterol	rs71603401	-0.865183136	0.392659044	0.027566891	-1.634794863	-0.095571409
HDL cholesterol	rs71647892	0.487349415	0.303088128	0.107846954	-0.106703317	1.081402146
HDL cholesterol	rs7170463	-0.275261488	0.23667125	0.244807517	-0.739137137	0.188614161
HDL cholesterol	rs7186799	-0.546674295	0.189737966	0.003961558	-0.918560709	-0.17478788
HDL cholesterol	rs7218647	-0.18556163	0.383083812	0.62810974	-0.936405901	0.565282642
HDL cholesterol	rs7251640	0.669664275	0.370612817	0.0707762	-0.056736847	1.396065397
HDL cholesterol	rs72647336	-0.929206754	0.218657235	2.14E-05	-1.357774935	-0.500638574
HDL cholesterol	rs7281183	-0.676040094	0.372430818	0.069491664	-1.406004497	0.053924308
HDL cholesterol	rs72926946	0.589136591	0.218977562	0.007136705	0.159940571	1.018332612
HDL cholesterol	rs72964564	0.454637847	0.381764322	0.233698279	-0.293620225	1.202895919
HDL cholesterol	rs7305678	-1.041626057	0.368407152	0.004693098	-1.763704075	-0.319548039
HDL cholesterol	rs73151974	-0.125139751	0.366279379	0.73261348	-0.843047333	0.592767831
HDL cholesterol	rs7316878	-0.270149319	0.381192637	0.478513763	-1.017286886	0.476988249

HDL cholesterol	rs73243877	-0.443942087	0.218769536	0.042430969	-0.872730379	-0.015153796
HDL cholesterol	rs73455693	0.156812853	0.396581458	0.692539253	-0.620486805	0.934112512
HDL cholesterol	rs74500135	0.324352449	0.325439279	0.318929367	-0.313508538	0.962213436
HDL cholesterol	rs7488780	-0.090622787	0.353742979	0.797809863	-0.783959026	0.602713452
HDL cholesterol	rs75032664	0.103268359	0.361911364	0.775382462	-0.606077914	0.812614633
HDL cholesterol	rs75152587	-0.339151808	0.200097216	0.09008772	-0.731342351	0.053038735
HDL cholesterol	rs75246752	0.199691129	0.373012803	0.59241022	-0.531413964	0.930796223
HDL cholesterol	rs75479205	0.114909232	0.379472734	0.762032466	-0.628857326	0.85867579
HDL cholesterol	rs75609851	-0.452854682	0.122570537	0.000220194	-0.693092934	-0.21261643
HDL cholesterol	rs75662196	0.044335007	0.18933553	0.814859951	-0.326762631	0.415432645
HDL cholesterol	rs7583067	0.113424143	0.332879284	0.733301911	-0.539019254	0.76586754
HDL cholesterol	rs7622114	-0.820734395	0.364518053	0.024350069	-1.535189779	-0.106279011
HDL cholesterol	rs76247316	0.243966592	0.363719635	0.50237692	-0.468923893	0.956857078
HDL cholesterol	rs76428106	1.001970686	0.305770426	0.001049638	0.402660651	1.601280722
HDL cholesterol	rs76602912	-0.173502334	0.30897679	0.574430557	-0.779096843	0.432092175
HDL cholesterol	rs7665587	-0.318344938	0.299508886	0.287831932	-0.905382354	0.268692478
HDL cholesterol	rs76962725	-0.262368431	0.395862496	0.50747359	-1.038258923	0.51352206
HDL cholesterol	rs771481	-0.148908894	0.185223913	0.421431685	-0.511947763	0.214129975
HDL cholesterol	rs7725218	-0.517110452	0.356167683	0.146536758	-1.215199112	0.180978207
HDL cholesterol	rs77320712	0.205652751	0.38654705	0.59470858	-0.551979467	0.963284969
HDL cholesterol	rs77605964	0.016314585	0.289460433	0.955053385	-0.551027865	0.583657034
HDL cholesterol	rs77767539	-0.227273832	0.39354099	0.563595028	-0.998614172	0.544066508
HDL cholesterol	rs7794796	-0.703939807	0.26244742	0.007313694	-1.21833675	-0.189542864
HDL cholesterol	rs77960347	0.117526446	0.062067733	0.058288853	-0.00412631	0.239179203
HDL cholesterol	rs78058190	-0.049772235	0.136531488	0.715449088	-0.317373951	0.217829481
HDL cholesterol	rs7817574	-0.420720816	0.159872886	0.00849849	-0.734071672	-0.107369959
HDL cholesterol	rs7826177	-0.336617764	0.38407993	0.380798309	-1.089414425	0.416178898
HDL cholesterol	rs7853377	-0.066562149	0.330215091	0.840251258	-0.713783728	0.580659429
HDL cholesterol	rs79153732	-0.427496837	0.168353801	0.011108202	-0.757470288	-0.097523386
HDL cholesterol	rs7924036	0.07199951	0.298059548	0.809120482	-0.512197204	0.656196223
HDL cholesterol	rs79600951	-0.141913947	0.067368978	0.035159404	-0.273957144	-0.009870751
HDL cholesterol	rs79634051	-0.65530616	0.320835501	0.041102068	-1.284143742	-0.026468578
HDL cholesterol	rs80005209	-0.442903805	0.085410931	2.15E-07	-0.61030923	-0.275498381
HDL cholesterol	rs8007841	-0.15897416	0.340385661	0.640470334	-0.826130056	0.508181736
HDL cholesterol	rs8014289	0.601480986	0.27621617	0.029437811	0.060097294	1.142864679
HDL cholesterol	rs8081548	-0.40017019	0.23720542	0.091599586	-0.865092813	0.064752434
HDL cholesterol	rs8086351	0.045646371	0.064512909	0.479222226	-0.080798931	0.172091674
HDL cholesterol	rs830620	0.244459602	0.281022884	0.384359343	-0.30634525	0.795264455

HDL cholesterol	rs880674	-0.589529886	0.396992045	0.137546026	-1.367634293	0.188574521
HDL cholesterol	rs907866	0.856415912	0.227910938	0.000171497	0.409710473	1.303121351
HDL cholesterol	rs921919	-0.093945998	0.107780151	0.383402236	-0.305195094	0.117303099
HDL cholesterol	rs9327468	0.268169188	0.323717734	0.407441528	-0.36631757	0.902655946
HDL cholesterol	rs9347737	0.077656844	0.31301193	0.804060537	-0.53584654	0.691160227
HDL cholesterol	rs9465693	0.330887409	0.372536445	0.374432402	-0.399284024	1.061058841
HDL cholesterol	rs9604045	0.349835285	0.283417737	0.21707435	-0.205663479	0.905334049
HDL cholesterol	rs9622830	-0.573208563	0.263395413	0.029538191	-1.089463573	-0.056953553
HDL cholesterol	rs964184	-0.90659522	0.057783099	1.78E-55	-1.019850095	-0.793340345
HDL cholesterol	rs9647335	-0.78714314	0.189770349	3.36E-05	-1.159093024	-0.415193256
HDL cholesterol	rs968050	-0.176565325	0.30311784	0.560231755	-0.770676291	0.41754564
HDL cholesterol	rs983663	0.096562169	0.334500167	0.772829333	-0.559058158	0.752182497
HDL cholesterol	rs9904004	-0.876376803	0.286055224	0.002186451	-1.437045042	-0.315708564
HDL cholesterol	rs9916613	-0.142157017	0.324315087	0.661147114	-0.777814587	0.493500552
HDL cholesterol	rs9933509	0.638351139	0.299501145	0.033057743	0.051328894	1.225373384
HDL cholesterol	rs998584	-0.397650197	0.121027139	0.001017552	-0.63486339	-0.160437004
HDL cholesterol	rs9987289	0.302351238	0.082408564	0.000243568	0.140830453	0.463872022
HDL cholesterol	rs9989419	-0.216547143	0.029386847	1.72E-13	-0.274145362	-0.158948924
HDL cholesterol	All - Inverse variance weighted	-0.173135252	0.033849857	3.14E-07	-0.239480973	-0.106789532
HDL cholesterol	All - MR Egger	-0.214795286	0.051863054	4.34E-05	-0.316446873	-0.113143699
LDL cholesterol	rs1010759	1.209745414	0.258472918	2.86E-06	0.703138494	1.716352334
LDL cholesterol	rs1016988	0.937629389	0.301883849	0.001896831	0.345937044	1.529321734
LDL cholesterol	rs10231941	0.383647168	0.272353134	0.158942025	-0.150164974	0.917459311
LDL cholesterol	rs10448340	0.630487934	0.294311194	0.032173431	0.053637994	1.207337875
LDL cholesterol	rs10832963	0.873314881	0.275187653	0.001506	0.333947082	1.41268268
LDL cholesterol	rs10910476	0.726249415	0.337442108	0.031380087	0.064862883	1.387635947
LDL cholesterol	rs11014204	1.09687219	0.334861953	0.001054396	0.440542763	1.753201618
LDL cholesterol	rs11065385	0.733679319	0.182997828	6.09E-05	0.375003577	1.092355062
LDL cholesterol	rs11099097	0.775159999	0.250810442	0.001997385	0.283571532	1.266748466
LDL cholesterol	rs111273322	0.827968103	0.307892126	0.007163423	0.224499536	1.431436669
LDL cholesterol	rs111338114	1.209412566	0.342417713	0.00041246	0.538273849	1.880551284
LDL cholesterol	rs11226108	0.323806315	0.327613877	0.322967609	-0.318316885	0.965929515
LDL cholesterol	rs113177823	0.566045801	0.223903123	0.011468706	0.127195681	1.004895921
LDL cholesterol	rs114165349	1.319438368	0.246566718	8.74E-08	0.8361676	1.802709136
LDL cholesterol	rs115458560	0.120420905	0.303297039	0.691338331	-0.474041292	0.714883103
LDL cholesterol	rs11568318	0.472603726	0.32387031	0.14449973	-0.162182081	1.107389533
LDL cholesterol	rs11591147	0.863977087	0.045195663	1.85E-81	0.775393588	0.952560587
LDL cholesterol	rs11601507	1.178325652	0.250080121	2.46E-06	0.688168615	1.66848269

LDL cholesterol	rs11621792	1.190653249	0.217993723	4.71E-08	0.763385552	1.617920946
LDL cholesterol	rs116734477	0.98713897	0.2202726	7.41E-06	0.555404675	1.418873266
LDL cholesterol	rs117139027	0.656405291	0.273740777	0.016488991	0.119873369	1.192937213
LDL cholesterol	rs117733303	-1.851730778	0.182914608	4.35E-24	-2.210243409	-1.493218147
LDL cholesterol	rs118039278	-0.728885248	0.092213816	2.69E-15	-0.909624327	-0.54814617
LDL cholesterol	rs1183851	1.13263322	0.174185093	7.90E-11	0.791230439	1.474036001
LDL cholesterol	rs12078100	0.935429012	0.325681972	0.004076041	0.297092346	1.573765678
LDL cholesterol	rs12162782	1.463315637	0.335616396	1.30E-05	0.8055075	2.121123773
LDL cholesterol	rs12208357	1.203738574	0.142625425	3.18E-17	0.924192741	1.483284406
LDL cholesterol	rs12246352	0.971838265	0.265166029	0.000247321	0.452112849	1.491563682
LDL cholesterol	rs1229984	0.302375251	0.237907405	0.203736217	-0.163923263	0.768673766
LDL cholesterol	rs12445804	0.699575445	0.347535322	0.044118612	0.018406215	1.380744676
LDL cholesterol	rs12471768	0.703391937	0.333199509	0.034770307	0.050320899	1.356462974
LDL cholesterol	rs1250258	0.828102653	0.341796072	0.015401511	0.158182351	1.498022955
LDL cholesterol	rs1260326	1.384481383	0.121613077	5.01E-30	1.146119753	1.622843014
LDL cholesterol	rs12916	0.925029179	0.067925947	3.12E-42	0.791894323	1.058164034
LDL cholesterol	rs13020929	0.834366386	0.285013645	0.003417417	0.275739643	1.39299313
LDL cholesterol	rs13076933	0.904982376	0.225549205	6.01E-05	0.462905935	1.347058817
LDL cholesterol	rs13107325	-0.26928857	0.31737117	0.396161094	-0.891336064	0.352758923
LDL cholesterol	rs13108218	1.357427574	0.240587842	1.68E-08	0.885875404	1.828979744
LDL cholesterol	rs13121616	0.783116873	0.348357677	0.024574576	0.100335826	1.46589792
LDL cholesterol	rs1350559	0.087510158	0.307244747	0.775780281	-0.514689546	0.689709862
LDL cholesterol	rs140584594	1.037320307	0.322966504	0.001318827	0.40430596	1.670334655
LDL cholesterol	rs143020224	0.871229698	0.03764655	1.74E-118	0.797442459	0.945016937
LDL cholesterol	rs145730801	0.926732316	0.284084501	0.001105623	0.369926693	1.483537938
LDL cholesterol	rs146433259	0.707203278	0.357028887	0.047613563	0.007426659	1.406979896
LDL cholesterol	rs146534110	-0.014950802	0.263255078	0.954710827	-0.530930755	0.501029151
LDL cholesterol	rs148150904	0.898954065	0.333005767	0.006944062	0.246262761	1.551645369
LDL cholesterol	rs150474434	0.808605875	0.197698656	4.31E-05	0.421116508	1.196095241
LDL cholesterol	rs1551891	0.93102495	0.04208964	2.03E-108	0.848529256	1.013520644
LDL cholesterol	rs1556562	0.616612965	0.260432381	0.017901417	0.106165499	1.127060431
LDL cholesterol	rs17050272	0.655841282	0.204287028	0.001325544	0.255438708	1.056243856
LDL cholesterol	rs174564	1.02106735	0.135810732	5.55E-14	0.754878314	1.287256385
LDL cholesterol	rs17476364	1.074808935	0.308040921	0.000484512	0.47104873	1.678569139
LDL cholesterol	rs17569873	1.371165214	0.301649571	5.48E-06	0.779932054	1.962398374
LDL cholesterol	rs1801689	0.262314506	0.194237173	0.176860268	-0.118390353	0.643019366
LDL cholesterol	rs183130	1.630831975	0.134072543	4.84E-34	1.36804979	1.89361416
LDL cholesterol	rs1883711	0.993928189	0.118174565	4.08E-17	0.762306043	1.225550336

LDL cholesterol	rs200046586	0.907150342	0.050189529	5.06E-73	0.808778866	1.005521818
LDL cholesterol	rs2043085	2.335438947	0.249280174	7.34E-21	1.846849806	2.824028088
LDL cholesterol	rs2066714	0.889641258	0.292352166	0.002341961	0.316631013	1.462651503
LDL cholesterol	rs2068888	0.858460754	0.216233776	7.19E-05	0.434642552	1.282278955
LDL cholesterol	rs2073547	0.935119185	0.14913811	3.61E-10	0.642808488	1.227429881
LDL cholesterol	rs2160994	0.487153099	0.237538408	0.0402826	0.02157782	0.952728378
LDL cholesterol	rs2238162	1.125339377	0.250530883	7.06E-06	0.634298846	1.616379909
LDL cholesterol	rs2250802	0.379992745	0.254233562	0.135003189	-0.118305036	0.878290526
LDL cholesterol	rs2256814	0.608898033	0.341006303	0.074165388	-0.059474322	1.277270388
LDL cholesterol	rs2287622	0.949700338	0.199680487	1.97E-06	0.558326584	1.341074093
LDL cholesterol	rs2391825	0.82476434	0.355844526	0.020462042	0.127309069	1.522219611
LDL cholesterol	rs2519093	0.872025107	0.095841296	9.15E-20	0.684176167	1.059874047
LDL cholesterol	rs2611867	1.199471106	0.156119893	1.55E-14	0.893476117	1.505466096
LDL cholesterol	rs2618566	0.809677445	0.175161919	3.79E-06	0.466360083	1.152994807
LDL cholesterol	rs2642438	0.343912755	0.17877718	0.054392834	-0.006490518	0.694316028
LDL cholesterol	rs2737265	1.305692428	0.226941991	8.75E-09	0.860886127	1.75049873
LDL cholesterol	rs2738447	0.784415259	0.09956048	3.31E-15	0.589276718	0.9795538
LDL cholesterol	rs2740488	1.135011804	0.185915342	1.03E-09	0.770617733	1.499405875
LDL cholesterol	rs2745353	1.549853107	0.326587273	2.08E-06	0.909742051	2.189964163
LDL cholesterol	rs2820226	1.429827425	0.323977767	1.02E-05	0.794831001	2.06482385
LDL cholesterol	rs28406917	0.833736782	0.352439588	0.018000096	0.14295519	1.524518374
LDL cholesterol	rs28590710	0.492974231	0.309727551	0.111466001	-0.11409177	1.100040232
LDL cholesterol	rs28615248	0.841912263	0.283109571	0.002941333	0.287017505	1.396807022
LDL cholesterol	rs28631087	1.035836715	0.314147535	0.000976213	0.420107547	1.651565883
LDL cholesterol	rs28814720	1.386818566	0.349492801	7.25E-05	0.701812677	2.071824456
LDL cholesterol	rs3104412	1.433029551	0.219304352	6.39E-11	1.003193022	1.86286608
LDL cholesterol	rs3127580	0.064097235	0.159180015	0.687189996	-0.247895595	0.376090065
LDL cholesterol	rs34042070	0.959911547	0.10969031	2.11E-18	0.744918539	1.174904554
LDL cholesterol	rs34207171	0.839882787	0.356324338	0.018419582	0.141487083	1.53827849
LDL cholesterol	rs34265667	1.305179875	0.360906501	0.000298736	0.597803132	2.012556617
LDL cholesterol	rs34568880	1.341385133	0.322892799	3.26E-05	0.708515246	1.97425502
LDL cholesterol	rs34596921	0.877167156	0.157846111	2.74E-08	0.567788778	1.186545535
LDL cholesterol	rs35278712	0.701863663	0.253673486	0.005660957	0.20466363	1.199063696
LDL cholesterol	rs35511051	0.79583406	0.232857497	0.000631557	0.339433366	1.252234754
LDL cholesterol	rs35882350	0.860635434	0.337345007	0.010735192	0.19943922	1.521831649
LDL cholesterol	rs35980001	1.945166023	0.228319223	1.60E-17	1.497660345	2.392671701
LDL cholesterol	rs35990695	0.642530734	0.300451533	0.032472102	0.053645729	1.23141574
LDL cholesterol	rs3732359	0.893244832	0.287615525	0.001898352	0.329518403	1.45697126

LDL cholesterol	rs375972689	1.025160297	0.121437952	3.12E-17	0.787141911	1.263178683
LDL cholesterol	rs3780181	0.874942201	0.295917314	0.003109386	0.294944265	1.454940136
LDL cholesterol	rs3822855	0.845127734	0.236369649	0.000349627	0.381843222	1.308412245
LDL cholesterol	rs3823376	1.057039832	0.238730943	9.52E-06	0.589127183	1.524952481
LDL cholesterol	rs4148826	1.0255324	0.342434317	0.002745983	0.354361139	1.696703661
LDL cholesterol	rs4263041	0.847002774	0.071469298	2.12E-32	0.706922951	0.987082597
LDL cholesterol	rs4307732	1.068460792	0.150244806	1.15E-12	0.773980973	1.362940611
LDL cholesterol	rs438568	0.883488143	0.340430528	0.00945336	0.216244308	1.550731978
LDL cholesterol	rs440677	0.813800726	0.269627225	0.002542442	0.285331366	1.342270086
LDL cholesterol	rs4666384	0.807016173	0.267690134	0.002571996	0.28234351	1.331688835
LDL cholesterol	rs472495	0.757616684	0.101615529	8.94E-14	0.558450248	0.95678312
LDL cholesterol	rs4738684	0.906904346	0.13927035	7.42E-11	0.63393446	1.179874232
LDL cholesterol	rs4930163	0.725361833	0.323810833	0.025085797	0.090692601	1.360031066
LDL cholesterol	rs4954192	0.383980659	0.289579142	0.184841166	-0.183594459	0.951555777
LDL cholesterol	rs4970834	0.933723636	0.050619546	5.62E-76	0.834509326	1.032937945
LDL cholesterol	rs556107	0.879424074	0.117852106	8.52E-14	0.648433946	1.110414203
LDL cholesterol	rs55637835	1.025966045	0.344600249	0.002908331	0.350549557	1.701382533
LDL cholesterol	rs55714927	1.034799519	0.198882012	1.96E-07	0.644990776	1.424608261
LDL cholesterol	rs55831924	0.793035257	0.276457792	0.004123474	0.251177984	1.334892531
LDL cholesterol	rs56130071	0.757596489	0.151317669	5.54E-07	0.461013858	1.054179121
LDL cholesterol	rs56236159	-0.100321217	0.345387521	0.771464442	-0.777280757	0.576638324
LDL cholesterol	rs5843957	1.421223507	0.304922482	3.15E-06	0.823575442	2.018871572
LDL cholesterol	rs5849920	1.415248017	0.315008046	7.03E-06	0.797832246	2.032663787
LDL cholesterol	rs597808	0.74406971	0.153244228	1.20E-06	0.443711022	1.044428398
LDL cholesterol	rs60229127	1.244173962	0.313170546	7.10E-05	0.630359693	1.857988232
LDL cholesterol	rs6031587	0.646941855	0.321995953	0.044520015	0.015829787	1.278053923
LDL cholesterol	rs60612724	0.792800415	0.332358608	0.01706165	0.141377543	1.444223287
LDL cholesterol	rs6074012	0.691528871	0.355312905	0.051624335	-0.004884423	1.387942164
LDL cholesterol	rs61003864	0.994902735	0.341341703	0.003560504	0.325872998	1.663932472
LDL cholesterol	rs61754230	1.304082378	0.345977955	0.000163729	0.625965586	1.982199169
LDL cholesterol	rs61988556	0.779298531	0.328932271	0.017827755	0.134591279	1.424005782
LDL cholesterol	rs62033400	0.654096985	0.293178744	0.025677693	0.079466646	1.228727323
LDL cholesterol	rs6475606	0.543186853	0.20375156	0.007677629	0.143833795	0.942539911
LDL cholesterol	rs6495122	1.29361427	0.292552763	9.79E-06	0.720210856	1.867017685
LDL cholesterol	rs6544713	0.864766398	0.082300541	7.98E-26	0.703457338	1.026075457
LDL cholesterol	rs6560499	0.654707116	0.344915921	0.057674431	-0.021328089	1.330742321
LDL cholesterol	rs6602912	1.072009982	0.205847376	1.91E-07	0.668549125	1.47547084
LDL cholesterol	rs6667939	0.450854235	0.301813656	0.135223574	-0.140700531	1.042409

LDL cholesterol	rs6680227	1.230579133	0.150909278	3.51E-16	0.934796948	1.526361319
LDL cholesterol	rs6709904	0.542208973	0.150832624	0.000324675	0.246577031	0.837840915
LDL cholesterol	rs6732741	0.804744025	0.229195153	0.000446133	0.355521526	1.253966525
LDL cholesterol	rs6874202	1.373239306	0.132451208	3.47E-25	1.113634939	1.632843673
LDL cholesterol	rs7108486	1.053928591	0.355165026	0.003002973	0.357805139	1.750052043
LDL cholesterol	rs71311871	0.853062638	0.265079679	0.001290263	0.333506467	1.372618809
LDL cholesterol	rs7202323	0.923149218	0.191892389	1.50E-06	0.547040135	1.299258301
LDL cholesterol	rs7241918	0.241106616	0.344850163	0.484449913	-0.434799703	0.917012936
LDL cholesterol	rs72631343	1.45236315	0.210594654	5.33E-12	1.039597628	1.865128672
LDL cholesterol	rs72911393	0.651257536	0.319238962	0.041347189	0.025549171	1.276965902
LDL cholesterol	rs7562734	0.835103368	0.219873612	0.000145806	0.404151089	1.266055647
LDL cholesterol	rs7569317	0.797494547	0.23168447	0.000577093	0.343392987	1.251596108
LDL cholesterol	rs76468627	0.853721716	0.360641558	0.017921764	0.146864263	1.560579169
LDL cholesterol	rs7707394	0.860932555	0.106463113	6.13E-16	0.652264854	1.069600257
LDL cholesterol	rs7734476	1.069324875	0.221697273	1.41E-06	0.63479822	1.503851531
LDL cholesterol	rs7746081	0.974029183	0.191654489	3.73E-07	0.598386384	1.349671982
LDL cholesterol	rs77542162	1.011791351	0.10819713	8.65E-21	0.799724977	1.223857725
LDL cholesterol	rs7776054	0.573327953	0.290079844	0.048103972	0.004771458	1.141884448
LDL cholesterol	rs77960347	0.484222093	0.255725996	0.058288853	-0.01700086	0.985445045
LDL cholesterol	rs78508096	1.321831412	0.27990264	2.33E-06	0.773222238	1.870440586
LDL cholesterol	rs79220007	0.892334926	0.13592539	5.21E-11	0.625921162	1.15874869
LDL cholesterol	rs79828839	1.08618014	0.357169953	0.002357361	0.386127032	1.786233248
LDL cholesterol	rs8107974	1.003693198	0.074437644	1.95E-41	0.857795416	1.14959098
LDL cholesterol	rs869412	1.159253482	0.347804854	0.000858973	0.477555968	1.840950996
LDL cholesterol	rs880315	0.863123666	0.287165709	0.002650015	0.300278877	1.425968455
LDL cholesterol	rs9289196	0.966862788	0.314920381	0.002139286	0.349618841	1.584106735
LDL cholesterol	rs934197	0.957444482	0.052497753	2.59E-74	0.854548886	1.060340079
LDL cholesterol	rs9471968	1.237524538	0.356800806	0.000523586	0.538194958	1.936854117
LDL cholesterol	rs9496567	1.299737259	0.27669405	2.64E-06	0.757416921	1.842057597
LDL cholesterol	rs960596	0.883999171	0.325369444	0.006589472	0.246275061	1.52172328
LDL cholesterol	rs964184	1.660083046	0.105807687	1.78E-55	1.452699979	1.867466114
LDL cholesterol	rs9832727	0.993997453	0.296672177	0.000806684	0.412519986	1.575474919
LDL cholesterol	rs9834932	0.896434203	0.225199583	6.87E-05	0.45504302	1.337825385
LDL cholesterol	rs9884390	0.582749146	0.196462271	0.003014906	0.197683095	0.967815197
LDL cholesterol	rs9894946	0.921174848	0.331314868	0.005429854	0.271797707	1.57055199
LDL cholesterol	rs9929977	0.508375132	0.256368484	0.047368986	0.005892903	1.010857361
LDL cholesterol	rs9987289	0.581553642	0.158507703	0.000243568	0.270878543	0.892228741
LDL cholesterol	All - Inverse variance weighted	0.881020948	0.028057176	1.97E-216	0.826028884	0.936013012

LDL cholesterol	All - MR Egger	0.856607973	0.04198324	3.21E-47	0.774320823	0.938895124
Triglycerides	rs1009360	0.178713102	0.226250866	0.429592514	-0.264738595	0.622164798
Triglycerides	rs1009590	1.080891423	0.358617363	0.002577835	0.378001392	1.783781454
Triglycerides	rs10152471	-0.955335813	0.315169109	0.002435961	-1.573067266	-0.337604361
Triglycerides	rs10210970	0.688257072	0.261348401	0.008451359	0.176014207	1.200499938
Triglycerides	rs10242866	0.046501425	0.267212056	0.861846266	-0.477234205	0.570237054
Triglycerides	rs10277582	0.685483035	0.369533831	0.063597085	-0.038803274	1.409769345
Triglycerides	rs1037117	-0.472466074	0.27664404	0.08766448	-1.014688392	0.069756244
Triglycerides	rs10405944	0.584435612	0.317280642	0.065472997	-0.037434446	1.20630567
Triglycerides	rs1043897	0.32785461	0.285567376	0.250934937	-0.231857447	0.887566667
Triglycerides	rs1044808	0.490266352	0.304762982	0.107686161	-0.107069093	1.087601797
Triglycerides	rs1045241	0.092621311	0.225841112	0.681720018	-0.350027267	0.53526989
Triglycerides	rs10513688	0.791665994	0.280760043	0.004806488	0.241376309	1.341955679
Triglycerides	rs10631642	0.301503954	0.348089994	0.386398763	-0.380752434	0.983760342
Triglycerides	rs1064173	1.260481775	0.223579371	1.72E-08	0.822266208	1.698697343
Triglycerides	rs10642257	-0.481072445	0.15258503	0.00161704	-0.780139103	-0.182005786
Triglycerides	rs10750766	-0.303456707	0.234914756	0.19643552	-0.763889629	0.156976214
Triglycerides	rs10773000	0.424349271	0.29525567	0.150653648	-0.154351843	1.003050384
Triglycerides	rs10773049	0.361503867	0.145862575	0.013197802	0.07561322	0.647394514
Triglycerides	rs10775406	0.134123376	0.234602631	0.567522194	-0.325697779	0.593944532
Triglycerides	rs1077835	0.883994697	0.105574157	5.61E-17	0.677069348	1.090920045
Triglycerides	rs10797119	-0.049836042	0.26476867	0.850700398	-0.568782634	0.469110551
Triglycerides	rs10811662	0.263514127	0.353693753	0.456250729	-0.429725629	0.956753884
Triglycerides	rs10883026	0.652515836	0.288355094	0.023642476	0.087339852	1.21769182
Triglycerides	rs10899490	0.40591009	0.329288226	0.217691685	-0.239494832	1.051315013
Triglycerides	rs11000468	-0.140788588	0.324934699	0.664809396	-0.777660599	0.496083423
Triglycerides	rs11030107	-0.140850777	0.293127953	0.630865123	-0.715381566	0.433680011
Triglycerides	rs11078597	0.872983913	0.27807864	0.001693278	0.327949778	1.418018048
Triglycerides	rs11100083	-0.249447731	0.308310868	0.418469927	-0.853737033	0.354841571
Triglycerides	rs11118310	0.306079367	0.21784727	0.160015883	-0.120901283	0.733060017
Triglycerides	rs11122450	0.560630624	0.088000291	1.88E-10	0.388150054	0.733111194
Triglycerides	rs11185542	0.024255232	0.367612643	0.947393425	-0.696265548	0.744776012
Triglycerides	rs11187019	0.78126197	0.358594777	0.029355718	0.078416208	1.484107732
Triglycerides	rs11206374	0.21311383	0.198026232	0.281842336	-0.175017584	0.601245244
Triglycerides	rs112108602	0.081235506	0.270603697	0.764023827	-0.44914774	0.611618752
Triglycerides	rs112381903	0.63471459	0.359866868	0.077774359	-0.070624471	1.34005365
Triglycerides	rs11240358	0.721585539	0.310948684	0.020308786	0.112126118	1.331044961
Triglycerides	rs11274835	0.644338777	0.226387578	0.00442478	0.200619124	1.088058429

Triglycerides	rs1133400	0.110183905	0.363268394	0.761651594	-0.601822147	0.822189958
Triglycerides	rs113344423	0.180240892	0.206408527	0.382540963	-0.224319821	0.584801606
Triglycerides	rs114165349	0.906837089	0.169462894	8.74E-08	0.574689816	1.238984362
Triglycerides	rs11429307	0.520153336	0.113183578	4.31E-06	0.298313523	0.74199315
Triglycerides	rs11434143	1.005926717	0.337268556	0.002858403	0.344880347	1.666973088
Triglycerides	rs11600815	0.573918733	0.295235201	0.051903007	-0.00474226	1.152579727
Triglycerides	rs11637681	0.100085011	0.36992357	0.786732604	-0.624965185	0.825135207
Triglycerides	rs11664106	0.96052642	0.347723534	0.005739081	0.278988294	1.642064547
Triglycerides	rs116843064	0.308929604	0.065336306	2.26E-06	0.180870444	0.436988764
Triglycerides	rs117233107	0.499361615	0.245612214	0.042039369	0.017961675	0.980761555
Triglycerides	rs117287238	0.735631105	0.327814183	0.024829246	0.093115306	1.378146905
Triglycerides	rs117291242	0.824284626	0.369171163	0.02556227	0.100709147	1.547860105
Triglycerides	rs117316645	0.972827828	0.369831654	0.008526909	0.247957786	1.69769787
Triglycerides	rs117431393	-0.076098305	0.35865033	0.831966875	-0.77905295	0.626856341
Triglycerides	rs11746801	0.586766621	0.350334393	0.093958589	-0.099888789	1.27342203
Triglycerides	rs11904650	0.914896467	0.352607265	0.009468414	0.223786227	1.606006707
Triglycerides	rs12185242	0.269094799	0.237817591	0.257837282	-0.197027678	0.735217277
Triglycerides	rs12424054	0.927122775	0.255900257	0.000291223	0.42555827	1.428687279
Triglycerides	rs12440800	0.263536025	0.297296273	0.375379175	-0.319164671	0.84623672
Triglycerides	rs12446515	1.605528909	0.132710266	1.08E-33	1.345416787	1.865641031
Triglycerides	rs12475332	0.212945067	0.335143957	0.52517808	-0.443937089	0.869827223
Triglycerides	rs12504746	0.627040623	0.345180581	0.069284785	-0.049513316	1.303594561
Triglycerides	rs12530679	0.013836965	0.346306235	0.968128314	-0.664923256	0.692597185
Triglycerides	rs12669911	-0.087053722	0.360235709	0.809045432	-0.793115712	0.619008268
Triglycerides	rs12880341	0.513080841	0.271467744	0.058754608	-0.018995937	1.045157619
Triglycerides	rs12902047	0.558718173	0.345463548	0.105813554	-0.118390382	1.235826727
Triglycerides	rs1292065	0.13953475	0.326985643	0.669575626	-0.501357111	0.780426611
Triglycerides	rs12926107	0.02173847	0.329185528	0.947348259	-0.623465164	0.666942104
Triglycerides	rs12928099	-0.211922046	0.161081966	0.188302889	-0.5276427	0.103798608
Triglycerides	rs12948505	0.085341321	0.375976873	0.820434972	-0.651573351	0.822255992
Triglycerides	rs13066793	0.157076894	0.321651238	0.625305229	-0.473359533	0.78751332
Triglycerides	rs13101504	0.61620262	0.256520005	0.016298487	0.113423409	1.11898183
Triglycerides	rs13107325	0.222353857	0.262056067	0.396161094	-0.291276034	0.735983747
Triglycerides	rs13108218	0.79119111	0.140229184	1.68E-08	0.516341909	1.066040312
Triglycerides	rs13118477	0.224024497	0.283366578	0.429187994	-0.331373997	0.77942299
Triglycerides	rs1316753	0.213143304	0.289941811	0.462263726	-0.355142645	0.781429254
Triglycerides	rs13264304	-0.002914533	0.306713837	0.992418256	-0.604073653	0.598244588
Triglycerides	rs13269725	0.255433037	0.220097863	0.245827792	-0.175958774	0.686824848

Triglycerides	rs13354321	0.249144227	0.271805191	0.359337648	-0.283593947	0.781882402
Triglycerides	rs13389219	0.601828079	0.112507376	8.83E-08	0.381313623	0.822342535
Triglycerides	rs1340819	0.228560273	0.357644158	0.522776265	-0.472422276	0.929542822
Triglycerides	rs134551	0.586403343	0.376124116	0.118980117	-0.150799924	1.323606611
Triglycerides	rs1347188	0.689852612	0.346230426	0.046319869	0.011240978	1.368464246
Triglycerides	rs138191773	0.280704321	0.349732997	0.422191722	-0.404772353	0.966180994
Triglycerides	rs139386986	0.366870444	0.341072277	0.282088998	-0.301631218	1.035372106
Triglycerides	rs139974673	0.192377318	0.091106638	0.034724032	0.013808308	0.370946328
Triglycerides	rs140107293	0.618405924	0.25243499	0.014295023	0.123633344	1.113178504
Triglycerides	rs140288	0.263326874	0.31488505	0.403006682	-0.353847824	0.880501572
Triglycerides	rs1420384	-0.248165754	0.342919468	0.469259003	-0.920287912	0.423956403
Triglycerides	rs143076454	0.939361236	0.374156867	0.012052344	0.206013777	1.672708695
Triglycerides	rs145947882	0.216669701	0.095823291	0.023750702	0.028856051	0.404483351
Triglycerides	rs1473886	0.650570032	0.228779702	0.004459962	0.202161816	1.098978247
Triglycerides	rs148827772	0.486189519	0.3180707	0.12637405	-0.137229053	1.109608091
Triglycerides	rs149142833	0.900170054	0.338849065	0.007894435	0.236025886	1.564314222
Triglycerides	rs149778057	0.280730859	0.294043841	0.339717037	-0.29559507	0.857056788
Triglycerides	rs150419156	0.115074452	0.345511592	0.739092572	-0.562128267	0.792277172
Triglycerides	rs150423652	0.50481765	0.090515842	2.45E-08	0.3274066	0.6822287
Triglycerides	rs150460588	0.472387384	0.309338986	0.126739507	-0.133917028	1.078691796
Triglycerides	rs150555490	0.341487219	0.231606521	0.140366298	-0.112461562	0.795436
Triglycerides	rs150564454	0.4508909	0.198450138	0.023082802	0.06192863	0.839853171
Triglycerides	rs151235402	0.147598753	0.327949746	0.652663462	-0.49518275	0.790380256
Triglycerides	rs1544980	0.205142771	0.217530079	0.345652907	-0.221216184	0.631501726
Triglycerides	rs1567353	0.406920959	0.303665942	0.180236142	-0.188264288	1.002106206
Triglycerides	rs17184382	0.44248008	0.190925925	0.020473875	0.068265267	0.816694893
Triglycerides	rs17326656	0.520239802	0.279897244	0.063072311	-0.028358797	1.0688384
Triglycerides	rs174566	-0.670924232	0.089267225	5.65E-14	-0.845887992	-0.495960472
Triglycerides	rs17585887	0.684396307	0.147012584	3.23E-06	0.396251643	0.972540971
Triglycerides	rs1760801	0.188202837	0.224216552	0.401256236	-0.251261606	0.627667279
Triglycerides	rs1799831	0.508757039	0.232656084	0.028761926	0.052751114	0.964762964
Triglycerides	rs1801689	-0.245384848	0.181701195	0.176860268	-0.601519189	0.110749493
Triglycerides	rs1835346	0.999688678	0.348282752	0.004100353	0.317054484	1.682322872
Triglycerides	rs186413375	0.43733379	0.230613234	0.057907829	-0.014668148	0.889335728
Triglycerides	rs186696265	1.766991315	0.167148527	4.04E-26	1.439380203	2.094602427
Triglycerides	rs188247550	0.619824107	0.140369808	1.01E-05	0.344699284	0.89494893
Triglycerides	rs193735	0.044744957	0.333443499	0.893251921	-0.608804301	0.698294214
Triglycerides	rs1938566	0.429148105	0.26200578	0.101435518	-0.084383223	0.942679433

Triglycerides	rs200610097	0.133951618	0.373290249	0.719714497	-0.597697271	0.865600506
Triglycerides	rs2043085	1.290288545	0.137722869	7.34E-21	1.020351721	1.560225369
Triglycerides	rs2068888	0.518182618	0.130522664	7.19E-05	0.262358197	0.774007039
Triglycerides	rs2070341	1.027961632	0.374942484	0.006113116	0.293074364	1.762848901
Triglycerides	rs2071887	0.601849559	0.266274522	0.02380514	0.079951496	1.123747621
Triglycerides	rs2081194	1.164098732	0.201985382	8.25E-09	0.768207384	1.55999008
Triglycerides	rs2081687	1.094303674	0.167606043	6.62E-11	0.765795829	1.422811519
Triglycerides	rs2092203	1.587586651	0.301839042	1.44E-07	0.995982129	2.179191173
Triglycerides	rs2131311	0.044373616	0.37404756	0.905567747	-0.688759601	0.777506833
Triglycerides	rs2131919	0.2712122	0.321904663	0.399495067	-0.35972094	0.902145339
Triglycerides	rs213494	0.302331749	0.277246871	0.275502423	-0.241072118	0.845735616
Triglycerides	rs2137557	0.040560345	0.367012684	0.912001088	-0.678784517	0.759905206
Triglycerides	rs2187114	0.381492403	0.369775481	0.302218963	-0.343267538	1.106252345
Triglycerides	rs2237029	-0.22551453	0.304492141	0.45892077	-0.822319127	0.371290067
Triglycerides	rs2240466	0.234279012	0.050991863	4.34E-06	0.134334961	0.334223063
Triglycerides	rs2240533	0.569475046	0.344618428	0.098436748	-0.105977074	1.244927165
Triglycerides	rs2244278	1.211446694	0.236205875	2.92E-07	0.74848318	1.674410208
Triglycerides	rs2267373	0.518442946	0.194926683	0.007821451	0.136386648	0.900499244
Triglycerides	rs2302263	0.097537559	0.167659913	0.560729443	-0.23107587	0.426150989
Triglycerides	rs2304969	0.683982684	0.365743889	0.06146802	-0.032875338	1.400840706
Triglycerides	rs2305746	0.860789824	0.290514752	0.003046749	0.291380911	1.430198737
Triglycerides	rs2382825	0.786487008	0.317068574	0.013120011	0.165032603	1.407941413
Triglycerides	rs2407278	0.493750166	0.362466821	0.173136637	-0.216684804	1.204185135
Triglycerides	rs2487294	-0.350739938	0.252297875	0.164473649	-0.845243773	0.143763897
Triglycerides	rs2519093	-2.296861707	0.252440212	9.15E-20	-2.791644522	-1.802078892
Triglycerides	rs2604568	1.259469908	0.37523926	0.000789514	0.524000958	1.994938857
Triglycerides	rs2699805	-0.132696845	0.210435491	0.528313833	-0.545150408	0.279756717
Triglycerides	rs275184	1.086796129	0.329969969	0.000989056	0.44005499	1.733537268
Triglycerides	rs2773469	0.96067127	0.248616712	0.000111515	0.473382515	1.447960025
Triglycerides	rs278981	0.811664323	0.378526428	0.03201095	0.069752524	1.553576122
Triglycerides	rs2812208	0.302868294	0.30002478	0.312745641	-0.285180275	0.890916863
Triglycerides	rs28383314	0.630110421	0.112493767	2.13E-08	0.409622638	0.850598203
Triglycerides	rs28439112	0.787493453	0.376293904	0.036370436	0.049957402	1.525029505
Triglycerides	rs28577186	0.231112172	0.268596569	0.389544615	-0.295337103	0.757561446
Triglycerides	rs28752924	0.609283444	0.235917171	0.009805441	0.14688579	1.071681099
Triglycerides	rs2925979	0.261866796	0.139819679	0.061083658	-0.012179775	0.535913366
Triglycerides	rs2937124	0.375901206	0.242169467	0.120608687	-0.098750949	0.85055336
Triglycerides	rs2943645	0.148555041	0.106999439	0.165023753	-0.06116386	0.358273942

Triglycerides	rs2983896	0.413059052	0.366334329	0.259511421	-0.304956233	1.131074337
Triglycerides	rs308	0.460251046	0.092351314	6.24E-07	0.279242471	0.641259621
Triglycerides	rs3103310	0.677682931	0.243452202	0.00537529	0.200516616	1.154849246
Triglycerides	rs320369	0.688777358	0.356484237	0.053342262	-0.009931747	1.387486463
Triglycerides	rs325485	0.92092647	0.361643462	0.010880801	0.212105284	1.629747656
Triglycerides	rs326222	-0.043671963	0.17820129	0.806401482	-0.392946492	0.305602566
Triglycerides	rs343	0.338625232	0.05334384	2.18E-10	0.234071305	0.443179159
Triglycerides	rs34302257	0.020893675	0.374274108	0.955481596	-0.712683577	0.754470927
Triglycerides	rs34389637	0.588055853	0.331620453	0.076182353	-0.061920235	1.238031941
Triglycerides	rs34672664	-0.436724589	0.375878664	0.245285632	-1.173446771	0.299997594
Triglycerides	rs34682685	1.353927356	0.202113932	2.10E-11	0.957784049	1.750070664
Triglycerides	rs35140741	0.138102453	0.276443737	0.617379273	-0.403727271	0.679932177
Triglycerides	rs35763453	0.755732865	0.318573638	0.017680641	0.131328534	1.380137195
Triglycerides	rs35786744	0.183997003	0.371477717	0.620380601	-0.544099322	0.912093327
Triglycerides	rs36043408	0.379838684	0.322798894	0.239313752	-0.252847148	1.012524517
Triglycerides	rs3731696	0.684026144	0.289086915	0.017973633	0.117415792	1.250636497
Triglycerides	rs3758413	1.083923691	0.377940701	0.004131109	0.343159918	1.824687464
Triglycerides	rs3775228	0.412055695	0.125287037	0.001005821	0.166493103	0.657618288
Triglycerides	rs3808477	2.014724999	0.343965066	4.70E-09	1.340553469	2.688896529
Triglycerides	rs3814883	-0.077249775	0.278767307	0.781694083	-0.623633697	0.469134147
Triglycerides	rs3820897	0.230618597	0.275217089	0.402057894	-0.308806898	0.770044092
Triglycerides	rs3860846	0.503846452	0.157515647	0.001380452	0.195115784	0.812577119
Triglycerides	rs394872	0.365122041	0.372671657	0.327213534	-0.365314408	1.095558489
Triglycerides	rs3974807	0.183045013	0.332380958	0.581833529	-0.468421665	0.834511691
Triglycerides	rs4128205	1.057490153	0.361428037	0.003434984	0.3490912	1.765889106
Triglycerides	rs4134963	-0.304970877	0.279079264	0.274492546	-0.851966235	0.24202448
Triglycerides	rs41785	0.476026455	0.278985676	0.087956777	-0.07078547	1.022838379
Triglycerides	rs4253750	0.296697752	0.285021061	0.297890485	-0.261943528	0.855339032
Triglycerides	rs4471666	-0.476247718	0.365979253	0.1931568	-1.193567054	0.241071617
Triglycerides	rs4665972	0.461117207	0.042401596	1.52E-27	0.378010079	0.544224335
Triglycerides	rs4675812	0.302971786	0.295124556	0.304613776	-0.275472345	0.881415917
Triglycerides	rs4731701	0.47219025	0.127117921	0.000203538	0.223039125	0.721341375
Triglycerides	rs4760254	0.671992154	0.171727064	9.11E-05	0.335407108	1.008577199
Triglycerides	rs4761234	0.274376052	0.295321458	0.352849749	-0.304454006	0.85320611
Triglycerides	rs4765148	0.850105551	0.177182283	1.60E-06	0.502828276	1.197382826
Triglycerides	rs480823	0.365915104	0.049788852	1.99E-13	0.268328955	0.463501254
Triglycerides	rs483082	-0.550421694	0.056369828	1.60E-22	-0.660906557	-0.43993683
Triglycerides	rs483808	0.319173571	0.332782722	0.337505855	-0.333080564	0.971427706

Triglycerides	rs4841580	0.338188057	0.170396482	0.047176448	0.004210952	0.672165162
Triglycerides	rs4969179	0.254915535	0.238538492	0.285224811	-0.21261991	0.72245098
Triglycerides	rs4976033	0.650321232	0.240352652	0.006816139	0.179230033	1.12141243
Triglycerides	rs499293	0.680152147	0.369114255	0.065378638	-0.043311792	1.403616087
Triglycerides	rs55646464	0.751763143	0.371321352	0.042912348	0.023973292	1.479552993
Triglycerides	rs55767272	-0.106574212	0.296872462	0.719602655	-0.688444238	0.475295815
Triglycerides	rs55807798	0.118265351	0.359258356	0.742009783	-0.585881028	0.822411729
Triglycerides	rs55966194	0.182405369	0.257611101	0.478904978	-0.322512389	0.687323127
Triglycerides	rs56397607	0.477789089	0.30627906	0.1187647	-0.12251787	1.078096047
Triglycerides	rs56902258	0.358500733	0.345157626	0.298963817	-0.318008215	1.035009681
Triglycerides	rs57996145	0.456087241	0.192195758	0.017642597	0.079383556	0.832790926
Triglycerides	rs581080	0.384269119	0.30717372	0.21094066	-0.217791372	0.986329609
Triglycerides	rs58542926	1.031594046	0.076239243	1.03E-41	0.88216513	1.181022961
Triglycerides	rs6028716	0.206978794	0.370649758	0.576556595	-0.519494732	0.933452319
Triglycerides	rs6073958	0.139966101	0.093141958	0.132911768	-0.042592137	0.32252434
Triglycerides	rs60856912	0.887765827	0.227023022	9.21E-05	0.442800704	1.33273095
Triglycerides	rs61729990	0.408359714	0.280525275	0.145476486	-0.141469826	0.958189254
Triglycerides	rs61830291	0.754431551	0.243581904	0.001953318	0.27701102	1.231852082
Triglycerides	rs61905078	0.342968414	0.039694694	5.61E-18	0.265166813	0.420770015
Triglycerides	rs61993685	0.58277478	0.331060247	0.078352128	-0.066103303	1.231652864
Triglycerides	rs62102718	0.165276652	0.226491984	0.465558866	-0.278647637	0.609200941
Triglycerides	rs62117489	0.593678915	0.20938601	0.004577804	0.183282335	1.004075494
Triglycerides	rs62128802	0.524487546	0.337712446	0.120408904	-0.137428849	1.186403941
Triglycerides	rs62135012	0.434194174	0.368950137	0.239260569	-0.288948094	1.157336443
Triglycerides	rs62271373	0.461132068	0.211498931	0.029234899	0.046594163	0.875669973
Triglycerides	rs62274099	0.460137159	0.348759948	0.187051438	-0.223432339	1.143706656
Triglycerides	rs62397245	0.504714521	0.331779166	0.128200542	-0.145572645	1.155001687
Triglycerides	rs62427982	0.625729049	0.333562143	0.060669085	-0.028052751	1.279510848
Triglycerides	rs62459095	0.499989058	0.276763635	0.070831505	-0.042467666	1.042445782
Triglycerides	rs62473520	0.876001763	0.374736852	0.019405667	0.141517533	1.610485993
Triglycerides	rs6432622	0.413758864	0.37896076	0.274909991	-0.329004225	1.156521953
Triglycerides	rs6506033	-0.130019044	0.348576969	0.709148798	-0.813229904	0.553191816
Triglycerides	rs6517522	0.819051236	0.32055165	0.010614805	0.190770001	1.44733247
Triglycerides	rs6532798	0.406969258	0.326791618	0.213003893	-0.233542314	1.047480829
Triglycerides	rs6562773	0.744922763	0.346762017	0.031695902	0.06526921	1.424576316
Triglycerides	rs6572807	0.099560753	0.374251156	0.790218743	-0.633971513	0.833093019
Triglycerides	rs676210	1.09267529	0.069311954	5.45E-56	0.95682386	1.22852672
Triglycerides	rs6792725	0.633051664	0.302429479	0.036329452	0.040289885	1.225813443

Triglycerides	rs67981690	0.453922633	0.20708898	0.028385229	0.048028233	0.859817034
Triglycerides	rs6800707	0.394227166	0.176786192	0.025749712	0.047726229	0.740728103
Triglycerides	rs6805924	1.016292258	0.380886911	0.007625301	0.269753911	1.762830604
Triglycerides	rs684773	0.8352746	0.168011527	6.64E-07	0.505972006	1.164577193
Triglycerides	rs6882076	1.341317962	0.129352499	3.41E-25	1.087787064	1.59484886
Triglycerides	rs696825	-0.049122748	0.234834696	0.834307636	-0.509398752	0.411153255
Triglycerides	rs698927	0.233367931	0.291547534	0.42345269	-0.338065236	0.804801097
Triglycerides	rs6999569	0.665364131	0.048129193	1.81E-43	0.571030914	0.759697349
Triglycerides	rs7000494	0.475939299	0.089647571	1.10E-07	0.30023006	0.651648537
Triglycerides	rs7077812	1.001159653	0.366831829	0.006348702	0.282169267	1.720150038
Triglycerides	rs7134375	0.350629101	0.243099717	0.14921036	-0.125846344	0.827104547
Triglycerides	rs7135509	0.887974089	0.378929107	0.019110017	0.145273039	1.630675139
Triglycerides	rs71368855	0.436420818	0.249823572	0.080651894	-0.053233383	0.92607502
Triglycerides	rs7140110	0.846492591	0.159474807	1.11E-07	0.53392197	1.159063212
Triglycerides	rs71538127	0.061531837	0.357956078	0.863517932	-0.640062076	0.763125749
Triglycerides	rs71603401	0.505156779	0.229262881	0.027566891	0.055801533	0.954512025
Triglycerides	rs7215055	0.636097145	0.218866078	0.00365695	0.207119633	1.065074657
Triglycerides	rs7239575	0.248336412	0.257592043	0.335011511	-0.256543992	0.753216817
Triglycerides	rs7244	0.532235587	0.358849307	0.138028465	-0.171109055	1.235580229
Triglycerides	rs72555385	0.60278291	0.146765253	4.01E-05	0.315123015	0.890442806
Triglycerides	rs72603744	0.195440463	0.354257698	0.581160698	-0.498904625	0.88978555
Triglycerides	rs72644085	0.558739211	0.298405905	0.061149434	-0.026136363	1.143614785
Triglycerides	rs7274718	0.228372761	0.263506405	0.386123544	-0.288099792	0.744845314
Triglycerides	rs72784786	0.171600726	0.331917349	0.605157302	-0.478957278	0.822158729
Triglycerides	rs72801474	0.16186635	0.231693393	0.484787621	-0.2922527	0.6159854
Triglycerides	rs729761	0.310589876	0.259507612	0.231367745	-0.198045045	0.819224796
Triglycerides	rs73025562	1.8476986	0.347756306	1.08E-07	1.16609624	2.52930096
Triglycerides	rs7308584	-0.042279322	0.356798363	0.905674347	-0.741604112	0.657045469
Triglycerides	rs73238173	0.675432993	0.370643855	0.06840592	-0.051028963	1.401894949
Triglycerides	rs7400002	1.035697669	0.352403347	0.003293206	0.344987108	1.726408229
Triglycerides	rs74090351	0.934945241	0.329024738	0.004489267	0.290056754	1.579833729
Triglycerides	rs742036	0.592659714	0.297355742	0.046250504	0.009842459	1.175476969
Triglycerides	rs7424120	0.09062892	0.342183213	0.791121199	-0.580050178	0.761308018
Triglycerides	rs75268115	1.34880806	0.360468098	0.00018269	0.642290587	2.055325533
Triglycerides	rs75609851	0.386837823	0.104702284	0.000220194	0.181621346	0.5920543
Triglycerides	rs75634664	0.679084233	0.247999658	0.006176773	0.193004904	1.165163563
Triglycerides	rs75721796	0.37251949	0.243398055	0.125894266	-0.104540698	0.849579678
Triglycerides	rs75942983	0.546628714	0.372967119	0.142751368	-0.18438684	1.277644268

Triglycerides	rs77009508	0.397019818	0.175078951	0.023349839	0.053865073	0.740174562
Triglycerides	rs7704653	0.656459282	0.296586747	0.026871433	0.075149258	1.237769305
Triglycerides	rs7714361	0.5136708	0.354897809	0.147791463	-0.181928906	1.209270507
Triglycerides	rs7735249	0.427565284	0.245743853	0.081879756	-0.054092668	0.909223236
Triglycerides	rs7786339	0.027289809	0.339431039	0.935920173	-0.637995027	0.692574645
Triglycerides	rs78058190	0.047798047	0.131116043	0.715449088	-0.209189397	0.30478549
Triglycerides	rs7847285	0.371047509	0.363514608	0.30738597	-0.341441122	1.08353614
Triglycerides	rs78484485	0.253644884	0.121483819	0.036807735	0.015536598	0.49175317
Triglycerides	rs78588343	-0.038505453	0.348032287	0.911903739	-0.720648736	0.64363783
Triglycerides	rs7861679	0.755846753	0.3697857	0.040952269	0.031066781	1.480626724
Triglycerides	rs79153732	0.519300024	0.204507087	0.011108202	0.118466132	0.920133915
Triglycerides	rs79287178	0.584342424	0.250176666	0.019505885	0.093996159	1.074688689
Triglycerides	rs79357714	0.792218695	0.34195873	0.020519617	0.121979583	1.462457806
Triglycerides	rs7947951	0.358437792	0.230602187	0.120099627	-0.093542495	0.810418079
Triglycerides	rs80276949	0.681844904	0.304030265	0.024916967	0.085945586	1.277744223
Triglycerides	rs8102873	0.241658692	0.339627094	0.476748891	-0.424010413	0.907327797
Triglycerides	rs8126001	0.598891793	0.252720146	0.017798549	0.103560307	1.094223278
Triglycerides	rs852388	0.20366844	0.324741605	0.530547306	-0.432825106	0.840161987
Triglycerides	rs867939	0.317683465	0.309045337	0.303972875	-0.288045396	0.923412325
Triglycerides	rs880315	1.116114509	0.371337071	0.002650015	0.38829385	1.843935168
Triglycerides	rs921971	0.33111246	0.300856869	0.271086141	-0.258567003	0.920791923
Triglycerides	rs9373056	0.210154755	0.359847443	0.559213116	-0.495146232	0.915455743
Triglycerides	rs9376511	0.465727484	0.330162007	0.158361737	-0.18139005	1.112845019
Triglycerides	rs9425589	0.529685797	0.302288945	0.079730833	-0.062800535	1.122172129
Triglycerides	rs9436661	0.528908226	0.055659945	2.05E-21	0.419814733	0.638001719
Triglycerides	rs9480889	0.459232708	0.308124881	0.136116206	-0.144692059	1.063157475
Triglycerides	rs954244	0.825014684	0.309738302	0.007731259	0.217927612	1.432101756
Triglycerides	rs9561643	0.148222245	0.266114066	0.577535876	-0.373361324	0.669805814
Triglycerides	rs9584870	-0.06821191	0.353526974	0.847000607	-0.761124779	0.62470096
Triglycerides	rs970069	-0.088101428	0.312566996	0.778047686	-0.700732741	0.524529885
Triglycerides	rs9831084	0.79870566	0.35111293	0.022918961	0.110524317	1.486887003
Triglycerides	rs9859117	0.036225179	0.349364246	0.917416272	-0.648528744	0.720979101
Triglycerides	rs9889402	0.389632573	0.377933191	0.302561352	-0.351116481	1.130381626
Triglycerides	rs9902027	0.874018033	0.326882309	0.007499708	0.233328707	1.514707359
Triglycerides	rs998584	0.338891077	0.103143461	0.001017552	0.136729893	0.541052261
Triglycerides	All - Inverse variance weighted	0.419592851	0.023567393	6.58E-71	0.373400761	0.46578494
Triglycerides	All - MR Egger	0.400156257	0.035964174	2.78E-24	0.329666476	0.470646038
