# Supplementary material for: Small dense low density lipoprotein predominance in patients with type 2 diabetes mellitus using Mendelian randomization
Source: PLoS One. 2024 Feb 8;19(2):e0298070. doi: 10.1371/journal.pone.0298070 (PMC10852223; doi:10.1371/journal.pone.0298070)
Supplement: S4 Table — (PDF) [file pone.0298070.s004.pdf]

Supplementary Table 4

Mendelian randomization analysis of individual SNPS (with cholesterol in small LDL as the outcome)

Exposure	SNP	b	se	p	95% CI	
T2DM	rs1046317	-0.069543468	0.052631948	0.186395811	-0.172702086	0.03361515
T2DM	rs10743152	-0.028428158	0.065040487	0.662050408	-0.155907513	0.099051196
T2DM	rs10830963	-0.017159498	0.035184422	0.625761232	-0.086120967	0.05180197
T2DM	rs10882099	0.059202157	0.05337703	0.267373365	-0.045416822	0.163821137
T2DM	rs10938397	0.073556334	0.056291779	0.191315424	-0.036775553	0.183888221
T2DM	rs112108223	-0.054368024	0.051084389	0.287202497	-0.154493427	0.045757379
T2DM	rs11257658	-0.045502225	0.06130726	0.457966401	-0.165664454	0.074660005
T2DM	rs11263763	-0.015123008	0.062651729	0.809258966	-0.137920397	0.107674382
T2DM	rs112694524	-0.038454621	0.045131046	0.39417757	-0.126911471	0.050002229
T2DM	rs11558471	0.076223219	0.054726781	0.163681618	-0.031041273	0.18348771
T2DM	rs11712037	-0.012351604	0.058479377	0.832721075	-0.126971182	0.102267974
T2DM	rs117657619	-0.156899576	0.087603253	0.073289358	-0.328601952	0.014802801
T2DM	rs12449219	-0.075243535	0.047433129	0.112669543	-0.168212468	0.017725399
T2DM	rs12967878	0.062645844	0.064474026	0.331227348	-0.063723247	0.189014935
T2DM	rs13389219	0.219583569	0.059966997	0.000250508	0.102048255	0.337118884
T2DM	rs144155527	-0.001793966	0.071440443	0.979966149	-0.141817235	0.138229303
T2DM	rs1798085	0.053683946	0.069387625	0.439118955	-0.082315799	0.189683692
T2DM	rs182788819	0.05281269	0.109003807	0.628028813	-0.160834772	0.266460152
T2DM	rs2237897	-0.006712815	0.052977296	0.899169001	-0.110548315	0.097122684
T2DM	rs2303700	0.000856637	0.066329778	0.989695752	-0.129149727	0.130863001
T2DM	rs2383208	0.00840512	0.04315392	0.845572447	-0.076176563	0.092986803
T2DM	rs2781655	-0.114685901	0.069775587	0.100250722	-0.251446052	0.022074251
T2DM	rs28553330	-0.006112104	0.102992985	0.95267743	-0.207978355	0.195754147
T2DM	rs28642213	-0.01909512	0.047523606	0.687829134	-0.112241386	0.074051147
T2DM	rs2943656	-0.029563351	0.056155236	0.598570301	-0.139627613	0.080500911
T2DM	rs34872471	-0.041534296	0.014914342	0.005355149	-0.070766406	-0.012302186
T2DM	rs3887925	-0.166311987	0.068404598	0.015044819	-0.300384998	-0.032238975
T2DM	rs429358	-2.387503075	0.070223985	2.35E-253	-2.525142086	-2.249864064
T2DM	rs45551238	0.213388235	0.115236166	0.064062147	-0.012474649	0.43925112
T2DM	rs498475	-0.037493598	0.065592226	0.567581824	-0.16605436	0.091067165
T2DM	rs5215	-0.061939257	0.069753312	0.374553738	-0.198655748	0.074777234
T2DM	rs55993634	-0.095761847	0.046344957	0.038801666	-0.186597964	-0.00492573
T2DM	rs56348580	0.112744841	0.057261274	0.048958321	0.000512744	0.224976938

T2DM	rs6017317	-0.017197266	0.074098177	0.816470001	-0.162429693	0.128035161
T2DM	rs62137406	0.021673823	0.172229672	0.89985655	-0.315896334	0.35924398
T2DM	rs62492368	0.092922526	0.05885013	0.114343188	-0.022423729	0.208268781
T2DM	rs6780171	-0.037212766	0.047470426	0.433090144	-0.1302548	0.055829268
T2DM	rs6786846	0.002488458	0.058590859	0.966122604	-0.112349626	0.117326543
T2DM	rs7018475	-0.020410052	0.041320192	0.621342458	-0.101397629	0.060577524
T2DM	rs71330995	0.068637069	0.050239655	0.171878371	-0.029832655	0.167106793
T2DM	rs7224685	-0.01058809	0.069213975	0.878417054	-0.146247481	0.125071301
T2DM	rs73113806	-0.049576138	0.054914443	0.366638051	-0.157208446	0.05805617
T2DM	rs73541184	0.025630475	0.052991078	0.628616329	-0.078232037	0.129492987
T2DM	rs7451008	-0.0172975	0.036807188	0.638391474	-0.089439588	0.054844588
T2DM	rs745805	-0.125285542	0.065442289	0.055563557	-0.253552429	0.002981345
T2DM	rs74862545	-0.014279535	0.054579457	0.793607964	-0.121255271	0.092696202
T2DM	rs7507893	-0.058197054	0.069195417	0.400317914	-0.193820072	0.077425964
T2DM	rs76177300	-0.013199856	0.0670057	0.843830658	-0.144531027	0.118131316
T2DM	rs76895963	0.094599724	0.031728546	0.002868086	0.032411774	0.156787674
T2DM	rs77655131	-0.066353414	0.063515361	0.296169539	-0.190843522	0.058136695
T2DM	rs77735929	0.050791013	0.09605347	0.596959094	-0.137473788	0.239055813
T2DM	rs78470967	-0.003896591	0.043056884	0.927890944	-0.088288083	0.080494901
T2DM	rs7998259	0.072227438	0.056914434	0.204422133	-0.039324853	0.18377973
T2DM	rs8100204	-0.621986667	0.061497026	4.78E-24	-0.742520837	-0.501452496
T2DM	rs8353	0.009884126	0.062032392	0.873402469	-0.111699363	0.131467616
T2DM	rs878521	0.021304698	0.053438143	0.690129295	-0.083434063	0.126043459
T2DM	rs9505086	-0.001411011	0.066413438	0.983049527	-0.131581348	0.128759327
T2DM	rs9940128	-0.078742347	0.035596088	0.026959291	-0.14851068	-0.008974014
T2DM	All - Inverse variance weighted	-0.040047129	0.031606114	0.205130594	-0.101995112	0.021900854
T2DM	All - MR Egger	-0.015411765	0.068686659	0.823279962	-0.150037617	0.119214086
Fasting glucose	rs10305457	0.086665532	0.300865957	0.773305685	-0.503031745	0.676362809
Fasting glucose	rs1057394	0.367067742	0.341592742	0.282564104	-0.302454032	1.036589516
Fasting glucose	rs10811660	0.016926368	0.244501794	0.944808144	-0.462297148	0.496149883
Fasting glucose	rs10830963	-0.029251166	0.059977591	0.625761232	-0.146807244	0.088304912
Fasting glucose	rs10838524	-0.088786134	0.17502605	0.6119627	-0.431837193	0.254264924
Fasting glucose	rs10838693	0.208870056	0.249488136	0.402483335	-0.280126689	0.697866802
Fasting glucose	rs10974438	-0.399367677	0.218669192	0.067796733	-0.827959293	0.029223939
Fasting glucose	rs11603349	-0.052880932	0.241810169	0.826893259	-0.526828864	0.421067
Fasting glucose	rs11610045	0.483843056	0.286940972	0.091755145	-0.07856125	1.046247361
Fasting glucose	rs11619319	0.153002312	0.288174566	0.595462984	-0.411819838	0.717824462

Fasting glucose	rs11708067	0.279736299	0.172003203	0.103876593	-0.057389979	0.616862577
Fasting glucose	rs12055786	-0.024260583	0.351281667	0.944939433	-0.71277265	0.664251483
Fasting glucose	rs12541643	0.869788136	0.353012712	0.013743432	0.17788322	1.561693051
Fasting glucose	rs1260326	-1.635847518	0.149797163	9.21E-28	-1.929449957	-1.342245078
Fasting glucose	rs12784552	0.086291793	0.218320061	0.692655728	-0.341615526	0.514199112
Fasting glucose	rs12888855	0.057895407	0.365442963	0.874121687	-0.6583728	0.774163615
Fasting glucose	rs12898997	-2.006673469	0.449163265	7.91E-06	-2.887033469	-1.126313469
Fasting glucose	rs157512	0.628178358	0.362162687	0.082825892	-0.081660507	1.338017224
Fasting glucose	rs1604038	-0.070198485	0.23134798	0.761560317	-0.523640525	0.383243556
Fasting glucose	rs16851397	0.476345566	0.300277064	0.112659147	-0.11219748	1.064888612
Fasting glucose	rs16913693	-0.367870558	0.337286802	0.275415555	-1.02895269	0.293211574
Fasting glucose	rs17168486	-0.204077857	0.195444286	0.296404857	-0.587148657	0.178992943
Fasting glucose	rs17265513	0.892006329	0.327340506	0.006429943	0.250418937	1.533593722
Fasting glucose	rs17270243	0.613494231	0.467785577	0.189693624	-0.3033655	1.530353962
Fasting glucose	rs17437560	-0.28766	0.402708	0.475032979	-1.07696768	0.50164768
Fasting glucose	rs174583	1.880744048	0.257170833	2.61E-13	1.376689214	2.384798881
Fasting glucose	rs1820176	-0.10208502	0.185384211	0.581861774	-0.465438073	0.261268032
Fasting glucose	rs189548	0.687717886	0.373186179	0.065354148	-0.043727024	1.419162797
Fasting glucose	rs194518	0.624191176	0.407310784	0.125406726	-0.174137961	1.422520314
Fasting glucose	rs2075423	-0.061165714	0.27253913	0.822423422	-0.59534241	0.473010981
Fasting glucose	rs2238435	0.019649196	0.38010625	0.958772552	-0.725359054	0.764657446
Fasting glucose	rs2461385	-0.038121475	0.263658525	0.885037323	-0.554892184	0.478649235
Fasting glucose	rs2595701	0.298331746	0.235583598	0.205387109	-0.163412106	0.760075598
Fasting glucose	rs2657879	1.298663866	0.449021849	0.003825465	0.418581042	2.178746689
Fasting glucose	rs2839671	-0.21988125	0.338391875	0.515832474	-0.883129325	0.443366825
Fasting glucose	rs348330	-0.197534426	0.35247541	0.575192689	-0.88838623	0.493317377
Fasting glucose	rs35889227	0.344978462	0.330380769	0.29640006	-0.302567846	0.992524769
Fasting glucose	rs3778321	-0.031673871	0.290241398	0.913099853	-0.600547011	0.537199269
Fasting glucose	rs3829109	0.232406135	0.28706319	0.418170556	-0.330237718	0.795049988
Fasting glucose	rs3842753	0.235526866	0.341340299	0.490190393	-0.433500119	0.904553851
Fasting glucose	rs39713	0.243951479	0.424707692	0.565698139	-0.588475598	1.076378556
Fasting glucose	rs4760278	1.326318182	0.449601818	0.003177904	0.445098618	2.207537745
Fasting glucose	rs4862423	-0.321813008	0.345739837	0.351959473	-0.999463089	0.355837073
Fasting glucose	rs507666	3.300646341	0.325639634	3.83E-24	2.662392659	3.938900024
Fasting glucose	rs537183	0.0298546	0.065457014	0.648321845	-0.098441146	0.158150347
Fasting glucose	rs58925536	0.209522222	0.373588235	0.574908447	-0.522710719	0.941755163
Fasting glucose	rs6113722	-0.105657783	0.255415094	0.679115167	-0.606271368	0.394955802

Fasting glucose	rs6489811	-0.398075455	0.376662727	0.290580716	-1.1363344	0.340183491
Fasting glucose	rs6538804	0.192960563	0.301130986	0.521661636	-0.397256169	0.783177296
Fasting glucose	rs6598541	-0.305802632	0.379502632	0.420359073	-1.049627789	0.438022526
Fasting glucose	rs6662924	0.844531469	0.381092308	0.026685947	0.097590545	1.591472392
Fasting glucose	rs6808574	-0.286055118	0.335548031	0.393935943	-0.94372926	0.371619024
Fasting glucose	rs7012637	-0.796572222	0.231730556	0.000587125	-1.250764111	-0.342380333
Fasting glucose	rs7095788	-0.165958491	0.406411321	0.683015963	-0.962524679	0.630607698
Fasting glucose	rs7163757	-0.630271889	0.192641475	0.001068896	-1.00784918	-0.252694599
Fasting glucose	rs7178572	0.428238017	0.376929752	0.255905695	-0.310544298	1.167020331
Fasting glucose	rs7708285	0.070156692	0.336303759	0.834751857	-0.588998677	0.72931206
Fasting glucose	rs77981966	-0.430934959	0.317882927	0.17521359	-1.053985496	0.192115577
Fasting glucose	rs78132593	0.258502721	0.340988435	0.448392194	-0.409834612	0.926840054
Fasting glucose	rs7903146	-0.533471042	0.175325483	0.002344295	-0.877108988	-0.189833097
Fasting glucose	rs878521	0.034692896	0.08701949	0.690129295	-0.135865304	0.205251097
Fasting glucose	rs896854	-0.195724242	0.41749697	0.639209752	-1.014018303	0.622569818
Fasting glucose	rs9348441	-0.155525568	0.267434659	0.560872495	-0.6796975	0.368646364
Fasting glucose	rs9650069	0.139373077	0.157953147	0.377576895	-0.170215091	0.448961245
Fasting glucose	All - Inverse variance weighted	-0.00607092	0.066513807	0.92727561	-0.136437982	0.124296143
Fasting glucose	All - MR Egger	-0.121894595	0.120739376	0.316626252	-0.358543772	0.114754581
Fasting insulin	rs10050393	-0.012734778	0.463511111	0.978081206	-0.921216556	0.895747
Fasting insulin	rs10865959	-0.638313768	0.321321739	0.046975025	-1.268104377	-0.008523159
Fasting insulin	rs116141873	0.245172897	0.226224065	0.278470629	-0.198226271	0.688572065
Fasting insulin	rs11708067	-0.582265926	0.358021481	0.103876593	-1.28398803	0.119456178
Fasting insulin	rs11727676	0.320847291	0.344893596	0.352226616	-0.355144158	0.996838739
Fasting insulin	rs118164457	0.601771014	0.298846377	0.044046642	0.016032116	1.187509913
Fasting insulin	rs1206760	0.476500893	0.373491071	0.202025296	-0.255541607	1.208543393
Fasting insulin	rs12454712	0.50331831	0.299719718	0.093094249	-0.084132338	1.090768958
Fasting insulin	rs1260326	-1.997008658	0.182869264	9.21E-28	-2.355432416	-1.6385849
Fasting insulin	rs13258890	-0.2771875	0.377135156	0.46235054	-1.016372406	0.461997406
Fasting insulin	rs13389219	0.779025126	0.212747236	0.000250508	0.362040543	1.196009709
Fasting insulin	rs1351394	-0.183638739	0.373124324	0.622602694	-0.914962414	0.547684937
Fasting insulin	rs1474696	1.101	0.282298639	9.61E-05	0.547694667	1.654305333
Fasting insulin	rs17036126	-0.031059665	0.294559809	0.916023233	-0.60839689	0.54627756
Fasting insulin	rs17331151	1.995574074	0.402037654	6.92E-07	1.207580272	2.783567877
Fasting insulin	rs2108349	0.281177391	0.385170435	0.465385465	-0.473756661	1.036111443
Fasting insulin	rs2780215	-0.02956352	0.225596429	0.89573888	-0.47173252	0.41260548
Fasting insulin	rs2845885	-0.068231863	0.443845098	0.877823418	-0.938168255	0.801704529

Fasting insulin	rs2943646	-0.054626	0.172448	0.751419682	-0.39262408	0.28337208
Fasting insulin	rs35000407	-0.004588372	0.247373643	0.985201409	-0.489440713	0.480263969
Fasting insulin	rs3775380	0.076908487	0.348756303	0.825464529	-0.606653866	0.76047084
Fasting insulin	rs459193	0.503031492	0.262394475	0.055227628	-0.01126168	1.017324663
Fasting insulin	rs4865796	-0.139848485	0.27169697	0.606747099	-0.672374545	0.392677576
Fasting insulin	rs5017305	0.082710949	0.401161314	0.836651266	-0.703565226	0.868987124
Fasting insulin	rs62271373	0.392578125	0.346710156	0.257510494	-0.286973781	1.072130031
Fasting insulin	rs6487237	0.241448052	0.335184416	0.471313277	-0.415513403	0.898409506
Fasting insulin	rs6674544	0.356657062	0.239024859	0.135664082	-0.111831661	0.825145785
Fasting insulin	rs6855363	-0.5279288	0.35304	0.134815412	-1.2198872	0.1640296
Fasting insulin	rs6905288	0.781725	0.371749107	0.035480674	0.05309675	1.51035325
Fasting insulin	rs7012814	-0.659452055	0.190441096	0.000534663	-1.032716603	-0.286187507
Fasting insulin	rs7133378	1.349007874	0.3491	0.000111433	0.664771874	2.033243874
Fasting insulin	rs73013411	0.919461111	0.341329444	0.007065008	0.2504554	1.588466822
Fasting insulin	rs731839	-0.20804876	0.361538017	0.564983801	-0.916663273	0.500565752
Fasting insulin	rs75179845	-1.358712963	0.405705093	0.00081099	-2.153894944	-0.563530981
Fasting insulin	rs7903146	1.191112069	0.391459483	0.002344295	0.423851483	1.958372655
Fasting insulin	rs860598	-0.442533898	0.318189266	0.164289641	-1.066184859	0.181117062
Fasting insulin	rs972283	1.030914286	0.393406667	0.008780609	0.259837219	1.801991352
Fasting insulin	rs9884482	0.7270104	0.3426576	0.033864466	0.055401504	1.398619296
Fasting insulin	All - Inverse variance weighted	0.041802775	0.129660135	0.747147658	-0.212331091	0.29593664
Fasting insulin	All - MR Egger	-0.518832222	0.380564927	0.181247277	-1.264739479	0.227075034
Hypertension	rs10245376	0.401653137	0.982762451	0.682761235	-1.524561268	2.327867542
Hypertension	rs10749409	-0.289288288	0.94305843	0.75902954	-2.137682811	1.559106234
Hypertension	rs1077394	1.529935862	1.058524106	0.148360118	-0.544771385	3.604643109
Hypertension	rs10804330	-0.733518986	1.037683585	0.479640327	-2.767378813	1.300340841
Hypertension	rs11191559	-3.584283345	0.99764872	0.000327231	-5.539674837	-1.628891854
Hypertension	rs11604462	-1.599145833	0.984912886	0.104452257	-3.529575089	0.331283423
Hypertension	rs11801879	-0.723776426	0.958724185	0.450286743	-2.602875829	1.155322977
Hypertension	rs12258967	-2.063635083	0.899458792	0.02177284	-3.826574315	-0.30069585
Hypertension	rs12263737	-1.294438737	1.054016115	0.21940888	-3.360310322	0.771432848
Hypertension	rs12360772	-2.2563077	0.923369739	0.014543224	-4.066112388	-0.446503013
Hypertension	rs12656497	-0.739274161	0.782528911	0.344799594	-2.273030826	0.794482505
Hypertension	rs1275985	-1.477411782	0.686199439	0.031315885	-2.822362682	-0.132460882
Hypertension	rs12762222	-2.043387679	1.121147416	0.068366105	-4.240836614	0.154061256
Hypertension	rs12932686	-1.767281136	1.079990453	0.101758851	-3.884062424	0.349500153
Hypertension	rs13125101	-1.313437242	0.476798479	0.00587458	-2.247962261	-0.378912223

Hypertension	rs1327235	-1.343751675	0.96334316	0.163050912	-3.231904268	0.544400918
Hypertension	rs162395	-1.802704334	1.077070675	0.094187539	-3.913762856	0.308354189
Hypertension	rs167479	-0.572965121	0.711115122	0.420399899	-1.966750761	0.820820518
Hypertension	rs16948048	0.158526484	1.00075196	0.874135808	-1.802947358	2.120000326
Hypertension	rs17035646	-2.125776963	0.722349348	0.003251897	-3.541581685	-0.709972241
Hypertension	rs17558745	-2.056078909	1.121282691	0.066700812	-4.253792983	0.141635165
Hypertension	rs1918898	-1.396480136	1.092300013	0.201081389	-3.537388161	0.74442789
Hypertension	rs2003476	-0.644967318	1.057940865	0.542097647	-2.718531414	1.428596777
Hypertension	rs2643826	-1.80436486	0.842811992	0.032283188	-3.456276364	-0.152453356
Hypertension	rs2728624	-0.558664577	1.078325532	0.604398776	-2.67218262	1.554853467
Hypertension	rs2759315	-0.87265827	0.883880329	0.323493805	-2.605063715	0.859747175
Hypertension	rs3184504	-3.898113951	0.67987886	9.84E-09	-5.230676515	-2.565551386
Hypertension	rs346078	-2.101686599	1.104873344	0.057145424	-4.267238352	0.063865155
Hypertension	rs35443	-1.635902188	0.860097036	0.057171516	-3.321692379	0.049888003
Hypertension	rs35587371	-2.099189189	0.935995842	0.024914008	-3.93374104	-0.264637339
Hypertension	rs3735533	-0.772901791	0.906778937	0.3940145	-2.550188508	1.004384925
Hypertension	rs3790604	-2.187084009	0.88087397	0.013033344	-3.913596991	-0.460571027
Hypertension	rs3796581	-0.347395767	0.929016755	0.708449563	-2.168268607	1.473477072
Hypertension	rs3821843	-1.91037289	0.96991259	0.048880323	-3.811401567	-0.009344213
Hypertension	rs3918226	-1.825060312	0.757129634	0.01593071	-3.309034395	-0.341086229
Hypertension	rs4291	-1.85214375	1.096625603	0.091229373	-4.001529931	0.297242431
Hypertension	rs4412193	-5.143516949	0.910423729	1.61E-08	-6.927947458	-3.359086441
Hypertension	rs55670730	-2.711046696	1.112056241	0.014774154	-4.890676929	-0.531416463
Hypertension	rs55730499	-4.186920205	0.979969719	1.93E-05	-6.107660854	-2.266179555
Hypertension	rs56094641	-2.379926509	1.04524915	0.022792271	-4.428614842	-0.331238176
Hypertension	rs56273825	-0.83245914	1.093648686	0.446551947	-2.976010565	1.311092284
Hypertension	rs568546	-0.786724688	0.795054768	0.322407502	-2.345032034	0.771582658
Hypertension	rs57139556	0.185025213	1.035393799	0.858173011	-1.844346632	2.214397058
Hypertension	rs6026744	-1.435800036	0.769603707	0.062092372	-2.944223302	0.07262323
Hypertension	rs6031435	-2.209970106	0.996205935	0.02652847	-4.162533738	-0.257406474
Hypertension	rs6108171	-0.058372081	0.64214561	0.927570757	-1.316977477	1.200233315
Hypertension	rs62089932	0.70042921	1.099040702	0.523923187	-1.453690565	2.854548985
Hypertension	rs633185	-0.04718689	0.704685837	0.946612275	-1.42837113	1.33399735
Hypertension	rs6766859	-1.13854651	1.064545664	0.284838116	-3.225056011	0.947962991
Hypertension	rs68096471	-0.393540673	1.045270135	0.706547728	-2.442270137	1.655188792
Hypertension	rs6822044	-0.309025658	1.035861797	0.765453513	-2.339314781	1.721263464
Hypertension	rs6866614	-1.376080365	1.075702667	0.20081305	-3.484457593	0.732296863

Hypertension	rs6918911	0.698864332	0.963345216	0.468172478	-1.189292291	2.587020956
Hypertension	rs6961048	-1.602795971	1.072979443	0.135232951	-3.705835678	0.500243737
Hypertension	rs6991641	-1.129068853	0.911856824	0.215638768	-2.916308229	0.658170522
Hypertension	rs72831345	-0.09297168	0.609931728	0.878848073	-1.288437867	1.102494507
Hypertension	rs7297416	-1.508504906	1.128681409	0.181379475	-3.720720468	0.703710656
Hypertension	rs740746	-2.727678363	0.840660089	0.0011758	-4.375372138	-1.079984589
Hypertension	rs7497304	-0.898945544	0.755303414	0.233976419	-2.379340236	0.581449148
Hypertension	rs7528118	-2.50176998	1.110391954	0.024255918	-4.678138209	-0.325401751
Hypertension	rs76452347	-2.519790511	1.036711251	0.015075445	-4.551744563	-0.487836458
Hypertension	rs7685862	-1.218513156	1.079292039	0.258900787	-3.333925553	0.89689924
Hypertension	rs7700842	-1.074299967	0.617299927	0.081802685	-2.284207823	0.135607889
Hypertension	rs7763350	-0.904542588	0.985737427	0.358812419	-2.836587945	1.027502769
Hypertension	rs77924615	-1.873916622	1.01699511	0.065387228	-3.867227037	0.119393793
Hypertension	rs8042127	0.06853555	1.062338915	0.948561097	-2.013648724	2.150719823
Hypertension	rs8118848	1.821273682	0.99376239	0.066846396	-0.126500603	3.769047967
Hypertension	rs9330353	-1.707990213	0.913797408	0.061607156	-3.499033133	0.083052707
Hypertension	rs9375459	-2.327383608	0.670074564	0.000514061	-3.640729753	-1.014037464
Hypertension	All - Inverse variance weighted	-1.321880138	0.142201607	1.46E-20	-1.600595288	-1.043164987
Hypertension	All - MR Egger	-1.216678422	0.536600107	0.026597761	-2.268414632	-0.164942212
HDL cholesterol	rs10031010	0.177072485	0.386639919	0.646968417	-0.580741757	0.934886728
HDL cholesterol	rs10053349	0.016798644	0.352803758	0.962023319	-0.674696721	0.708294009
HDL cholesterol	rs10108282	0.121819315	0.302795431	0.687451802	-0.47165973	0.715298359
HDL cholesterol	rs10162642	0.094390503	0.106502609	0.375469849	-0.114354612	0.303135617
HDL cholesterol	rs10233430	0.129470002	0.204002557	0.525657417	-0.27037501	0.529315013
HDL cholesterol	rs1045241	0.031861596	0.284792077	0.910921177	-0.526330875	0.590054066
HDL cholesterol	rs1047891	-0.275392044	0.235430231	0.24210581	-0.736835296	0.186051208
HDL cholesterol	rs10504477	0.505641805	0.279321789	0.070257284	-0.041828901	1.053112511
HDL cholesterol	rs10513801	0.134660513	0.198174236	0.496817529	-0.253760989	0.523082014
HDL cholesterol	rs1055582	0.031990645	0.29487419	0.9136077	-0.545962767	0.609944057
HDL cholesterol	rs10750766	0.427047392	0.245305204	0.081704279	-0.053750807	0.907845591
HDL cholesterol	rs10774439	0.212934237	0.263017111	0.418179762	-0.302579301	0.728447775
HDL cholesterol	rs10786114	0.361633192	0.262558485	0.168406109	-0.15298144	0.876247823
HDL cholesterol	rs1083470	0.047680978	0.368365435	0.897010022	-0.674315275	0.769677231
HDL cholesterol	rs11009262	-0.310711607	0.380601743	0.414288907	-1.056691023	0.435267809
HDL cholesterol	rs11021232	0.29392692	0.32216719	0.361588285	-0.337520773	0.925374613
HDL cholesterol	rs11045171	-0.120376083	0.184073595	0.513139975	-0.481160329	0.240408163
HDL cholesterol	rs111363680	-1.056388285	0.388620567	0.00656178	-1.818084596	-0.294691975

HDL cholesterol	rs11171710	-0.219626136	0.364866514	0.547217022	-0.934764504	0.495512233
HDL cholesterol	rs112001035	-0.427444328	0.190662315	0.02496835	-0.801142465	-0.053746191
HDL cholesterol	rs11218738	0.657690044	0.203604295	0.001236878	0.258625626	1.056754463
HDL cholesterol	rs112233856	-0.030624483	0.240890079	0.89883709	-0.502769037	0.441520072
HDL cholesterol	rs112350227	1.333541028	0.398996177	0.000831051	0.551508521	2.115573535
HDL cholesterol	rs11239536	0.187504043	0.168419588	0.265573193	-0.142598349	0.517606436
HDL cholesterol	rs11254464	0.788651915	0.328568517	0.016383152	0.144657621	1.432646209
HDL cholesterol	rs1132274	-0.260506682	0.262031872	0.320135548	-0.774089152	0.253075787
HDL cholesterol	rs113740515	0.126210583	0.134523709	0.348139961	-0.137455886	0.389877052
HDL cholesterol	rs11381821	-0.081867921	0.225327855	0.716359871	-0.523510517	0.359774674
HDL cholesterol	rs113851927	0.281775272	0.39624689	0.477015845	-0.494868632	1.058419177
HDL cholesterol	rs113966472	-0.05335423	0.374395339	0.886678973	-0.787169096	0.680460635
HDL cholesterol	rs114165349	-0.614468385	0.171062242	0.000328056	-0.94975038	-0.279186391
HDL cholesterol	rs11429307	-0.594720678	0.171030607	0.000506536	-0.929940669	-0.259500688
HDL cholesterol	rs11456863	0.643919239	0.228336926	0.004801768	0.196378863	1.091459614
HDL cholesterol	rs115912456	-0.53487395	0.371834497	0.150299843	-1.263669563	0.193921664
HDL cholesterol	rs116006942	-0.091884165	0.293608972	0.754320624	-0.66735775	0.483589421
HDL cholesterol	rs11614202	0.694724407	0.249111366	0.0052902	0.20646613	1.182982684
HDL cholesterol	rs11631178	0.105239726	0.319780854	0.742080465	-0.521530748	0.7320102
HDL cholesterol	rs11640494	0.137711252	0.271375985	0.611835213	-0.39418568	0.669608183
HDL cholesterol	rs11664369	-0.062942766	0.204536137	0.758284588	-0.463833595	0.337948063
HDL cholesterol	rs1168114	2.015730699	0.279184501	5.20E-13	1.468529077	2.562932321
HDL cholesterol	rs116843064	-0.052957639	0.071730738	0.460342034	-0.193549884	0.087634607
HDL cholesterol	rs116857878	-0.336106589	0.319141885	0.29226893	-0.961624685	0.289411506
HDL cholesterol	rs11688682	0.446339461	0.326023791	0.170986915	-0.192667169	1.08534609
HDL cholesterol	rs11704977	0.069889917	0.395284307	0.859658232	-0.704867324	0.844647158
HDL cholesterol	rs117230571	0.518454153	0.296214206	0.080072134	-0.06212569	1.099033996
HDL cholesterol	rs117291242	-0.698786371	0.348103269	0.044705907	-1.381068779	-0.016503963
HDL cholesterol	rs117762989	0.33447418	0.37742774	0.375513022	-0.405284189	1.07423255
HDL cholesterol	rs117847213	0.163699258	0.356234501	0.645855997	-0.534520364	0.861918881
HDL cholesterol	rs12045101	0.585835527	0.331967647	0.077608009	-0.064821061	1.236492114
HDL cholesterol	rs12046972	-0.491437998	0.281239837	0.080568282	-1.042668079	0.059792083
HDL cholesterol	rs12205778	-0.545122319	0.291086181	0.061107342	-1.115651234	0.025406596
HDL cholesterol	rs12229011	-0.515748289	0.268773107	0.054997191	-1.042543579	0.011047001
HDL cholesterol	rs1225053	-0.04503857	0.31361188	0.88580651	-0.659717854	0.569640714
HDL cholesterol	rs1240820	-0.244761255	0.345789813	0.479049305	-0.922509288	0.432986779
HDL cholesterol	rs12411732	-0.064656173	0.194729854	0.739866529	-0.446326687	0.31701434

HDL cholesterol	rs12462109	0.224824024	0.285808486	0.431501575	-0.335360608	0.785008656
HDL cholesterol	rs12475332	0.00555171	0.363882298	0.987827241	-0.707657595	0.718761014
HDL cholesterol	rs12575456	0.088251627	0.099071694	0.373044263	-0.105928893	0.282432148
HDL cholesterol	rs12686780	-0.409793553	0.336960071	0.223928281	-1.070235293	0.250648187
HDL cholesterol	rs1270076	0.483871035	0.346511677	0.162592225	-0.195291851	1.163033921
HDL cholesterol	rs12705595	0.289594225	0.389248693	0.456887047	-0.473333213	1.052521664
HDL cholesterol	rs12740374	-4.23470961	0.172294191	2.15E-133	-4.572406224	-3.897012996
HDL cholesterol	rs12740811	-0.151271046	0.386688335	0.695652142	-0.909180182	0.60663809
HDL cholesterol	rs12781812	-0.001450762	0.393461937	0.997058069	-0.772636158	0.769734634
HDL cholesterol	rs12921195	0.1275126	0.37046126	0.730696219	-0.59859147	0.853616669
HDL cholesterol	rs12926854	0.142010665	0.382820909	0.710668452	-0.608318316	0.892339646
HDL cholesterol	rs12928099	0.282593355	0.212129019	0.182802103	-0.133179522	0.698366232
HDL cholesterol	rs12986742	0.73025509	0.391715251	0.062286629	-0.037506802	1.498016981
HDL cholesterol	rs12998038	-0.298208736	0.358879001	0.406004662	-1.001611577	0.405194105
HDL cholesterol	rs13066793	0.037919454	0.327224837	0.907746096	-0.603441226	0.679280134
HDL cholesterol	rs13087167	0.134684518	0.257219721	0.600545227	-0.369466135	0.638835171
HDL cholesterol	rs13097947	-0.31224927	0.276389115	0.258583614	-0.853971936	0.229473395
HDL cholesterol	rs13107325	0.032389914	0.098095114	0.741257265	-0.159876509	0.224656336
HDL cholesterol	rs13111599	-0.277773001	0.367567483	0.449825407	-0.998205267	0.442659266
HDL cholesterol	rs13137144	-0.45577883	0.255063904	0.073950395	-0.955704083	0.044146423
HDL cholesterol	rs13144151	0.202826044	0.328103752	0.536458969	-0.44025731	0.845909399
HDL cholesterol	rs13235365	0.448964099	0.179909831	0.01257815	0.096340831	0.801587367
HDL cholesterol	rs13269725	-0.252206901	0.296357229	0.394756421	-0.833067069	0.328653267
HDL cholesterol	rs13379043	-0.830552816	0.235816345	0.000428252	-1.292752853	-0.36835278
HDL cholesterol	rs13389219	-0.559006793	0.152661508	0.000250508	-0.858223349	-0.259790238
HDL cholesterol	rs13402475	0.122685343	0.21806586	0.573702637	-0.304723742	0.550094428
HDL cholesterol	rs1349852	0.083885683	0.373186654	0.822148785	-0.647560159	0.815331526
HDL cholesterol	rs138354839	0.372586821	0.304717779	0.22143262	-0.224660025	0.969833668
HDL cholesterol	rs1383732	0.024645582	0.382990844	0.948691289	-0.726016471	0.775307636
HDL cholesterol	rs1395221	0.072006828	0.379262298	0.849418655	-0.671347276	0.815360932
HDL cholesterol	rs140064750	-0.564087377	0.313876332	0.072309521	-1.179284988	0.051110234
HDL cholesterol	rs140164052	0.303468894	0.283651094	0.28467926	-0.25248725	0.859425037
HDL cholesterol	rs140584594	-0.471455698	0.14895948	0.001550867	-0.763416279	-0.179495118
HDL cholesterol	rs141062196	-0.030655678	0.277232927	0.911951523	-0.574032214	0.512720858
HDL cholesterol	rs1411432	-1.114992451	0.380511888	0.003386966	-1.860795753	-0.36918915
HDL cholesterol	rs1412234	0.579701393	0.368195012	0.115385488	-0.141960831	1.301363617
HDL cholesterol	rs141440048	-0.443296892	0.376169679	0.238617536	-1.180589463	0.293995678

HDL cholesterol	rs141469619	-0.239177016	0.107340315	0.025866358	-0.449564034	-0.028789998
HDL cholesterol	rs142288236	-0.534685425	0.222454178	0.016235611	-0.970695613	-0.098675237
HDL cholesterol	rs1431659	0.046105317	0.365649088	0.899659332	-0.670566894	0.762777529
HDL cholesterol	rs144033177	0.168223322	0.308234659	0.585228303	-0.43591661	0.772363254
HDL cholesterol	rs144311893	-5.996665082	0.177409015	1.88E-250	-6.344386752	-5.648943411
HDL cholesterol	rs1446585	0.56637311	0.281007228	0.043851153	0.015598944	1.117147277
HDL cholesterol	rs145947882	-0.008985722	0.079794464	0.910339091	-0.165382872	0.147411429
HDL cholesterol	rs1471251	-0.630154599	0.219061561	0.004019725	-1.059515259	-0.20079394
HDL cholesterol	rs147460434	0.210102472	0.357658208	0.556908734	-0.490907615	0.91111256
HDL cholesterol	rs147627829	-0.134663461	0.188962284	0.476064035	-0.505029537	0.235702615
HDL cholesterol	rs147772065	-0.242011707	0.393623669	0.538666884	-1.013514099	0.529490685
HDL cholesterol	rs150224153	0.244946715	0.134674742	0.068941615	-0.01901578	0.508909209
HDL cholesterol	rs150237291	0.227992884	0.313668449	0.467312045	-0.386797275	0.842783043
HDL cholesterol	rs150844304	-0.085577902	0.144450703	0.553558176	-0.36870128	0.197545476
HDL cholesterol	rs150861794	0.543366788	0.373067578	0.145258996	-0.187845666	1.274579241
HDL cholesterol	rs1534696	-0.148990533	0.248322357	0.548513969	-0.635702353	0.337721287
HDL cholesterol	rs1601934	-0.105027717	0.044474757	0.018200535	-0.192198242	-0.017857193
HDL cholesterol	rs16928809	0.3895017	0.272996158	0.153647287	-0.14557077	0.92457417
HDL cholesterol	rs17124112	-0.35253534	0.368127224	0.338241601	-1.074064699	0.368994019
HDL cholesterol	rs17138358	0.150424943	0.15506589	0.33201104	-0.153504202	0.454354087
HDL cholesterol	rs17309930	0.361180985	0.232981246	0.12107966	-0.095462256	0.817824227
HDL cholesterol	rs17326656	-0.221376098	0.21802182	0.309922314	-0.648698865	0.205946669
HDL cholesterol	rs174566	0.574753531	0.077014661	8.46E-14	0.423804795	0.725702266
HDL cholesterol	rs1760940	0.393299176	0.395100623	0.319522051	-0.381098045	1.167696398
HDL cholesterol	rs17713879	0.231926653	0.308557385	0.452262552	-0.372845822	0.836699128
HDL cholesterol	rs1771582	0.721644085	0.331552318	0.029513284	0.071801542	1.371486629
HDL cholesterol	rs183906992	-0.20180757	0.351074051	0.565406904	-0.889912711	0.48629757
HDL cholesterol	rs185073199	-0.229451759	0.341069818	0.501111876	-0.897948603	0.439045084
HDL cholesterol	rs1862205	0.755803721	0.372384394	0.04239384	0.025930309	1.485677133
HDL cholesterol	rs188502504	0.021242307	0.34828796	0.951366661	-0.661402095	0.703886708
HDL cholesterol	rs1955512	-0.374382194	0.387766625	0.334302805	-1.134404779	0.385640391
HDL cholesterol	rs1970811	0.151371951	0.357731328	0.672190026	-0.549781452	0.852525355
HDL cholesterol	rs201441	0.058167354	0.382711175	0.879196715	-0.69194655	0.808281257
HDL cholesterol	rs201639483	0.195722437	0.39320081	0.618648189	-0.57495115	0.966396024
HDL cholesterol	rs2066714	0.320864382	0.132021117	0.015082086	0.062102992	0.579625772
HDL cholesterol	rs2068888	-0.716354802	0.216568041	0.000940458	-1.140828162	-0.291881441
HDL cholesterol	rs2098368	-0.202168423	0.360790837	0.575242094	-0.909318463	0.504981617

HDL cholesterol	rs2098918	0.345026875	0.349890478	0.32408422	-0.340758463	1.030812213
HDL cholesterol	rs2111216	0.010997154	0.197900111	0.955684988	-0.376887064	0.398881372
HDL cholesterol	rs2155220	-0.188222881	0.395133265	0.633822976	-0.96268408	0.586238318
HDL cholesterol	rs2159607	-0.362368138	0.220497883	0.100298402	-0.794543988	0.069807713
HDL cholesterol	rs2196808	0.200360702	0.365546728	0.583614712	-0.516110885	0.916832289
HDL cholesterol	rs2236464	0.505658513	0.325891963	0.120754626	-0.133089735	1.144406761
HDL cholesterol	rs2237035	0.352326617	0.305940132	0.24947747	-0.247316041	0.951969275
HDL cholesterol	rs2247355	0.026120414	0.259704179	0.919885805	-0.482899776	0.535140605
HDL cholesterol	rs2256609	0.200082705	0.160122354	0.21145983	-0.11375711	0.513922519
HDL cholesterol	rs2268840	0.266810049	0.28428206	0.347967036	-0.29038279	0.824002887
HDL cholesterol	rs2271308	0.171163668	0.167531061	0.306930871	-0.157197211	0.499524547
HDL cholesterol	rs2281718	-0.395286295	0.071122561	2.73E-08	-0.534686514	-0.255886076
HDL cholesterol	rs2290866	-0.876914431	0.399029574	0.027976365	-1.659012397	-0.094816466
HDL cholesterol	rs2297409	0.304510365	0.156219927	0.051266591	-0.001680692	0.610701423
HDL cholesterol	rs2298214	0.066750363	0.339899275	0.844310578	-0.599452216	0.732952941
HDL cholesterol	rs2298624	0.03710535	0.204096404	0.855737286	-0.362923602	0.437134302
HDL cholesterol	rs2298632	0.071709231	0.29191415	0.805951801	-0.500442503	0.643860966
HDL cholesterol	rs2302263	0.05064563	0.200690227	0.800764956	-0.342707215	0.443998474
HDL cholesterol	rs2307111	1.655617117	0.222530557	1.01E-13	1.219457225	2.09177701
HDL cholesterol	rs2339234	0.466577868	0.374357121	0.212638017	-0.267162089	1.200317826
HDL cholesterol	rs235314	-0.122992814	0.233413555	0.598241577	-0.580483382	0.334497754
HDL cholesterol	rs2362541	0.387319861	0.379505089	0.307447777	-0.356510114	1.131149835
HDL cholesterol	rs2364723	-0.290733821	0.36826532	0.429838311	-1.012533847	0.431066206
HDL cholesterol	rs2417125	0.087938323	0.350079833	0.801663307	-0.59821815	0.774094796
HDL cholesterol	rs2435307	-0.329354845	0.254911008	0.1963438	-0.82898042	0.170270731
HDL cholesterol	rs2498786	-0.041820123	0.167171755	0.802461596	-0.369476763	0.285836516
HDL cholesterol	rs2516331	-0.018160524	0.336716995	0.956987679	-0.678125833	0.641804786
HDL cholesterol	rs2520096	-0.0757549	0.317630515	0.811493262	-0.69831071	0.54680091
HDL cholesterol	rs254562	-0.007971861	0.366153967	0.982629924	-0.725633636	0.709689914
HDL cholesterol	rs2586116	0.685465964	0.290068706	0.018122002	0.1169313	1.254000627
HDL cholesterol	rs2642438	0.431523492	0.163694578	0.008385261	0.110682118	0.752364866
HDL cholesterol	rs2645979	-0.30350977	0.377350566	0.421213471	-1.043116879	0.436097339
HDL cholesterol	rs267738	-0.182989712	0.23251868	0.431288133	-0.638726326	0.272746901
HDL cholesterol	rs2723065	-0.024203832	0.284773063	0.932266634	-0.582359036	0.533951371
HDL cholesterol	rs2726111	-0.620003781	0.297047128	0.036867926	-1.202216152	-0.037791409
HDL cholesterol	rs2740488	0.387851686	0.068370975	1.41E-08	0.253844574	0.521858797
HDL cholesterol	rs2750411	0.122257276	0.382790462	0.74943509	-0.628012029	0.872526581

HDL cholesterol	rs2792751	0.219402324	0.128175225	0.086945078	-0.031821118	0.470625765
HDL cholesterol	rs2800710	-0.80478009	0.20463503	8.40E-05	-1.205864748	-0.403695432
HDL cholesterol	rs2804894	-0.232392828	0.273172197	0.394925366	-0.767810335	0.303024679
HDL cholesterol	rs2814982	0.716360197	0.24676594	0.00369613	0.232698954	1.20002144
HDL cholesterol	rs28362901	0.1139369	0.307105751	0.710635915	-0.487990372	0.715864172
HDL cholesterol	rs28510484	-0.160333912	0.357063416	0.653406529	-0.860178207	0.539510384
HDL cholesterol	rs286965	-0.86669251	0.319027739	0.006594268	-1.491986879	-0.241398141
HDL cholesterol	rs28746806	1.075690174	0.266132209	5.30E-05	0.554071044	1.597309305
HDL cholesterol	rs2910949	0.612230216	0.32318502	0.058176651	-0.021212424	1.245672856
HDL cholesterol	rs2925979	-0.039918496	0.120760342	0.740977129	-0.276608767	0.196771774
HDL cholesterol	rs2943645	0.033252359	0.099190947	0.7374472	-0.161161898	0.227666616
HDL cholesterol	rs2963468	-0.24949327	0.250108567	0.318502526	-0.739706061	0.240719521
HDL cholesterol	rs2965169	-6.317203367	0.350090622	8.72E-73	-7.003380987	-5.631025747
HDL cholesterol	rs3027167	0.164835262	0.357210803	0.644475109	-0.535297912	0.864968437
HDL cholesterol	rs308	-0.235670698	0.116399494	0.042901143	-0.463813706	-0.00752769
HDL cholesterol	rs3184504	0.894832829	0.156069815	9.84E-09	0.588935992	1.200729666
HDL cholesterol	rs32578	0.073272939	0.336735843	0.827742591	-0.586729313	0.73327519
HDL cholesterol	rs330089	0.83149317	0.338457292	0.01402138	0.168116878	1.494869462
HDL cholesterol	rs34045894	-0.873546201	0.328822097	0.007893488	-1.518037511	-0.22905489
HDL cholesterol	rs34073570	-0.62270722	0.38143223	0.102563656	-1.37031439	0.124899951
HDL cholesterol	rs34138141	-0.598694943	0.262662134	0.022647171	-1.113512725	-0.08387716
HDL cholesterol	rs343	-0.163061191	0.056434391	0.003859851	-0.273672598	-0.052449784
HDL cholesterol	rs34518086	-0.300462258	0.137332281	0.028680622	-0.569633528	-0.031290987
HDL cholesterol	rs34940374	-0.589753372	0.314484264	0.060751013	-1.20614253	0.026635786
HDL cholesterol	rs35493868	-0.666224326	0.138316562	1.46E-06	-0.937324787	-0.395123864
HDL cholesterol	rs35980001	-0.121736385	0.042847029	0.004494607	-0.205716562	-0.037756209
HDL cholesterol	rs36057735	0.57240117	0.171311264	0.000833891	0.236631093	0.908171247
HDL cholesterol	rs367070	0.160218352	0.118525945	0.176452857	-0.072092499	0.392529203
HDL cholesterol	rs367677	-0.627163278	0.292974332	0.032300132	-1.201392968	-0.052933588
HDL cholesterol	rs3732356	0.445579983	0.279382729	0.110740629	-0.102010166	0.993170132
HDL cholesterol	rs3745683	-0.10094719	0.1432054	0.480865068	-0.381629774	0.179735395
HDL cholesterol	rs3746915	-0.451216307	0.385894149	0.242292929	-1.207568838	0.305136225
HDL cholesterol	rs3747973	-0.238758338	0.29761056	0.422407841	-0.822075036	0.34455836
HDL cholesterol	rs3768321	0.158778543	0.115191158	0.168082365	-0.066996126	0.384553213
HDL cholesterol	rs3794752	-0.402961987	0.3610305	0.264360547	-1.110581768	0.304657793
HDL cholesterol	rs3814883	0.192704937	0.271327474	0.477561463	-0.339096912	0.724506786
HDL cholesterol	rs3924313	-0.315896775	0.185975549	0.089395886	-0.680408851	0.0486153

HDL cholesterol	rs4074448	0.121050823	0.28225233	0.668014569	-0.432163743	0.674265389
HDL cholesterol	rs41272086	0.126246201	0.118325391	0.285998519	-0.105671566	0.358163967
HDL cholesterol	rs42125	-1.320422809	0.38219366	0.000550593	-2.069522383	-0.571323235
HDL cholesterol	rs429358	-2.565633121	0.075463351	2.35E-253	-2.713541289	-2.417724953
HDL cholesterol	rs4330777	-0.38416388	0.210157779	0.067552794	-0.796073128	0.027745368
HDL cholesterol	rs454968	-0.556805967	0.391765228	0.155236945	-1.324665815	0.21105388
HDL cholesterol	rs4599108	-0.330456481	0.30629473	0.280639445	-0.930794152	0.269881189
HDL cholesterol	rs460428	0.003676779	0.343302756	0.991454806	-0.669196623	0.676550181
HDL cholesterol	rs4614	0.167879607	0.236074	0.477002883	-0.294825432	0.630584647
HDL cholesterol	rs4650994	-0.281334736	0.228691294	0.218624337	-0.729569672	0.166900199
HDL cholesterol	rs4691379	0.670262894	0.373392185	0.072643493	-0.061585788	1.402111576
HDL cholesterol	rs4784709	0.019343079	0.141513348	0.891277958	-0.258023082	0.296709241
HDL cholesterol	rs4803773	-0.882486042	0.107556518	2.31E-16	-1.093296817	-0.671675267
HDL cholesterol	rs4804101	-0.083573309	0.299656938	0.780324159	-0.670900908	0.50375429
HDL cholesterol	rs4855582	0.78857432	0.374964174	0.035460021	0.053644538	1.523504102
HDL cholesterol	rs4871603	-0.749267485	0.120859799	5.67E-10	-0.986152691	-0.51238228
HDL cholesterol	rs4871624	-0.382609718	0.225240008	0.089380313	-0.824080133	0.058860697
HDL cholesterol	rs4875043	-0.311184347	0.338178904	0.35748043	-0.974014999	0.351646305
HDL cholesterol	rs4899251	-0.424474678	0.38474669	0.269915351	-1.178578191	0.329628835
HDL cholesterol	rs4917675	-0.406769674	0.326504601	0.21282631	-1.046718692	0.233179343
HDL cholesterol	rs4930352	-0.339331144	0.260679987	0.193013705	-0.850263919	0.17160163
HDL cholesterol	rs4969141	0.278259828	0.139931438	0.046751552	0.00399421	0.552525446
HDL cholesterol	rs532436	2.326646426	0.230491002	5.85E-24	1.874884062	2.77840879
HDL cholesterol	rs549058	-0.332740875	0.371017856	0.369808297	-1.059935872	0.394454122
HDL cholesterol	rs554146	-0.24943068	0.360021846	0.488421919	-0.955073497	0.456212138
HDL cholesterol	rs55781197	-0.057905606	0.110397566	0.5999177	-0.274284836	0.158473623
HDL cholesterol	rs557933	2.077548665	0.271600031	2.02E-14	1.545212603	2.609884726
HDL cholesterol	rs55935382	0.483856009	0.250828798	0.05372727	-0.007768435	0.975480454
HDL cholesterol	rs559355	0.041453032	0.162753249	0.798955863	-0.277543335	0.360449399
HDL cholesterol	rs56017932	-0.123566583	0.375774375	0.742282788	-0.860084357	0.612951192
HDL cholesterol	rs564832	-0.332898018	0.381864537	0.383333905	-1.081352511	0.415556474
HDL cholesterol	rs57512892	0.594346952	0.23299327	0.01074393	0.137680143	1.051013761
HDL cholesterol	rs57760538	-0.041129282	0.394926369	0.917054933	-0.815184964	0.7329264
HDL cholesterol	rs58123204	-0.694572367	0.312543478	0.026261527	-1.307157584	-0.081987149
HDL cholesterol	rs58298943	0.179740675	0.36872407	0.625927694	-0.542958502	0.902439852
HDL cholesterol	rs59037995	0.182178281	0.322753194	0.572447412	-0.450417979	0.814774541
HDL cholesterol	rs59104589	-0.504365489	0.28700343	0.078858163	-1.066892211	0.058161233

HDL cholesterol	rs59781045	0.039639234	0.111226414	0.721553547	-0.178364537	0.257643006
HDL cholesterol	rs6018652	-0.283035511	0.197258081	0.151330203	-0.66966135	0.103590328
HDL cholesterol	rs6059958	0.426348257	0.377738653	0.259030398	-0.314019503	1.166716018
HDL cholesterol	rs6066148	0.076457288	0.355134311	0.829540299	-0.619605961	0.772520537
HDL cholesterol	rs6073958	-0.705582973	0.085141778	1.16E-16	-0.872460857	-0.538705089
HDL cholesterol	rs6075860	0.005855094	0.3259125	0.98566659	-0.632933406	0.644643594
HDL cholesterol	rs6123685	-0.061146618	0.297475952	0.837141293	-0.644199483	0.521906248
HDL cholesterol	rs61352607	-0.447448801	0.157700311	0.004549168	-0.756541411	-0.138356191
HDL cholesterol	rs6142206	0.035319541	0.260279567	0.892059651	-0.474828411	0.545467493
HDL cholesterol	rs61435086	-0.355421242	0.208147253	0.087720408	-0.763389857	0.052547374
HDL cholesterol	rs61596977	0.02921245	0.367982126	0.936725991	-0.692032518	0.750457418
HDL cholesterol	rs61748951	0.145782439	0.381748264	0.702549576	-0.602444159	0.894009036
HDL cholesterol	rs61805075	-0.484040547	0.170774507	0.004591411	-0.81875858	-0.149322514
HDL cholesterol	rs61884005	-0.570954555	0.390100119	0.143300454	-1.335550788	0.193641678
HDL cholesterol	rs62102718	0.038759754	0.19328812	0.841067363	-0.340084962	0.41760447
HDL cholesterol	rs62117487	-0.316059254	0.194626295	0.104390997	-0.697526793	0.065408285
HDL cholesterol	rs62246443	-0.45473786	0.369014685	0.217835963	-1.178006642	0.268530922
HDL cholesterol	rs62271373	-0.247130945	0.218256706	0.257510494	-0.674914089	0.180652199
HDL cholesterol	rs62331150	-0.723493036	0.395309267	0.067220788	-1.4982992	0.051313127
HDL cholesterol	rs62428831	0.333636073	0.335181952	0.319547622	-0.323320554	0.990592699
HDL cholesterol	rs635769	-0.630555795	0.21701739	0.003666033	-1.055909879	-0.205201711
HDL cholesterol	rs6460894	0.522936549	0.35995742	0.146286692	-0.182579994	1.228453092
HDL cholesterol	rs6469605	-0.291139068	0.132493351	0.027992872	-0.550826036	-0.031452101
HDL cholesterol	rs6693842	0.784499415	0.317262881	0.013409253	0.162664169	1.40633466
HDL cholesterol	rs67016280	-0.265085222	0.376712723	0.481632057	-1.003442159	0.473271715
HDL cholesterol	rs6705285	-0.116885225	0.362479497	0.747104056	-0.827345038	0.593574589
HDL cholesterol	rs676210	-0.91472328	0.085987204	1.99E-26	-1.083258201	-0.74618836
HDL cholesterol	rs6762415	-0.053554752	0.380572483	0.888089915	-0.799476818	0.692367314
HDL cholesterol	rs6765484	0.353405534	0.184193209	0.05502642	-0.007613156	0.714424223
HDL cholesterol	rs680321	-0.046271644	0.382916041	0.903817623	-0.796787084	0.704243796
HDL cholesterol	rs6824451	-0.029814362	0.207119694	0.885541927	-0.435768963	0.376140239
HDL cholesterol	rs686030	0.093774097	0.119460261	0.432464404	-0.140368015	0.327916209
HDL cholesterol	rs689183	-0.158900302	0.303398021	0.600462549	-0.753560424	0.43575982
HDL cholesterol	rs6934962	0.828912016	0.260383558	0.001455429	0.318560242	1.33926379
HDL cholesterol	rs6939861	0.018137943	0.335378103	0.956869776	-0.639203139	0.675479024
HDL cholesterol	rs7036107	0.099479286	0.343741634	0.772274278	-0.574254318	0.773212889
HDL cholesterol	rs703966	-0.417538818	0.267597118	0.118682793	-0.942029169	0.106951532

HDL cholesterol	rs71571682	-0.218190114	0.277158866	0.431142297	-0.761421491	0.325041263
HDL cholesterol	rs7158166	0.135844946	0.299852411	0.650520395	-0.451865779	0.723555672
HDL cholesterol	rs71603401	-0.81584271	0.392671325	0.037739464	-1.585478506	-0.046206914
HDL cholesterol	rs71647892	0.636568167	0.303138972	0.035735712	0.042415782	1.230720553
HDL cholesterol	rs7170463	-0.158290413	0.236700862	0.50366383	-0.622224102	0.305643276
HDL cholesterol	rs7186799	-0.435757972	0.189770204	0.021662151	-0.807707572	-0.063808372
HDL cholesterol	rs7218647	-0.277405708	0.383117692	0.469020067	-1.028316384	0.473504968
HDL cholesterol	rs7251640	0.975102536	0.370370396	0.008469002	0.249176558	1.701028513
HDL cholesterol	rs72647336	-0.795761123	0.218712173	0.000274343	-1.224436982	-0.367085263
HDL cholesterol	rs7281183	-0.427824686	0.372449686	0.250688863	-1.157826069	0.302176698
HDL cholesterol	rs72926946	0.823856897	0.219011933	0.000168763	0.394593508	1.253120287
HDL cholesterol	rs72964564	0.535797786	0.381784203	0.16049616	-0.212499252	1.284094825
HDL cholesterol	rs7305678	-0.928587758	0.368450469	0.011727109	-1.650750677	-0.20642484
HDL cholesterol	rs73151974	-0.079693011	0.366305939	0.827773036	-0.797652651	0.63826663
HDL cholesterol	rs7316878	-0.203695592	0.381236963	0.593132754	-0.950920039	0.543528856
HDL cholesterol	rs73243877	-0.205752228	0.218776237	0.34697714	-0.634553653	0.223049197
HDL cholesterol	rs73455693	0.338440954	0.396592148	0.393452597	-0.438879656	1.115761564
HDL cholesterol	rs74500135	0.232047922	0.325460151	0.475855579	-0.405853974	0.869949818
HDL cholesterol	rs7488780	-0.154649076	0.353784228	0.662018349	-0.848066163	0.53876801
HDL cholesterol	rs75032664	0.344422647	0.362002101	0.341381949	-0.365101472	1.053946766
HDL cholesterol	rs75152587	-0.213037083	0.200131104	0.287107858	-0.605294046	0.17921988
HDL cholesterol	rs75246752	0.012992055	0.373031127	0.972216622	-0.718148954	0.744133065
HDL cholesterol	rs75479205	0.09220469	0.379556198	0.808061457	-0.651725459	0.836134839
HDL cholesterol	rs75609851	-0.146530934	0.122601109	0.232014989	-0.386829107	0.09376724
HDL cholesterol	rs75662196	0.220771127	0.189382677	0.24371923	-0.15041892	0.591961174
HDL cholesterol	rs7583067	0.178754471	0.332931358	0.59132917	-0.473790991	0.831299933
HDL cholesterol	rs7622114	-0.676761434	0.36453707	0.063382962	-1.391254091	0.037731223
HDL cholesterol	rs76247316	0.285812271	0.363738956	0.432006899	-0.427116084	0.998740625
HDL cholesterol	rs76428106	1.13634106	0.305786448	0.0002023	0.536999622	1.735682498
HDL cholesterol	rs76602912	-0.048138908	0.308983695	0.87619259	-0.65374695	0.557469135
HDL cholesterol	rs7665587	-0.463034185	0.299518179	0.122121437	-1.050089816	0.124021446
HDL cholesterol	rs76962725	-0.424736503	0.395880488	0.283320149	-1.20066226	0.351189254
HDL cholesterol	rs771481	0.004208532	0.185232242	0.981873385	-0.358846662	0.367263726
HDL cholesterol	rs7725218	-0.508908377	0.35622974	0.153120388	-1.207118667	0.189301913
HDL cholesterol	rs77320712	0.114711123	0.386567457	0.766662853	-0.642961093	0.872383338
HDL cholesterol	rs77605964	0.004349867	0.289480974	0.98801109	-0.563032841	0.571732576
HDL cholesterol	rs77767539	-0.335417408	0.393552036	0.394057465	-1.106779398	0.435944583

HDL cholesterol	rs7794796	-0.426474317	0.262466378	0.104189796	-0.940908419	0.087959784
HDL cholesterol	rs77960347	0.121422434	0.062069795	0.050438751	-0.000234364	0.243079233
HDL cholesterol	rs78058190	0.099126581	0.13655318	0.467888277	-0.168517651	0.366770813
HDL cholesterol	rs7817574	-0.504143259	0.159912948	0.001618175	-0.817572637	-0.190713882
HDL cholesterol	rs7826177	-0.176716875	0.384176016	0.645523867	-0.929701866	0.576268116
HDL cholesterol	rs7853377	0.093242242	0.330236082	0.777674955	-0.554020479	0.740504962
HDL cholesterol	rs79153732	-0.252110381	0.168396365	0.134360778	-0.582167258	0.077946495
HDL cholesterol	rs7924036	0.216830234	0.298088391	0.466979438	-0.367423013	0.801083481
HDL cholesterol	rs79600951	-0.014195889	0.067380308	0.833134596	-0.146261293	0.117869514
HDL cholesterol	rs79634051	-0.427547763	0.320868652	0.182705885	-1.05645032	0.201354795
HDL cholesterol	rs80005209	-0.209094503	0.085432429	0.014385752	-0.376542064	-0.041646941
HDL cholesterol	rs8007841	-0.142038162	0.340399646	0.676481784	-0.809221468	0.525145143
HDL cholesterol	rs8014289	0.567751973	0.276227424	0.039842438	0.026346221	1.109157725
HDL cholesterol	rs8081548	-0.306675881	0.237226016	0.196094697	-0.771638873	0.158287111
HDL cholesterol	rs8086351	0.055253714	0.064515053	0.391750661	-0.07119579	0.181703218
HDL cholesterol	rs830620	0.339996523	0.281036927	0.226358312	-0.210835854	0.8908289
HDL cholesterol	rs880674	-0.482923997	0.397004058	0.223824661	-1.26105195	0.295203956
HDL cholesterol	rs907866	0.775620516	0.227946974	0.000667392	0.328844448	1.222396584
HDL cholesterol	rs921919	-0.091088924	0.107792632	0.398089345	-0.302362482	0.120184635
HDL cholesterol	rs9327468	0.338713639	0.323774783	0.295496559	-0.295884935	0.973312214
HDL cholesterol	rs9347737	0.09239153	0.313080929	0.767914201	-0.521247092	0.706030152
HDL cholesterol	rs9465693	0.402448679	0.372618261	0.280117135	-0.327883111	1.13278047
HDL cholesterol	rs9604045	0.535166799	0.283432581	0.059004125	-0.02036106	1.090694658
HDL cholesterol	rs9622830	-0.487299553	0.263414947	0.064323453	-1.003592848	0.028993743
HDL cholesterol	rs964184	-0.640917109	0.057789169	1.39E-28	-0.754183881	-0.527650338
HDL cholesterol	rs9647335	-0.321676183	0.189780075	0.090076465	-0.69364513	0.050292765
HDL cholesterol	rs968050	-0.220709219	0.303185189	0.466632967	-0.814952189	0.373533752
HDL cholesterol	rs983663	0.131549774	0.334511309	0.694127774	-0.52409239	0.787191939
HDL cholesterol	rs9904004	-0.691734887	0.286080332	0.01560718	-1.252452338	-0.131017437
HDL cholesterol	rs9916613	-0.109703348	0.324343632	0.735188473	-0.745416868	0.526010171
HDL cholesterol	rs9933509	0.689434846	0.299551102	0.021359996	0.102314685	1.276555006
HDL cholesterol	rs998584	-0.282546995	0.121053755	0.019592465	-0.519812355	-0.045281634
HDL cholesterol	rs9987289	0.558314679	0.082429299	1.26E-11	0.396753253	0.719876105
HDL cholesterol	rs9989419	-0.100888255	0.029391785	0.000597972	-0.158496154	-0.043280356
HDL cholesterol	All - Inverse variance weighted	-0.130374343	0.034473799	0.000155674	-0.19794299	-0.062805697
HDL cholesterol	All - MR Egger	-0.205694736	0.052634292	0.00011196	-0.308857949	-0.102531524
LDL cholesterol	rs1010759	1.128461492	0.258478978	1.27E-05	0.621842695	1.635080288

LDL cholesterol	rs1016988	1.355826743	0.301937182	7.11E-06	0.764029866	1.94762362
LDL cholesterol	rs10231941	0.406579206	0.272372775	0.135507691	-0.127271433	0.940429844
LDL cholesterol	rs10448340	0.782361847	0.294330451	0.007858093	0.205474162	1.359249532
LDL cholesterol	rs10832963	0.971061892	0.275215967	0.000418138	0.431638597	1.510485187
LDL cholesterol	rs10910476	0.673727993	0.337457438	0.045881389	0.012311414	1.335144572
LDL cholesterol	rs11014204	1.137191497	0.334893855	0.00068459	0.480799542	1.793583452
LDL cholesterol	rs11065385	0.543078104	0.183019469	0.00300404	0.184359945	0.901796263
LDL cholesterol	rs11099097	0.685335063	0.25081814	0.006287508	0.193731509	1.176938618
LDL cholesterol	rs111273322	0.768798978	0.30769047	0.012468088	0.165725657	1.3718723
LDL cholesterol	rs111338114	1.280323066	0.342442388	0.000184902	0.609135986	1.951510146
LDL cholesterol	rs11226108	0.635741919	0.327647573	0.052340523	-0.006447323	1.277931162
LDL cholesterol	rs113177823	0.624707034	0.223914781	0.00527191	0.185834064	1.063580004
LDL cholesterol	rs114165349	0.885724448	0.246577389	0.000328056	0.402432766	1.36901613
LDL cholesterol	rs115458560	0.159032442	0.303310945	0.600054963	-0.435457011	0.753521895
LDL cholesterol	rs11568318	0.441099665	0.323921682	0.173277463	-0.193786831	1.075986161
LDL cholesterol	rs11591147	0.876366026	0.045197672	9.44E-84	0.787778589	0.964953463
LDL cholesterol	rs11601507	0.951784406	0.250105897	0.000141503	0.461576848	1.441991963
LDL cholesterol	rs11621792	1.429732256	0.218002601	5.44E-11	1.002447159	1.857017353
LDL cholesterol	rs116734477	1.376791418	0.220310625	4.12E-10	0.944982593	1.808600243
LDL cholesterol	rs117139027	0.558241377	0.273810816	0.041471493	0.021572177	1.094910577
LDL cholesterol	rs117733303	-1.383775284	0.182955172	3.92E-14	-1.742367421	-1.025183147
LDL cholesterol	rs118039278	-0.384819701	0.092234174	3.02E-05	-0.565598681	-0.204040721
LDL cholesterol	rs1183851	0.853833532	0.174192887	9.50E-07	0.512415474	1.19525159
LDL cholesterol	rs12078100	0.938509994	0.325696462	0.003957269	0.300144928	1.57687506
LDL cholesterol	rs12162782	1.366419223	0.335641089	4.68E-05	0.708562689	2.024275758
LDL cholesterol	rs12208357	1.099423876	0.142657013	1.29E-14	0.819816132	1.379031621
LDL cholesterol	rs12246352	1.068548779	0.265191288	5.59E-05	0.548773855	1.588323703
LDL cholesterol	rs1229984	0.372501174	0.237915013	0.117421191	-0.093812251	0.8388146
LDL cholesterol	rs12445804	0.52267626	0.347593611	0.132658817	-0.158607217	1.203959737
LDL cholesterol	rs12471768	0.834903198	0.33325245	0.012234151	0.181728395	1.488078001
LDL cholesterol	rs1250258	0.929529203	0.341849795	0.006545667	0.259503604	1.599554801
LDL cholesterol	rs1260326	1.328276212	0.121632369	9.21E-28	1.089876769	1.566675654
LDL cholesterol	rs12916	0.8941667	0.06793786	1.46E-39	0.761008495	1.027324905
LDL cholesterol	rs13020929	0.874826872	0.285058898	0.002148242	0.316111433	1.433542312
LDL cholesterol	rs13076933	0.682990378	0.225560636	0.002462042	0.240891531	1.125089225
LDL cholesterol	rs13107325	0.104795616	0.317380834	0.741257265	-0.51727082	0.726862051
LDL cholesterol	rs13108218	1.556178354	0.24059515	9.93E-11	1.084611859	2.027744849

LDL cholesterol	rs13121616	0.946641039	0.348368571	0.006580601	0.263838639	1.629443439
LDL cholesterol	rs1350559	0.320928866	0.307322382	0.296358499	-0.281423003	0.923280735
LDL cholesterol	rs140584594	1.022233077	0.322981158	0.001550867	0.389190007	1.655276148
LDL cholesterol	rs143020224	0.86427506	0.037621928	8.75E-117	0.790536081	0.938014039
LDL cholesterol	rs145730801	0.969746802	0.284095612	0.000641454	0.412919402	1.526574202
LDL cholesterol	rs146433259	0.759395751	0.357092078	0.033452462	0.059495277	1.459296225
LDL cholesterol	rs146534110	0.18045577	0.263312413	0.493136007	-0.33563656	0.696548101
LDL cholesterol	rs148150904	1.011673496	0.333058886	0.002385325	0.35887808	1.664468913
LDL cholesterol	rs150474434	0.865158519	0.197730012	1.21E-05	0.477607696	1.252709343
LDL cholesterol	rs1551891	1.091580851	0.04206211	1.74E-148	1.009139116	1.174022586
LDL cholesterol	rs1556562	0.526851652	0.260443945	0.043083618	0.016381521	1.037321784
LDL cholesterol	rs17050272	0.671619722	0.204319616	0.001012236	0.271153276	1.072086169
LDL cholesterol	rs174564	1.009160535	0.13582484	1.09E-13	0.742943849	1.275377222
LDL cholesterol	rs17476364	1.26780582	0.308070031	3.87E-05	0.663988559	1.871623081
LDL cholesterol	rs17569873	1.359827332	0.301657165	6.55E-06	0.768579289	1.951075376
LDL cholesterol	rs1801689	0.431796579	0.194254955	0.026226974	0.051056869	0.81253629
LDL cholesterol	rs183130	0.991218088	0.134094999	1.45E-13	0.728391891	1.254044286
LDL cholesterol	rs1883711	0.958503454	0.118177484	5.03E-16	0.726875586	1.190131322
LDL cholesterol	rs200046586	1.096232713	0.050156539	6.78E-106	0.997925896	1.194539529
LDL cholesterol	rs2043085	-0.602438594	0.249311317	0.01567436	-1.091088777	-0.113788412
LDL cholesterol	rs2066714	0.710579536	0.2923712	0.015082086	0.137531984	1.283627087
LDL cholesterol	rs2068888	0.715318017	0.216254601	0.000940458	0.291458999	1.139177035
LDL cholesterol	rs2073547	0.815546079	0.1491488	4.55E-08	0.523214432	1.107877726
LDL cholesterol	rs2160994	0.56764395	0.237565793	0.016875097	0.102014996	1.033272904
LDL cholesterol	rs2238162	1.18382139	0.250544822	2.30E-06	0.692753539	1.674889242
LDL cholesterol	rs2250802	0.466689935	0.254257746	0.066431731	-0.031655247	0.965035116
LDL cholesterol	rs2256814	0.78639479	0.341014166	0.021107991	0.118007024	1.454782555
LDL cholesterol	rs2287622	0.942454578	0.199712155	2.37E-06	0.551018755	1.333890401
LDL cholesterol	rs2391825	0.817648327	0.355863779	0.021582051	0.12015532	1.515141334
LDL cholesterol	rs2519093	0.970410326	0.095847397	4.30E-24	0.782549428	1.158271224
LDL cholesterol	rs2611867	1.208502923	0.156133435	9.93E-15	0.902481391	1.514524455
LDL cholesterol	rs2618566	0.886303261	0.175166334	4.20E-07	0.542977247	1.229629275
LDL cholesterol	rs2642438	0.471305357	0.178785473	0.008385261	0.12088583	0.821724885
LDL cholesterol	rs2737265	1.06894936	0.226998713	2.49E-06	0.624031883	1.513866836
LDL cholesterol	rs2738447	0.775615399	0.099495427	6.42E-15	0.580604361	0.970626436
LDL cholesterol	rs2740488	1.054719313	0.185927226	1.41E-08	0.69030195	1.419136675
LDL cholesterol	rs2745353	1.286776462	0.326658948	8.18E-05	0.646524925	1.927028

LDL cholesterol	rs2820226	1.431949627	0.324049285	9.92E-06	0.796813029	2.067086225
LDL cholesterol	rs28406917	0.727699337	0.352464786	0.038960718	0.036868357	1.418530317
LDL cholesterol	rs28590710	0.516790782	0.309781473	0.095267493	-0.090380906	1.12396247
LDL cholesterol	rs28615248	0.945624285	0.283180632	0.000839907	0.390590246	1.500658324
LDL cholesterol	rs28631087	1.083798124	0.314161741	0.000560982	0.468041112	1.699555136
LDL cholesterol	rs28814720	1.316336337	0.349579866	0.000166229	0.631159799	2.001512875
LDL cholesterol	rs3104412	1.317945002	0.219352484	1.87E-09	0.888014133	1.747875871
LDL cholesterol	rs3127580	0.244868373	0.159215026	0.124055276	-0.067193078	0.556929824
LDL cholesterol	rs34042070	0.924728014	0.109708858	3.49E-17	0.709698652	1.139757375
LDL cholesterol	rs34207171	0.891412032	0.356414336	0.012382378	0.192839933	1.589984132
LDL cholesterol	rs34265667	1.432144985	0.360996977	7.27E-05	0.724590911	2.13969906
LDL cholesterol	rs34568880	1.339814239	0.322964123	3.35E-05	0.706804558	1.972823919
LDL cholesterol	rs34596921	0.807013163	0.157853192	3.18E-07	0.497620906	1.11640542
LDL cholesterol	rs35278712	0.795339463	0.253695574	0.001718452	0.298096137	1.292582788
LDL cholesterol	rs35511051	0.678998632	0.232705107	0.003524537	0.222896623	1.135100642
LDL cholesterol	rs35882350	0.972044042	0.337384382	0.003962679	0.310770654	1.633317431
LDL cholesterol	rs35980001	-0.648779	0.228347937	0.004494607	-1.096340956	-0.201217043
LDL cholesterol	rs35990695	0.57695748	0.300473001	0.05483717	-0.011969602	1.165884562
LDL cholesterol	rs3732359	0.991656872	0.287630505	0.000565431	0.427901081	1.555412662
LDL cholesterol	rs375972689	1.072592866	0.121358323	9.72E-19	0.834730552	1.310455179
LDL cholesterol	rs3780181	0.903879448	0.295936312	0.002255834	0.323844277	1.483914618
LDL cholesterol	rs3822855	0.760478203	0.236421774	0.001297118	0.297091527	1.223864879
LDL cholesterol	rs3823376	1.090279787	0.238783613	4.97E-06	0.622263905	1.558295669
LDL cholesterol	rs4148826	1.038963506	0.342459013	0.002414679	0.36774384	1.710183172
LDL cholesterol	rs4263041	1.011727687	0.071422427	1.50E-45	0.87173973	1.151715643
LDL cholesterol	rs4307732	1.17790616	0.15026042	4.54E-15	0.883395737	1.472416582
LDL cholesterol	rs438568	1.056436683	0.340438542	0.001914634	0.38917714	1.723696226
LDL cholesterol	rs440677	0.807765734	0.269450759	0.002719154	0.279642246	1.335889222
LDL cholesterol	rs4666384	0.649858715	0.267732219	0.015212878	0.125103565	1.174613864
LDL cholesterol	rs472495	0.798171667	0.101620226	4.02E-15	0.598996023	0.99734731
LDL cholesterol	rs4738684	0.757740799	0.139305222	5.35E-08	0.484702564	1.030779035
LDL cholesterol	rs4930163	0.767097946	0.323844052	0.017849454	0.132363603	1.401832288
LDL cholesterol	rs4954192	0.482785124	0.289625517	0.095528558	-0.08488089	1.050451139
LDL cholesterol	rs4970834	1.024177849	0.05062192	5.13E-91	0.924958886	1.123396811
LDL cholesterol	rs556107	0.892534233	0.117857508	3.65E-14	0.661533516	1.123534949
LDL cholesterol	rs55637835	1.080543368	0.344616281	0.001715631	0.405095458	1.755991279
LDL cholesterol	rs55714927	1.401738084	0.198899793	1.82E-12	1.011894489	1.791581679

LDL cholesterol	rs55831924	0.885047939	0.276526687	0.001371476	0.343055632	1.427040246
LDL cholesterol	rs56130071	0.670829842	0.151328519	9.30E-06	0.374225945	0.96743374
LDL cholesterol	rs56236159	0.266067326	0.345441715	0.441167328	-0.410998436	0.943133088
LDL cholesterol	rs5843957	1.438959618	0.304937803	2.37E-06	0.841281523	2.036637712
LDL cholesterol	rs5849920	1.427902939	0.315024279	5.82E-06	0.810455351	2.045350527
LDL cholesterol	rs597808	0.88027639	0.153261936	9.27E-09	0.579882995	1.180669785
LDL cholesterol	rs60229127	1.245265527	0.313184003	7.00E-05	0.63142488	1.859106173
LDL cholesterol	rs6031587	1.008998787	0.322003361	0.001727326	0.3778722	1.640125375
LDL cholesterol	rs60612724	0.723852253	0.332383311	0.029423646	0.072380964	1.375323543
LDL cholesterol	rs6074012	1.103392056	0.355321486	0.00190064	0.406961943	1.799822168
LDL cholesterol	rs61003864	0.710776708	0.341366067	0.037328409	0.041699216	1.379854199
LDL cholesterol	rs61754230	1.122273707	0.346017479	0.001181115	0.444079448	1.800467966
LDL cholesterol	rs61988556	0.885964364	0.328946101	0.007073965	0.241230007	1.530698721
LDL cholesterol	rs62033400	0.676838666	0.293227993	0.020986155	0.102111801	1.251565532
LDL cholesterol	rs6475606	0.544395601	0.203764388	0.007546998	0.145017401	0.943773801
LDL cholesterol	rs6495122	1.246684092	0.292589606	2.04E-05	0.673208464	1.82015972
LDL cholesterol	rs6544713	0.848002055	0.082313572	6.89E-25	0.686667454	1.009336657
LDL cholesterol	rs6560499	0.709686302	0.344938047	0.039644766	0.033607729	1.385764874
LDL cholesterol	rs6602912	0.951062402	0.205858187	3.84E-06	0.547580355	1.354544449
LDL cholesterol	rs6667939	0.296315195	0.301827376	0.326229282	-0.295266462	0.887896852
LDL cholesterol	rs6680227	1.239917705	0.150915976	2.10E-16	0.944122393	1.535713017
LDL cholesterol	rs6709904	0.540222371	0.150856564	0.000342238	0.244543505	0.835901236
LDL cholesterol	rs6732741	0.850657508	0.229231454	0.000206515	0.401363859	1.299951158
LDL cholesterol	rs6874202	1.240852433	0.132474405	7.48E-21	0.981202598	1.500502267
LDL cholesterol	rs7108486	0.961368187	0.355203884	0.006799147	0.265168575	1.657567799
LDL cholesterol	rs71311871	0.866341898	0.265093456	0.001082866	0.346758723	1.385925072
LDL cholesterol	rs7202323	0.725898224	0.1919245	0.000155444	0.349726204	1.102070245
LDL cholesterol	rs7241918	0.322812454	0.344860959	0.349239452	-0.353115024	0.998739933
LDL cholesterol	rs72631343	1.338239562	0.210613091	2.10E-10	0.925437904	1.75104122
LDL cholesterol	rs72911393	0.972702731	0.319266889	0.002313871	0.346939628	1.598465833
LDL cholesterol	rs7562734	0.69659318	0.219908416	0.001536757	0.265572686	1.127613675
LDL cholesterol	rs7569317	1.015536044	0.23172138	1.17E-05	0.561362139	1.469709949
LDL cholesterol	rs76468627	0.821860076	0.360676065	0.022686962	0.114934988	1.528785163
LDL cholesterol	rs7707394	0.881685127	0.106481903	1.23E-16	0.672980597	1.090389656
LDL cholesterol	rs7734476	1.209243317	0.221736187	4.94E-08	0.77464039	1.643846243
LDL cholesterol	rs7746081	0.96727234	0.191697092	4.52E-07	0.591546039	1.342998641
LDL cholesterol	rs77542162	0.89931042	0.108206469	9.49E-17	0.68722574	1.1113951

LDL cholesterol	rs7776054	0.621017029	0.290143917	0.032324406	0.052334952	1.189699105
LDL cholesterol	rs77960347	0.500273998	0.255734492	0.050438751	-0.000965606	1.001513603
LDL cholesterol	rs78508096	1.5120626	0.279935432	6.61E-08	0.963389152	2.060736048
LDL cholesterol	rs79220007	1.033900729	0.135955387	2.86E-14	0.76742817	1.300373288
LDL cholesterol	rs79828839	0.991081959	0.357203749	0.005527704	0.29096261	1.691201308
LDL cholesterol	rs8107974	1.042481296	0.074389004	1.28E-44	0.896678848	1.188283744
LDL cholesterol	rs869412	0.785045744	0.34783015	0.024009315	0.103298649	1.466792838
LDL cholesterol	rs880315	0.857099688	0.287178847	0.002839956	0.294229148	1.419970228
LDL cholesterol	rs9289196	1.098283267	0.3149365	0.000487894	0.481007726	1.715558808
LDL cholesterol	rs934197	0.884338041	0.052506163	1.19E-63	0.781425961	0.987250121
LDL cholesterol	rs9471968	1.065494025	0.356880015	0.002830406	0.366009195	1.764978855
LDL cholesterol	rs9496567	1.195839739	0.276755433	1.55E-05	0.653399091	1.738280387
LDL cholesterol	rs960596	0.805127787	0.325393135	0.013348887	0.167357242	1.442898333
LDL cholesterol	rs964184	1.173595011	0.105818802	1.39E-28	0.966190159	1.380999863
LDL cholesterol	rs9832727	0.667211593	0.296687759	0.024520899	0.085703585	1.248719601
LDL cholesterol	rs9834932	0.901204946	0.225211067	6.29E-05	0.459791254	1.342618638
LDL cholesterol	rs9884390	0.430881669	0.196468235	0.028297542	0.045803928	0.81595941
LDL cholesterol	rs9894946	0.883432468	0.331344291	0.007671244	0.233997658	1.532867279
LDL cholesterol	rs9929977	0.452938082	0.256411529	0.077320424	-0.049628515	0.955504678
LDL cholesterol	rs9987289	1.073883268	0.158547587	1.26E-11	0.763129998	1.384636538
LDL cholesterol	All - Inverse variance weighted	0.896585088	0.024503402	4.09E-293	0.848558419	0.944611757
LDL cholesterol	All - MR Egger	0.922271312	0.0366306	9.72E-59	0.850475336	0.994067287
Triglycerides	rs1009360	0.060920187	0.226286574	0.787762718	-0.382601498	0.504441872
Triglycerides	rs1009590	0.964471707	0.358633682	0.007160315	0.26154969	1.667393725
Triglycerides	rs10152471	-1.025368134	0.315209047	0.001142023	-1.643177865	-0.407558402
Triglycerides	rs10210970	0.761942234	0.261389681	0.003557264	0.249618459	1.274266008
Triglycerides	rs10242866	-0.28614766	0.267231685	0.284266146	-0.809921763	0.237626442
Triglycerides	rs10277582	0.606784715	0.369560312	0.100608689	-0.117553496	1.331122926
Triglycerides	rs1037117	-0.673671558	0.276679461	0.014898177	-1.215963301	-0.131379815
Triglycerides	rs10405944	0.259799379	0.317073208	0.412576956	-0.361664108	0.881262866
Triglycerides	rs1043897	0.364165985	0.285580333	0.202245965	-0.195571468	0.923903437
Triglycerides	rs1044808	0.236020558	0.304777124	0.438692047	-0.361342605	0.833383721
Triglycerides	rs1045241	-0.025270789	0.225880727	0.910921177	-0.467997014	0.417455437
Triglycerides	rs10513688	0.552867311	0.280774166	0.048943694	0.002549946	1.103184676
Triglycerides	rs10631642	0.448399766	0.348105977	0.197706699	-0.233887949	1.13068748
Triglycerides	rs1064173	1.087936887	0.223629	1.15E-06	0.649624047	1.526249727
Triglycerides	rs10642257	-0.655800798	0.152596095	1.73E-05	-0.954889144	-0.356712451

Triglycerides	rs10750766	-0.409001057	0.234939001	0.081704279	-0.869481499	0.051479384
Triglycerides	rs10773000	0.303324313	0.295290501	0.304323252	-0.275445068	0.882093694
Triglycerides	rs10773049	0.249410464	0.145879448	0.087320891	-0.036513254	0.535334183
Triglycerides	rs10775406	0.044827541	0.234622963	0.84847699	-0.415033465	0.504688548
Triglycerides	rs1077835	-0.324919564	0.105587669	0.002089206	-0.531871395	-0.117967733
Triglycerides	rs10797119	-0.144196852	0.264785855	0.586043008	-0.663177127	0.374783423
Triglycerides	rs10811662	0.009006801	0.353716466	0.979685393	-0.684277473	0.702291075
Triglycerides	rs10883026	0.468575558	0.288382756	0.104196861	-0.096654643	1.033805759
Triglycerides	rs10899490	0.154283228	0.329322327	0.639436367	-0.491188533	0.799754989
Triglycerides	rs11000468	-0.156833665	0.324965667	0.629368339	-0.793766372	0.480099041
Triglycerides	rs11030107	-0.427895249	0.293158498	0.144399003	-1.002485906	0.146695408
Triglycerides	rs11078597	0.866213199	0.278102635	0.001841165	0.321132034	1.411294364
Triglycerides	rs11100083	-0.318605536	0.308320229	0.301435845	-0.922913185	0.285702112
Triglycerides	rs11118310	0.279669528	0.217857624	0.199237792	-0.147331415	0.70667047
Triglycerides	rs11122450	0.491326324	0.088004235	2.36E-08	0.318838024	0.663814624
Triglycerides	rs11185542	-0.100064085	0.36763084	0.785478761	-0.820620531	0.620492361
Triglycerides	rs11187019	0.782302876	0.35862913	0.02915605	0.079389782	1.485215971
Triglycerides	rs11206374	-0.042290754	0.198035423	0.830896854	-0.430440184	0.345858675
Triglycerides	rs112108602	0.086084246	0.270616001	0.75040545	-0.444323115	0.616491607
Triglycerides	rs112381903	0.403402562	0.359898812	0.262339914	-0.301999111	1.108804234
Triglycerides	rs11240358	0.669868265	0.310963428	0.031226798	0.060379946	1.279356584
Triglycerides	rs11274835	0.508161066	0.22639977	0.024798324	0.064417516	0.951904616
Triglycerides	rs1133400	0.001710669	0.363302585	0.996243045	-0.710362398	0.713783737
Triglycerides	rs113344423	0.060169909	0.206428091	0.770683316	-0.34442915	0.464768968
Triglycerides	rs114165349	0.608749752	0.169470228	0.000328056	0.276588105	0.9409114
Triglycerides	rs11429307	0.393640063	0.113203562	0.000506536	0.171761082	0.615519045
Triglycerides	rs11434143	1.153334171	0.337353403	0.000629045	0.492121501	1.814546842
Triglycerides	rs11600815	0.270245202	0.295265902	0.360054917	-0.308475965	0.848966369
Triglycerides	rs11637681	0.023474965	0.369970445	0.94940744	-0.701667106	0.748617037
Triglycerides	rs11664106	0.953405759	0.347735455	0.006111172	0.271844267	1.63496725
Triglycerides	rs116843064	0.048205117	0.065293481	0.460342034	-0.079770107	0.17618034
Triglycerides	rs117233107	0.578966486	0.24564089	0.018425083	0.09751034	1.060422631
Triglycerides	rs117287238	0.458828273	0.327849687	0.161660689	-0.183757113	1.10141366
Triglycerides	rs117291242	0.74115917	0.369211451	0.044705907	0.017504726	1.464813615
Triglycerides	rs117316645	0.985260293	0.369590492	0.007680169	0.26086293	1.709657657
Triglycerides	rs117431393	-0.255861254	0.358682355	0.47563749	-0.958878669	0.447156161
Triglycerides	rs11746801	0.537370307	0.350395853	0.125125822	-0.149405565	1.224146179

Triglycerides	rs11904650	0.855265305	0.352663275	0.015301589	0.164045285	1.546485324
Triglycerides	rs12185242	-0.001850723	0.237838701	0.993791387	-0.468014578	0.464313132
Triglycerides	rs12424054	0.754361651	0.255930067	0.003203233	0.252738719	1.255984583
Triglycerides	rs12440800	0.113563354	0.297334161	0.702507006	-0.469211602	0.696338311
Triglycerides	rs12446515	0.977115979	0.132732708	1.82E-13	0.716959872	1.237272086
Triglycerides	rs12475332	-0.005114059	0.335196812	0.987827241	-0.662099809	0.651871692
Triglycerides	rs12504746	0.536687976	0.345191061	0.120004121	-0.139886503	1.213262455
Triglycerides	rs12530679	-0.287549774	0.346330967	0.406383597	-0.96635847	0.391258922
Triglycerides	rs12669911	-0.260838901	0.360261861	0.469049864	-0.96695215	0.445274347
Triglycerides	rs12880341	0.260236724	0.271479189	0.337766283	-0.271862487	0.792335934
Triglycerides	rs12902047	0.367200761	0.345506874	0.287877838	-0.309992712	1.044394235
Triglycerides	rs1292065	0.139645353	0.327014371	0.669356573	-0.501302815	0.78059352
Triglycerides	rs12926107	-0.031892707	0.329240923	0.922831711	-0.677204916	0.613419502
Triglycerides	rs12928099	-0.214625544	0.161108906	0.182802103	-0.530398999	0.101147911
Triglycerides	rs12948505	0.061378171	0.376009994	0.870333034	-0.675601417	0.798357758
Triglycerides	rs13066793	-0.037275484	0.321667713	0.907746096	-0.667744201	0.593193234
Triglycerides	rs13101504	0.41801092	0.256528185	0.103208664	-0.084784323	0.920806164
Triglycerides	rs13107325	-0.086530629	0.262064047	0.741257265	-0.60017616	0.427114903
Triglycerides	rs13108218	0.907035118	0.140233444	9.93E-11	0.632177567	1.181892669
Triglycerides	rs13118477	0.109497855	0.283375251	0.699196117	-0.445917637	0.664913347
Triglycerides	rs1316753	0.101906925	0.289992589	0.725279154	-0.46647855	0.6702924
Triglycerides	rs13264304	-0.034774725	0.306790515	0.909753027	-0.636084134	0.566534685
Triglycerides	rs13269725	0.187355593	0.220153311	0.394756421	-0.244144896	0.618856081
Triglycerides	rs13354321	0.10206539	0.2718525	0.707330287	-0.43076551	0.63489629
Triglycerides	rs13389219	0.412037997	0.112525183	0.000250508	0.191488638	0.632587356
Triglycerides	rs1340819	0.175606428	0.357663064	0.623438582	-0.525413177	0.876626033
Triglycerides	rs134551	0.716636061	0.376151575	0.056756814	-0.020621027	1.453893149
Triglycerides	rs1347188	0.783989469	0.346240496	0.023556182	0.105358097	1.462620841
Triglycerides	rs138191773	0.081583237	0.349811434	0.815590099	-0.604047172	0.767213647
Triglycerides	rs139386986	0.328062775	0.341112387	0.336178239	-0.340517503	0.996643052
Triglycerides	rs139974673	0.052050562	0.091117821	0.567833861	-0.126540368	0.230641491
Triglycerides	rs140107293	0.627264838	0.252458778	0.012968877	0.132445633	1.122084043
Triglycerides	rs140288	0.20070576	0.314907671	0.523898221	-0.416513274	0.817924794
Triglycerides	rs1420384	-0.211171228	0.342974199	0.538088662	-0.883400657	0.461058202
Triglycerides	rs143076454	0.707739376	0.374221223	0.058593302	-0.025734222	1.441212973
Triglycerides	rs145947882	0.010791725	0.095832029	0.910339091	-0.177039051	0.198622502
Triglycerides	rs1473886	0.590250417	0.228816122	0.009891939	0.141770817	1.038730016

Triglycerides	rs148827772	0.172568075	0.318081441	0.587454852	-0.450871549	0.796007699
Triglycerides	rs149142833	0.92778611	0.338856741	0.00618161	0.263626897	1.591945323
Triglycerides	rs149778057	0.163668384	0.294059073	0.577812131	-0.412687398	0.740024167
Triglycerides	rs150419156	0.008669729	0.345565005	0.979984324	-0.668637681	0.685977139
Triglycerides	rs150423652	0.363139554	0.090525288	6.03E-05	0.185709989	0.540569119
Triglycerides	rs150460588	0.542544471	0.309378747	0.07948915	-0.063837872	1.148926815
Triglycerides	rs150555490	0.211910971	0.231630725	0.360262217	-0.24208525	0.665907192
Triglycerides	rs150564454	0.234338729	0.19850026	0.237783045	-0.154721782	0.623399239
Triglycerides	rs151235402	-0.145539282	0.327957412	0.657205306	-0.788335809	0.497257245
Triglycerides	rs1544980	0.050589014	0.21758454	0.816147515	-0.375876684	0.477054712
Triglycerides	rs1567353	0.285426647	0.30368551	0.347281296	-0.309796953	0.880650248
Triglycerides	rs17184382	0.289218293	0.190950098	0.129866733	-0.085043899	0.663480486
Triglycerides	rs17326656	0.284248311	0.279941397	0.309922314	-0.264436827	0.83293345
Triglycerides	rs174566	-0.666262559	0.089276503	8.46E-14	-0.841244504	-0.491280614
Triglycerides	rs17585887	0.461967682	0.147045118	0.001679855	0.173759251	0.750176114
Triglycerides	rs1760801	0.264433617	0.224226898	0.238273704	-0.175051103	0.703918337
Triglycerides	rs1799831	0.439663756	0.232673223	0.058808927	-0.016375761	0.895703273
Triglycerides	rs1801689	-0.403928625	0.181717829	0.026226974	-0.760095569	-0.047761682
Triglycerides	rs1835346	0.63770775	0.348359307	0.067159753	-0.045076491	1.320491991
Triglycerides	rs186413375	0.271652755	0.230664051	0.238916741	-0.180448785	0.723754296
Triglycerides	rs186696265	1.309102935	0.167184954	4.87E-15	0.981420426	1.636785445
Triglycerides	rs188247550	0.716038902	0.140278212	3.32E-07	0.441093606	0.990984198
Triglycerides	rs193735	-0.162038101	0.333501108	0.627059515	-0.815700273	0.491624071
Triglycerides	rs1938566	0.346290194	0.262017565	0.186291491	-0.167264233	0.859844621
Triglycerides	rs200610097	0.027677593	0.373307681	0.940897791	-0.704005462	0.759360647
Triglycerides	rs2043085	-0.332836625	0.137740076	0.01567436	-0.602807173	-0.062866077
Triglycerides	rs2068888	0.431779043	0.130535234	0.000940458	0.175929985	0.687628102
Triglycerides	rs2070341	0.909726401	0.374957527	0.01525736	0.174809649	1.644643153
Triglycerides	rs2071887	0.497911354	0.266294139	0.061514428	-0.024025159	1.019847867
Triglycerides	rs2081194	0.907432922	0.20185321	6.94E-06	0.511800631	1.303065213
Triglycerides	rs2081687	0.913294488	0.167648128	5.10E-08	0.584704158	1.241884819
Triglycerides	rs2092203	1.742512212	0.301846354	7.79E-09	1.150893358	2.334131066
Triglycerides	rs2131311	0.010331693	0.37406447	0.977965161	-0.722834669	0.743498055
Triglycerides	rs2131919	0.404606624	0.321925411	0.208813952	-0.226367181	1.03558043
Triglycerides	rs213494	0.04405199	0.277259683	0.873760656	-0.499376987	0.587480968
Triglycerides	rs2137557	-0.007566908	0.367031388	0.983551566	-0.726948429	0.711814613
Triglycerides	rs2187114	0.340310341	0.369787363	0.357423029	-0.38447289	1.065093572

Triglycerides	rs2237029	-0.382459702	0.304501467	0.209108993	-0.979282577	0.214363173
Triglycerides	rs2240466	0.190033315	0.050995528	0.00019418	0.090082079	0.28998455
Triglycerides	rs2240533	0.443141874	0.344633055	0.198500015	-0.232338915	1.118622663
Triglycerides	rs2244278	0.981393936	0.236221065	3.26E-05	0.518400649	1.444387223
Triglycerides	rs2267373	0.319451117	0.194941059	0.101274467	-0.062633358	0.701535592
Triglycerides	rs2302263	-0.042314589	0.16767734	0.800764956	-0.370962175	0.286332997
Triglycerides	rs2304969	0.336092377	0.365776093	0.358175446	-0.380828766	1.05301352
Triglycerides	rs2305746	1.028077043	0.290324693	0.000398419	0.459040645	1.597113441
Triglycerides	rs2382825	0.89990651	0.317119029	0.004543186	0.278353214	1.521459807
Triglycerides	rs2407278	0.446016927	0.362558146	0.218624392	-0.264597038	1.156630893
Triglycerides	rs2487294	-0.433728731	0.252321894	0.085623468	-0.928279643	0.06082218
Triglycerides	rs2519093	-2.556002458	0.252456281	4.30E-24	-3.050816769	-2.061188146
Triglycerides	rs2604568	0.888345321	0.375251234	0.017916715	0.152852903	1.623837739
Triglycerides	rs2699805	-0.249079791	0.21045089	0.236590038	-0.661563535	0.163403953
Triglycerides	rs275184	0.926778375	0.330012047	0.004980121	0.279954763	1.573601987
Triglycerides	rs2773469	0.794390542	0.248640557	0.001398621	0.307055051	1.281726034
Triglycerides	rs278981	0.878558206	0.378538345	0.020291021	0.136623051	1.620493362
Triglycerides	rs2812208	0.342062115	0.3000413	0.254265428	-0.246018833	0.930143064
Triglycerides	rs28383314	0.576097941	0.112518568	3.05E-07	0.355561547	0.796634335
Triglycerides	rs28439112	0.650152364	0.376389131	0.084106447	-0.087570333	1.387875062
Triglycerides	rs28577186	0.07363059	0.268641848	0.78401962	-0.452907433	0.600168612
Triglycerides	rs28752924	0.563788826	0.235969247	0.016882945	0.101289103	1.026288549
Triglycerides	rs2925979	0.046226544	0.139843275	0.740977129	-0.227866274	0.320319362
Triglycerides	rs2937124	0.341408625	0.242210001	0.158670748	-0.133322977	0.816140227
Triglycerides	rs2943645	-0.035875786	0.107016564	0.7374472	-0.245628252	0.17387668
Triglycerides	rs2983896	0.300044231	0.366414815	0.412863498	-0.418128807	1.018217269
Triglycerides	rs308	0.187028034	0.092374524	0.042901143	0.005973967	0.368082101
Triglycerides	rs3103310	0.447597372	0.243464544	0.065996135	-0.029593135	0.924787878
Triglycerides	rs320369	0.432705642	0.356500993	0.22484046	-0.266036305	1.131447589
Triglycerides	rs325485	0.974275131	0.361707327	0.00706965	0.265328769	1.683221493
Triglycerides	rs326222	-0.12776216	0.17821953	0.473447647	-0.477072438	0.221548118
Triglycerides	rs343	0.154170171	0.053357269	0.003859851	0.049589925	0.258750417
Triglycerides	rs34302257	-0.016569686	0.374291625	0.964689619	-0.750181271	0.717041898
Triglycerides	rs34389637	0.330261415	0.331637907	0.319323318	-0.319748883	0.980271712
Triglycerides	rs34672664	-0.683574354	0.37591476	0.068998568	-1.420367283	0.053218575
Triglycerides	rs34682685	1.310990789	0.202147717	8.85E-11	0.914781263	1.707200315
Triglycerides	rs35140741	0.020372563	0.276463639	0.941257179	-0.52149617	0.562241296

Triglycerides	rs35763453	0.652365554	0.318610896	0.040606017	0.027888197	1.27684291
Triglycerides	rs35786744	0.075934416	0.371558922	0.838066646	-0.652321071	0.804189902
Triglycerides	rs36043408	0.395351833	0.322812949	0.220684988	-0.237361547	1.028065213
Triglycerides	rs3731696	0.833656863	0.289133046	0.003935344	0.266956093	1.400357634
Triglycerides	rs3758413	1.011013018	0.377976661	0.007477323	0.270178762	1.751847274
Triglycerides	rs3775228	0.203556803	0.125290881	0.104232098	-0.042013324	0.449126929
Triglycerides	rs3808477	1.665904642	0.344051728	1.29E-06	0.991563255	2.340246029
Triglycerides	rs3814883	-0.198021836	0.278813639	0.477561463	-0.744496569	0.348452896
Triglycerides	rs3820897	-0.060763222	0.275260685	0.825288722	-0.600274164	0.47874772
Triglycerides	rs3860846	0.283737505	0.157555037	0.071722006	-0.025070367	0.592545378
Triglycerides	rs394872	0.099585894	0.372689545	0.789308468	-0.630885615	0.830057402
Triglycerides	rs3974807	0.08085695	0.332416007	0.807819295	-0.570678425	0.732392324
Triglycerides	rs4128205	0.964299067	0.361484554	0.007639334	0.255789342	1.672808792
Triglycerides	rs4134963	-0.26367176	0.279140991	0.344871947	-0.810788102	0.283444583
Triglycerides	rs41785	0.452011034	0.279005617	0.10521539	-0.094839975	0.998862043
Triglycerides	rs4253750	0.175747849	0.285041898	0.537519392	-0.38293427	0.734429968
Triglycerides	rs4471666	-0.470735942	0.366041138	0.198436351	-1.188176573	0.246704688
Triglycerides	rs4665972	0.447167082	0.042408279	5.40E-26	0.364046855	0.53028731
Triglycerides	rs4675812	0.244436246	0.295170936	0.407603904	-0.334098788	0.82297128
Triglycerides	rs4731701	0.339828056	0.127127129	0.007514587	0.090658883	0.588997228
Triglycerides	rs4760254	0.52619628	0.171747319	0.002185584	0.189571535	0.862821025
Triglycerides	rs4761234	0.329431815	0.295355686	0.264690532	-0.24946533	0.90832896
Triglycerides	rs4765148	0.675860808	0.177202956	0.000136708	0.328543014	1.023178602
Triglycerides	rs480823	0.251126381	0.049793989	4.58E-07	0.153530162	0.3487226
Triglycerides	rs483082	-0.770986372	0.056332942	1.23E-42	-0.881398939	-0.660573805
Triglycerides	rs483808	0.112101496	0.332564624	0.736055206	-0.539725168	0.763928159
Triglycerides	rs4841580	0.232606171	0.170439272	0.172333163	-0.101454803	0.566667145
Triglycerides	rs4969179	-0.177196965	0.238559334	0.457615234	-0.64477326	0.29037933
Triglycerides	rs4976033	0.507048547	0.240394809	0.034924516	0.035874722	0.978222372
Triglycerides	rs499293	0.718382353	0.369152419	0.051650918	-0.005156388	1.441921094
Triglycerides	rs55646464	0.841009572	0.371386152	0.023542448	0.113092714	1.568926431
Triglycerides	rs55767272	-0.270875959	0.29690083	0.361587184	-0.852801586	0.311049668
Triglycerides	rs55807798	0.082854345	0.359276851	0.817614533	-0.621328284	0.787036973
Triglycerides	rs55966194	0.022211317	0.257617263	0.9312929	-0.482718518	0.527141153
Triglycerides	rs56397607	0.338868522	0.306308228	0.268597074	-0.261495605	0.93923265
Triglycerides	rs56902258	0.362238335	0.345171494	0.293973574	-0.314297793	1.038774463
Triglycerides	rs57996145	0.247815394	0.192201573	0.197275901	-0.128899689	0.624530476

Triglycerides	rs581080	0.395519062	0.30719367	0.197911893	-0.206580533	0.997618656
Triglycerides	rs58542926	1.074934098	0.076189332	3.36E-45	0.925603008	1.224265188
Triglycerides	rs6028716	0.440112466	0.370658337	0.235077585	-0.286377875	1.166602806
Triglycerides	rs6073958	0.771899553	0.093144113	1.16E-16	0.589337092	0.954462015
Triglycerides	rs60856912	0.842773095	0.227043156	0.000205668	0.397768509	1.287777682
Triglycerides	rs61729990	0.391449772	0.280553924	0.162933041	-0.15843592	0.941335463
Triglycerides	rs61830291	0.847772398	0.243593035	0.000500887	0.37033005	1.325214746
Triglycerides	rs61905078	0.234016849	0.039698796	3.75E-09	0.156207208	0.31182649
Triglycerides	rs61993685	0.348408498	0.331073917	0.292635036	-0.30049638	0.997313375
Triglycerides	rs62102718	-0.045388341	0.226343725	0.841067363	-0.489022041	0.398245359
Triglycerides	rs62117489	0.318808256	0.209249012	0.127612889	-0.091319807	0.728936319
Triglycerides	rs62128802	0.413708062	0.337491338	0.220261479	-0.247774961	1.075191085
Triglycerides	rs62135012	0.367880186	0.369008869	0.318792998	-0.355377198	1.09113757
Triglycerides	rs62271373	0.239491563	0.211509893	0.257510494	-0.175067827	0.654050953
Triglycerides	rs62274099	0.345694975	0.348778147	0.321607421	-0.337910193	1.029300144
Triglycerides	rs62397245	0.169630054	0.331837189	0.609221815	-0.480770837	0.820030946
Triglycerides	rs62427982	0.625203338	0.333635742	0.060942838	-0.028722717	1.279129393
Triglycerides	rs62459095	0.223505541	0.276783643	0.419372753	-0.318990399	0.766001482
Triglycerides	rs62473520	0.652526267	0.374763866	0.081654407	-0.08201091	1.387063445
Triglycerides	rs6432622	0.425881802	0.379020465	0.261166624	-0.31699831	1.168761914
Triglycerides	rs6506033	-0.420637437	0.348588326	0.227552225	-1.103870556	0.262595681
Triglycerides	rs6517522	0.816338709	0.320567151	0.010879515	0.188027094	1.444650324
Triglycerides	rs6532798	0.601347158	0.326801769	0.065753907	-0.03918431	1.241878625
Triglycerides	rs6562773	0.671577428	0.346780268	0.052793284	-0.008111898	1.351266754
Triglycerides	rs6572807	-0.258211432	0.374267187	0.490249516	-0.991775118	0.475352255
Triglycerides	rs676210	0.737450845	0.069322972	1.99E-26	0.60157782	0.87332387
Triglycerides	rs6792725	0.282848771	0.302445212	0.349681817	-0.309943845	0.875641386
Triglycerides	rs67981690	0.550692623	0.207113042	0.00783965	0.14475106	0.956634185
Triglycerides	rs6800707	0.27364145	0.176795227	0.121673457	-0.072877195	0.620160095
Triglycerides	rs6805924	1.149666314	0.380906057	0.002542465	0.403090442	1.896242187
Triglycerides	rs684773	0.406937723	0.168020115	0.015437119	0.077618298	0.736257147
Triglycerides	rs6882076	1.211850966	0.129375157	7.47E-21	0.958275659	1.465426273
Triglycerides	rs696825	-0.180723533	0.234849486	0.441579227	-0.641028525	0.279581459
Triglycerides	rs698927	-0.00654401	0.291560644	0.982093171	-0.578002873	0.564914853
Triglycerides	rs6999569	0.547732856	0.048141273	5.41E-30	0.453375961	0.642089752
Triglycerides	rs7000494	0.232842872	0.089669543	0.009413029	0.057090568	0.408595175
Triglycerides	rs7077812	0.693573417	0.36686697	0.058686965	-0.025485845	1.412632678

Triglycerides	rs7134375	0.018581678	0.243128299	0.939079024	-0.457949789	0.495113145
Triglycerides	rs7135509	1.030180158	0.378972787	0.006560867	0.287393496	1.77296682
Triglycerides	rs71368855	0.267696084	0.249660185	0.283611444	-0.221637878	0.757030046
Triglycerides	rs7140110	0.743749382	0.159483282	3.11E-06	0.431162149	1.056336615
Triglycerides	rs71538127	0.113710381	0.357981574	0.750755668	-0.587933505	0.815354267
Triglycerides	rs71603401	0.476348253	0.229270051	0.037739464	0.026978954	0.925717553
Triglycerides	rs7215055	0.503490197	0.218885346	0.021434273	0.074474919	0.932505476
Triglycerides	rs7239575	0.349921937	0.257600129	0.174339354	-0.154974317	0.85481819
Triglycerides	rs7244	0.373013199	0.358911816	0.298670224	-0.33045396	1.076480359
Triglycerides	rs72555385	0.470671912	0.14677596	0.001342496	0.182991031	0.758352794
Triglycerides	rs72603744	0.195584196	0.35428325	0.580909983	-0.498810975	0.889979366
Triglycerides	rs72644085	0.494698423	0.298458446	0.097416028	-0.090280132	1.079676978
Triglycerides	rs7274718	0.144399997	0.263512675	0.583704797	-0.372084845	0.66088484
Triglycerides	rs72784786	0.213499932	0.331970106	0.520139755	-0.437161477	0.86416134
Triglycerides	rs72801474	0.123859998	0.231733985	0.593001074	-0.330338614	0.578058609
Triglycerides	rs729761	0.050710302	0.259564396	0.845105695	-0.458035914	0.559456518
Triglycerides	rs73025562	1.762978512	0.347832767	4.01E-07	1.081226288	2.444730735
Triglycerides	rs7308584	-0.219044349	0.356839698	0.539317601	-0.918450157	0.480361459
Triglycerides	rs73238173	0.492881076	0.370663139	0.183607976	-0.233618677	1.219380829
Triglycerides	rs7400002	0.910916887	0.352421958	0.009745426	0.220169848	1.601663925
Triglycerides	rs74090351	0.792004702	0.329039933	0.016083422	0.147086434	1.43692297
Triglycerides	rs742036	0.592810794	0.297405871	0.046231671	0.009895288	1.175726301
Triglycerides	rs7424120	-0.102780003	0.342237437	0.763934737	-0.773565381	0.568005374
Triglycerides	rs75268115	1.305016029	0.360505701	0.000294649	0.598424856	2.011607202
Triglycerides	rs75609851	0.12516975	0.1047284	0.232014989	-0.080097913	0.330437413
Triglycerides	rs75634664	0.500028976	0.248021459	0.043792508	0.013906917	0.986151036
Triglycerides	rs75721796	0.274517675	0.243415633	0.259415631	-0.202576965	0.751612316
Triglycerides	rs75942983	0.524999627	0.373010338	0.159289046	-0.206100634	1.256099889
Triglycerides	rs77009508	0.166051247	0.17511754	0.343013707	-0.17717913	0.509281625
Triglycerides	rs7704653	0.595247345	0.296638799	0.044787986	0.013835298	1.176659392
Triglycerides	rs7714361	0.472927019	0.354960463	0.182749418	-0.222795489	1.168649527
Triglycerides	rs7735249	0.337783982	0.245787034	0.169350005	-0.143958606	0.81952657
Triglycerides	rs7786339	0.105073493	0.339455537	0.756914315	-0.560259358	0.770406345
Triglycerides	rs78058190	-0.095194781	0.131136874	0.467888277	-0.352223054	0.161833492
Triglycerides	rs7847285	0.431685386	0.363537863	0.235047619	-0.280848825	1.144219596
Triglycerides	rs78484485	0.114264847	0.121496344	0.346971549	-0.123867987	0.35239768
Triglycerides	rs78588343	-0.052534545	0.348108947	0.880043485	-0.734828082	0.629758991

Triglycerides	rs7861679	0.661995493	0.369809465	0.073438024	-0.062831059	1.386822045
Triglycerides	rs79153732	0.306250048	0.204558792	0.134360778	-0.094685184	0.707185281
Triglycerides	rs79287178	0.570402798	0.250188643	0.022614163	0.080033057	1.060772538
Triglycerides	rs79357714	0.746437369	0.341993968	0.029064992	0.076129191	1.416745546
Triglycerides	rs7947951	0.129209349	0.230626442	0.57530631	-0.322818477	0.581237175
Triglycerides	rs80276949	0.523954078	0.304058676	0.084852264	-0.072000927	1.119909084
Triglycerides	rs8102873	-0.044253459	0.339404876	0.896261474	-0.709487016	0.620980098
Triglycerides	rs8126001	0.544365544	0.252726251	0.031242283	0.049022091	1.039708996
Triglycerides	rs852388	0.124987271	0.324765154	0.700345354	-0.51155243	0.761526972
Triglycerides	rs867939	0.117291691	0.309055627	0.704304399	-0.488457339	0.723040721
Triglycerides	rs880315	1.108324839	0.37135406	0.002839956	0.38047088	1.836178797
Triglycerides	rs921971	0.129641534	0.300866454	0.666545485	-0.460056716	0.719339783
Triglycerides	rs9373056	0.254700869	0.359926408	0.479164453	-0.450754891	0.960156628
Triglycerides	rs9376511	0.300424535	0.330234914	0.362965035	-0.346835896	0.947684966
Triglycerides	rs9425589	0.556638666	0.302302751	0.065573436	-0.035874726	1.149152059
Triglycerides	rs9436661	0.396728769	0.055662518	1.02E-12	0.287630233	0.505827305
Triglycerides	rs9480889	0.480169501	0.308192951	0.119229493	-0.123888684	1.084227686
Triglycerides	rs954244	0.795405599	0.309787248	0.010240919	0.188222593	1.402588605
Triglycerides	rs9561643	-0.164463443	0.266127826	0.536584141	-0.686073982	0.357147096
Triglycerides	rs9584870	-0.250887004	0.353546398	0.477933832	-0.943837944	0.442063936
Triglycerides	rs970069	-0.367553998	0.312621826	0.239709003	-0.980292776	0.245184781
Triglycerides	rs9831084	0.533597151	0.351131493	0.128598837	-0.154620574	1.221814877
Triglycerides	rs9859117	-0.106302078	0.349381831	0.760931644	-0.791090466	0.578486311
Triglycerides	rs9889402	0.225601049	0.377966571	0.550586862	-0.51521343	0.966415528
Triglycerides	rs9902027	0.726065075	0.326910672	0.026351692	0.085320159	1.366809991
Triglycerides	rs998584	0.240796197	0.103166144	0.019592465	0.038590555	0.44300184
Triglycerides	All - Inverse variance weighted	0.279314364	0.023931488	1.78E-31	0.232408648	0.326220081
Triglycerides	All - MR Egger	0.251355254	0.036487467	3.35E-11	0.179839818	0.32287069
