# Supplementary material for: Small dense low density lipoprotein predominance in patients with type 2 diabetes mellitus using Mendelian randomization
Source: PLoS One. 2024 Feb 8;19(2):e0298070. doi: 10.1371/journal.pone.0298070 (PMC10852223; doi:10.1371/journal.pone.0298070)
Supplement: S5 Table — (PDF) [file pone.0298070.s005.pdf]

# Supplementary Table 5

Heterogeneity and pleiotropic analysis

| Exposure | Outcome | Q-statistics | | Pleiotropic test | |
|---|---|---|---|---|---|
| | | MR Egger | IVW | egger_intercept | pval |
| T2DM | Concentration of small LDL particles | Q=1095.14725391471 p=1.30220083384947E-192 | Q=1095.77414071586 p=4.29902620560193E-192 | -1.19E-03 | 0.858551775 |
| | Cholesterol in small LDL | Q=1305.53704302898 p=3.06241814258402E-236 | Q=1309.35686929938 p=2.38518980919161E-236 | -2.95E-03 | 0.687179802 |
| Fasting glucose | Concentration of small LDL particles | Q=380.384190044291 p=2.67170019954959E-47 | Q=390.974451640394 p=7.68574506784542E-49 | 3.57E-03 | 0.193745404 |
| | Cholesterol in small LDL | Q=396.996351911585 p=2.36092383457886E-50 | Q=405.437587765317 p=1.67261930287515E-51 | 3.19E-03 | 0.255309842 |
| Fasting insulin | Concentration of small LDL particles | Q=288.28466924004 p=4.00147520957703E-41 | Q=305.97675449562 p=4.63633387803972E-44 | 1.00E-02 | 0.145884689 |
| | Cholesterol in small LDL | Q=255.97924876315 p=5.58496387579094E-35 | Q=273.351501732576 p=7.94997622939732E-38 | 9.94E-03 | 0.126786455 |
| Essential (primary) hypertension | Concentration of small LDL particles | Q=153.187817335591 p=1.06494067626756E-08 | Q=154.48811852205 p=0.0000000112057394943 | 2.47E-03 | 0.453412421 |
| | Cholesterol in small LDL | Q=119.719911984519 p=0.0000805571001868944 | Q=119.793860310348 p=0.000108019187247974 | -5.88E-04 | 0.839414308 |
| HDL cholesterol | Concentration of small LDL particles | Q=4529.82111928503 p=0 | Q=4544.53381337873 p=0 | 1.61E-03 | 0.289842116 |
| | Cholesterol in small LDL | Q=4664.81053695056 | Q=4712.89535851247 | 2.91E-03 | 0.059790424 |

| | | | | | |
|---|---|---|---|---|---|
| | | p=0 | p=0 | | |
| LDL cholesterol | Concentration of small LDL particles | Q=961.772166529509<br>p=7.37754676494999E-112 | Q=965.297756365452<br>p=4.12360671775696E-112 | 1.12E-03 | 0.43507782 |
| | Cholesterol in small LDL | Q=732.529495257396<br>p=8.33341436787416E-72 | Q=736.435165290423<br>p=3.85652092826063E-72 | -1.18E-03 | 0.346731093 |
| Triglycerides | Concentration of small LDL particles | Q=1624.7147880092<br>p=3.30999430322059E-181 | Q=1627.50912641207<br>p=2.46987337875193E-181 | 7.63E-04 | 0.474606873 |
| | Cholesterol in small LDL | Q=1672.21385392645<br>p=1.13495770076208E-189 | Q=1677.99568188668<br>p=2.49443182453321E-190 | 1.10E-03 | 0.310897207 |
