# Supplementary material for: Small dense low density lipoprotein predominance in patients with type 2 diabetes mellitus using Mendelian randomization
Source: PLoS One. 2024 Feb 8;19(2):e0298070. doi: 10.1371/journal.pone.0298070 (PMC10852223; doi:10.1371/journal.pone.0298070)
Supplement: S6 Table — (PDF) [file pone.0298070.s006.pdf]

Supplementary Table 6

Results of MVMR analysis (lipid profile as exposure, sd-LDL level as outcome)

Outcome	Method	Exposure	Estimate	Std Error	95%CI		p-value
Concentration of small LDL particles	IVW	HDL cholesterol	-0.048	0.019	-0.085	-0.011	0.011
		LDL cholesterol	0.813	0.022	0.77	0.855	0
		Triglycerides	0.221	0.021	0.179	0.262	0
	MR-Egger	HDL cholesterol	-0.037	0.023	-0.083	0.008	0.104
		LDL cholesterol	0.813	0.022	0.771	0.856	0
		Triglycerides	0.218	0.022	0.176	0.26	0
	MR-Lasso	HDL cholesterol	-0.105	0.013	-0.131	-0.078	0
		LDL cholesterol	0.874	0.015	0.845	0.903	0
		Triglycerides	0.157	0.015	0.128	0.186	0
	Median	HDL cholesterol	-0.087	0.022	-0.13	-0.045	0
		LDL cholesterol	0.862	0.026	0.812	0.913	0
		Triglycerides	0.163	0.025	0.114	0.212	0
Cholesterol in small LDL	IVW	HDL cholesterol	-0.058	0.018	-0.094	-0.022	0.002
		LDL cholesterol	0.871	0.021	0.83	0.912	0
		Triglycerides	0.069	0.021	0.029	0.11	0.001
	MR-Egger	HDL cholesterol	-0.051	0.022	-0.095	-0.007	0.022
		LDL cholesterol	0.871	0.021	0.83	0.912	0
		Triglycerides	0.068	0.021	0.027	0.109	0.001
	MR-Lasso	HDL cholesterol	-0.035	0.015	-0.066	-0.005	0.022
		LDL cholesterol	0.89	0.017	0.856	0.924	0
		Triglycerides	0.051	0.016	0.019	0.083	0.002
	Median	HDL cholesterol	-0.066	0.025	-0.116	-0.016	0.009
		LDL cholesterol	0.895	0.029	0.839	0.951	0
		Triglycerides	0.018	0.029	-0.038	0.074	0.529
