# Supplementary material for: Small dense low density lipoprotein predominance in patients with type 2 diabetes mellitus using Mendelian randomization
Source: PLoS One. 2024 Feb 8;19(2):e0298070. doi: 10.1371/journal.pone.0298070 (PMC10852223; doi:10.1371/journal.pone.0298070)
Supplement: S7 Table — (PDF) [file pone.0298070.s007.pdf]

Supplementary Table 7

MR-PRESSO validation of MVMR analysis (lipid profile as exposure, sd-LDL level as outcome)

Outcome	Exposure	MR Analysis	Causal Estimate	Sd	T-stat	P-value
Concentration of small LDL particles	HDL cholesterol	Raw	-0.04777497	0.01885769	-2.533447	1.17E-02
	LDL cholesterol	Raw	0.8126397	0.02174683	37.368197	4.18E-131
	Triglycerides	Raw	0.22085483	0.0212463	10.394977	1.65E-22
Cholesterol in small LDL	HDL cholesterol	Raw	-0.05778309	0.01824305	-3.167403	1.66E-03
	LDL cholesterol	Raw	0.87073664	0.0210352	41.394265	5.57E-145
	Triglycerides	Raw	0.06941982	0.02055344	3.377528	8.05E-04
