# Supplementary material for: Small dense low density lipoprotein predominance in patients with type 2 diabetes mellitus using Mendelian randomization
Source: PLoS One. 2024 Feb 8;19(2):e0298070. doi: 10.1371/journal.pone.0298070 (PMC10852223; doi:10.1371/journal.pone.0298070)
Supplement: S8 Table — (PDF) [file pone.0298070.s008.pdf]

Supplementary Table 8

Information about the MVMR (lipid profile as exposure, sd-LDL level as outcome)

Outcome	Exposure	Heterogeneity (Q)		Egger_intercept(p-value)	F
		IVW(p-value)	MR-egger(p-value)		
Concentration of small LDL particles	HDL cholesterol	0	0	0.437	51.46568919
	LDL cholesterol				45.57366978
	Triglycerides				40.90451244
Cholesterol in small LDL	HDL cholesterol	0	0	0.609	51.46568919
	LDL cholesterol				45.57366978
	Triglycerides				40.90451244
