# Supplementary material for: Small dense low density lipoprotein predominance in patients with type 2 diabetes mellitus using Mendelian randomization
Source: PLoS One. 2024 Feb 8;19(2):e0298070. doi: 10.1371/journal.pone.0298070 (PMC10852223; doi:10.1371/journal.pone.0298070)
Supplement: S9 Table — (PDF) [file pone.0298070.s009.pdf]

Supplementary Table 9

Results of MVMR analysis (T2DM and glucose characteristics as exposures and hypertension and lipid characteristics as outcomes)

Outcome	Method	Exposure	Estimate	Std Error	95%CI		p-value
Essential (primary) hypertension	IVW	Fasting glucose	-0.018	0.009	-0.036	0	0.056
		Fasting insulin	0.042	0.014	0.014	0.069	0.003
		T2DM	0.009	0.002	0.004	0.013	0
	MR-Egger	Fasting glucose	-0.013	0.012	-0.035	0.01	0.281
		Fasting insulin	0.046	0.015	0.016	0.076	0.003
		T2DM	0.01	0.002	0.005	0.014	0
	MR-Lasso	Fasting glucose	-0.008	0.007	-0.021	0.005	0.217
		Fasting insulin	0.071	0.01	0.052	0.09	0
		T2DM	0.008	0.002	0.005	0.011	0
	Median	Fasting glucose	-0.013	0.01	-0.031	0.006	0.186
		Fasting insulin	0.068	0.014	0.041	0.094	0
		T2DM	0.009	0.002	0.005	0.014	0
HDL cholesterol	IVW	Fasting glucose	0.076	0.089	-0.097	0.250	0.389
		Fasting insulin	-0.904	0.13	-1.159	-0.649	0
		T2DM	-0.058	0.022	-0.102	-0.015	0.008
	MR-Egger	Fasting glucose	0.094	0.109	-0.120	0.308	0.389
		Fasting insulin	-0.889	0.141	-1.165	-0.613	0
		T2DM	-0.056	0.024	-0.102	-0.010	0.017
	MR-Lasso	Fasting glucose	0.003	0.018	-0.032	0.039	0.86
		Fasting insulin	-0.795	0.043	-0.879	-0.71	0
		T2DM	-0.045	0.005	-0.054	-0.035	0
	Median	Fasting glucose	-0.008	0.033	-0.073	0.057	0.814
		Fasting insulin	-0.880	0.079	-1.035	-0.726	0
		T2DM	-0.055	0.01	-0.075	-0.036	0
LDL cholesterol	IVW	Fasting glucose	0.076	0.119	-0.157	0.309	0.524
		Fasting insulin	-0.139	0.175	-0.481	0.203	0.427
		T2DM	-0.05	0.03	-0.108	0.008	0.09
	MR-Egger	Fasting glucose	0.046	0.146	-0.241	0.333	0.755
		Fasting insulin	-0.164	0.189	-0.535	0.207	0.386
		T2DM	-0.054	0.032	-0.116	0.008	0.087
	MR-Lasso	Fasting glucose	0.053	0.018	0.017	0.089	0.004
		Fasting insulin	0.06	0.032	-0.004	0.123	0.064
		T2DM	-0.029	0.005	-0.038	-0.02	0

		Fasting glucose	0.043	0.027	-0.009	0.095	0.102
	Median	Fasting insulin	0.051	0.048	-0.044	0.145	0.292
		T2DM	-0.028	0.007	-0.043	-0.014	0
		Fasting glucose	-0.106	0.122	-0.345	0.133	0.385
	IVW	Fasting insulin	0.456	0.179	0.106	0.807	0.011
		T2DM	0.066	0.03	0.007	0.126	0.029
		Fasting glucose	-0.241	0.149	-0.532	0.05	0.105
	MR-Egger	Fasting insulin	0.343	0.192	-0.034	0.719	0.074
Triglycerides		T2DM	0.049	0.032	-0.014	0.112	0.127
		Fasting glucose	0.084	0.02	0.044	0.123	0
	MR-Lasso	Fasting insulin	0.742	0.04	0.664	0.82	0
		T2DM	0.052	0.005	0.043	0.062	0
		Fasting glucose	0.08	0.033	0.015	0.144	0.016
	Median	Fasting insulin	0.815	0.068	0.682	0.948	0
		T2DM	0.063	0.01	0.043	0.082	0
