# Supplementary material for: Small dense low density lipoprotein predominance in patients with type 2 diabetes mellitus using Mendelian randomization
Source: PLoS One. 2024 Feb 8;19(2):e0298070. doi: 10.1371/journal.pone.0298070 (PMC10852223; doi:10.1371/journal.pone.0298070)
Supplement: S10 Table — (PDF) [file pone.0298070.s010.pdf]

Supplementary Table 10

MR-PRESSO validated the results of MVMR analysis (T2DM and glucose characteristics as exposure, hypertension and lipid characteristics as outcome)

Outcome	Exposure	MR Analysis	Causal Estimate	Sd	T-stat	P-value
Essential (primary) hypertension	Fasting glucose	Raw	-0.017829089	0.00934531	-1.9078114	0.058931265
	Fasting insulin	Raw	0.041621388	0.014149617	2.9415205	0.003956593
	T2DM	Raw	0.008944292	0.002293755	3.8994096	0.000163365
HDL cholesterol	Fasting glucose	Raw	0.07642061	0.08862756	0.8622668	0.3903509
	Fasting insulin	Raw	-0.90376518	0.13000059	-6.9520083	2.38769E-10
	T2DM	Raw	-0.05843199	0.02203731	-2.6515023	0.009154211
LDL cholesterol	Fasting glucose	Raw	0.075852298	0.11905671	0.6371106	0.52533018
	Fasting insulin	Raw	-0.138715638	0.17458085	-0.7945639	0.42851825
	T2DM	Raw	-0.050206901	0.02961166	-1.6955115	0.09270801
Triglycerides	Fasting glucose	Raw	-0.10604376	0.12202581	-0.8690272	0.386658169
	Fasting insulin	Raw	0.45636334	0.17894489	2.5503011	0.01208938
	T2DM	Raw	0.06619583	0.03037071	2.1795942	0.031344152
