# Supplementary material for: Small dense low density lipoprotein predominance in patients with type 2 diabetes mellitus using Mendelian randomization
Source: PLoS One. 2024 Feb 8;19(2):e0298070. doi: 10.1371/journal.pone.0298070 (PMC10852223; doi:10.1371/journal.pone.0298070)
Supplement: S11 Table — (PDF) [file pone.0298070.s011.pdf]

Supplementary Table 11

Information about the MVMR (T2DM and glucose characteristics as exposure, hypertension and lipid characteristics as outcome)

Outcome	Exposure	Heterogeneity (Q)		Egger_intercept(p-value)	Value of F
		IVW(p-value)	MR-egger(p-value)		
Essential (primary) hypertension	Fasting glucose	0	0	0.446	34.87087092
	Fasting insulin				16.59725163
	T2DM				16.42447153
HDL cholesterol	Fasting glucose	0	0	0.781	32.09635084
	Fasting insulin				16.92216679
	T2DM				15.85227146
LDL cholesterol	Fasting glucose	0	0	0.722	32.17098846
	Fasting insulin				16.92915919
	T2DM				15.87008307
Triglycerides	Fasting glucose	0	0	0.117	32.09635084
	Fasting insulin				16.92216679
	T2DM				15.85227146
