# Supplementary material for: Small dense low density lipoprotein predominance in patients with type 2 diabetes mellitus using Mendelian randomization
Source: PLoS One. 2024 Feb 8;19(2):e0298070. doi: 10.1371/journal.pone.0298070 (PMC10852223; doi:10.1371/journal.pone.0298070)

Supplementary Figure 1

Scattered point plot, exposure: T2DM, outcome: concentration of small LDL particles.

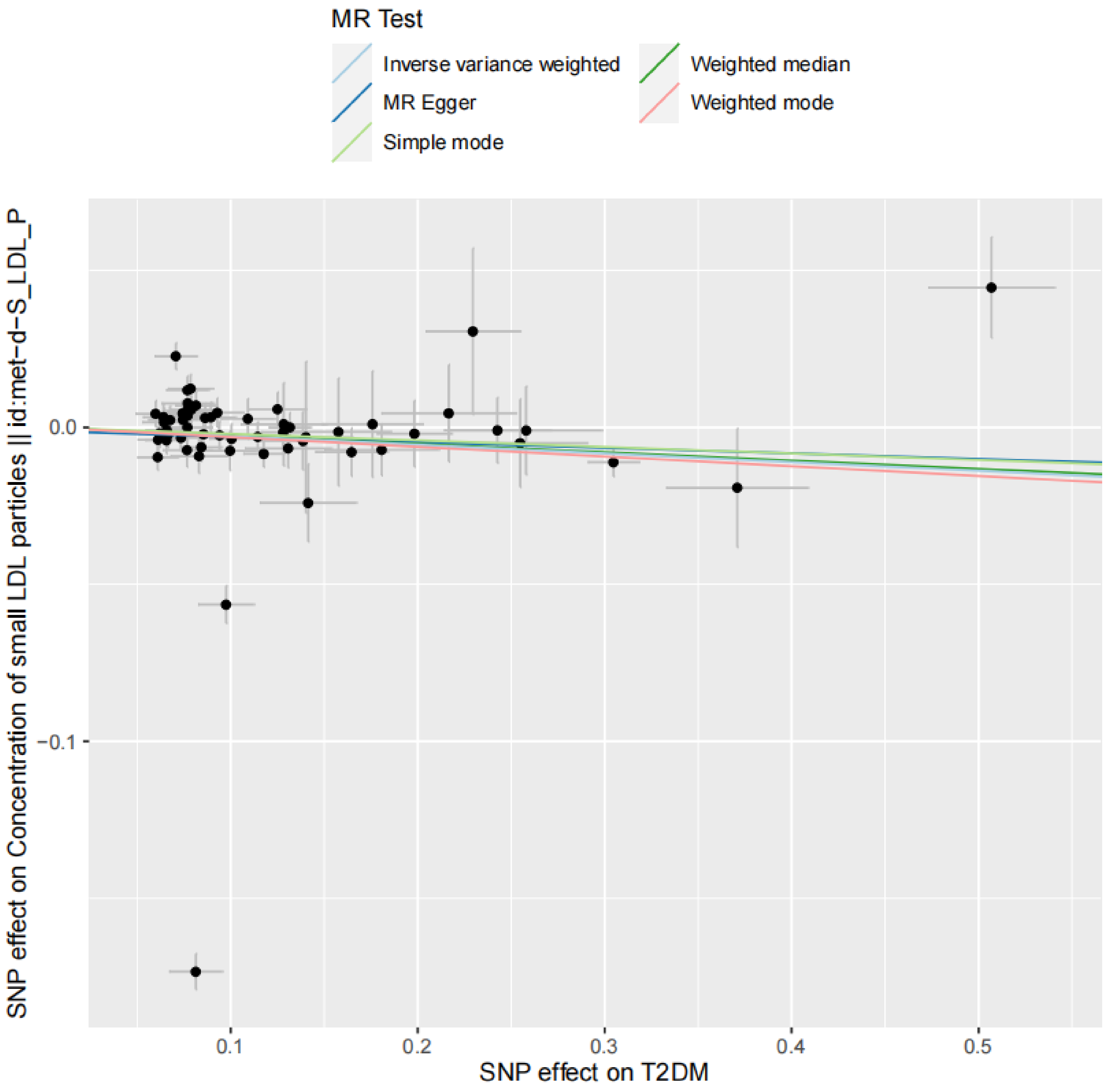

Supplementary Figure 2

Scattered point plot, exposure: T2DM, outcome: cholesterol in small LDL.

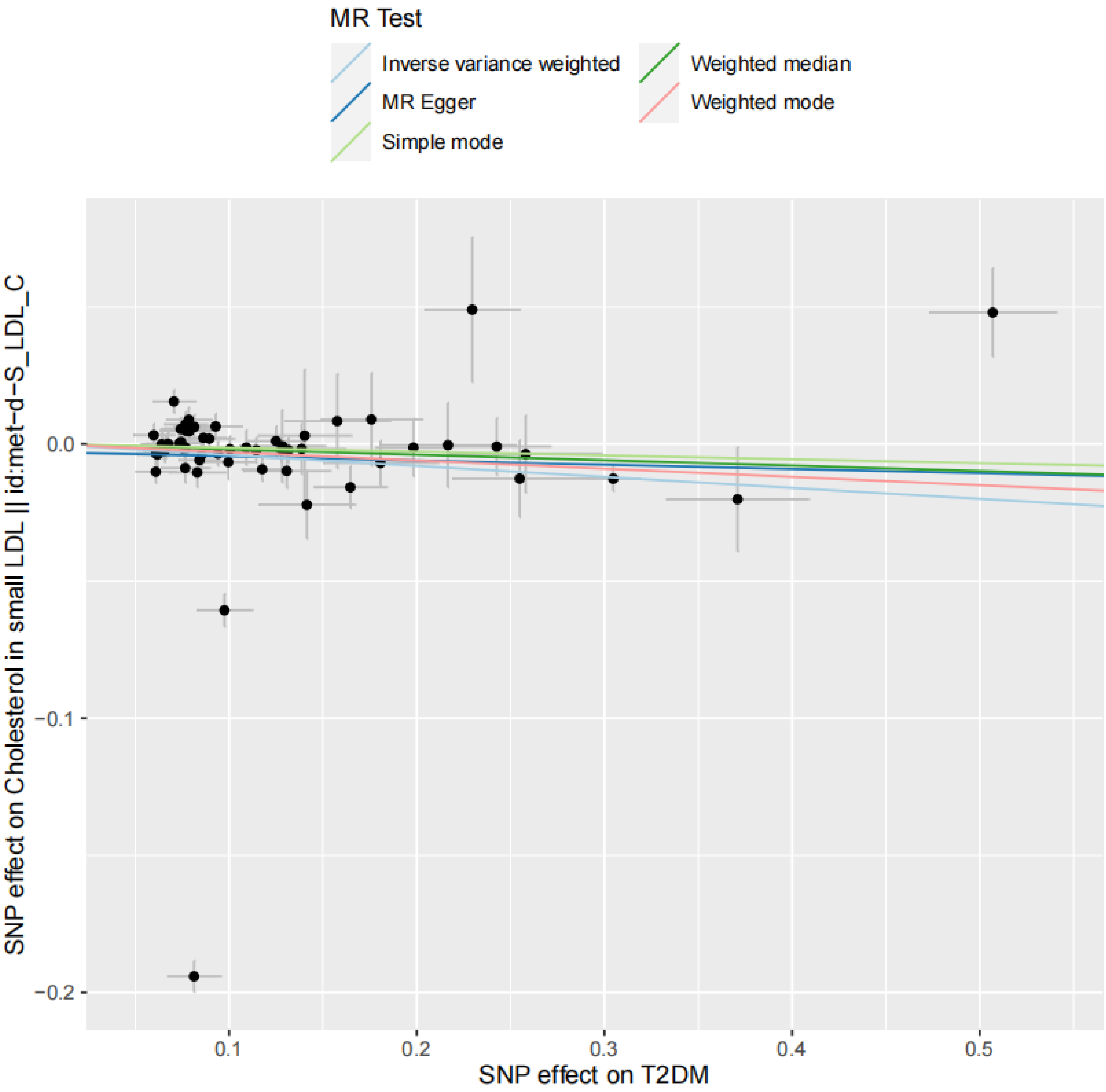

Supplementary Figure 3

Scattered point plot, exposure: fasting glucose, outcome: concentration of small LDL particles.

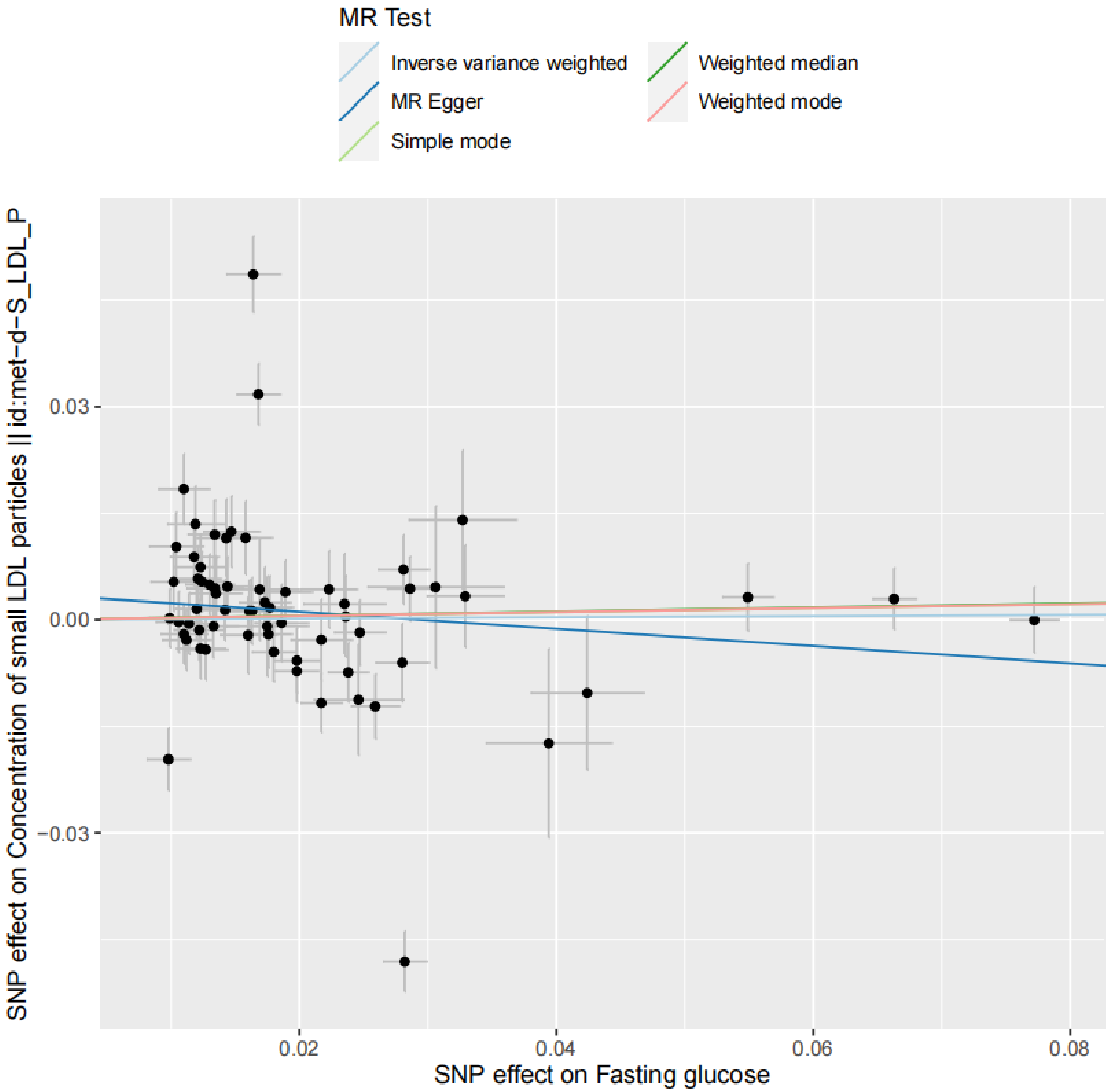

Supplementary Figure 4

Scattered point plot, exposure: fasting glucose, outcome: cholesterol in small LDL.

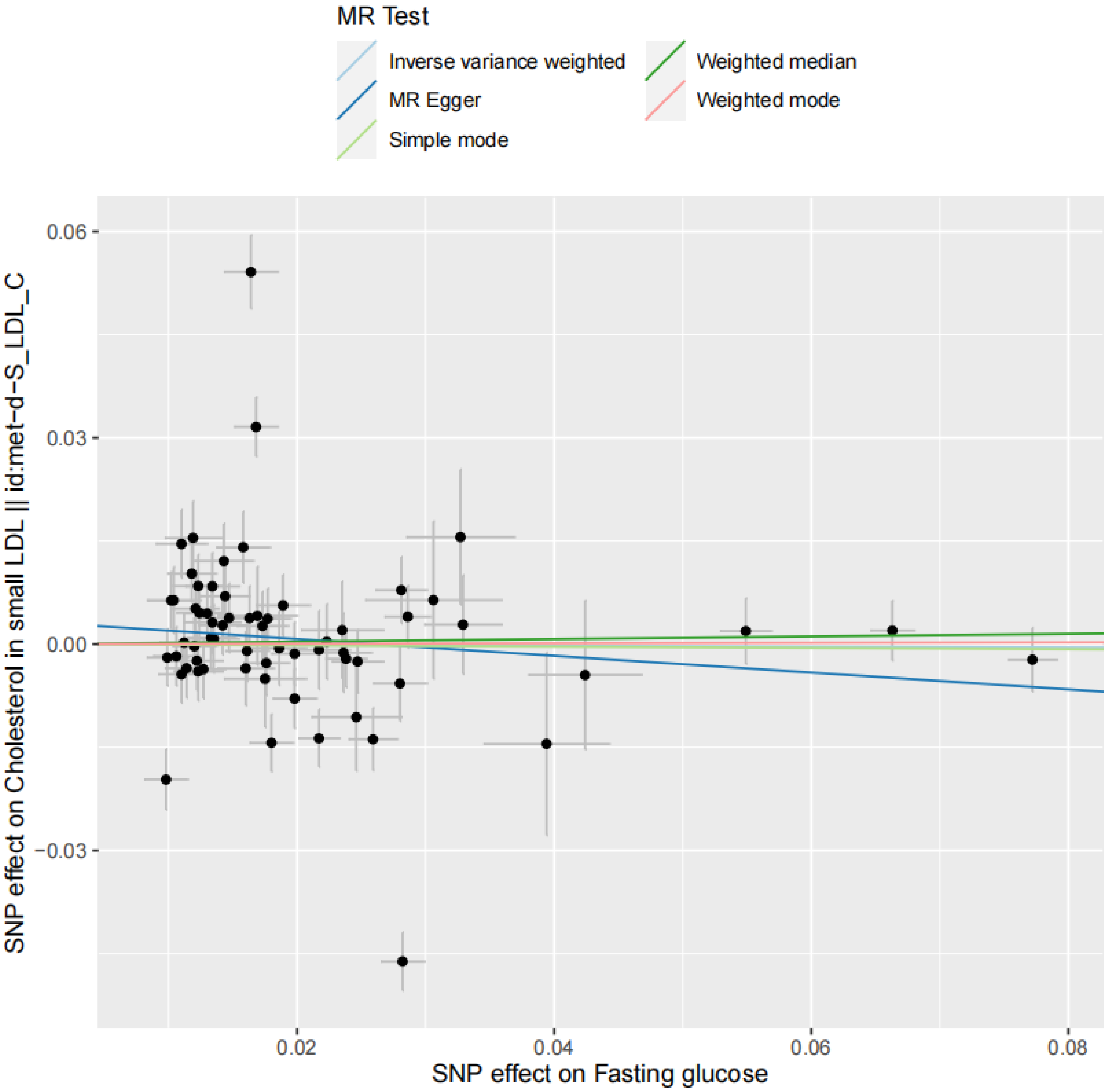

Supplementary Figure 5

Scattered point plot, exposure: fasting insulin, outcome: concentration of small LDL particles.

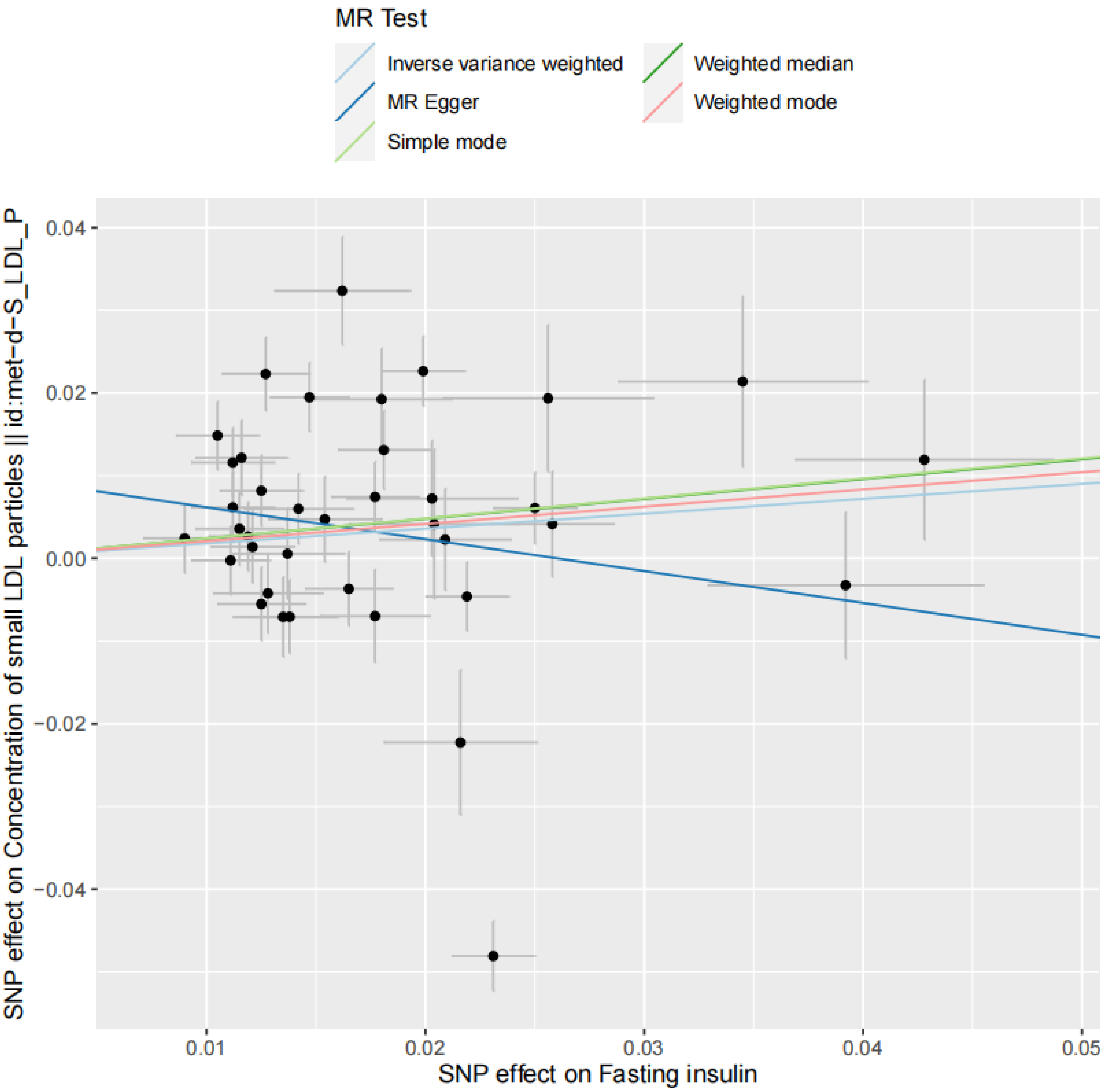

Supplementary Figure 6

Scattered point plot, exposure: fasting insulin, outcome: cholesterol in small LDL.

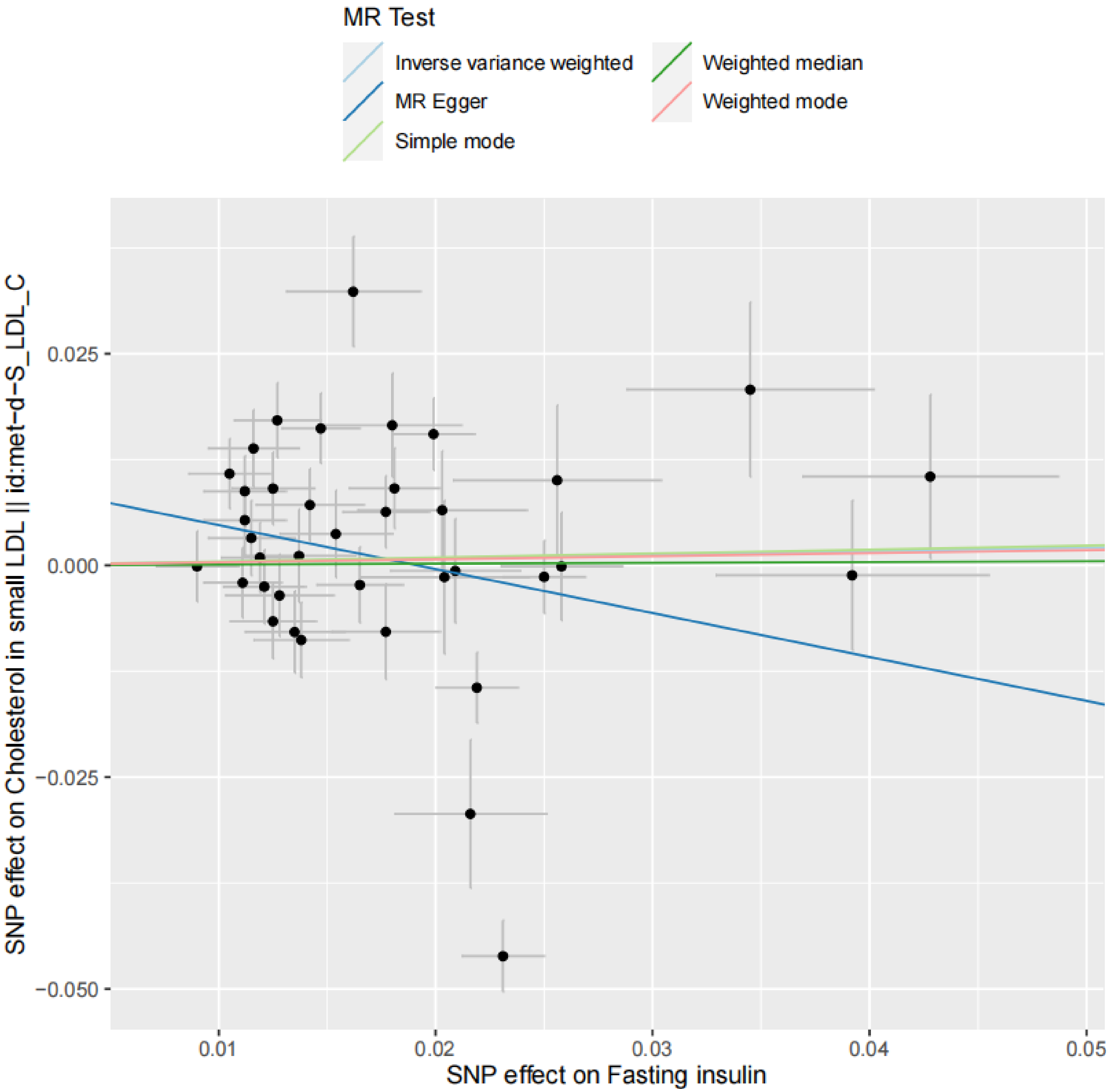

Supplementary Figure 7

Funnel plot, exposure: T2DM, outcome: concentration of small LDL particles.

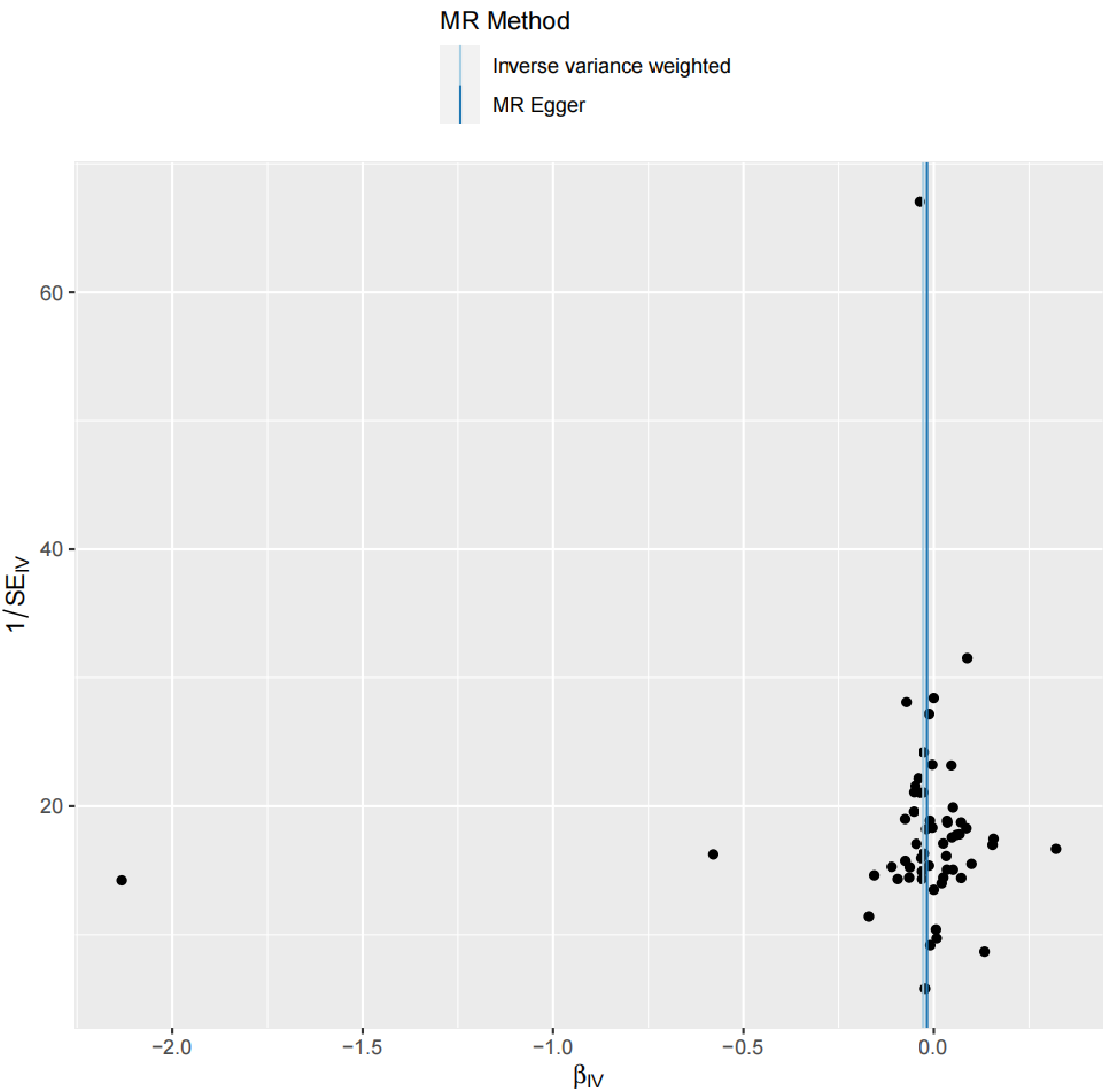

Supplementary Figure 8

Funnel plot, exposure: T2DM, outcome: cholesterol in small LDL.

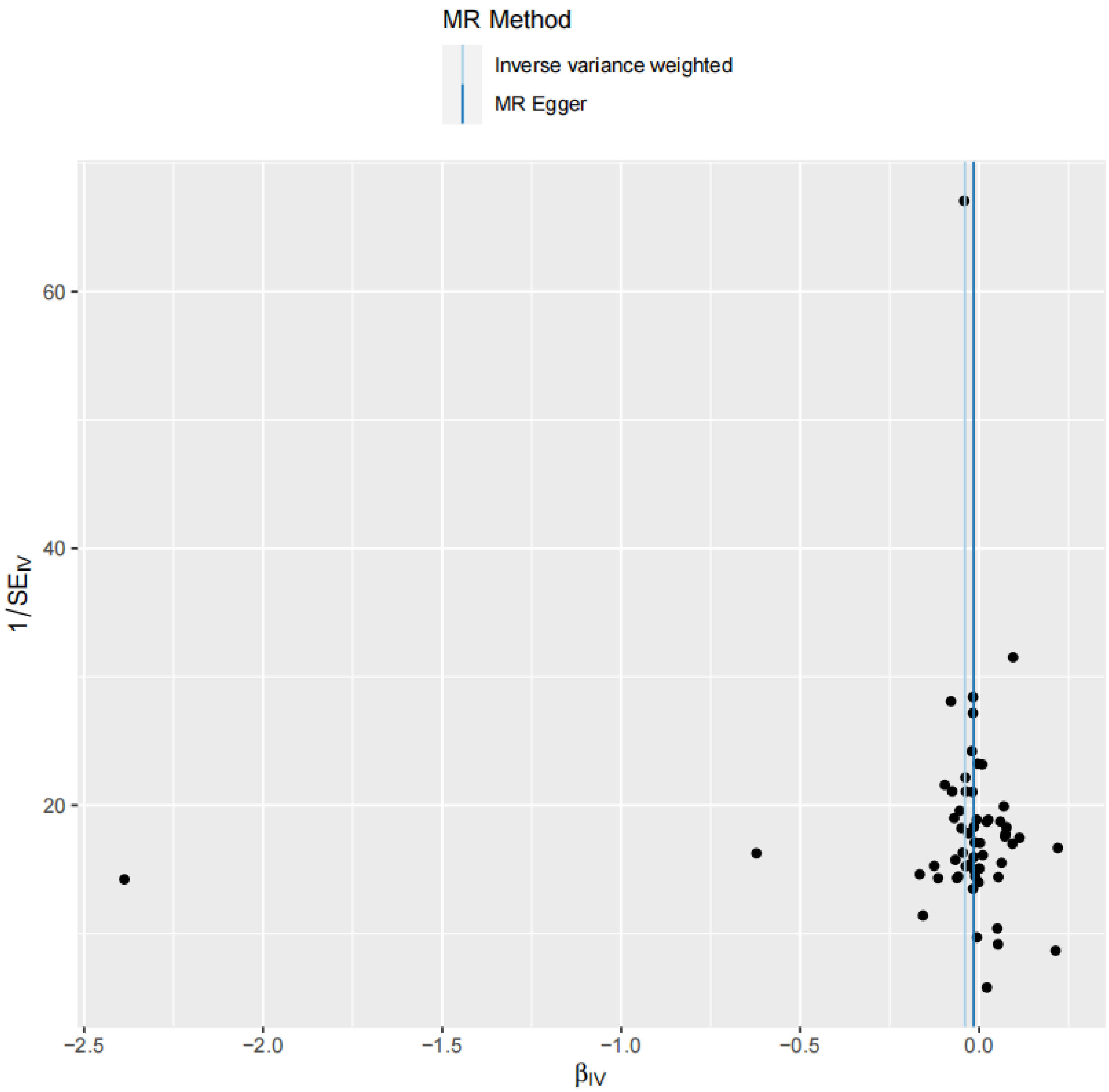

Supplementary Figure 9

Funnel plot, exposure: fasting glucose, outcome: concentration of small LDL particles.

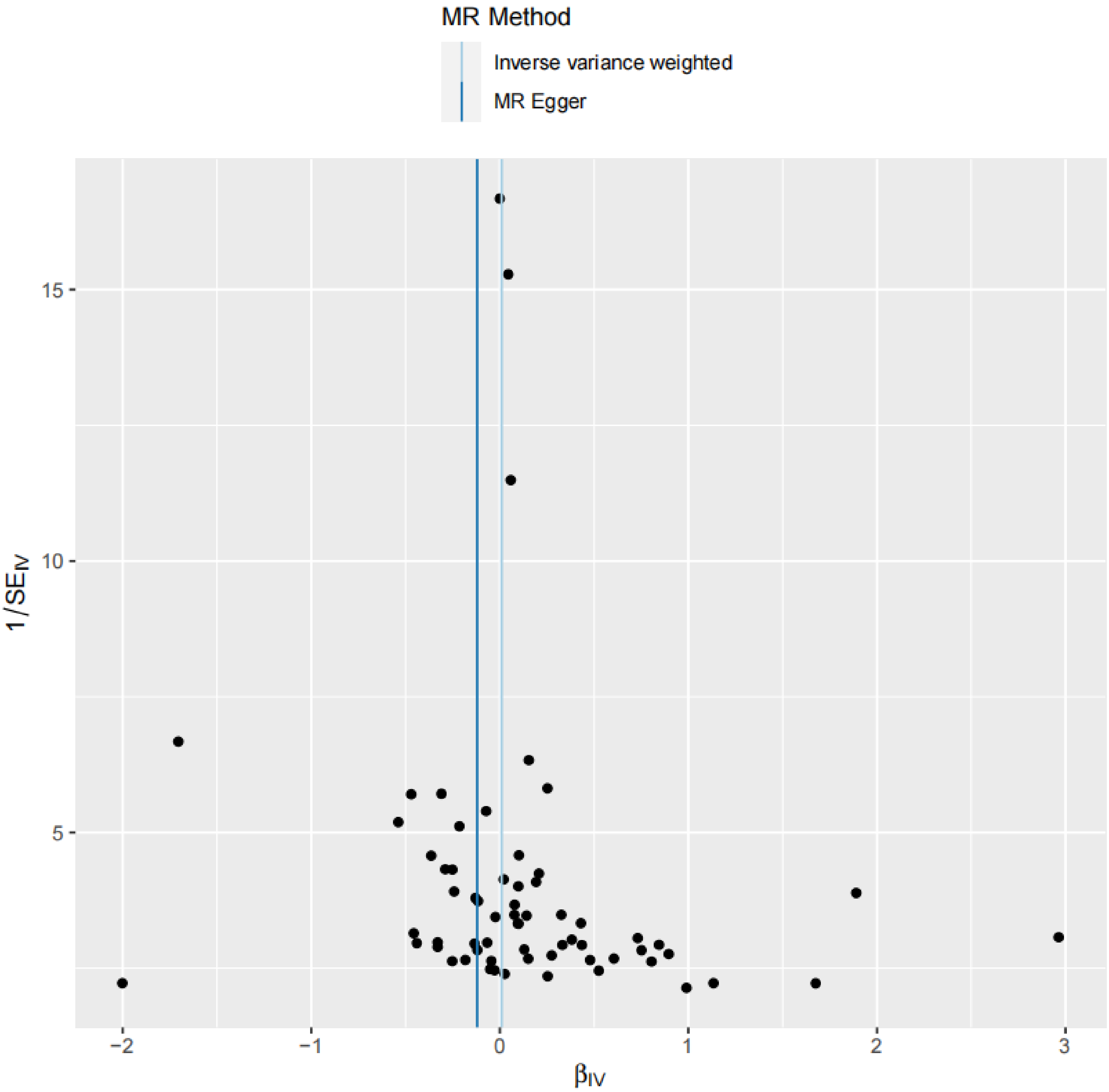

Supplementary Figure 10

Funnel plot, exposure: fasting glucose, outcome: cholesterol in small LDL.

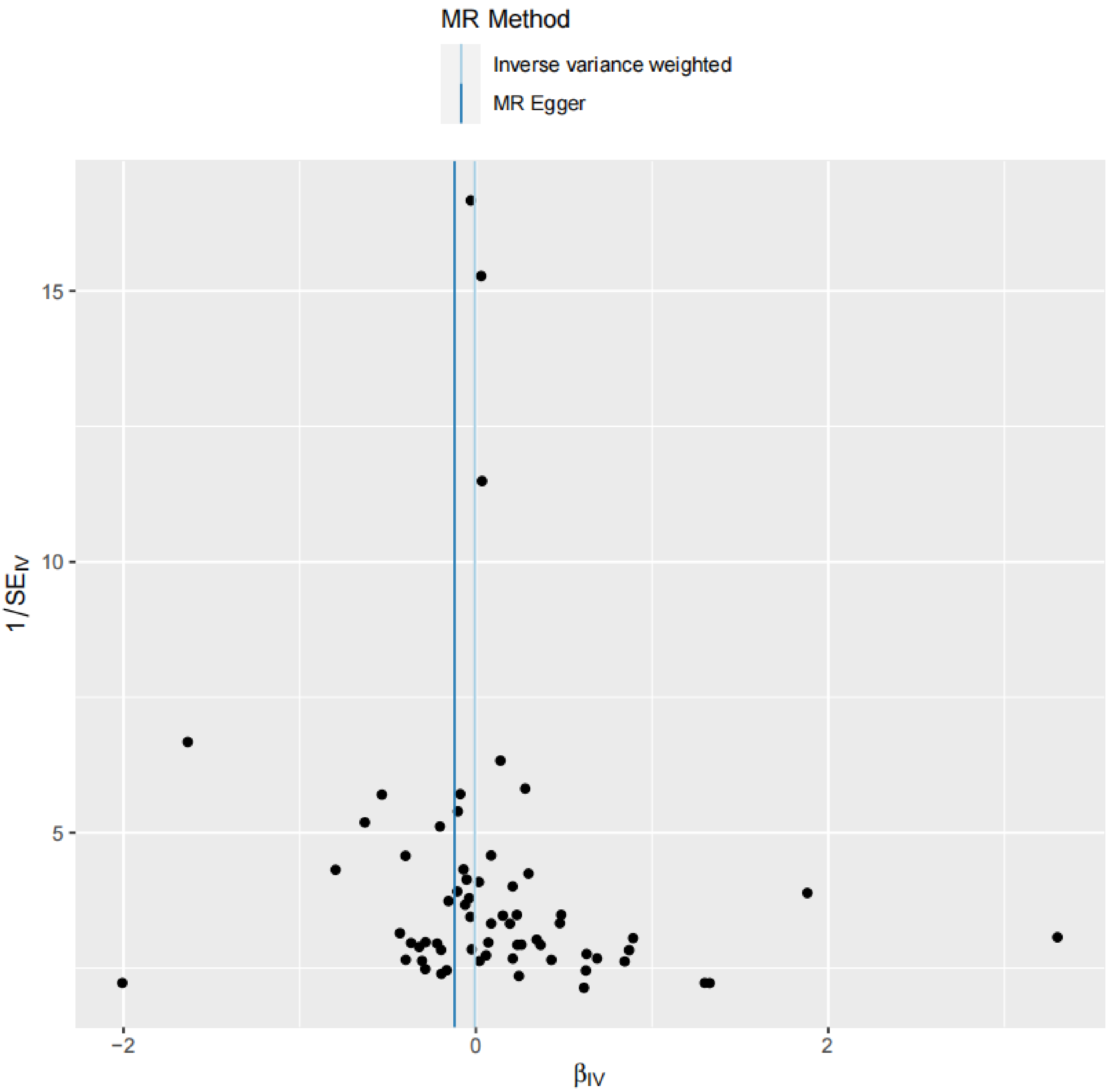

Supplementary Figure 11

Funnel plot, exposure: fasting insulin, outcome: concentration of small LDL particles.

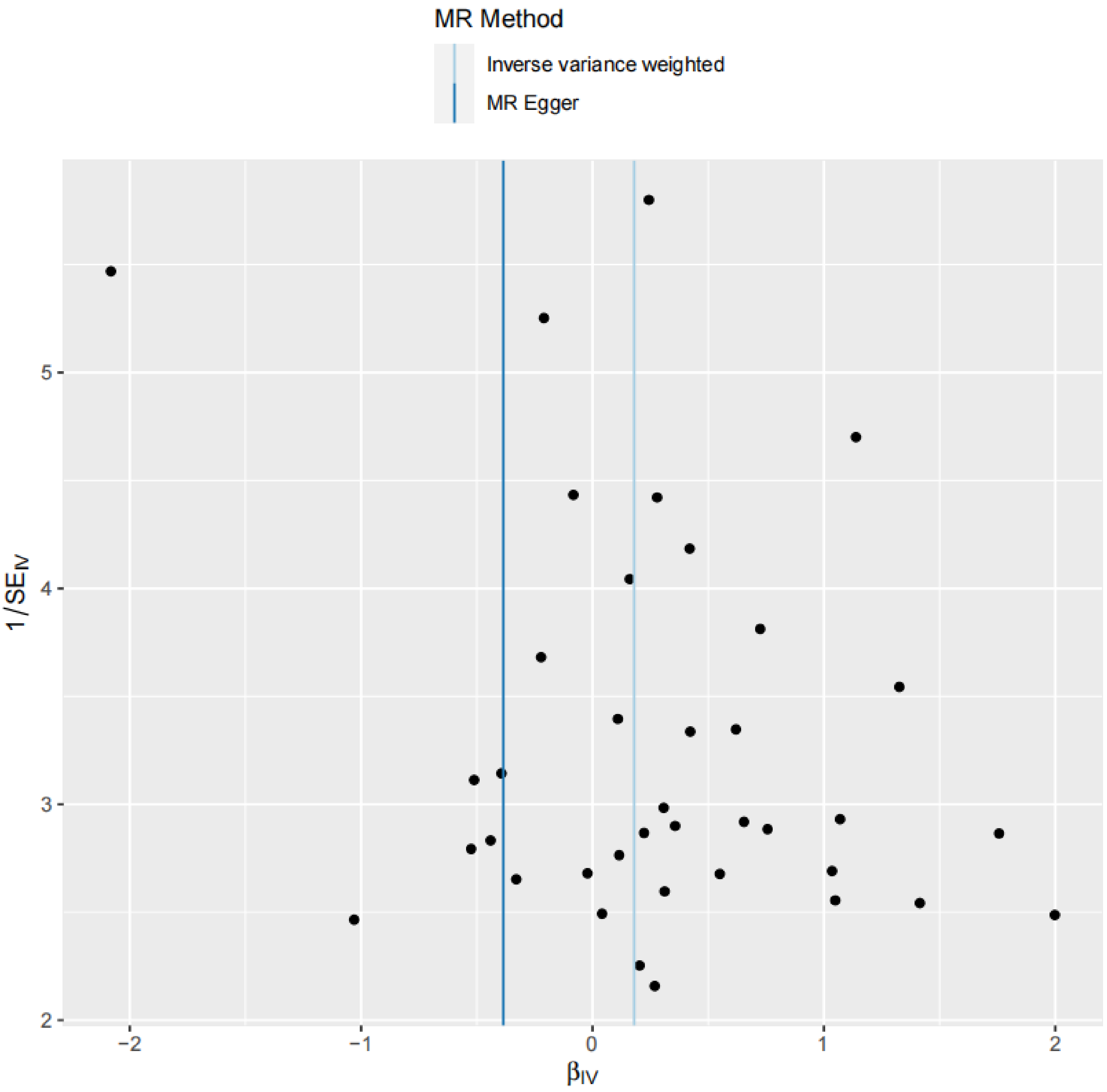

Supplementary Figure 12

Funnel plot, exposure: fasting insulin, outcome: cholesterol in small LDL.

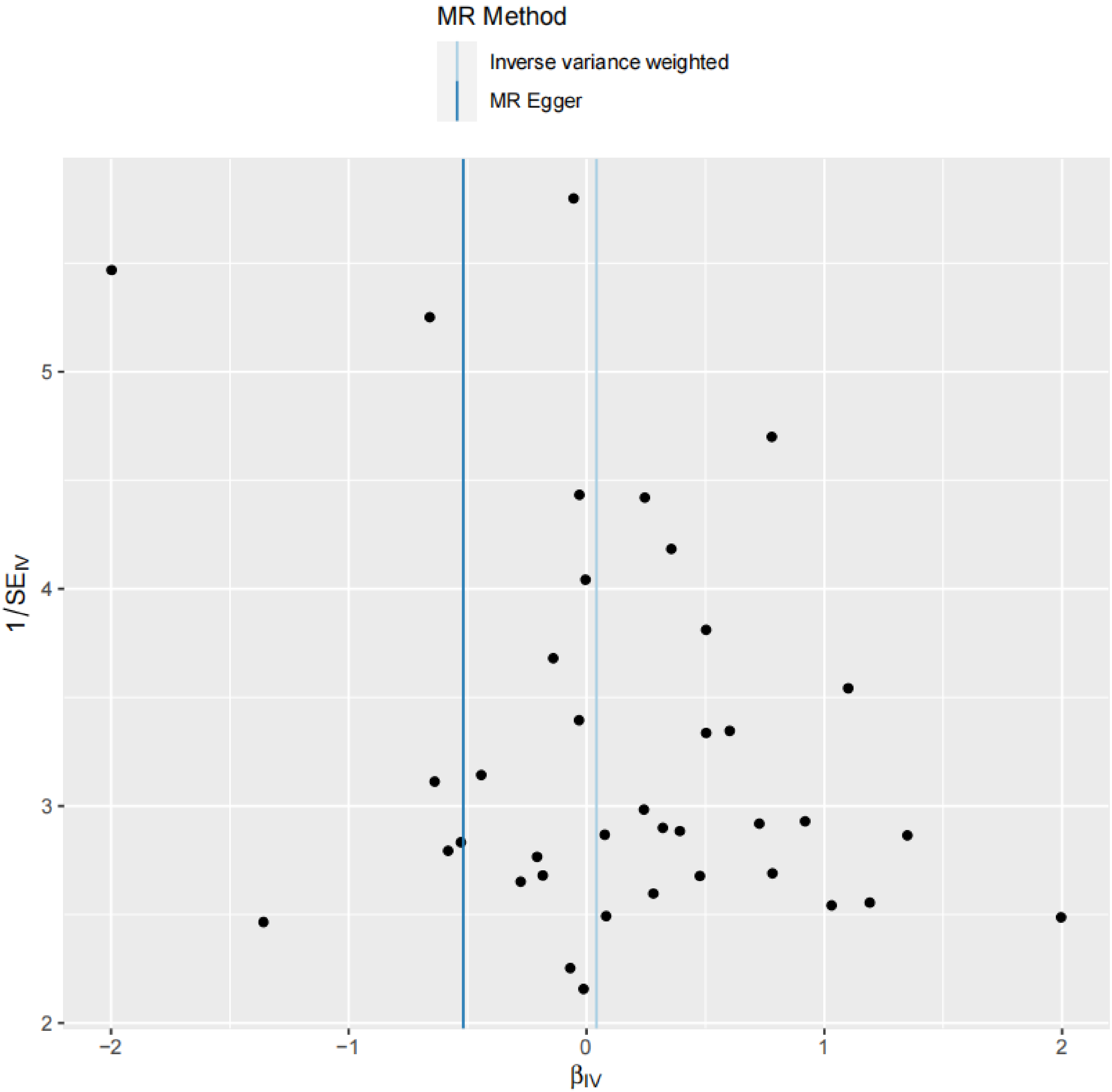

Supplementary Figure 13

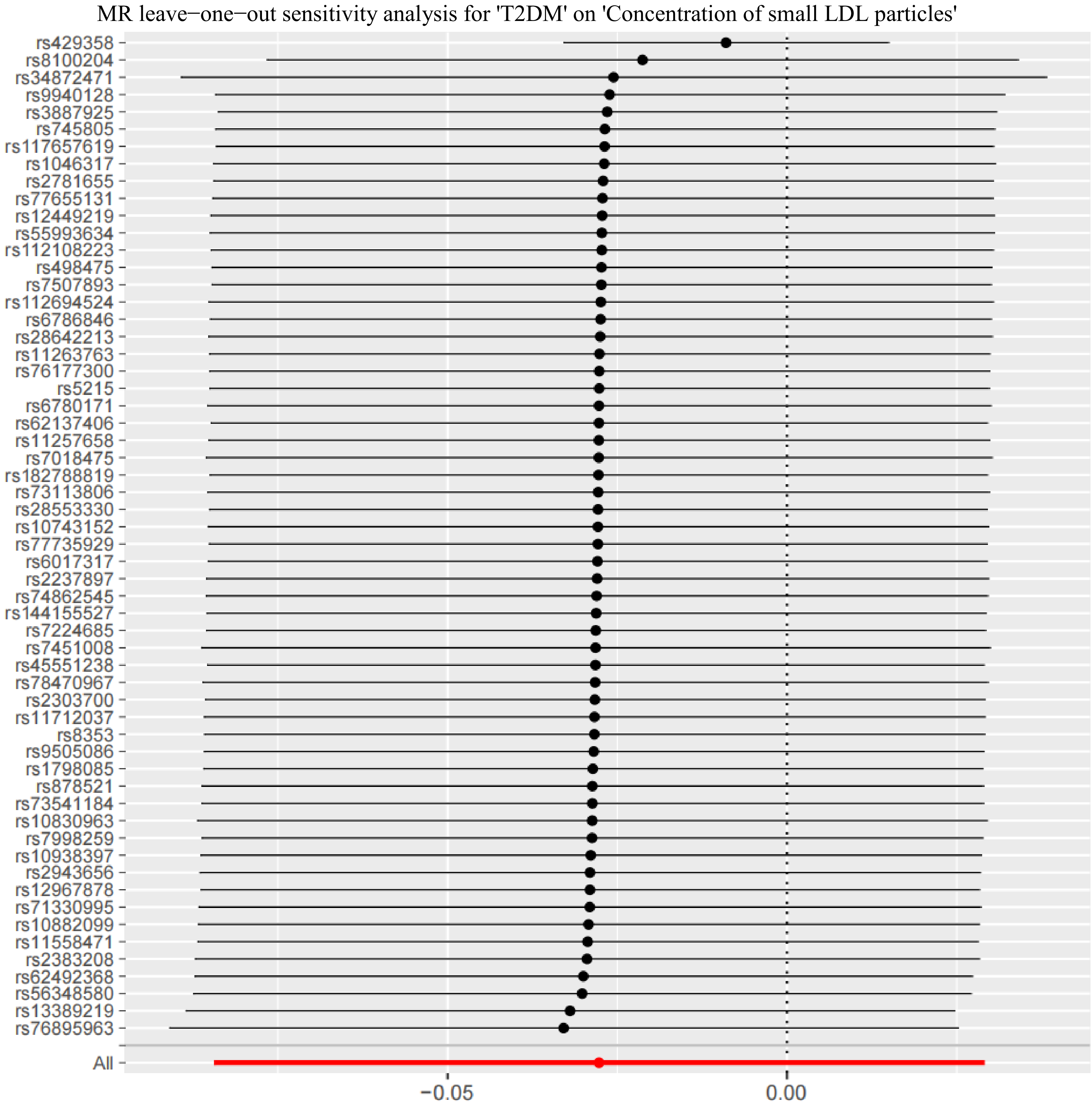

Supplementary Figure 14

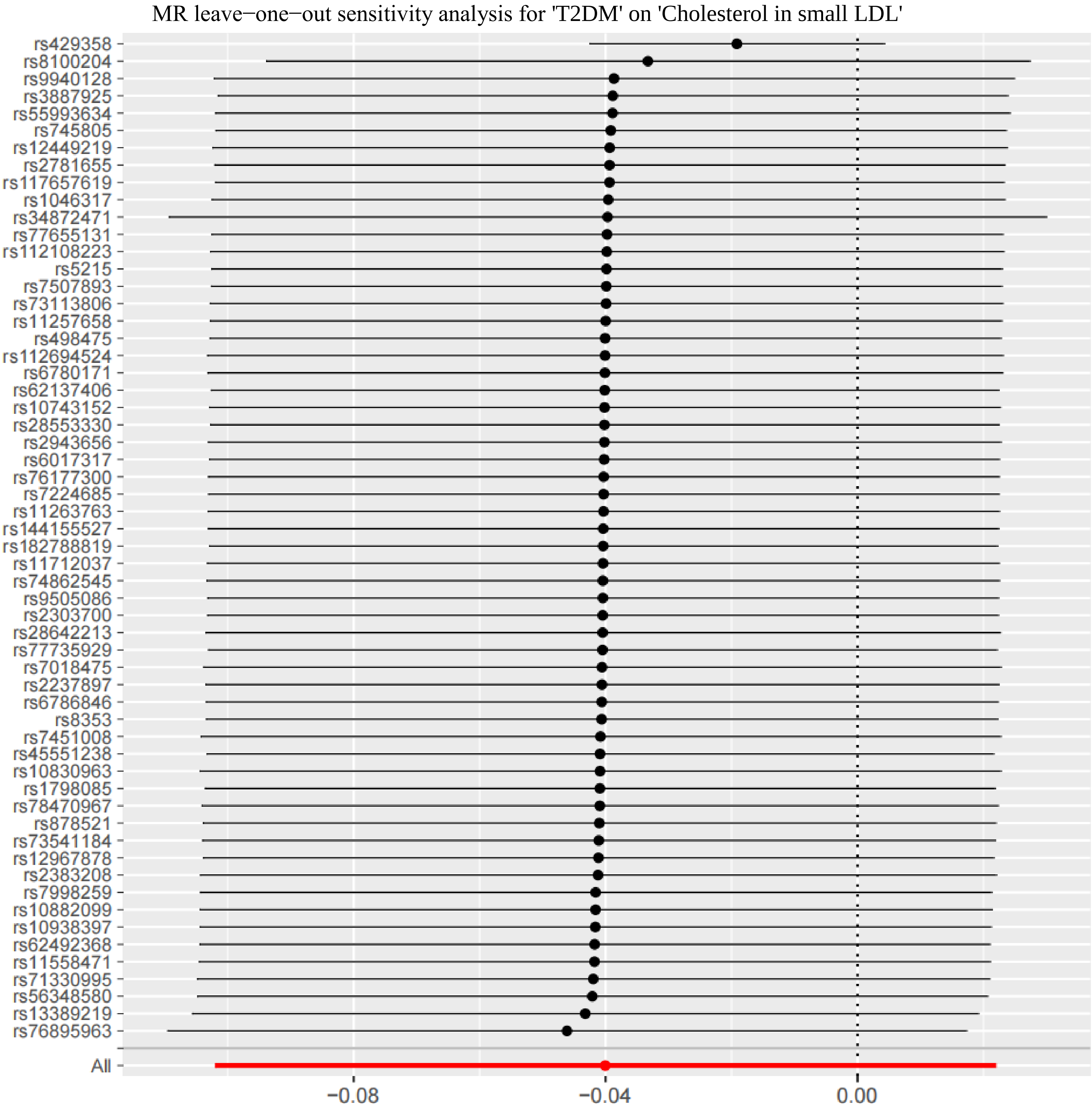

Supplementary Figure 15

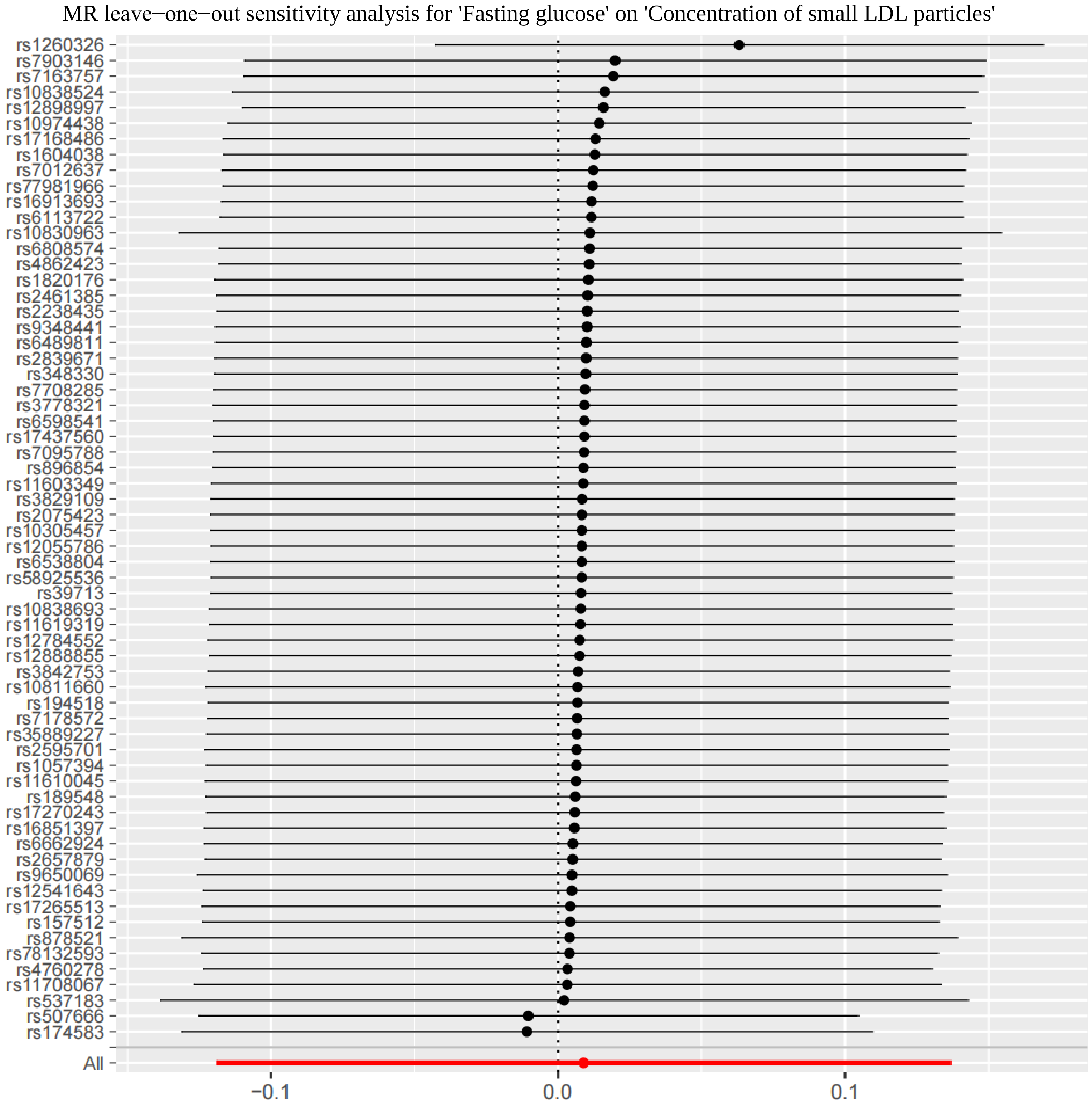

Supplementary Figure 16

MR leave−one−out sensitivity analysis for 'Fasting glucose' on 'Cholesterol in small LDL'

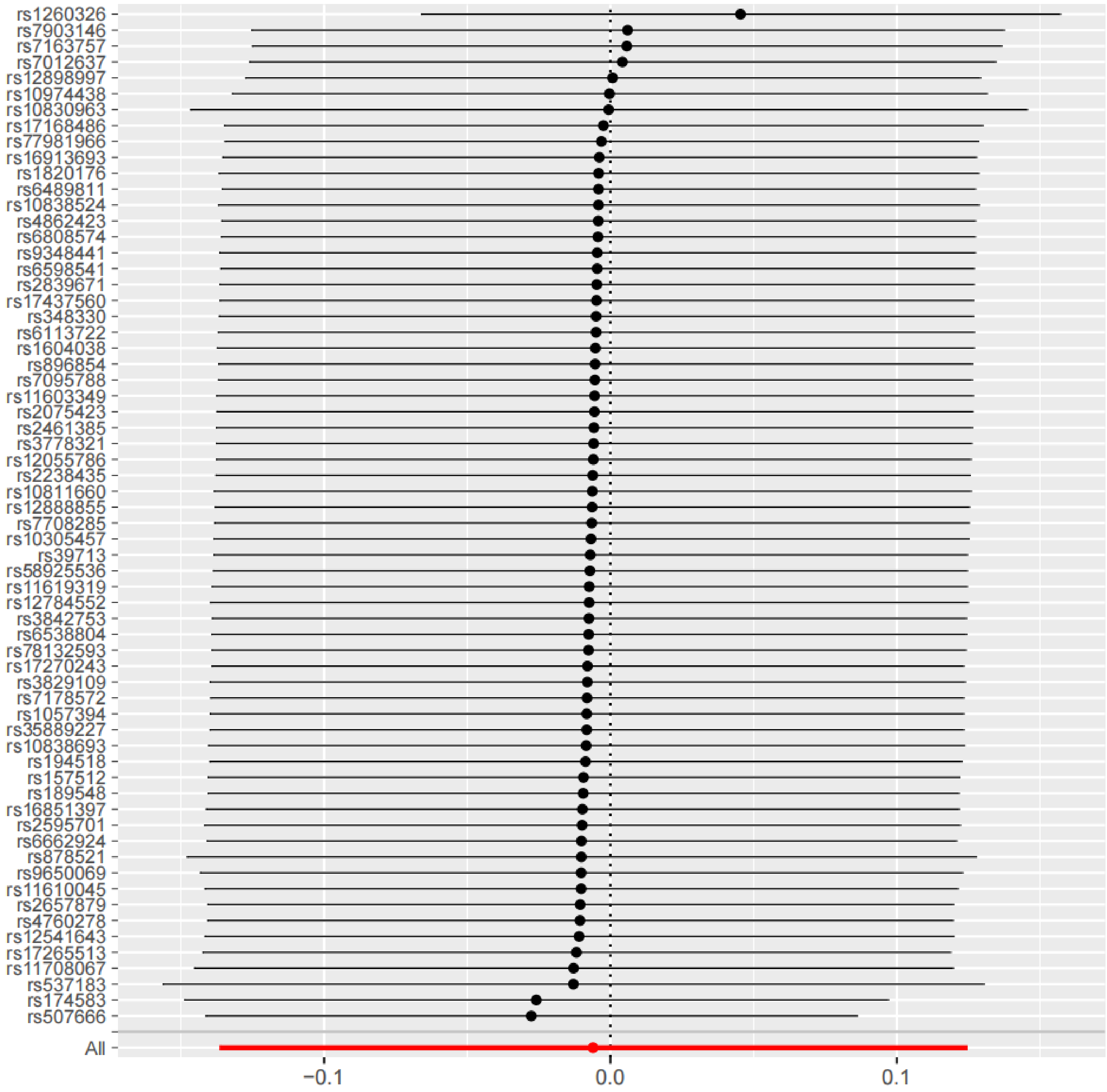

Supplementary Figure 17

MR leave−one−out sensitivity analysis for 'Fasting insulin' on 'Concentration of small LDL particles'

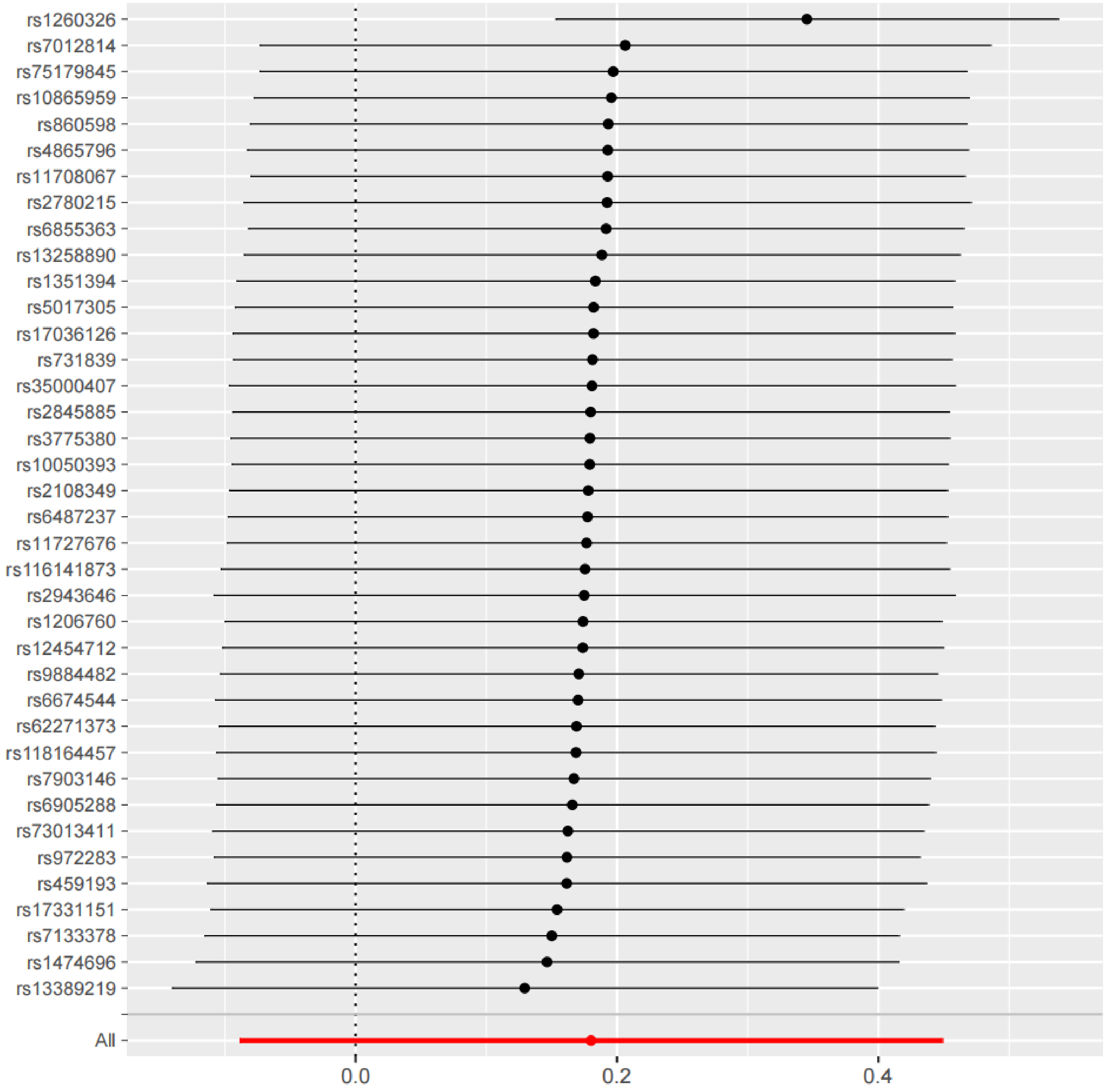

Supplementary Figure 18

MR leave−one−out sensitivity analysis for 'Fasting insulin' on 'Cholesterol in small LDL'

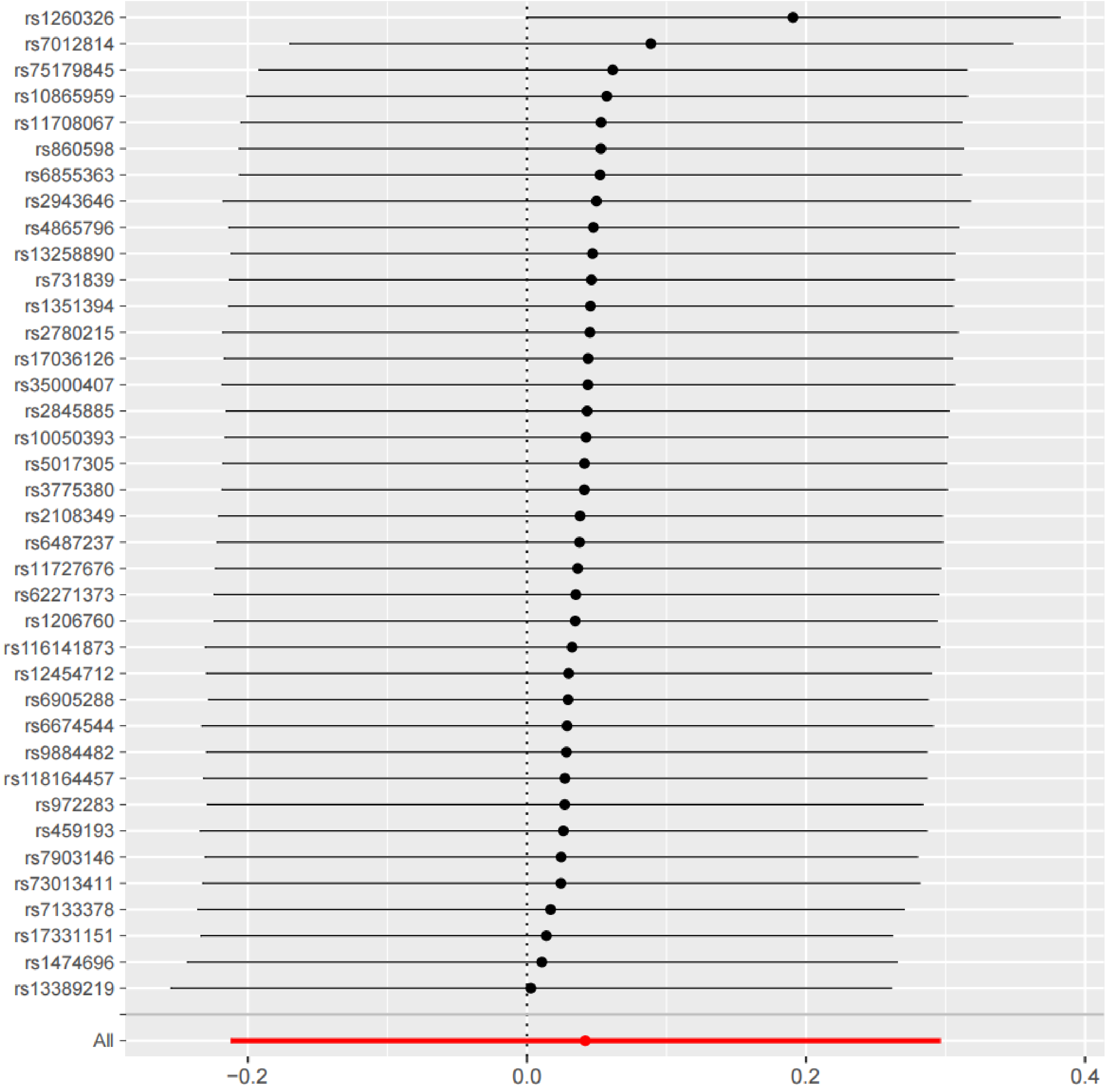

Supplementary Figure 19

MR leave−one−out sensitivity analysis for 'Hypertension' on 'Concentration of small LDL particles'

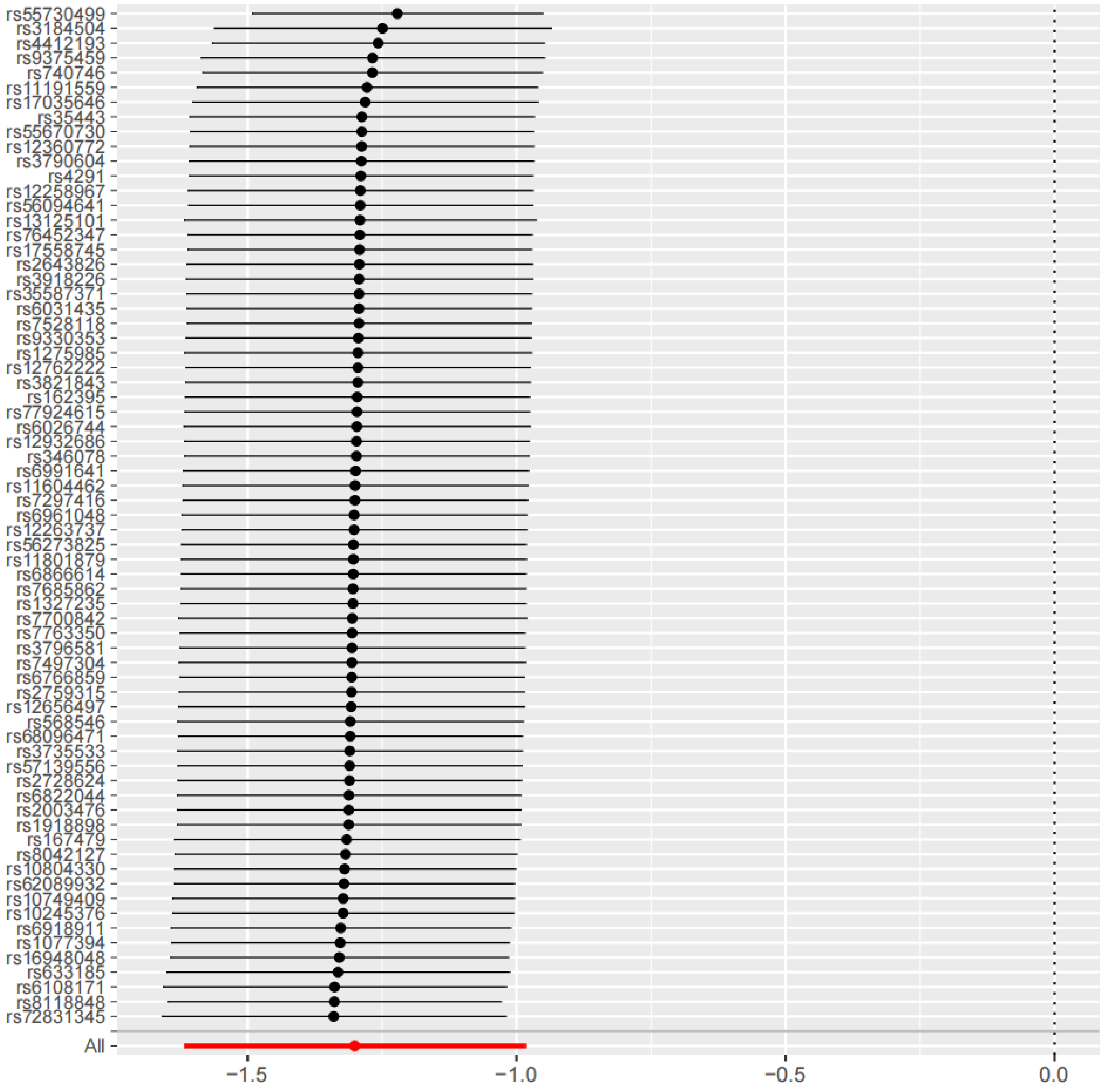

Supplementary Figure 20

MR leave−one−out sensitivity analysis for 'Hypertension' on 'Cholesterol in small LDL'

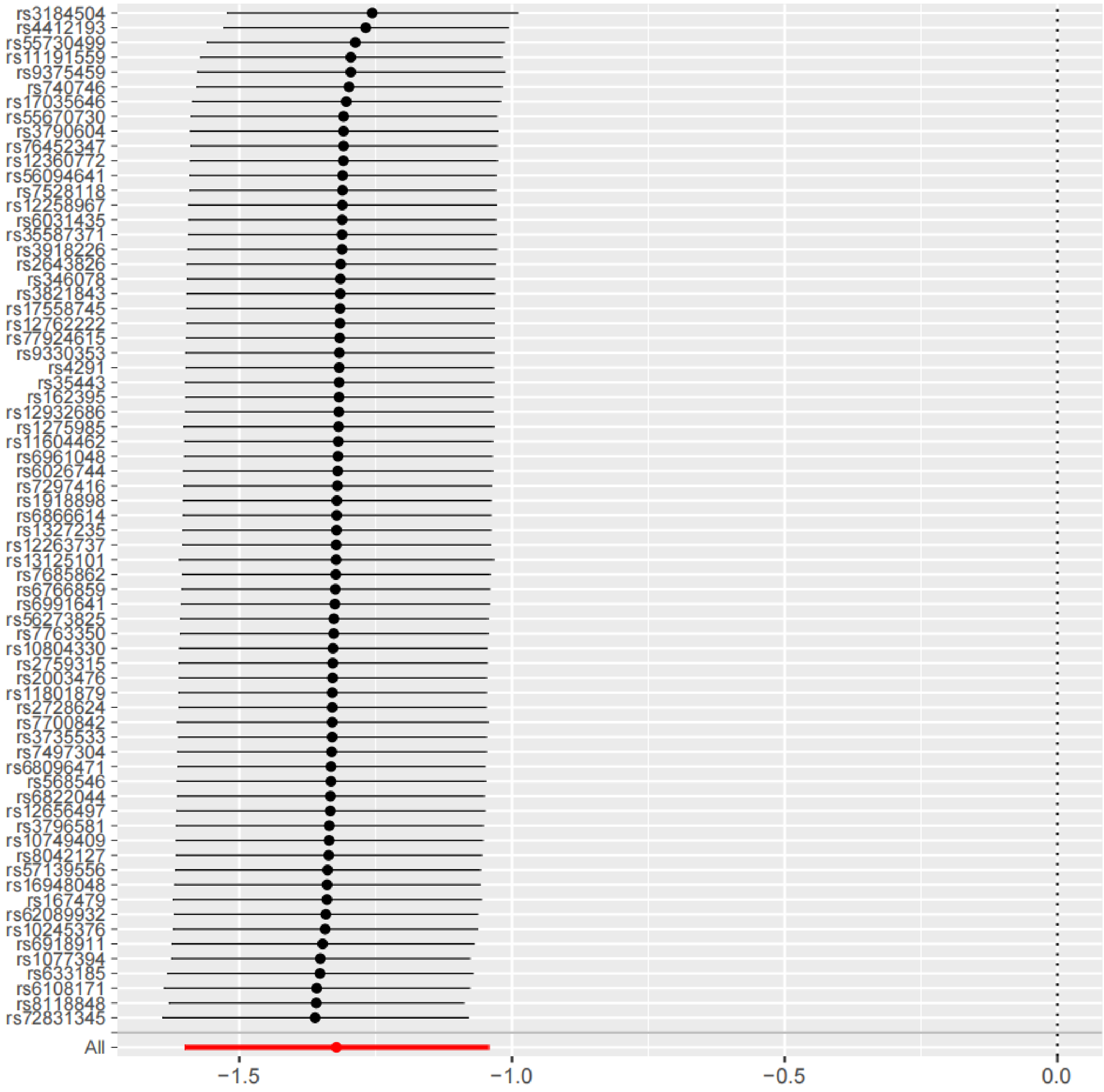

Supplementary Figure 21

MR leave−one−out sensitivity analysis for 'HDL cholesterol' on 'Concentration of small LDL particles'

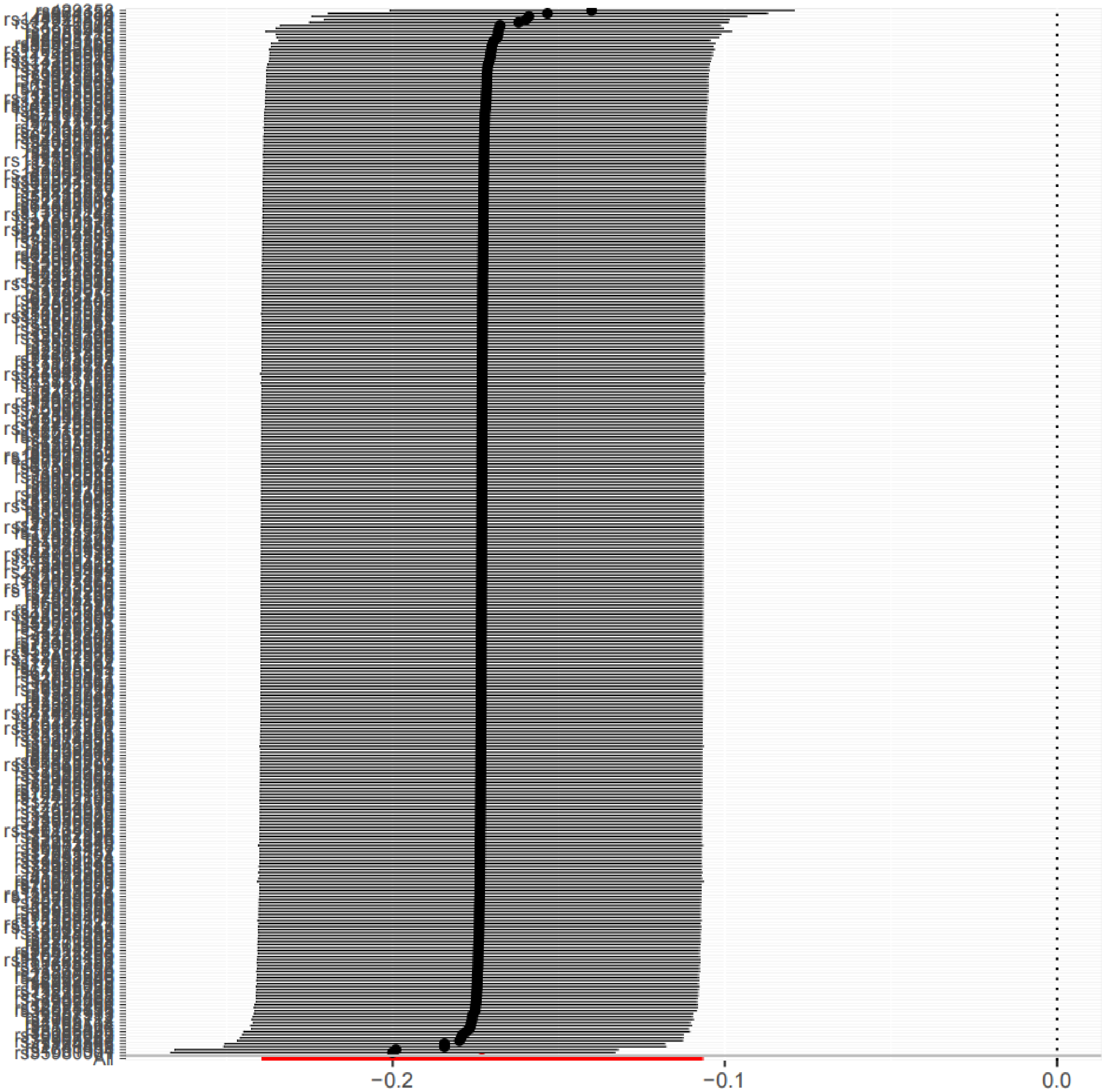

Supplementary Figure 22

MR leave−one−out sensitivity analysis for 'HDL cholesterol' on 'Cholesterol in small LDL'

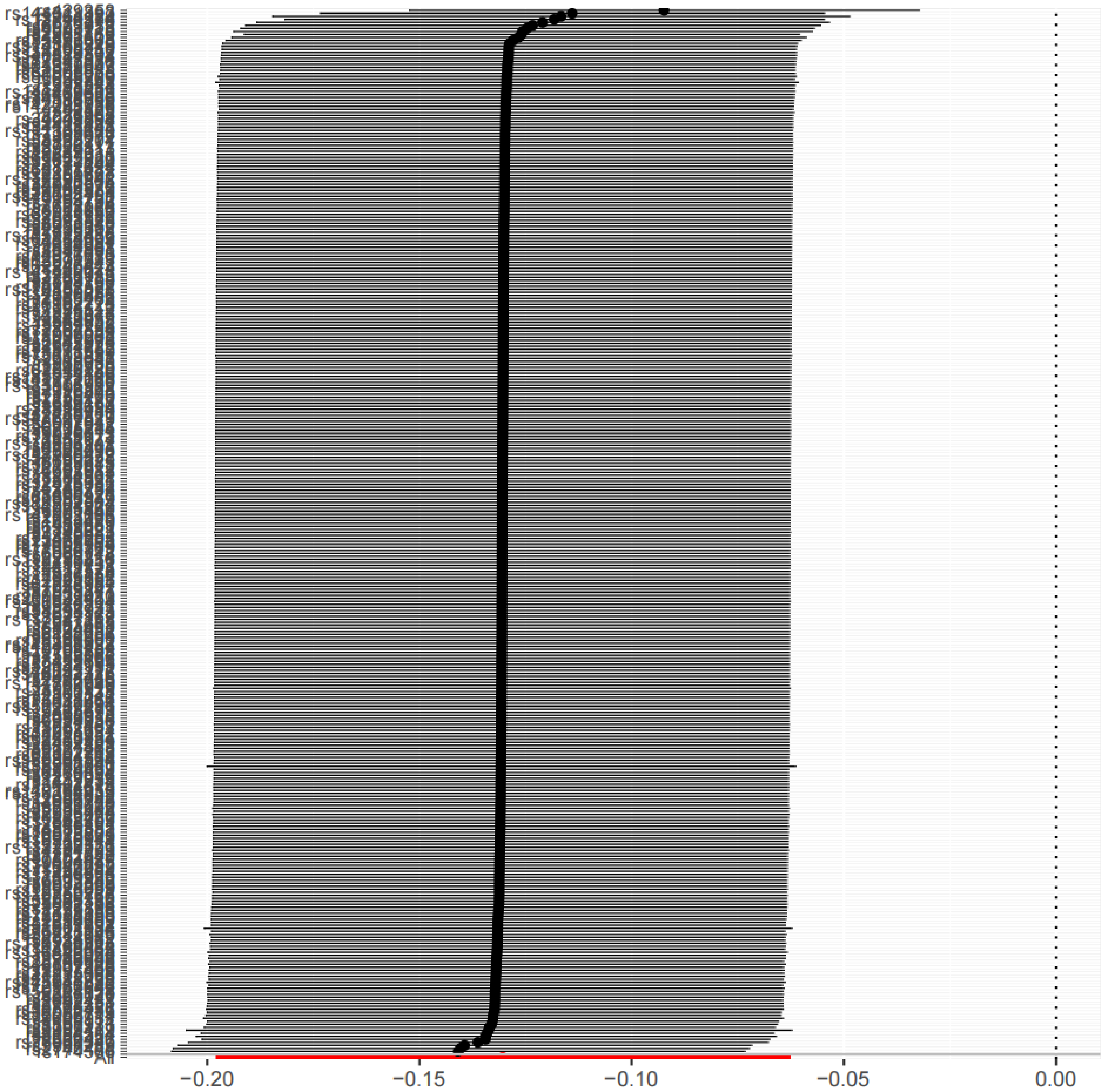

Supplementary Figure 23

MR leave−one−out sensitivity analysis for 'LDL cholesterol' on 'Concentration of small LDL particles'

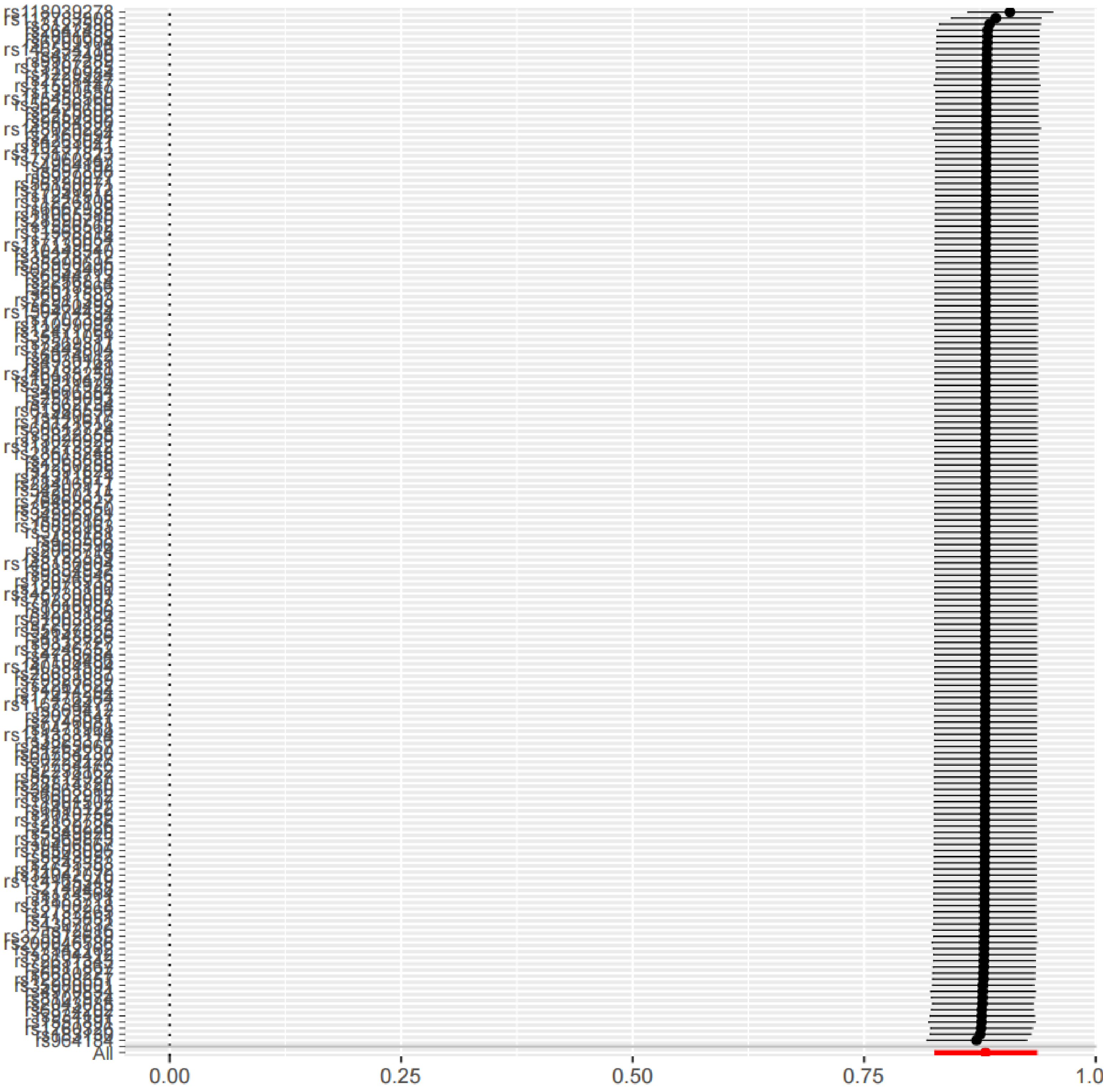

Supplementary Figure 24

MR leave−one−out sensitivity analysis for 'LDL cholesterol' on 'Cholesterol in small LDL'

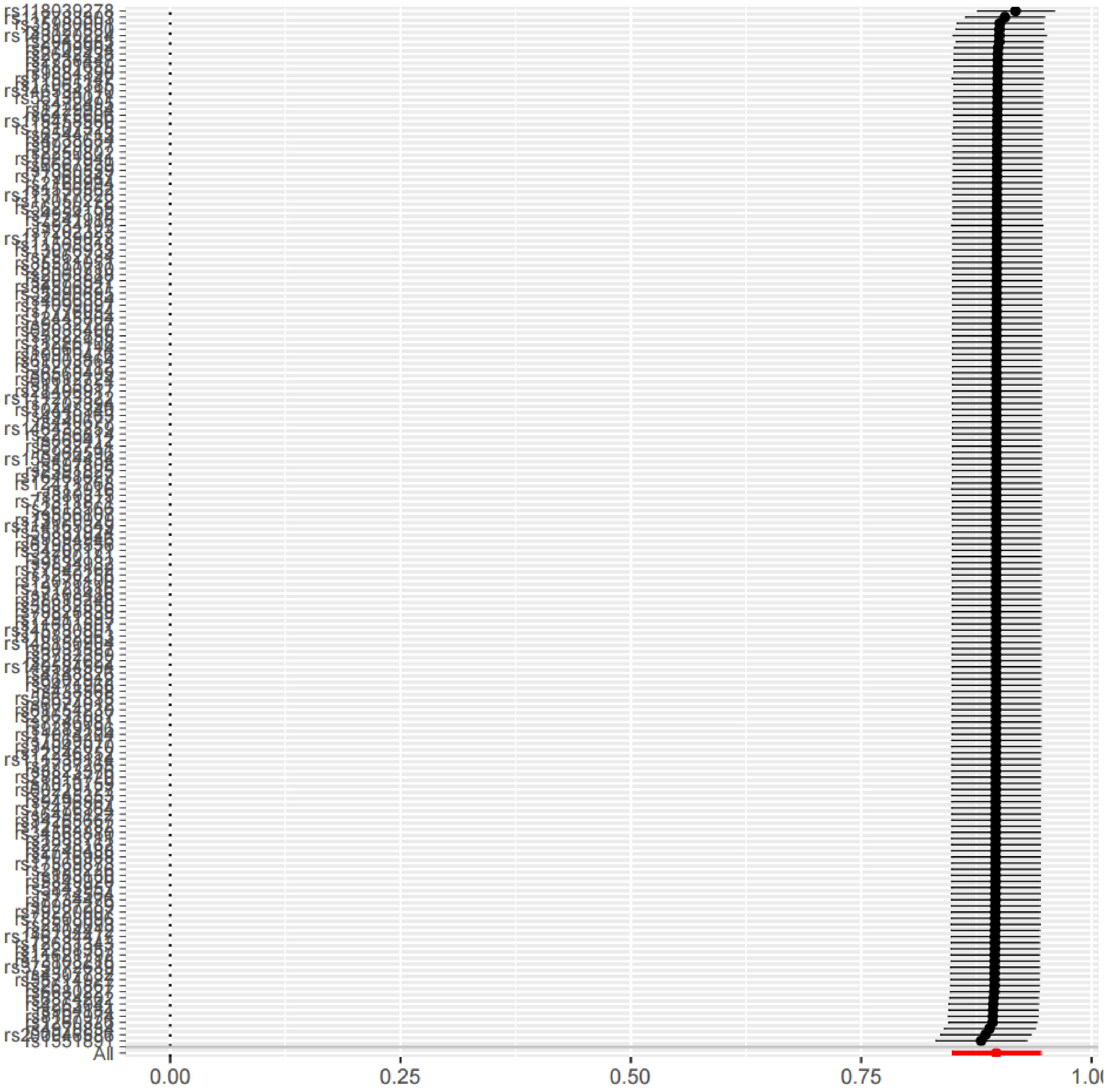

Supplementary Figure 25

MR leave−one−out sensitivity analysis for 'Triglycerides' on 'Concentration of small LDL particles'

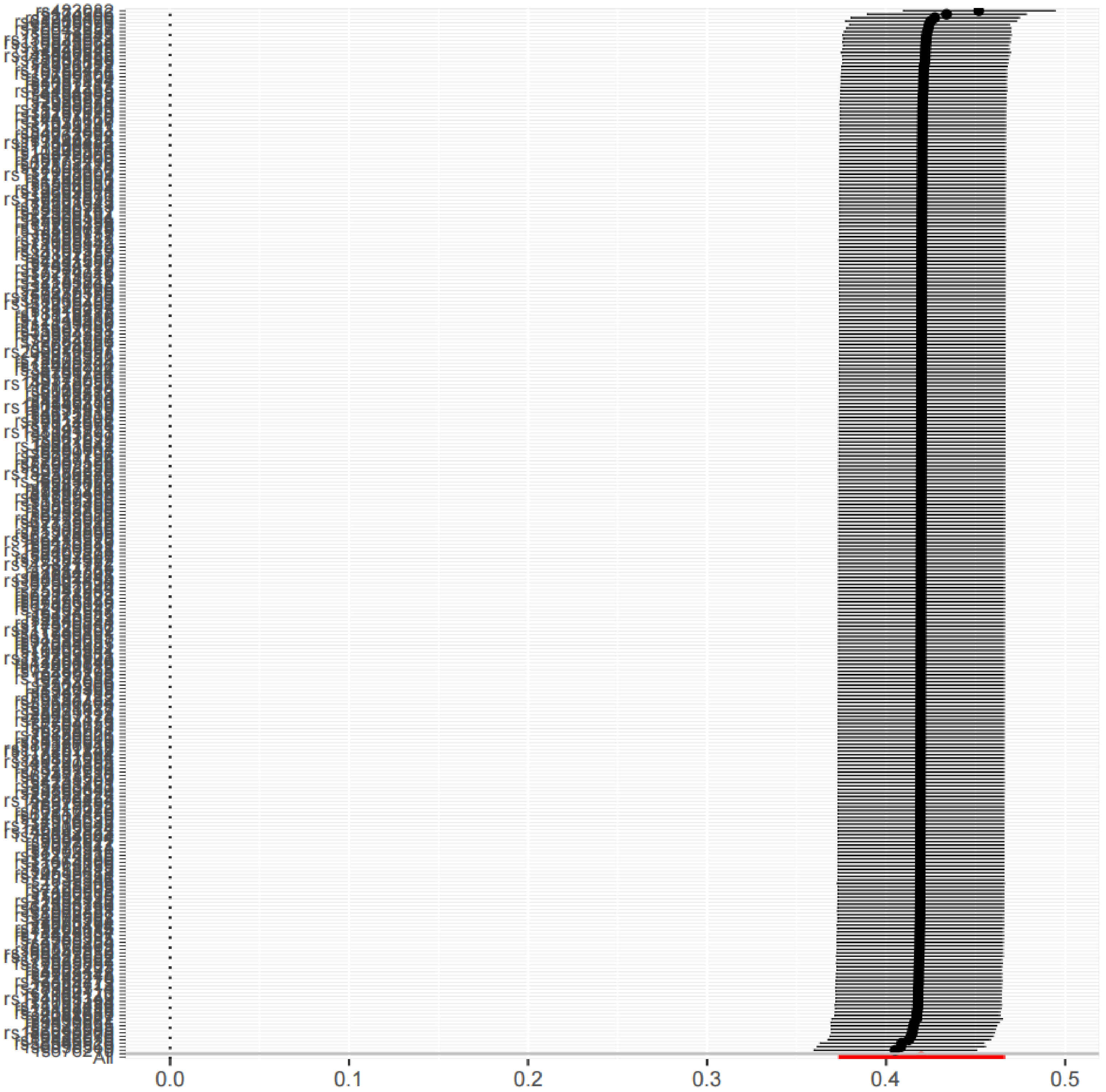

Supplementary Figure 26

MR leave−one−out sensitivity analysis for 'Triglycerides' on 'Cholesterol in small LDL'

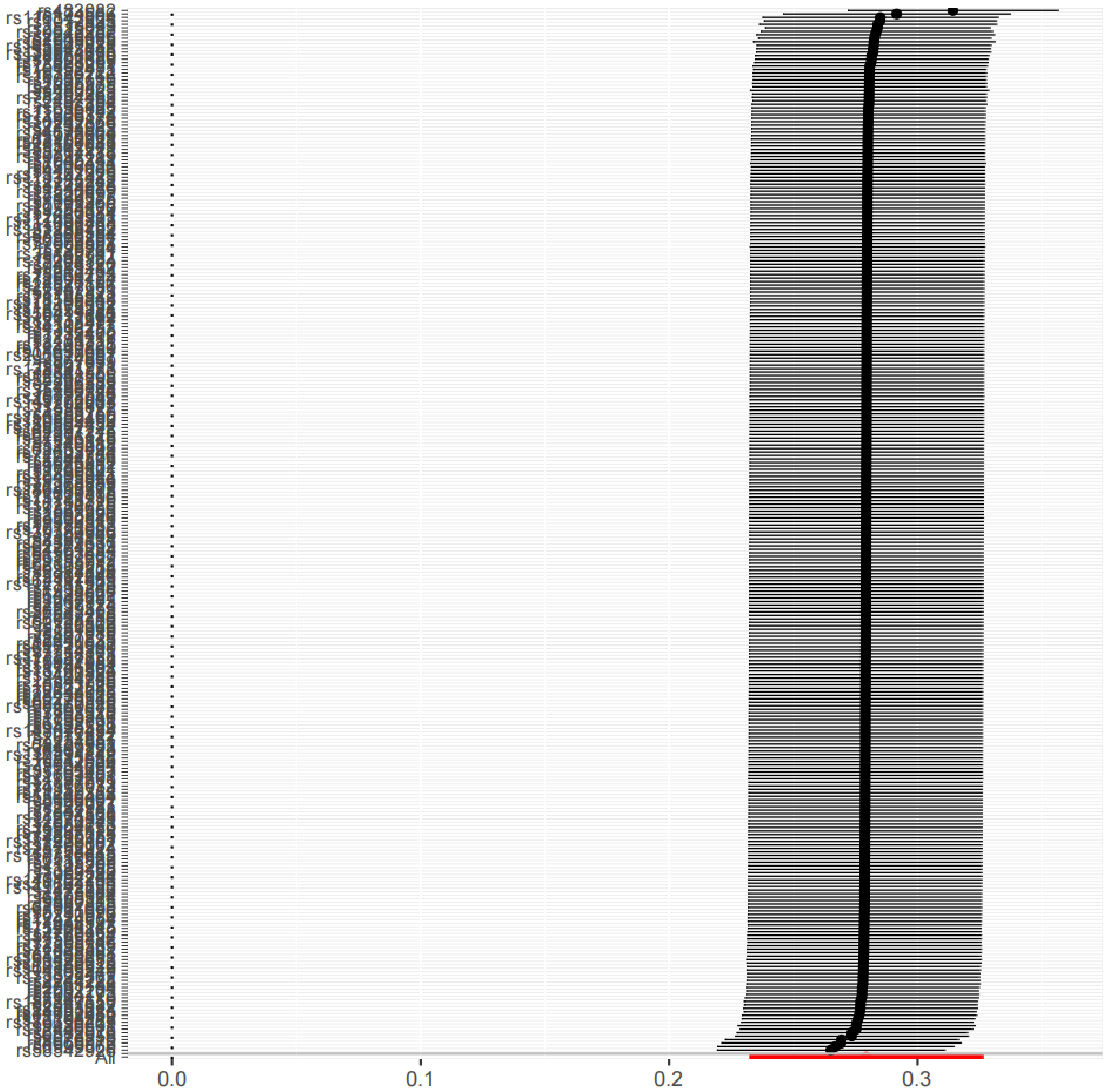

Supplement: S1 File — (PDF) [file pone.0298070.s012.pdf]
